# Modular Synthesis of a Lignin Oligomer Library

**DOI:** 10.1002/cssc.70939

**Published:** 2026-08-08

**Authors:** Eman Abdelraheem, Tom A. Ewing, Gijs van Erven, Fedor M. Miloserdov

**Affiliations:** ^1^ Laboratory of Organic Chemistry Wageningen University Wageningen The Netherlands; ^2^ Wageningen Food & Biobased Research Wageningen The Netherlands; ^3^ Laboratory of Food Chemistry Wageningen University Wageningen The Netherlands

**Keywords:** bromoketoesters, heteronuclear single quantum coherence (HSQC) NMR, lignin analysis, lignin model compounds, modular organic synthesis

## Abstract

We report a modular and scalable synthetic strategy for constructing a diverse library of oligomeric lignin model compounds (LMCs). Using bromoketoester building blocks, iterative β‐*O‐*4 aryl ether couplings under mild conditions enabled the efficient assembly of dimers, trimers, and tetramers. The developed approach is compatible with H, G, and S lignin units and can be extended to cinnamyl alcohols and β‐5 linkages, mimicking the structural diversity of native lignin. Linear and convergent routes were demonstrated, and total reductions afforded final LMCs representative of lignins from various botanical sources. Our platform and advanced LMC library will allow studying lignin chemistry in unprecedented detail, bridging the gap between currently available dimers and actual polymeric lignin.

## Graphical abstract

**Bridging the gap** between commercial lignin dimer models and actual polymeric lignin by the modular synthesis of a lignin oligomer library.

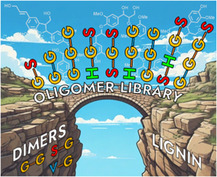

## 1 Introduction

Lignin is the most abundant natural source of aromatic compounds and holds tremendous potential for applications in the chemical, pharmaceutical, and material industries [1]. Despite this potential goldmine status, lignin remains significantly underutilized and is often burned merely for energy recovery [2]. This underutilization is primarily due to lignin’s notoriously complex and naturally diverse molecular structure [3, 4]. Recent advances in lignin research have relied heavily on lignin model compounds (LMCs), low molecular weight molecules that structurally mimic polymeric lignin, thus circumventing its overwhelming molecular complexity and the associated analytical challenges [5]. LMCs have enabled detailed studies of reactivity, selectivity, kinetics, and mechanisms, allowing researchers to pinpoint observations to individual structural motifs [6, 9]. However, there remains a significant gap in the availability of suitable LMCs. On the one hand, the very few commercially available LMCs are β‐*O*‐4 dimers that do not adequately represent lignin’s diverse building blocks and reactivity [9, 11]. On the other hand, existing synthetic methods for more advanced lignin model oligomers are labor‐intensive, typically yield only single target molecules, and increasing complexity is logically accompanied by lower yields [5, 12, 17]. Even more so, if all relevant subunits and interunit linkages were to be incorporated in a single (model) molecule to properly represent the “average” structural diversity of lignin, one ends up with polymeric lignin itself. In fact, lignin is a population of structures differing in the composition and sequence of subunits and interunit linkages, depending on the botanical source [2, 4, 18]. We would therefore argue that no single LMC, regardless of its complexity, can sufficiently represent the structural diversity of lignin. To advance the field, ***libraries*** of lignin model compounds are required.

**SCHEME 1:**
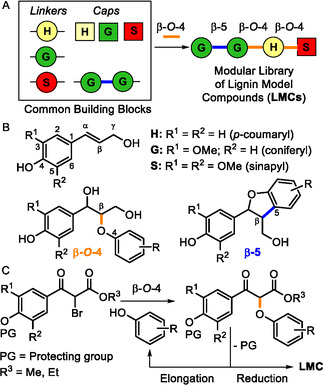
(A) Modular synthesis of LMCs. (B) Key monolignol units and linkages. (C) Construction of β‐*O*‐4 linkages via bromoketoesters.

To access a library of LMCs, a synthetic strategy must be developed with the following properties: 1) be modular [19], enabling the stepwise and controlled assembly of complex LMCs from readily available building blocks in an iterative stepwise growth (ISG) approach (Scheme 1A) [20], 2) use building blocks structurally related to the three primary monolignol units (H, G, and S, Scheme 1B), and 3) connect the building blocks through different types of interunit linkages. The β‐*O*‐4 aryl ether linkage is by far the most prevalent in natural lignin, accounting for 45–84% of total linkages [4, 21], and it was therefore selected as the main connectivity motif in our modular assembly. Importantly, other linkage types such as β‐5 (3–12% of native lignin linkages) [4, 21] can also be incorporated into a β‐*O*‐4 sequence by embedding them within pre‐synthesized building blocks.

Several synthetic strategies have been developed for the construction of β‐*O*‐4 linkages [5, 10, 12, 17, 22, 24] with the aldol‐based approaches being the most widely used [5]. While highly efficient for dimer synthesis, extending this method to trimers is considerably more challenging. For example, in a conventional synthetic route [13], preparing a single β‐*O*‐4 trimer requires a 9‐step linear sequence with multiple protection and deprotection steps. Because the synthesis is not modular, preparing an additional β‐*O*‐4 trimer requires another 7–9 step sequence, making this approach highly impractical.

An alternative method to make β‐*O*‐4 aryl ethers is based on coupling of bromoketoesters with phenols, a strategy that has been applied in the synthesis of artificial lignin polymers and symmetric lignin oligomers [14, 16]. Recognizing that bromoketoesters can serve as readily accessible building blocks of H, G, and S monolignol units and that with an appropriate protecting‐group strategy chain elongation can be achieved in only 1–2 steps (Scheme 1C), we here set out to employ this approach to establish the first modular ISG‐based strategy for synthesizing a diverse library of LMCs.

## 2 Results and Discussion

**SCHEME 2:**
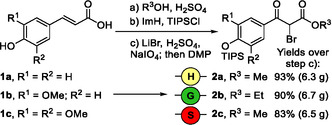
Synthesis of building blocks **2a‐c.** Abbreviations: ImH, imidazole; TIPSCl, triisopropylsilylchloride; DMP, Dess‐Martin periodinane.

We began our study by synthesizing the common **─** **H** **─**, **─** **G** **─**, and **─** **S** **─** building blocks featuring the following structural motifs: i) a bromoketoester moiety at one end to enable β‐*O*‐4 aryl ether formation via coupling with phenols; ii) a protected phenolic group at the other end, allowing for ISG of lignin oligomers upon deprotection; and iii) a central aromatic unit bearing zero, one or two OMe groups to mimic the H, G and S units of lignin. All building blocks were designed to be readily accessible on multigram scale. While a common route to bromoketoesters involves the reaction of acetophenones with dimethylcarbonate/NaH followed by bromination with bromine [25], we found an alternative approach more convenient, using hydroxycinnamic acids **1a‐c** as starting materials (Scheme 2). Following protection of the carboxylic acid and phenol groups [26], cinnamic double bonds were selectively converted into corresponding bromohydrines by LiBr/NaIO_4_/H_2_SO_4_ [27]. Subsequent oxidation with Dess‐Martin periodinane (DMP) in the same pot provided bromoketoesters **2a‐c** in excellent yields (83%–93%), resulting in >6 g of each of the key **─H** **─**, **─** **G** **─**, and **─** **S** **─** building blocks [28]. Notably, this protocol avoids the use of both NaH and elemental bromine.

Next, we focused on optimizing conditions for construction of the β‐*O*‐4 aryl ether linkage and subsequent removal of the TIPS (triisopropylsilyl) protecting group. Initially, we evaluated three phenol‐protecting groups: TMS (trimethylsilyl), TIPS, and benzyl. TMS proved too labile, while the benzyl group required an additional, separate deprotection step. In contrast, TIPS offered the ideal balance of stability and reactivity. It remained intact during the coupling with phenolates under basic conditions but could be cleaved in the same pot, either by extending the reaction time or by simply adding tetra‐*n*‐butylammonium fluoride (TBAF). Additionally, the TIPS group significantly altered the polarity of the coupled products, which proved advantageous for isolation and purification in later steps (vide infra).

**SCHEME 3:**
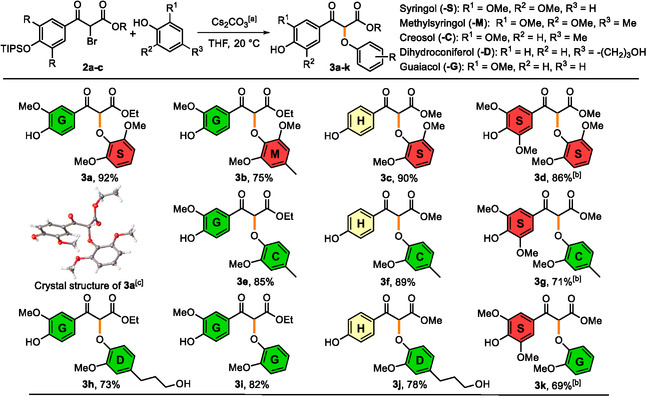
Synthesis of β‐*O*‐4 ketoester dimers **3a–k** and isolated yields. Reaction conditions: (a) **2a‒c** (0.21 mmol; 1 equiv.), phenols (1.2 equiv.), Cs_2_CO_3_ (3.0 equiv.) in dry THF (100 mM), N_2_, r.t., ~3–10 h. (b) After ~2 h TBAF·xH_2_O was added. (c) Crystal structure of **3a** with thermal ellipsoids set to a 50% probability level.

The model β‐*O*‐4 coupling between **2a** and bulky syringol, carried out under reported conditions (K_2_CO_3_, acetone, reflux, 6.5 h) [15], produced a protected dimer **3a‐TIPS** in a good isolated yield of 83% (Table S1). However, this protocol had two notable drawbacks: a prolonged reaction time at reflux and the use of 2 equiv. of the phenol nucleophile, which would become a limiting reagent in the synthesis of longer oligomers. To increase reactivity of the phenolate, we exploited the well‐established ‘‘cesium effect’’ [29, 30]. Previous studies have shown that Cs_2_CO_3_ in DMF, or the more synthetically convenient solvent THF, dramatically enhances reactivity of phenols toward alkylation [31, 32]. By using Cs_2_CO_3_ in THF, we improved the isolated yield to 89%, while reducing the reaction time to a few hours (2–5 h) at room temperature, using only 1.2 equiv. of syringol (Table S1). Moreover, upon completion of the β‐*O*‐4 coupling, partial deprotection of the TIPS group was observed in the same reaction mixture. Increasing the reaction time to 5.5 h led to complete deprotection, and the dimer **3a** was isolated in an excellent yield of 92% (Scheme 3). Although Cs_2_CO_3_‐mediated cleavage of *tert*‐butyldimethylsilyl (TBS)‐protected phenols has been described in the literature [33], these transformations are typically performed in the presence of water. Therefore, the deprotection observed under water‐free conditions employed here (anhydrous THF) was unexpected, yet convenient. Conversion and deprotection occur as subsequent events (90% conversion before deprotection; Table S1).

Next, we explored the first step in the modular lignin oligomer synthesis—the coupling of bromoketoesters **2a‐c** (representing lignin units in Scheme 1A) and various phenols (serving as phenolic caps) to produce capped dimers **3** (Scheme 3). We focused on phenols structurally representative of native lignin, including syringol (**─S**, **3a, c‐d**), 4‐methylsyringol (**─M**, **3b**), creosol (**─C**, **3e‐g**), dihydroconiferyl alcohol (**─D**, **3h, j**), and guaiacol (**─G**, **3i, k**). Using these phenolic caps establishes a defined initiation point for end‐wise coupling, mirroring the directional growth observed in native lignin biosynthesis.

For bromoketoesters **2a** and **2b** (corresponding to **─H**─ and **─G**─ units), the β‐*O*‐4 coupling with various phenols proceeded efficiently, followed by in situ TIPS deprotection in a sequential one‐pot process (~2 and ~4–10 h, respectively), affording the desired dimers **3** in 73%–92% isolated yields. For the syringyl‐derived bromoketoester **2c** (**─S**─ unit), TIPS deprotection did not proceed under Cs_2_CO_3_/THF conditions, necessitating the addition of TBAF. This way, the deprotected dimers **3d**, **3g**, and **3k** were obtained in 86%, 71%, and 69% yields, respectively. The transformation was generally well tolerated, even with sterically hindered phenols, as exemplified by the high yield obtained with syringol (92% for **3a**). The presence of a primary hydroxy group in dihydroconiferyl alcohol was also compatible with the reaction conditions, affording dimers **3h** and **3j** in 73 and 78% yields. The structures of dimers **3a** and **3i** were confirmed by single‐crystal X‐Ray diffraction (Scheme 3, Supporting Information) [34].

**SCHEME 4:**
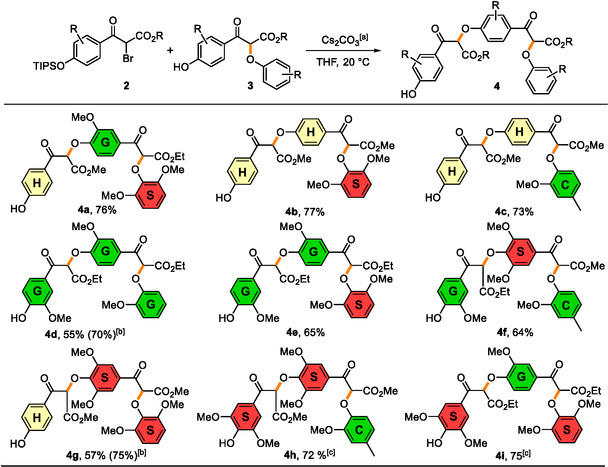
Synthesis of ketoester β‐*O*‐4 trimers **4a–i** and isolated yields. Reaction conditions: (a) dimers **3** (0.10 mmol; 1.0 equiv), **2a‒c** (1.5 equiv.), Cs_2_CO_3_ (3.0 equiv.) in dry THF (100 mM, 5%v/v DMF), N_2_, r.t., ~4–12 h. (b) Isolated yield after recovering the unreacted dimer. (c) After ~2 h, TBAF·xH_2_O was added.

The dimers **3** were subsequently employed as the phenolic component in couplings with bromoketoesters **2** to afford trimers **4** (Scheme 4). The reaction between **3a** and **2a** was used as a model reaction. In this case, dimer **3a** was used as the limiting reagent, with bromoketoester **2a** added in 1.5 equiv. excess. Although **2a** was fully consumed, only ~90% conversion of **3a** was observed by TLC. Adding to the reaction another 0.5 equiv. of **2a** improved the yield by only 4%. Under these conditions (1.5 equiv. of **2a‐c**), trimers **4a‐i** were obtained in 64%–77% yield as mixtures of two diastereomers (Supporting Information). For trimers **4d** and **4g**, the conversion of the starting phenols was incomplete, and ~20% unreacted starting material was recovered. For trimers **4h** and **4i**, which contain a terminal **─S**─ unit, TBAF was added to facilitate the TIPS deprotection. It is worth noting that all trimers **4** were obtained as 1:1 mixture of diastereomers, indicating a lack of diastereocontrol during the β‐*O*‒4 coupling step.

**SCHEME 5:**
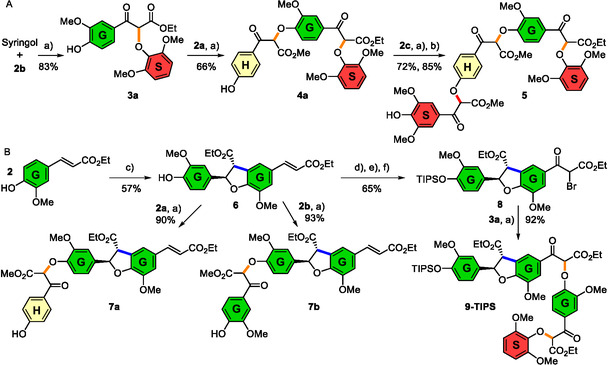
(A) Synthesis and isolated yields of β‐*O*‐4 tetramer **5** and intermediates **3a**, **4a**. (B) Synthesis and isolated yields of β‐5 building blocks **6** and **8**, β‐*O*‐4/β−5 trimers **7a, b** and tetramer **9‐TIPS**. Key reagents and conditions: a) Cs_2_CO_3_, dry THF, r.t.; b) TBAF·xH_2_O; c) Ag_2_O, dry DCM, r.t.; d) ImH, TIPSCl, dry DCM, r.t.; e) NBS, DPTU, *t*‐BuOH/H_2_O, r.t.; f) DMP, dry DCM, r.t.

Satisfied with the performance and scope of the β‐*O*‐4 coupling, we proceeded with the synthesis of a tetrameric LMC precursor **5** (Scheme 5A). This compound incorporates four primary lignin subunits (**─H** **─**, **─G** **─**, and two **─S** **─** units) coupled through three β‐*O*‐4 linkages. We scaled up the synthesis of dimer **3a** and trimer **4a** tenfold, isolating the corresponding products in 83% and 66% yield, respectively, on a > 1 g scale. Trimer **4a** was then coupled with compound **2c**, affording the tetramer **5**. Separation of **5** from unreacted starting material **4a** proved to be challenging. Therefore, we initially isolated **5** in its TIPS‐protected form (**5‐TIPS**), which was deprotected in a separate step to furnish tetramer **5**.

While the β‐*O*‐4 aryl ether is the most prevalent interunit linkage in native lignin, other interunit linkages, such as the β‐5 phenylcoumaran [4], also play key structural roles, particularly in softwood lignin. To showcase the versatility of our modular approach, we targeted the β‐5 motif by synthesizing the known β‐5 dimer **6** [35, 36], via oxidative phenol homocoupling with a 57% isolated yield without any optimization (Scheme 5B). Notably, dimer **6** was isolated as a single stereoisomer with trans‐orientation of the substituents on the dihydrobenzofuran ring, as confirmed by single‐crystal X‐ray diffraction (Supporting Information). We envisioned dimer **6** as a useful structural element for further oligomer construction, leveraging either the phenolic or alkene functionality, depending on the desired LMC sequence. Coupling of the phenolic group of **6** with bromoketoesters **2a** or **2b** under Cs_2_CO_3_/THF conditions furnished the β‐*O*‐4/β‐5 linked trimers **7a** and **7b** as an ~1:1 mixture of diastereomers, in 90%–93% isolated yield via a one‐pot, two‐step sequence (Scheme 5B, left and center).

Additionally, the alkene moiety of compound **6** was converted into the bromoketoester building block **8** via a concise three‐step sequence: TIPS‐protection of the phenol, hydroxybromination, and oxidation to the ketone (Scheme 5B, right). Initial attempts to generate the bromohydrin using LiBr/NaIO_4_/H_2_SO_4_ (previously successful for the synthesis of **2a‐c**, Scheme 2), resulted in significant formation of an undesired epoxide side product (confirmed by HLPC‐MS). Switching to *N*‐bromosuccinimide (NBS) [37] in the presence of diphenyl thiourea (DPTU) in *t*‐BuOH/H_2_O suppressed epoxide formation, improving the isolated yield from 58% to 86% at room temperature. Subsequent oxidation of the bromohydrin intermediate with DMP provided the desired bromoketoester **8** in 91% yield as 1:1 mixture of two diastereomers. This three‐step sequence afforded building block **8** in 65% overall yield (83%–91% yield of the individual steps), corresponding to 750 mg of isolated product. Bromoketoester **8** was then coupled with phenolic dimer **3a**, affording the tetrameric product. Due to the similar polarities of the unprotected tetramer and starting material **3a**, it proved more practical to isolate the product in its TIPS‐protected form, **9‐TIPS**. Tetramer **9‐TIPS**, featuring one β‐5 and two β‐*O*‐4 interunit linkages, was isolated in 92% yield as a 1:1:1:1 mixture of four diastereomers (Scheme 5B, right). The synthesis of a tetramer from two dimeric building blocks highlights the potential of our method to rapidly increase complexity through a convergent assembly approach.

**SCHEME 6:**
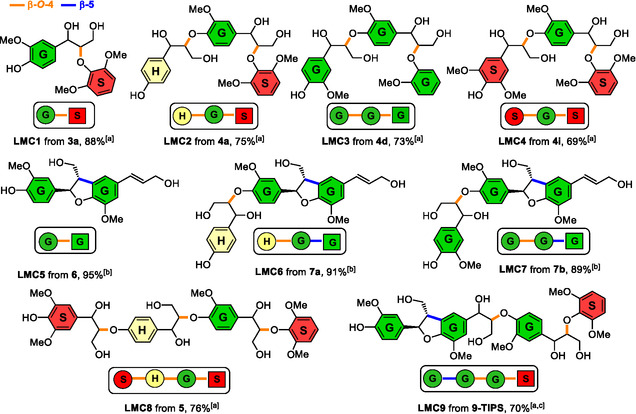
Lignin model compounds **LMC1‐9** obtained by reduction of keto esters, along with their isolated yields. Key reagents and conditions: [a] NaBH_4_, CHCl_3_/MeOH, 0 °C; [b] DIBAL‐H, dry THF, 0 °C; [c] TBAF·xH_2_O, THF, r.t.

Finally, total reduction of the keto‐ and ester groups formed the targeted lignin model compounds (Scheme 6). Most of the synthesized keto esters were readily reduced using excess of NaBH_4_ in CHCl_3_/MeOH [15], allowing the successful isolation of dimers, trimers (**LMC1‐4**), and even tetramers (**LMC8** and **LMC9**) in 68%–88% yield as mixtures of diastereomers. The cinnamic ester moiety present in compounds **6** and **7a, b** was unreactive toward NaBH_4_, but it was efficiently reduced together with other carbonyls using excess of DIBAL‐H [35, 38] in THF, affording **LMC5‐7** in 89–95% yield. Single‐crystal X‐Ray diffraction on **LMC5** (previously reported) [35] confirmed that the trans‐stereochemistry of substituents on the benzofuran ring was retained (Supporting Information).

**FIGURE 1:**
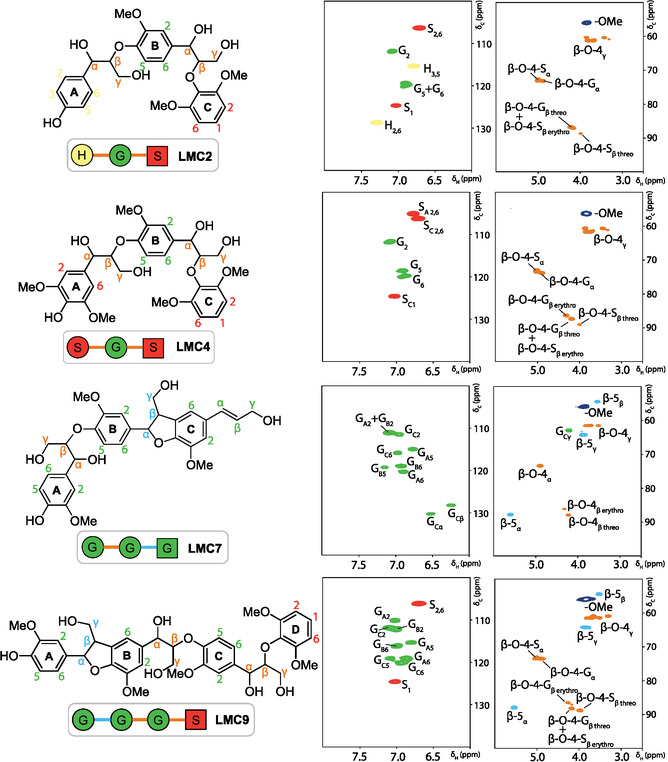
Aromatic and aliphatic regions of ^1^H‐^13^C HSQC NMR spectra of synthesized LMCs **2**, **4**, **7**, and **9**. β‐*O*‐4 and β‐5 linkages are presented in orange and light blue color, respectively.

2D ^1^H‐^13^C heteronuclear single quantum coherence (HSQC) NMR was performed for all LMC oligomers (Scheme 6, Figure 1). HSQC NMR again highlighted the structural variety of oligomers we synthesized and demonstrated the purity of the compounds. All peaks observed could be fully annotated (Figure 1 and Supporting Information). The aliphatic region resolved β‐*O*‐4 aryl ethers linked to different subunits (on the basis of the C_α_‐H_α_ signal) and different diastereomers of these linkages (on the basis of the C_β_‐H_β_ signal). In the aromatic region, H, G, S, and cinnamyl units could obviously be separated, but it is worthwhile to mention that also G or S subunits present in various parts of the LMC sequence could be distinguished, including phenolic, nonphenolic units, end‐caps, and guaiacyls part of a phenylcoumaran linkage [39, 40]. This will enable monitoring their chemical reactivities in subsequent LMC studies.

The developed synthetic route provides LMCs as a mixture of β‐*O*‐4 diastereomers without stereochemical control, which is advantageous for mimicking native lignin, in which the β‐*O*‐4 linkages are present as mixture of stereoisomers. Diastereoselective LMC conversion can be monitored through NMR and/or UHPLC analysis. When individual stereoisomers are required, they can be isolated by post‐synthesis via chiral chromatography separation. Moreover, the β‐ketoesters utilized in our synthetic routes represent promising intermediates for asymmetric hydrogenation via dynamic kinetic resolution [41, 43], enabling simultaneous control of the stereochemistry at both the α‐ and β‐positions of the β‐*O*‐4 linkage.

## 3 Conclusion

In conclusion, we have developed a modular and scalable strategy for the efficient construction of a structurally diverse library of lignin model compounds (LMCs). Utilizing readily accessible bromoketoester building blocks, our approach enables iterative β‐*O*‐4 aryl ether couplings followed by in situ or sequential TIPS deprotection, affording dimers, trimers, and tetramers in 64%–93% yield per coupling step. The method is compatible with all three primary lignin monomeric units (H, G, and S) and was further extended to include β‐5 interunit linkages and/or cinnamyl alcohols, highlighting its flexibility in mimicking the structural complexity of native lignin. Both linear and convergent assembly routes were demonstrated, enabling the integration of multiple linkage types within a single molecule. Total reduction of the oligomers furnished final LMCs (**LMC1–9**).

Importantly, the LMC library generated through this approach can be tailored to reflect lignin’s botanical source. For instance, G‐type compounds such as **LMC3**, **LMC5**, and **LMC7** serve as **softwood** models; S/G‐type **LMC1**, **LMC4**, and **LMC9** are suited for **hardwood**; while **LMC2**, **LMC6**, and **LMC8** incorporate H‐units, making them more suitable for modeling lignin from **grasses**.

This work provides a versatile platform to access advanced LMCs, a valuable tool for future studies on lignin structure, reactivity, and valorization. Our ongoing efforts aim to expand the number and variety of interunit building blocks (e.g., β‐β, 5‐5) as well as explore its adaptation to solid‐phase synthesis for further streamlining, automation, and upscaling. Further studies leveraging these lignin model compounds in diverse application areas are currently underway, including studies building on recent advances in understanding new lignin‐active enzymes [44, 45], and we look forward to sharing those results in due course.

## Funding

This work was supported by the VLAG Graduate School, the Dutch Ministry of Education—Sector Plan Beta for Science and Technology, and Regiodeal Foodvalley.

## Conflicts of Interest

The authors declare no conflicts of interest.

## Supporting information

The authors have cited additional references within the Supporting Information [35, 46, 54].

## Data Availability

The data that supports the findings of this study are available in the Supporting Information of this article.
