# Supplementary material for: Modular Synthesis of a Lignin Oligomer Library
Source: ChemSusChem. 2026 Aug 8;19(15):e70939. doi: 10.1002/cssc.70939 (PMC13452564; doi:10.1002/cssc.70939)
Supplement: Supplementary file 1 [file CSSC-19-e70939-s001.pdf]

Modular Synthesis of a Lignin Oligomer Library

SUPPORTING INFORMATION

Eman Abdelraheem, Tom A. Ewing, Gijs van Erven, and Fedor M. Miloserdov

General Experimental Information S2

Synthesis of Building Blocks -H- (2a), -G- (2b), and -S- (2c) S4

Step 1: Synthesis of Hydroxycinnamates Esters. S4

Step 2: Synthesis of TIPS-Protected Hydroxycinnamate Esters. S5

Step 3: Synthesis of Bromoketoesters **2a-c** S6

Optimization of β-*O*-4 Coupling S9

Synthesis of Oligomers with β-*O*-4 Linkages S10

Synthesis of dimers 3a-k S10

Synthesis of trimers 4a-i S19

Synthesis of tetramer 5 .... S25

Synthesis of Oligomers with β-5 Linkage S27

Reduction of Ketoesters into LMC1-9 .... S33

NMR Spectra of Synthesized Compounds S40

References S90

General Experimental Information

Starting materials, reagents, and solvents were purchased from commercial suppliers and used without further purification unless otherwise noted. Tetrabutylammonium fluoride hydrate ($Bu_4NF·xH_2O$) was obtained from Sigma-Aldrich (CAS no. 22206-57-1). Anhydrous dichloromethane (DCM), and anhydrous tetrahydrofuran (THF) were collected from a PureSolv Solvent Purification System (Innovative Technology).

The following abbreviations are used for solvents: ethyl acetate (EtOAc), methanol (MeOH), *tert*-butanol (*t*-BuOH), and petroleum ether 40-60 (PE). All solvents were used as received, without additional distillation.

Coupling reactions and reduction with DIBAL-H were performed under anhydrous conditions using oven-dried glassware. Glassware was heated at temperatures above 100 °C overnight, then allowed to cool, connected to nitrogen line, and flushed with nitrogen gas for at least 15 min after weighing any solids.

Reactions were monitored by thin layer chromatography (TLC) on alumina-backed silica gel plates. TLC plates were visualized under UV light (254 nm) or stained with a solution of $KMnO_4$. Flash column chromatography was conducted using a PuriFlash 5.020 automated system, with silica columns of varying sizes depending on the reaction scale.

^{1}H NMR and ^{13}C NMR spectra were recorded at 298 K on a Bruker Avance 400 MHz spectrometer. Chemical shifts were references to the residual signals as follows: $CDCl_3$ (δ = 7.26 ppm for ^{1}H, 77.16 ppm for ^{13}C), DMSO-d_6 (δ = 2.50 ppm for ^{1}H, 39.52 ppm for ^{13}C), and acetone-d_6 (δ = 2.05 ppm for ^{1}H, 29.84 ppm for ^{13}C). The following abbreviations are used in reporting NMR data: chemical shift (δ, ppm), multiplicity (s = singlet, d = doublet, t = triplet, q = quartet, dd = doublet of doublet and m = multiplet), and coupling constant (*J*, Hz). High-resolution mass spectra (HRMS) were acquired using a Thermo Scientific Q Exactive Focus Mass Spectrometer with electrospray ionization (ESI) in either positive (ESI^+) or negative (ESI^-) mode, using MeOH as the solvent.

Crystallographic data for compounds **3a**, **3i**, **6** and **LMC5** were collected on a Rigaku XtaLAB Synergy-I instrument using a Cu Kα source. Experiments were conducted at 100 K, solved using SHELXT,[46] and refined using SHELXL[47] within Olex2 interface[48]. Certain chemicals,

including DIBAL-H, H_2SO_4, and $NaBH_4$, are highly reactive and hazardous materials that should only be handled by properly trained personnel following established laboratory safety protocols. These substances may pose significant risks such as fire, explosion, severe chemical burns, toxic exposure, and dangerous reactions with water, air, acids, oxidizing agents, or other incompatible materials. It is strongly recommended to carefully review the relevant Safety Data Sheet (SDS) prior to handling or use.

Synthesis of Building Blocks -H- (2a), -G- (2b), and -S- (2c)

Step 1: Synthesis of Hydroxycinnamates Esters.

General procedure 1.[49] Hydroxycinnamic acid (100 mmol) was dissolved in an excess of methanol or ethanol (300 mL). Concentrated H_2SO_4 (25 mmol, 1.3 mL) was added dropwise at 0 °C. The reaction mixture was then allowed to warm to room temperature and then refluxed, with progress monitored by TLC. After 12 h, the reaction mixture was concentrated under reduced pressure to remove the solvent, The resulting residue was washed with MeOH (40 mL × 3) and dried under reduced pressure to afford the corresponding methyl or ethyl ester product.

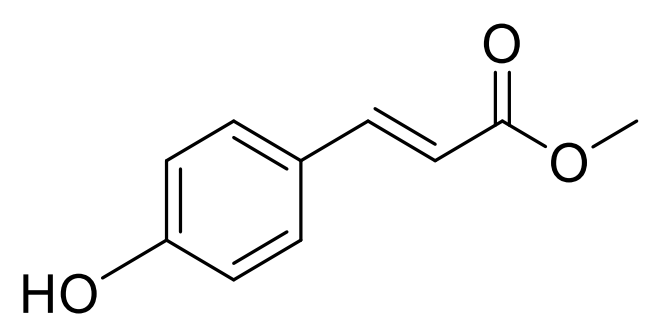

Methyl (*E*)-3-(4-hydroxyphenyl)acrylate was following the **general procedure 1** using (*E*)-3-(4-hydroxyphenyl)acrylic acid (100 mmol, 16.4 g) as the starting material. The title compound was obtained as a yellow solid (17.0 g, 96% yield). This compound has been previously characterized.[50] ^{1}H NMR (400 MHz, DMSO-d_6) δ 7.58 – 7.52 (m, 3H), 6.80 (d, *J* = 8.6 Hz, 2H), 6.38 (d, *J* = 16.0 Hz, 1H), 3.68 (s, 3H).

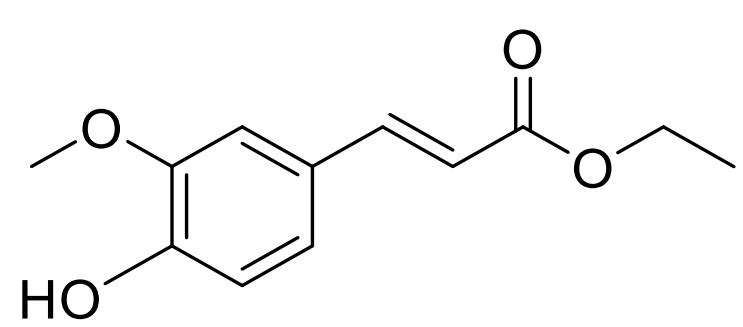

Ethyl (*E*)-3-(4-hydroxy-3-methoxyphenyl)acrylate was synthesized following the **general procedure 1** using (*E*)-3-(4-hydroxy-3-methoxyphenyl)acrylic acid (100 mmol, 19.4 g) as the starting material. The title compound was obtained as a white solid (21.5 g, 97% yield). This compound has been previously characterized.[51] ^{1}H NMR (400 MHz, $CDCl_3$) δ 7.61 (d, *J* = 15.9 Hz, 1H), 7.08 – 7.02 (m, 2H), 6.91 (d, *J* = 8.1 Hz, 1H), 6.29 (d, *J* = 15.9 Hz, 1H), 5.93 (s, 1H), 4.25 (q, *J* = 7.1 Hz, 2H), 3.91 (s, 3H), 1.33 (t, *J* = 7.1 Hz, 3H).

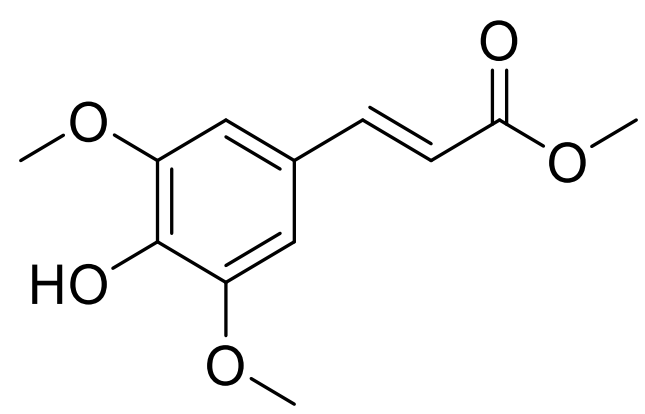

Methyl (*E*)-3-(4-hydroxy-3,5-dimethoxyphenyl)acrylate was synthesized following the **general procedure 1** using (*E*)-3-(4-hydroxy-3,5-dimethoxyphenyl)acrylic acid (100 mmol, 22.4 g) as the starting material. The title compound was obtained as a white solid (22.1 g, 93% yield). This compound has been previously characterized.[49] ^{1}H NMR (400 MHz, DMSO-d_6) δ 7.57 (d, *J* = 15.9 Hz, 1H), 7.03 (s, 2H), 6.53 (d, *J* = 15.9 Hz, 1H), 3.80 (s, 6H), 3.70 (s, 3H).

Step 2: Synthesis of TIPS-Protected Hydroxycinnamate Esters.

General procedure 2.[52] Hydroxycinnamate ester (30 mmol, 1 equiv.) and imidazole (60 mmol, 2 equiv.) were dissolved in dry DCM (18 mL, 1.7 M). The solution was cooled to 0 °C, and TIPSCl (33 mmol, 1.1 equiv.) was added in small portions over the course of one hour. The reaction mixture was then heated to 40°C and monitored by TLC (EtOAc/PE 1:9). After completion, the mixture was colled to room temperature and concentrated under reduced pressure. Water (30 mL) was added, and the resulting mixture was extracted with DCM (3 x 15 mL). The combined organic layers were dried over $MgSO_4$ and then concentrated over silica gel. Purification was performed with flash chromatography using solid loading and EtOAc/PE as a mobile phase at a 20 mL/min flow rate. The desired products eluted at ~5-15% EtOAc.

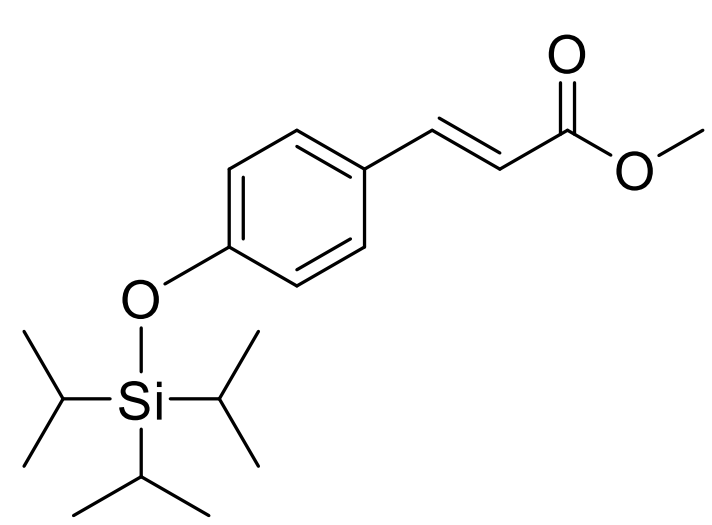

Methyl (*E*)-3-(4-((triisopropylsilyl)oxy)phenyl)acrylate was synthesized following the **general procedure 2** using methyl (*E*)-3-(4-hydroxyphenyl)acrylate (30 mmol, 5.3 g) as the starting material. The title compound was obtained as a colorless oil (9.1 g, 91% yield). This compound has been previously characterized.[52] ^{1}H NMR (400 MHz, $CDCl_3$) δ 7.63 (d, *J* = 16.0 Hz, 1H), 7.40 (d, *J* = 8.7 Hz, 2H), 6.86 (d, *J* = 8.6 Hz, 2H), 6.29 (d, *J* = 16.0 Hz, 1H), 3.77 (s, 3H), 1.30 – 1.21 (m, 3H), 1.09 (d, *J* = 7.4 Hz, 18H).

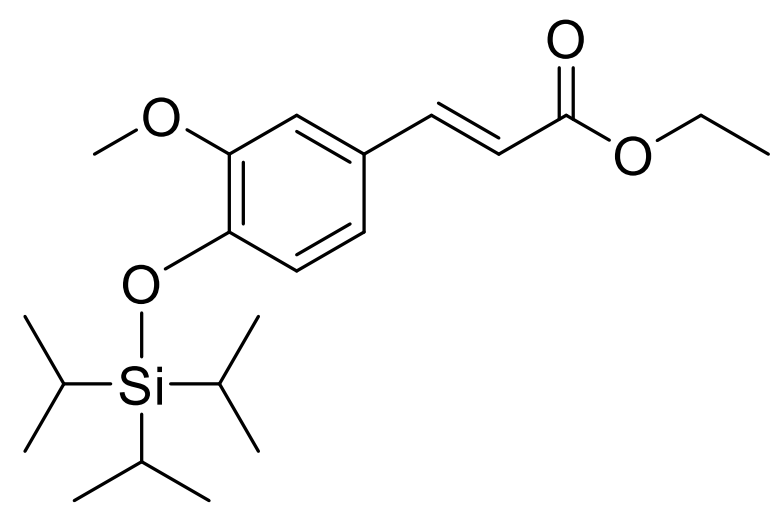

Ethyl (*E*)-3-(3-methoxy-4-((triisopropylsilyl)oxy)phenyl)acrylate was synthesized following the **general procedure 2** using ethyl (*E*)-3-(4-hydroxy-3-methoxyphenyl)acrylate (30 mmol, 6.7 g) as the starting material. The title compound was obtained as a white solid (9.6 g, 85% yield). This compound has been previously characterized.[53] ^{1}H NMR (400 MHz, $CDCl_3$) δ 7.61 (d, J = 15.9 Hz, 1H), 7.01 (s, 1H), 7.00 (d, J = 7.8 Hz, 1H), 6.85 (d, J = 7.9 Hz, 1H), 6.29 (d, J = 15.9 Hz, 1H), 4.25 (q, J = 7.1 Hz, 2H), 3.83 (s, 3H), 1.33 (t, J = 7.1 Hz, 3H), 1.30 – 1.20 (m, 3H), 1.09 (d, J = 7.4 Hz, 18H).

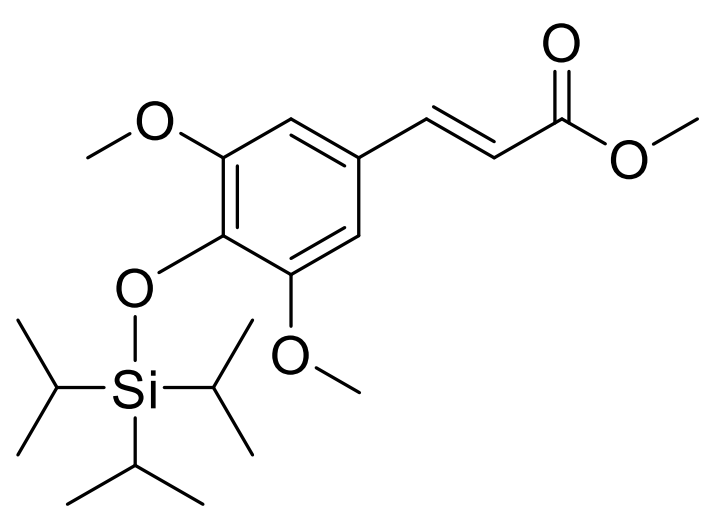

Methyl (*E*)-3-(3,5-dimethoxy-4-((triisopropylsilyl)oxy)phenyl)acrylate was synthesized following the **general procedure 2** using methyl (*E*)-3-(4-hydroxy-3,5-dimethoxyphenyl)acrylate (30 mmol, 7.1 g) as the starting material. The title compound was obtained as a colorless crystalline solid (11.3 g, 96% yield). This compound has been previously characterized.[54] ^{1}H NMR (400 MHz, $CDCl_3$) δ 7.60 (d, J = 15.9 Hz, 1H), 6.71 (s, 2H), 6.29 (d, J = 15.9 Hz, 1H), 3.81 (s, 6H), 3.79 (s, 3H), 1.33 – 1.16 (m, 3H), 1.07 (d, J = 7.4 Hz, 18H).

Step 3: Synthesis of Bromoketoesters **2a-c**.

General procedure 3. TIPS-protected hydroxycinnamate ester (16 mmol, 1 equiv.) was dissolved in a 2:1 mixture of acetonitrile and water (30 mL). The resulting suspension was treated with LiBr (2.10 g, 24 mmol, 1.5 equiv.) and 30% sulfuric acid (16 mmol, 2.8 mL), followed by the portionwise addition of $NaIO_4$ (856 mg, 4 mmol, 0.25 equiv.). Upon the addition of $NaIO_4$, the reaction mixture initially turned yellow, then deep orange. The reaction was stirred at room temperature and monitored by TLC (EtOAc/PE 3:7). After 24 hours, the organic solvent was

removed under reduced pressure, and the remaining water was evaporated under reduced pressure at 50 °C. The residue was dissolved in dry DCM (15 mL) and Dess-Martin periodinane (DMP, 7.50 g, 17.6 mmol, 1.1 equiv.) was added in four portions. The reaction was stirred at room temperature and monitored by TLC (EtOAc/PE 1:4). After 2 h, the mixture was concentrated under reduced pressure, and the residue was treated with diethyl ether (40 mL). This reaction mixture was washed successively with 1:1 mixture of 10% $Na_2S_2O_3$ and saturated $NaHCO_3$ (40 mL), brine (20 mL), and water (20 mL). The organic layer was dried over anhydrous $MgSO_4$ and concentrated onto silica gel. Purification was performed with flash chromatography using solid loading and EtOAc/PE as the mobile phase at a flow rate of 20 mL/min. The desired products eluted at ~15-20% EtOAc.

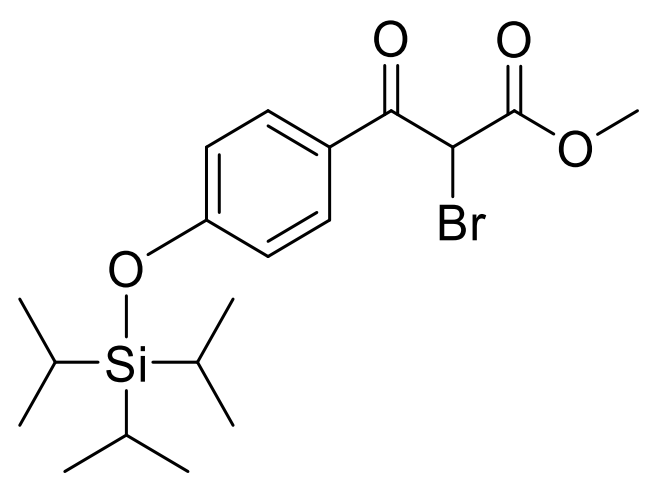

Methyl-2-bromo-3-oxo-3-(4-((triisopropylsilyl)oxy)phenyl) propanoate (2a) was synthesized following the **general procedure 3** using methyl (*E*)-3-(4-((triisopropylsilyl)oxy)phenyl)acrylate (5.30 g, 15.8 mmol) as the starting material. The title compound was obtained as a yellow oil (6.30 g, 14.7 mmol, 93% yield). ^{1}H NMR (400 MHz, $CDCl_3$) δ 7.91 (d, *J* = 8.7 Hz, 2H), 6.93 (d, *J* = 8.7 Hz, 2H), 5.64 (s, 1H), 3.82 (s, 3H), 1.29 (m, 3H), 1.10 (d, *J* = 7.5 Hz, 18H). ^{13}C NMR (101 MHz, $CDCl_3$) δ 186.5, 165.7, 161.7, 131.5, 126.2, 119.9, 53.7, 45.6, 17.6, 12.5. HRMS (ESI, m/z): calculated for $C_{19}H_{30}O_4BrSi$ $[M+H]^+$: 429.1091, found: 429.1086.

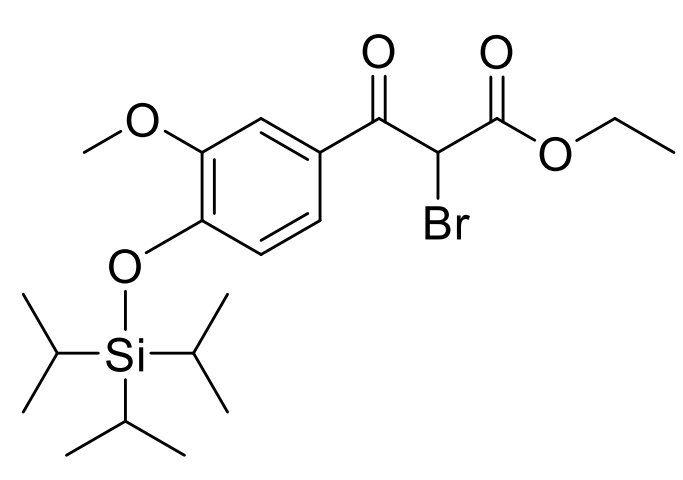

Ethyl-2-bromo-3-(3-methoxy-4-((triisopropylsilyl)oxy)phenyl)-3-oxopropanoate (2b) was synthesized following **the general procedure 3** using ethyl (*E*)-3-(3-methoxy-4-((triisopropylsilyl)oxy)phenyl)acrylate (6.00 g, 15.8 mmol) as the starting material. The title compound was obtained as a yellow gummy solid (6.70 g, 14.1 mmol, 90% yield): ^{1}H NMR (400

MHz, $CDCl_3$) δ 7.53 – 7.48 (m, 2H), 6.90 (d, *J* = 8.3 Hz, 1H), 5.63 (s, 1H), 4.27 (q, *J* = 7.1 Hz, 2H), 3.85 (s, 3H), 1.30 – 1.21 (m, 6H), 1.09 (d, *J* = 7.5 Hz, 18H). ^{13}C NMR (101 MHz, $CDCl_3$) δ 186.6, 165.3, 151.6, 151.1, 126.6, 123.5, 119.6, 112.0, 63.0, 55.3, 46.2, 17.6, 13.7, 12.8. HRMS (ESI, m/z): calculated for $C_{21}H_{34}O_5BrSi$ $[M+H]^+$: 473.1353, found: 473.1347.

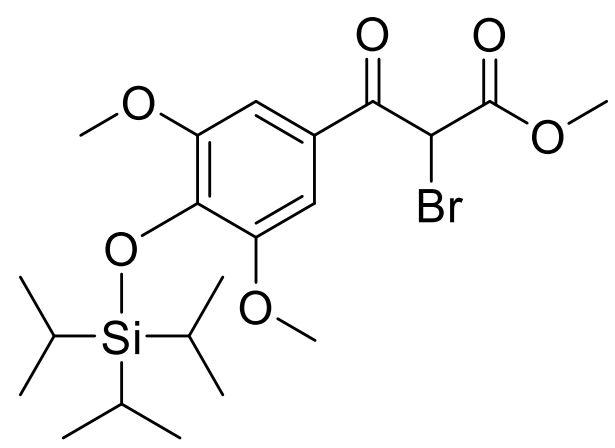

Methyl-2-bromo-3-(3,5-dimethoxy-4-((triisopropylsilyl)oxy)phenyl)-3-oxopropanoate (2c) was synthesized following **the general procedure 3** using methyl (*E*)-3-(3,5-dimethoxy-4-((triisopropylsilyl)oxy)phenyl)acrylate (6.30 g, 16.0 mmol) as the starting material. In the first step, an excess of LiBr (2.70 g, 32 mmol, 2.0 equiv.) and $NaIO_4$ (1.71 g, 8 mmol, 0.5 equiv.) was used to address the solubility issues of the starting material. The title compound was obtained as yellow crystals (6.50 g, 13.3 mmol, 83% yield): ^1H NMR (400 MHz, $CDCl_3$) δ 7.22 (s, 2H), 5.64 (s, 1H), 3.84 (s, 6H), 3.82 (s, 3H), 1.29 – 1.24 (m, 3H), 1.07 (d, *J* = 7.4 Hz, 18H). ^{13}C NMR (101 MHz, $CDCl_3$) δ 186.5, 165.8, 151.1, 141.4, 124.9, 106.4, 55.6, 53.7, 46.0, 17.7, 13.2. HRMS (ESI, m/z): calculated for $C_{21}H_{34}O_6BrSi$ $[M+H]^+$: 489.1303, found: 489.1297.

Optimization of β-*O*-4 Coupling

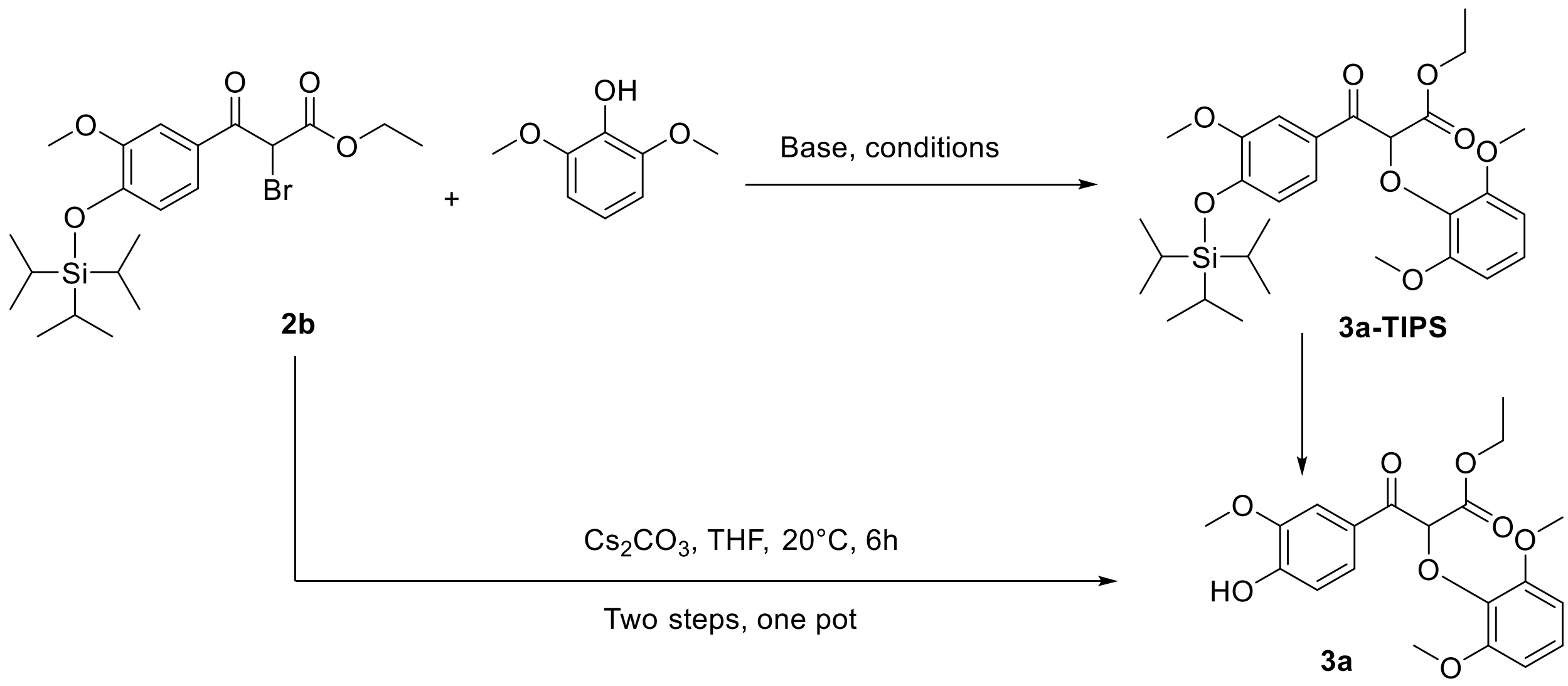

Table S1. Effect of different reaction conditions on the coupling between bromoketoester **2b** and syringol[a].

2b	Syringol	Solvent	Base	Conditions	Product, isolated yield
0.05 mmol (1 equiv.)	0.1 mmol (2 equiv.)	Acetone (0.5 mL)	K_2CO_3 (2 equiv.)	Reflux, N_2, 6.5 h	**3a-TIPS**, 83%
0.05 mmol (1 equiv.)	0.1 mmol (2 equiv.)	DMF (0.5 mL)	K_2CO_3 (2 equiv.)	Room temperature, N_2, then reflux, 6.5 h	**3a-TIPS,** 55%
0.05 mmol (1 equiv.)	0.1 mmol (2 equiv.)	THF (0.5 mL)	Cs_2CO_3 (2 equiv.)	Room temperature, N_2, 2 h	**3a-TIPS**, 80%; **3a**, 5%
0.05 mmol (1 equiv.)	0.075 mmol (1.5 equiv.)	THF (0.5 mL)	Cs_2CO_3 (3 equiv.)	Room temperature, N_2,2 h	**3a-TIPS**, 89%; **3a**, 8%
0.21 mmol (1 equiv.)	0.252 mmol (1.2 equiv.)	THF (2.1 mL)	Cs_2CO_3 (3 equiv.)	Room temperature, N_2, 5.5 h,	**3a**, 92%

[a] Reactions were performed according to general procedure 4 using 1.0 equiv. of **2b** and were monitored by TLC. The yield was reported after purifying the reactions using flash chromatography.

Synthesis of Oligomers with β-*O*-4 Linkages

Synthesis of dimers 3a-k

General procedure 4: Phenol (0.252 mmol, 1.2 equiv.) and Cs_2CO_3 (205.2 mg, 0.63 mmol, 3.0 equiv.) were added to a dried 10 mL flask and purged with nitrogen for 15 minutes. Dry THF (1.0 mL) was then added, and the reaction mixture was stirred at room temperature for 30 min, forming a creamy white suspension. A solution of brominated monomer **2** (0.21 mmol, 1.0 equiv.) in dry THF (1.0 mL) was then added in one portion, upon which the mixture turned yellowish-green. The reaction was stirred at room temperature until complete consumption of both brominated monomer **2** and TIPS-intermediate (3 – 10 h), as monitored by TLC (EtOAc/PE 3:7). The reaction mixture was treated with water (3 mL) and extracted with EtOAc (2 x 5 mL). The combined organic layers were dried over anhydrous $MgSO_4$, filtered, and concentrated under reduced pressure. The crude residue was adsorbed onto silica gel and purified by flash chromatography (solid loading, EtOAc/PE gradient, 10 mL/min flow rate). The desired products eluted at ~25-35% EtOAc.

General procedure 5: Phenol (0.252 mmol, 1.2 equiv.) and Cs_2CO_3 (0.63 mmol, 3.0 equiv., 204.8 mg) were added to a dried 10 mL flask and purged with nitrogen for 15 minutes. Dry THF (1.0 mL) was then added. The reaction mixture was stirred at room temperature for 30 min, forming a creamy white suspension. A solution of brominated monomer **2c** (102.7 mg, 0.21 mmol, 1.0 equiv.) in dry THF (1.0 mL) was then added, resulting in a yellow-colored reaction mixture. The reaction was stirred at room temperature until complete consumption of **2c**, as monitored by TLC (EtOAc/PE 1:9), typically within 2 hours. Subsequently, TBAF·xH_2O (55 mg, ~15 wt% H_2O, 0.18 mmol) was dissolved in THF (100 μL) and added under nitrogen. The reaction was stirred at room temperature and monitored by TLC (EtOAc/PE 3:7) until complete deprotection after around 30 min. The reaction was then quenched with saturated aqueous NH_4Cl (3 mL) and extracted with EtOAc (2 x 5 mL). The combined organic layers were dried over anhydrous $MgSO_4$, filtered, and concentrated onto silica gel. Purification was performed by flash chromatography (solid loading, EtOAc/PE gradient, 10 mL/min flow rate). The desired products eluted at ~25-35% EtOAc.

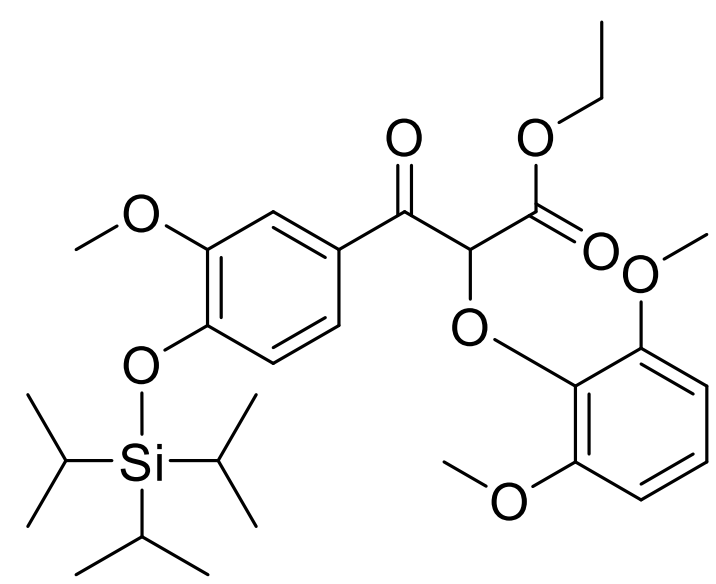

3a-TIPS was synthesized following the **general procedure 4**, (see Table S1). The title compound was obtained as a white foam (22.7 mg, 0.041 mmol, 83% yield). ^{1}H NMR (400 MHz, $CDCl_3$) δ 7.71 (dd, *J* = 8.3, 2.1 Hz, 1H), 7.67 (d, *J* = 2.1 Hz, 1H), 6.96 (t, *J* = 8.4 Hz, 1H), 6.87 (d, *J* = 8.3 Hz, 1H), 6.51 (d, *J* = 8.4 Hz, 2H), 5.55 (s, 1H), 4.28 – 4.20 (m, 2H), 3.83 (s, 3H), 3.67 (s, 6H), 1.30 – 1.22 (m, 3H), 1.18 (t, *J* = 7.1 Hz, 3H), 1.08 (d, *J* = 7.4 Hz, 18H). ^{13}C NMR (101 MHz, $CDCl_3$) δ 190.6, 167.6, 152.9, 150.9, 150.8, 136.4, 128.4, 124.5, 124.3, 119.7, 112.7, 105.5, 84.9, 61.8, 56.1, 55.5, 17.9, 14.1, 13.0. HRMS (ESI, m/z): calculated for $C_{29}H_{43}O_8Si[M+H]^+$: 547.2722, found: 547.2722.

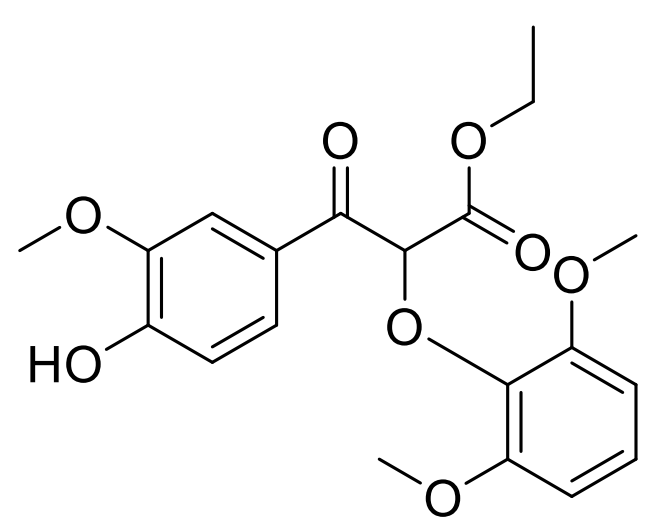

3a was synthesized following **the general procedure 4** using **2b** (99.3 mg, 0.210 mmol) and syringol (38.8 mg, 0.252 mmol) as starting materials. The title compound was obtained as a colorless crystalline solid (75.0 mg, 0.192 mmol, 92% yield). ^{1}H NMR (400 MHz, $CDCl_3$) δ 7.78 (dd, *J* = 8.4, 2.0 Hz, 1H), 7.70 (d, *J* = 2.0 Hz, 1H), 7.00 – 6.88 (m, 2H), 6.50 (d, *J* = 8.4 Hz, 2H), 6.27 (br s, 1H), 5.56 (s, 1H), 4.23 (q, *J* = 7.1 Hz, 2H), 3.90 (s, 3H), 3.67 (s, 6H), 1.20 (t, *J* = 7.1 Hz, 3H). ^{13}C NMR (101 MHz, $CDCl_3$) δ 190.5, 167.6, 152.9, 150.9, 146.4, 136.3, 127.5, 125.4, 124.5, 113.9, 111.6, 105.5, 85.0, 61.8, 56.1, 56.1, 14.1. HRMS (ESI, m/z): calculated for $C_{20}H_{23}O_8[M+H]^+$: 391.1379, found: 391.1387.

Procedure for gram-scale synthesis of 3a: Syringol (813.9 mg, 5.30 mmol, 1.2 equiv.) and Cs_2CO_3 (4.3 g, 13.2 mmol, 3.0 equiv.) were added to a dried 100 mL flask and purged with nitrogen for 30 minutes. Dry THF (30.0 mL) was then added, and the reaction mixture was stirred at room temperature for 60 min, forming a creamy white suspension. A solution of brominated monomer **2b** (2.1 g, 4.4 mmol, 1.0 equiv.) in dry THF (15.0 mL) was then added in three portions, upon which the mixture turned yellowish-dark green. The reaction was stirred at room temperature until complete consumption of both brominated monomer **2b** and TIPS-intermediate (~ 12 h), as monitored by TLC (EtOAc/PE 3:7). The reaction mixture was treated with water (20 mL) and extracted with EtOAc (3 x 10 mL). The combined organic layers were dried over anhydrous $MgSO_4$, filtered, and concentrated under reduced pressure. The crude residue was adsorbed onto silica gel and purified by flash chromatography (solid loading, EtOAc/PE gradient, 20 mL/min flow rate). The desired product eluted at ~30-45% EtOAc giving 1.4 g, 83% yield).

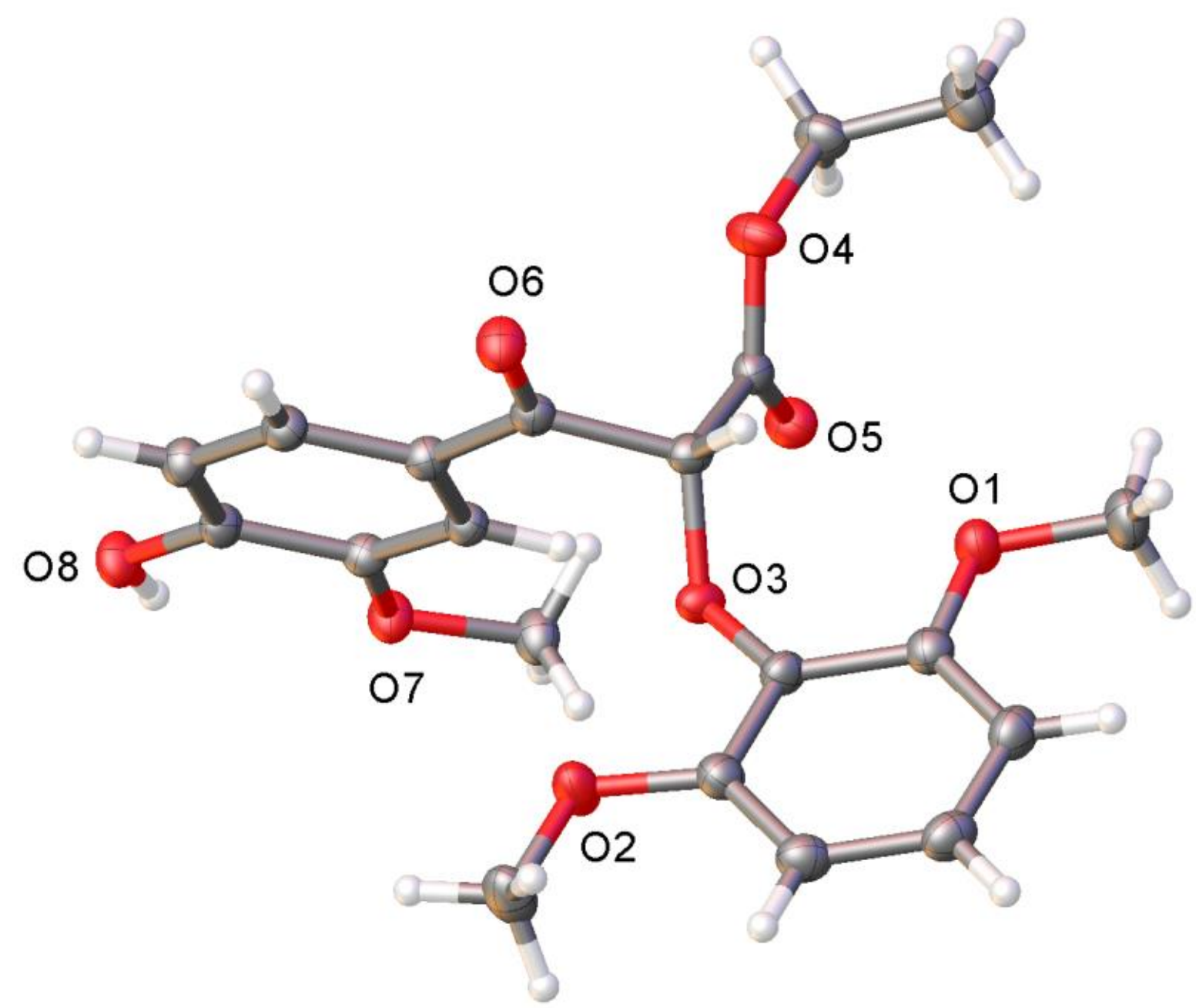

Crystal structure of **3a**. Thermal ellipsoids are set to a 50% probability level. The unit cell contains two molecules of **3a** and a molecule of ethyl acetate, only one molecule of **3a** is shown for clarity.

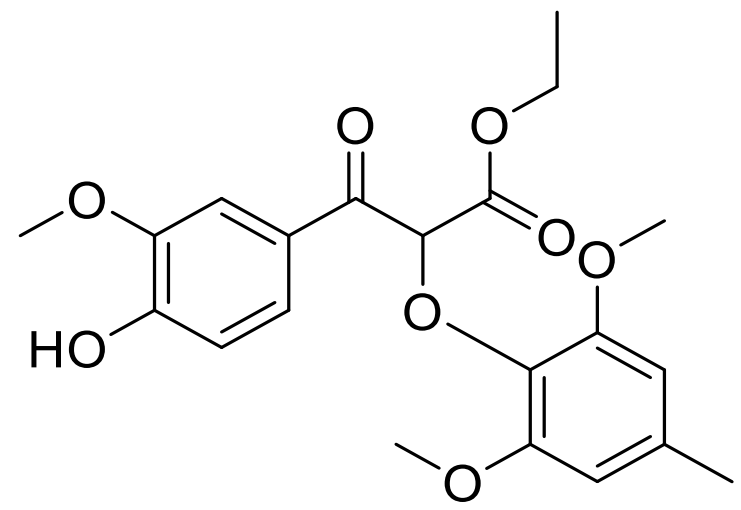

3b was synthesized following **the general procedure 4** using **2b** (99.3 mg, 0.210 mmol) and methylsyringol (42.4 mg, 0.252 mmol) as starting materials. The title compound was obtained as yellow gum (64.0 mg, 0.158 mmol, 75% yield). ^{1}H NMR (400 MHz, $CDCl_3$) δ 7.79 (dd, *J* = 8.4, 2.0 Hz, 1H), 7.70 (d, *J* = 2.0 Hz, 1H), 6.92 (d, *J* = 8.4 Hz, 1H), 6.31 (s, 2H), 6.22 (br s, 1H), 5.50 (s, 1H), 4.23 (q, *J* = 7.1 Hz, 2H), 3.91 (s, 3H), 3.65 (s, 6H), 2.26 (s, 3H), 1.20 (t, *J* = 7.1 Hz, 3H). ^{13}C NMR (101 MHz, $CDCl_3$) δ 190.5, 167.7, 152.3, 150.9, 146.4, 134.5, 134.0, 127.5, 125.3, 113.9, 111.6, 106.2, 85.1, 61.8, 56.1, 55.9, 21.9, 14.1. HRMS (ESI, m/z): calculated for $C_{21}H_{24}O_8$[M+Na]$^+$: 427.1363, found: 427.1363.

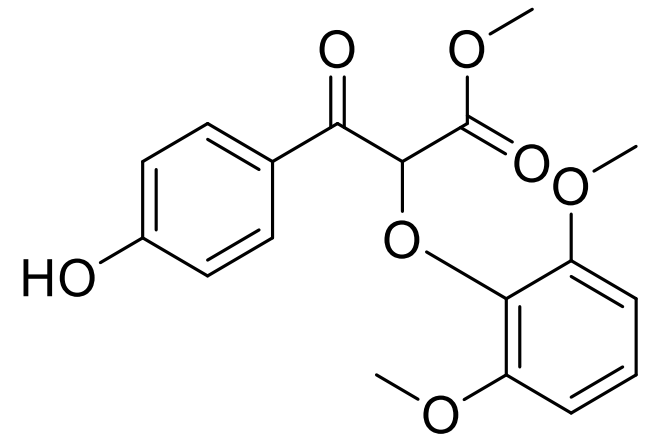

3c was synthesized following **the general procedure 4** using **2a** (90.1 mg, 0.210 mmol) and syringol (38.8 mg, 0.252 mmol) as starting materials. The title compound was obtained as a colorless oil (65.0 mg, 0.188 mmol, 90% yield). ^{1}H NMR (400 MHz, $CDCl_3$) δ 8.00 (d, *J* = 8.8 Hz, 2H), 6.96 (t, *J* = 8.4 Hz, 1H), 6.86 (d, *J* = 8.8 Hz, 2H), 6.50 (d, *J* = 8.4 Hz, 2H), 5.62 (s, 1H), 3.76 (s, 3H), 3.66 (s, 6H). ^{13}C NMR (101 MHz, $CDCl_3$) δ 190.8, 168.3, 161.8, 152.7, 136.0, 132.3, 126.8, 124.7, 115.5, 105.4, 84.6, 55.9, 52.9. HRMS (ESI, m/z): calculated for $C_{18}H_{18}O_7$[M+Na]$^+$: 369.0945, found: 369.0947.

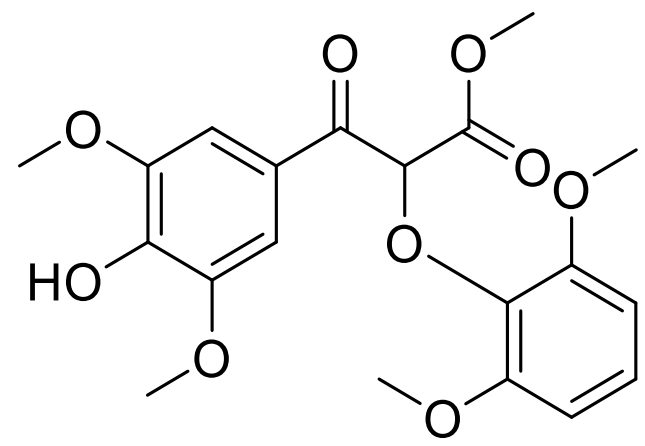

3d was synthesized following **the general procedure 5** using **2c** (102.7 mg, 0.210 mmol) and syringol (38.8 mg, 0.252 mmol) as starting materials. The title compound was obtained as a yellow solid (73.5 mg, 0.181 mmol, 86% yield). ^{1}H NMR (400 MHz, $CDCl_3$) δ 7.52 (s, 2H), 6.99 (t, *J* = 8.4 Hz, 1H), 6.53 (d, *J* = 8.4 Hz, 2H), 5.99 (s, 1H), 5.58 (s, 1H), 3.92 (s, 6H), 3.79 (s, 3H), 3.69 (s, 6H). ^{13}C NMR (101 MHz, $CDCl_3$) δ 190.1, 168.1, 152.8, 146.6, 140.1, 136.0, 125.9, 124.6, 107.1, 105.4, 85.0, 56.4, 56.0, 52.7. HRMS (ESI, m/z): calculated for $C_{20}H_{23}O_9[M+H]^+$: 407.1330, found: 407.1337.

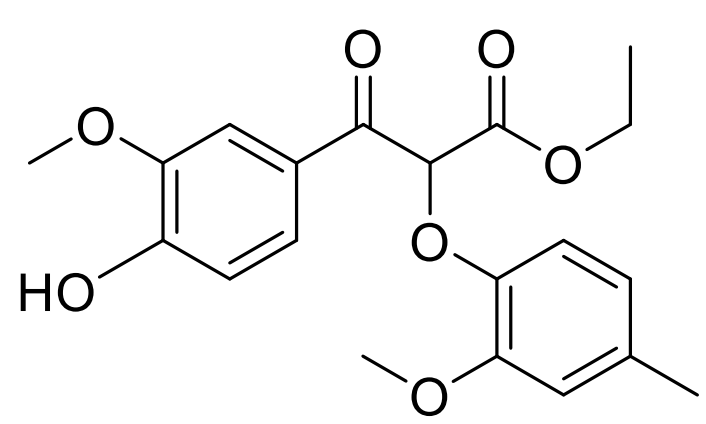

3e was synthesized following **the general procedure 4** using **2b** (99.3 mg, 0.210 mmol) and creosol (35.0 mg, 0.252 mmol) as starting materials. The title compound was obtained as a yellow oil (66.7 mg, 0.178 mmol, 85% yield). ^{1}H NMR (400 MHz, $CDCl_3$) δ 7.82 (dd, *J* = 8.4, 2.0 Hz, 1H), 7.70 (d, *J* = 2.0 Hz, 1H), 6.94 (d, *J* = 8.4 Hz, 1H), 6.86 (d, *J* = 8.1 Hz, 1H), 6.70 (s, 1H), 6.62 (d, *J* = 8.6 Hz, 1H), 5.71 (s, 1H), 4.25 (q, *J* = 7.1 Hz, 2H), 3.93 (s, 3H), 3.79 (s, 3H), 2.27 (s, 3H), 1.22 (t, *J* = 7.1 Hz, 3H). ^{13}C NMR (101 MHz, $CDCl_3$) δ 190.4, 167.4, 151.3, 150.3, 146.6, 144.2, 134.1, 127.2, 125.6, 121.2, 118.9, 114.2, 113.7, 111.6, 83.0, 62.2, 56.2, 55.8, 21.3, 14.2. HRMS (ESI, m/z): calculated for $C_{20}H_{23}O_7[M+H]^+$: 375.1431, found: 375.1438.

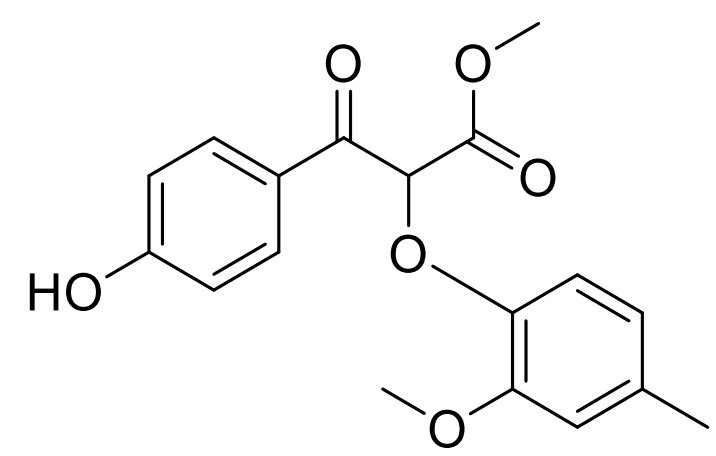

3f was synthesized following **the general procedure 4** using **2a** (90.1 mg, 0.210 mmol) and creosol (35.0 mg, 0.252 mmol) as starting materials. The title compound was obtained as a colorless oil (61.7 mg, 0.187 mmol, 89% yield). ^{1}H NMR (400 MHz, $CDCl_3$) δ 8.06 (d, *J* = 8.8 Hz, 2H), 6.88 – 6.83 (m, 3H), 6.71 (dd, *J* = 7.0, 2.0 Hz, 1H), 6.66 – 6.60 (m, 1H), 5.74 (s, 1H), 3.84 (s, 1H), 3.79 (s, 3H), 3.77 (s, 3H), 2.28 (s, 3H). ^{13}C NMR (101 MHz, $CDCl_3$) δ 190.5, 168.1, 161.5, 150.2, 144.1, 134.3, 132.6, 131.0, 127.1, 121.3, 118.9, 115.7, 113.8, 83.0, 55.9, 53.1, 21.3. HRMS (ESI, m/z): calculated for $C_{18}H_{19}O_6[M+H]^+$: 331.1170, found: 331.1176.

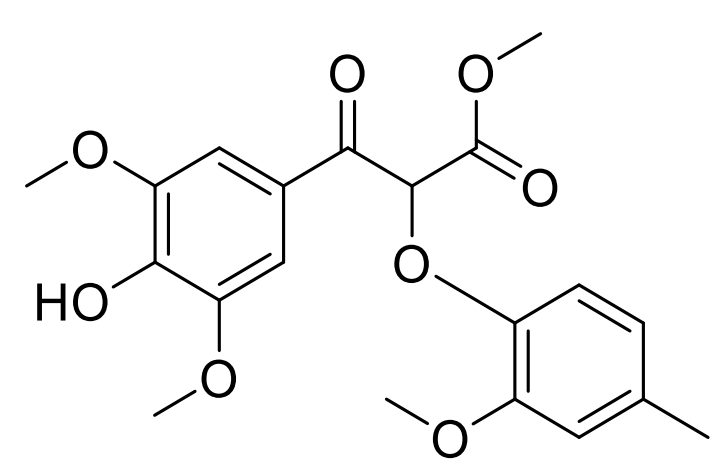

3g was synthesized following **the general procedure 5** using **2c** (102.7 mg, 0.210 mmol) and creosol (35.0 mg, 0.252 mmol) as starting materials. The title compound was obtained as a yellow solid (58.1 mg, 0.149 mmol, 71% yield). ^{1}H NMR (400 MHz, $CDCl_3$) δ 7.53 (s, 2H), 6.84 (d, *J* = 8.1 Hz, 1H), 6.70 (d, *J* = 2.0 Hz, 1H), 6.61 (dd, *J* = 8.1, 2.0 Hz, 1H), 6.10 (br s, 1H), 5.70 (s, 1H), 3.91 (s, 6H), 3.79 (s, 3H), 3.78 (s, 3H), 2.27 (s, 3H).^{13}C NMR (101 MHz, $CDCl_3$) δ 190.0, 167.8, 150.1, 146.7, 143.8, 140.6, 134.0, 125.4, 121.1, 118.4, 113.5, 107.3, 83.3, 56.4, 55.7, 52.9, 21.2. HRMS (ESI, m/z): calculated for $C_{20}H_{22}O_8[M+Na]^+$: 413.1207, found: 413.1207.

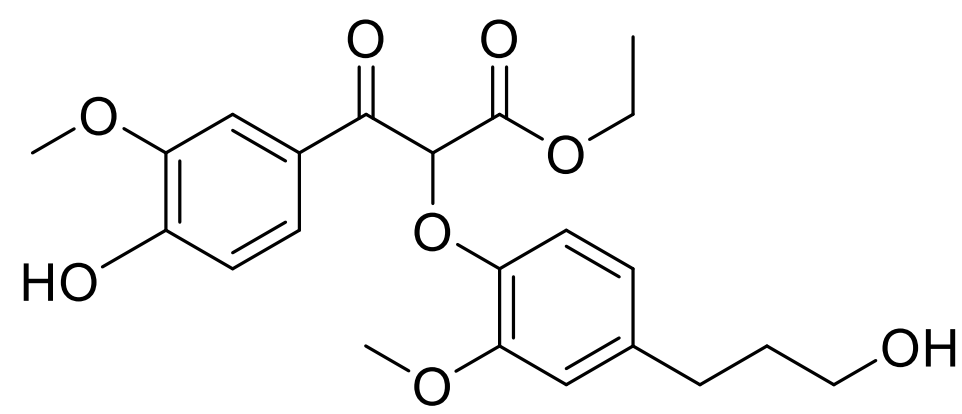

3h was synthesized following **the general procedure 4** using **2b** (99.3 mg, 0.210 mmol) and dihydroconiferol (45.9 mg, 0.252 mmol) as starting materials. The title compound was obtained as a yellow oil (64.0 mg, 0.153 mmol, 73% yield). ^{1}H NMR (400 MHz, acetone-d$_6$) δ 7.76 (dd, *J* = 8.4, 2.0 Hz, 1H), 7.70 (d, *J* = 2.0 Hz, 1H), 6.95 – 6.90 (m, 3H), 6.70 (dd, *J* = 8.1, 2.0 Hz, 1H), 5.91 (s, 1H), 4.21 (q, *J* = 7.1 Hz, 2H), 3.89 (s, 3H), 3.80 (s, 3H), 3.63 (br s, 1H), 3.55 (t, *J* = 6.4 Hz, 2H), 2.66 – 2.59 (m, 2H), 1.82 – 1.75 (m, 2H), 1.19 (t, *J* = 7.1 Hz, 3H). ^{13}C NMR (101 MHz, acetone-d$_6$) δ 190.5, 168.0, 153.3, 151.2, 148.4, 145.4, 139.1, 127.7, 125.7, 121.2, 118.8, 115.6, 114.2, 113.1, 82.4, 62.1, 61.7, 56.3, 56.2, 35.5, 32.4, 14.4. HRMS (ESI, m/z): calculated for $C_{22}H_{27}O_8$[M+H]$^+$: 419.1695, found: 419.1700.

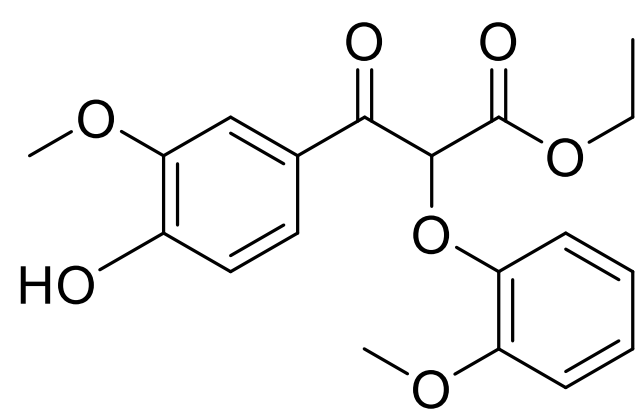

3i was synthesized following **the general procedure 4** using **2b** (99.3 mg, 0.210 mmol) and guaiacol (31.2 mg, 0.252 mmol) as starting materials. The title compound was obtained as a colorless crystalline solid (62.0 mg, 0.172 mmol, 82% yield). ^{1}H NMR (400 MHz, $CDCl_3$) δ 7.83 (dd, *J* = 8.4, 2.0 Hz, 1H), 7.71 (d, *J* = 1.9 Hz, 1H), 7.04 – 6.94 (m, 3H), 6.90 (dd, *J* = 8.1, 1.6 Hz, 1H), 6.87 – 6.81 (m, 1H), 6.13 (s, 1H), 5.75 (s, 1H), 4.26 (q, *J* = 7.1 Hz, 2H), 3.95 (s, 3H), 3.81 (s, 3H), 1.23 (t, *J* = 7.1 Hz, 3H). ^{13}C NMR (101 MHz, $CDCl_3$) δ 189.8, 167.0, 151.0, 150.3, 146.3, 146.1, 126.9, 125.4, 123.9, 120.7, 118.5, 113.9, 112.5, 111.3, 82.6, 61.9, 55.9, 55.6, 13.9. HRMS (ESI, m/z): calculated for $C_{19}H_{21}O_7$[M+H]$^+$: 361.1276, found: 361.1282.

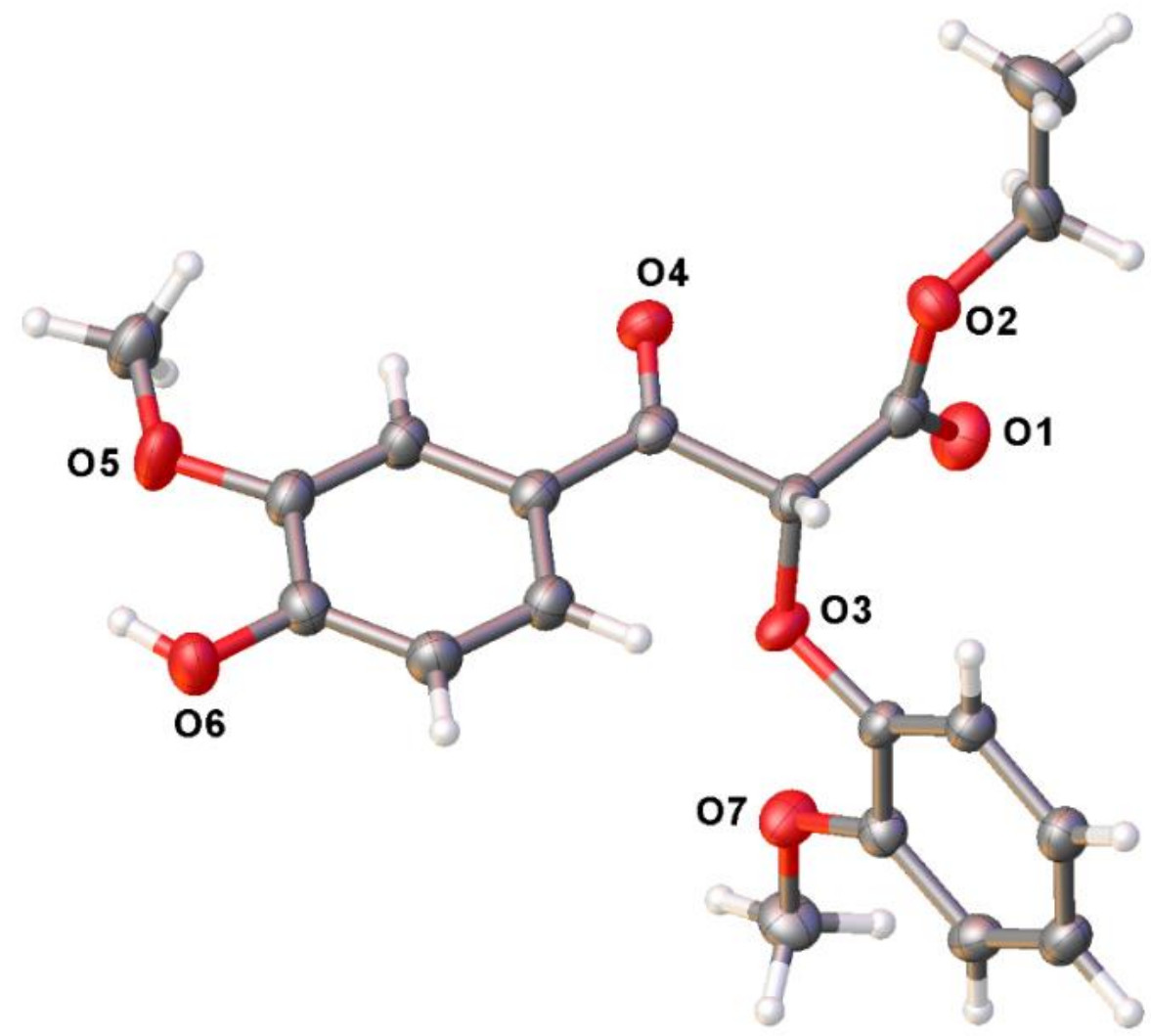

Crystal structure of **3i**. Thermal ellipsoids are set to a 50% probability level.

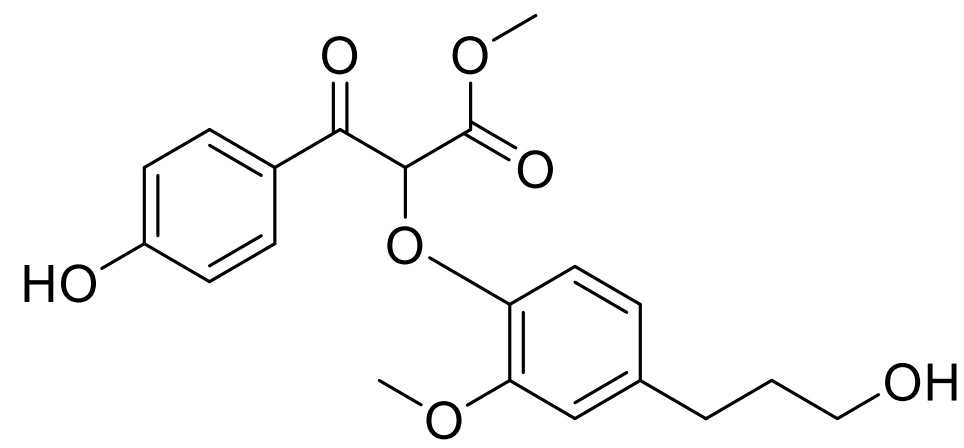

3j was synthesized following **the general procedure 4** using **2a** (90.1 mg, 0.210 mmol) and dihydroconiferol (45.9 mg, 0.252 mmol) as starting materials. The title compound was obtained as a colorless oil (61.5 mg, 0.164 mmol, 78% yield). ^{1}H NMR (400 MHz, acetone-d$_6$) δ 8.06 (d, *J* = 8.8 Hz, 2H), 6.95 (d, *J* = 8.8 Hz, 2H), 6.92 – 6.90 (m, 2H), 6.70 (dd, *J* = 8.1, 2.1 Hz, 1H), 5.92 (s, 1H), 3.79 (s, 3H), 3.74 (s, 3H), 3.55 (t, *J* = 6.4 Hz, 3H), 2.65 – 2.61 (m, 2H), 1.81 – 1.77 (m, 2H). ^{13}C NMR (101 MHz, acetone-d$_6$) δ 190.5, 168.5, 163.7, 151.2, 145.4, 139.2, 133.1, 127.6, 121.2, 118.9, 116.2, 114.2, 82.4, 61.7, 56.2, 52.7, 35.5, 32.5. HRMS (ESI, m/z): calculated for $C_{20}H_{23}O_7[M+H]^+$: 375.1431, found: 375.1438.

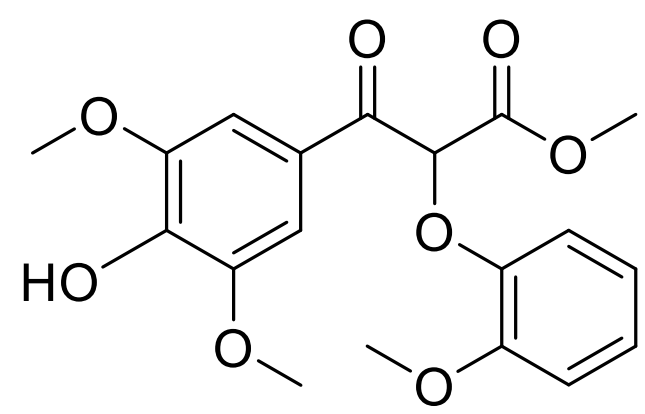

3k was synthesized following **the general procedure 5** using **2c** (102.7 mg, 0.210 mmol) and guaiacol (31.2 mg, 0.252 mmol) as starting materials. The title compound was obtained as a brown oil (54.4 mg, 0.145 mmol, 69% yield). ^{1}H NMR (400 MHz, $CDCl_3$) δ 7.54 (s, 2H), 7.03 – 6.99 (m, 1H), 6.97 – 6.94 (m, 1H), 6.91 – 6.89 (m, 1H), 6.86 – 6.81 (m, 1H), 6.06 (br s, 1H), 5.74 (s, 1H), 3.93 (s, 6H), 3.82 (s, 3H), 3.80 (s, 3H). ^{13}C NMR (101 MHz, $CDCl_3$) δ 190.0, 167.8, 150.5, 146.9, 146.2, 140.8, 125.5, 124.2, 121.1, 118.4, 112.7, 107.4, 83.3, 56.5, 55.9, 53.1. HRMS (ESI, m/z): calculated for $C_{19}H_{20}O_8[M+Na]^+$: 399.1050, found: 399.1051.

Synthesis of trimers 4a-i

General procedure 6: Unprotected dimer **3** (0.10 mmol, 1.0 equiv.) and Cs_2CO_3 (0.30 mmol, 3.0 equiv.) were added to a dried 10 mL flask and purged with nitrogen for 15 min. A solvent mixture of THF (0.4 mL) and DMF (0.1 mL) was added, and the reaction was stirred at room temperature for 30 min, forming a creamy white suspension. A solution of brominated monomer **2** (0.15 mmol; 1.5 equiv.) in dry THF (0.5 mL) was then added. The reaction mixture was stirred at room temperature until complete consumption of monomer **2**, as monitored by TLC (EtOAc/PE = 1:1). The reaction mixture was then treated with water (2 mL) and extracted with EtOAc (2 × 3 mL). The combined organic layers were dried over anhydrous $MgSO_4$, filtered, and concentrated under reduced pressure. The crude residue was adsorbed onto silica and purified by flash chromatography using solid loading, EtOAc/PE as the mobile phase at 5 mL/min flow rate. The desired trimers eluted at ~30-45% EtOAc.

General procedure 7: Unprotected dimer **3a** or **3g** (0.10 mmol, 1.0 equiv.) and Cs_2CO_3 (0.30 mmol, 3.0 equiv.) were added to a dried 10 mL flask and purged with nitrogen for 15 min. A solvent mixture of THF (0.4 mL) and DMF (0.1 mL) was added, and the reaction was stirred at room temperature for 30 min, forming a creamy yellow suspension. A solution of brominated monomer **2c** (0.15 mmol; 1.5 equiv.) in dry THF (0.5 mL) was added. The reaction mixture was stirred at room temperature until complete consumption of monomer **2c**, as monitored by TLC (EtOAc/PE = 2:3). The reaction proceeded for ~2.5 h to afford protected trimer. Subsequently, TBAF·xH_2O (26.1 mg, 0.085 mmol) was added under nitrogen in 50 µL of dry THF. The mixture was stirred at room temperature and monitored by TLC (EtOAc/PE = 2:3). Upon completion, the reaction was quenched with saturated aqueous NH_4Cl (2 mL) and extracted with EtOAc (2 × 3 mL). The combined organic layers were dried over anhydrous $MgSO_4$, filtered, and concentrated onto silica gel. The crude product was purified with flash chromatography using solid loading and EtOAc/PE as a mobile phase at 5 mL/min flow rate, with the desired products eluting at ~30-45% EtOAc.

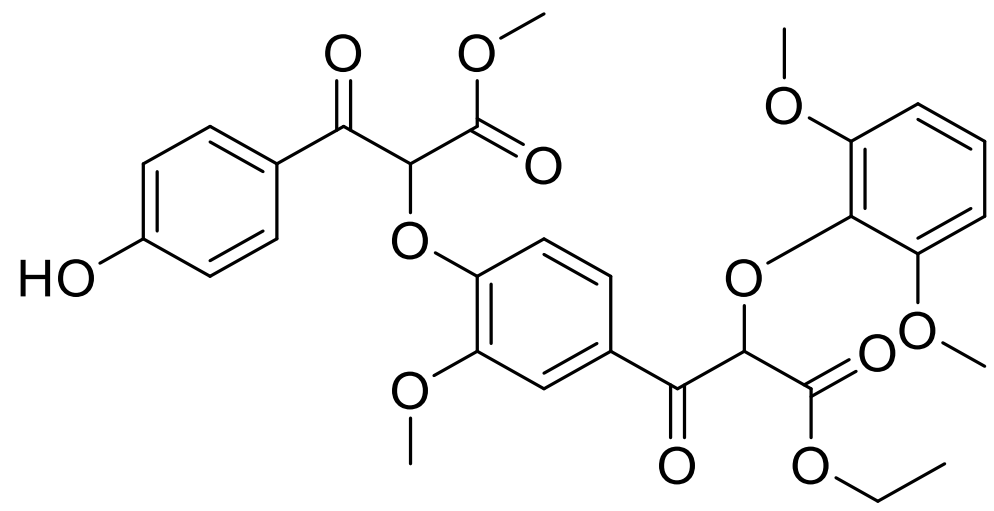

4a was synthesized following **the general procedure 6** using **3a** (39.0 mg, 0.100 mmol) and **2a** (64.2 mg, 0.15 mmol) as starting materials. The title compound was obtained as a white foam (44.2 mg, 0.076 mmol, 1:1 mixture of two diastereomers, 76% yield). ^{1}H NMR (400 MHz, acetone-d_6) δ 9.44 (br s, 1H), 8.06 (d, *J* = 8.9 Hz, 2H), 7.75 – 7.69 (m, 2H), 7.09 – 7.07 (m, 1H), 7.03 – 6.95 (m, 3H), 6.63 (d, *J* = 8.4 Hz, 2H), 6.26 (s, 1H), 5.68 (s, 1H, overlapping two singlets from two diastereoisomers), 4.20 (m, 2H), 3.82 (s, 3H), 3.77 (s, 3H), 3.66 (s, 6H), 1.18 (m, 3H, overlapping two triplets). ^{13}C NMR (101 MHz, acetone-d_6) δ 191.2, 189.5, 167.9, 167.7, 163.8, 153.6, 151.6, 150.4, 136.8, 136.7, 133.0, 130.6 (60), 130.6 (56), 127.1, 125.2, 124.4 (44), 124.4 (39), 116.4, 115.6, 115.5, 114.0 (98), 114.0 (96) 106.3, 84.7 (73), 84.7 (66), 80.7, 62.0, 56.3, 53.0, 14.4. HRMS (ESI, m/z): calculated for $C_{30}H_{31}O_{12}$[M+H]$^+$: 583.1810, found: 583.1799.

Procedure for 1g-scale synthesis of 4a: Dimer **3a** (1.17 g, 3.0 mmol, 1.0 equiv.) and Cs_2CO_3 (2.90 g, 9.0 mmol, 3.0 equiv.) were added to a dried 100 mL flask and purged with nitrogen for 30 min. A solvent mixture of THF (12 mL) and DMF (3 mL) was added, and the reaction was stirred at room temperature for 60 min, forming a creamy white suspension. A solution of brominated monomer **2a** (1.90 g, 4.5 mmol; 1.5 equiv.) in dry THF (15 mL) was then added. The reaction mixture was stirred at room temperature until complete consumption of monomer **2a** and TIPS-intermediate, as monitored by TLC (EtOAc/PE = 1:1). The reaction mixture was then treated with water (10 mL) and extracted with EtOAc (3 × 7 mL). The combined organic layers were dried over anhydrous $MgSO_4$, filtered, and concentrated under reduced pressure. The crude residue was adsorbed onto silica and purified by flash chromatography using solid loading, EtOAc/PE as the mobile phase at 10 mL/min flow rate. The desired trimer **4a** eluted at ~ 45-55% EtOAc (1.15 g, 66% yield).

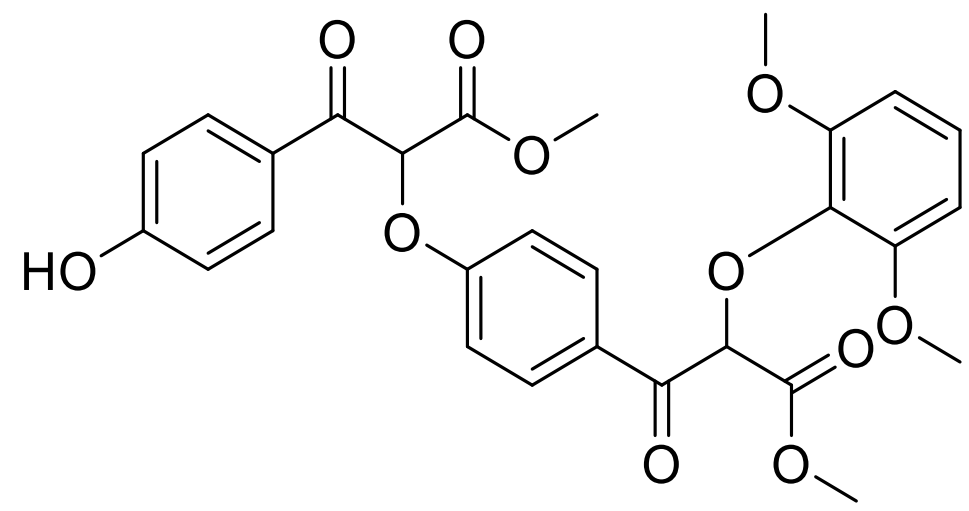

4b was synthesized following **the general procedure 6** using **3c** (34.6 mg, 0.100 mmol) and **2a** (64.2 mg, 0.15 mmol) as starting materials. The title compound was obtained as a pale yellow oil (41.4 mg, 0.077 mmol, 1:1 mixture of two diastereomers, 77% yield). ^{1}H NMR (400 MHz, $CDCl_3$) δ 8.11 (d, *J* = 9.0 Hz, 2H), 7.99 (d, *J* = 8.8 Hz, 2H), 6.98 – 6.94 (m, 3H), 6.89 (d, *J* = 8.8 Hz, 2H), 6.48 (d, *J* = 8.4 Hz, 2H), 5.82 (s, 1H, overlapping two singlets from two diastereoisomers), 5.53 (s, 1H, overlapping two singlets from two diastereoisomers), 3.82 (s, 3H), 3.77 (s, 3H), 3.63 (s, 3H), 3.62 (s, 3H). ^{13}C NMR (101 MHz, $CDCl_3$) δ 190.7, 188.8 (82), 188.8 (78), 167.9, 166.7, 162.0, 160.7 (70), 160.7 (68), 152.7, 135.9, 132.4, 132.2, 129.0, 126.2 (23), 126.2 (21), 124.7 (72), 124.7 (70), 115.9, 114.8, 105.3 (28), 105.3 (26), 85.1 (15), 85.1 (09), 80.4, 80.3, 55.9 (89), 55.9 (86), 53.4, 52.9. HRMS (ESI, m/z): calculated for $C_{28}H_{26}O_{11}[M+Na]^+$: 561.1367, found: 561.1370.

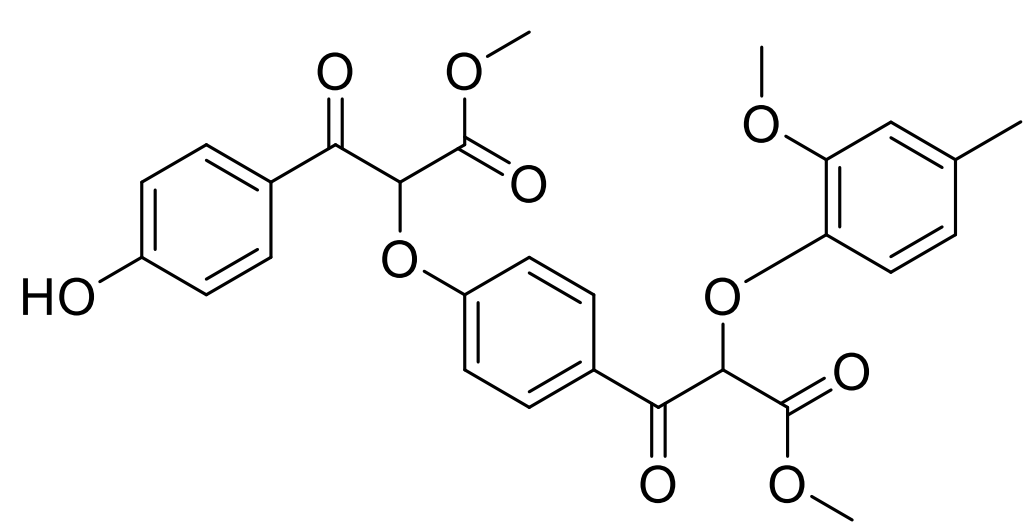

4c was synthesized following **the general procedure 6** using **3f** (33.0 mg, 0.100 mmol) and **2a** (64.2 mg, 0.15 mmol) as starting materials. The title compound was obtained as a yellow oil (38.0 mg, 0.073 mmol, 1:1 mixture of two diastereomers, 73% yield). ^{1}H NMR (400 MHz, acetone-d_6) δ 8.14 (d, *J* = 8.0 Hz, 2H), 8.02 (d, *J* = 8.8 Hz, 2H), 7.13 (d, *J* = 9.0 Hz, 2H), 6.97 (d, *J* = 8.8 Hz, 2H), 6.89 (d, *J* = 8.1 Hz, 1H), 6.84 (d, *J* = 2.0 Hz, 1H), 6.67 – 6.62 (m, 1H), 6.34 (s, 1H), 5.91 (s, 1H), 3.78 (s, 3H), 3.75 (s, 3H), 3.73 (s, 3H), 2.25 (s, 3H). ^{13}C NMR (101 MHz, acetone-d_6) δ 190.0, 188.4, 167.4, 166.6, 163.2, 161.3, 150.3, 144.3, 133.6, 132.1, 131.9, 130.5, 128.8, 126.2, 120.9, 118.3 (30), 118.3 (28), 115.6, 115.0, 113.8, 81.8, 78.8, 55.2, 52.3, 51.9, 20.2. HRMS (ESI, m/z): calculated for $C_{28}H_{26}O_{10}[M+Na]^+$: 545.1418, found: 545.1420.

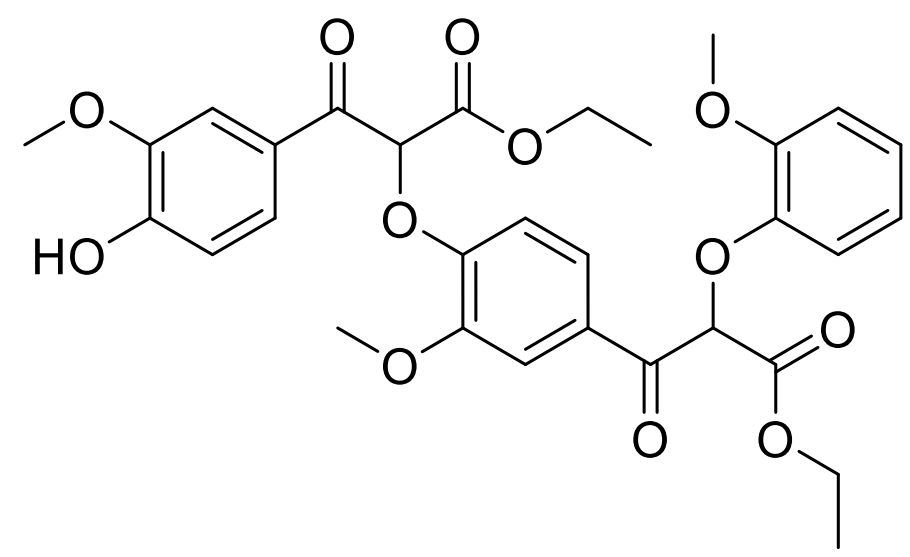

4d was synthesized following **the general procedure 6** using **3i** (36.0 mg, 0.100 mmol) and **2b** (70.8 mg, 0.15 mmol) as starting materials. The title compound was obtained as a yellow oil (33.0 mg, 0.055 mmol, 1:1 mixture of two diastereomers, 55% yield). In addition, 7.6 mg of unreacted **3i** were isolated (70% yield of **4d** after recovering the unreacted starting material). ^{1}H NMR (400 MHz, $CDCl_3$) δ 7.80 – 7.74 (m, 2H), 7.72 – 7.67 (m, 2H), 7.02 – 6.98 (m, 1H), 6.96 – 6.91 (m, 3H), 6.89 – 6.85 (m, 1H), 6.84 – 6.80 (m, 1H), 6.30 (br s, 1H), 5.86 (s, 1H), 5.71 (s, 1H), 4.29 – 4.21 (m, 4H), 3.92 (s, 3H), 3.86 (s, 3H), 3.78 (s, 3H), 1.25 – 1.18 (m, 6H). ^{13}C NMR (101 MHz, $CDCl_3$) δ 190.3, 189.0, 167.1, 166.4, 151.7, 150.9, 150.5, 149.9, 146.8, 146.3, 129.5, 126.6, 125.6, 124.3, 120.9, 118.9, 115.6, 114.3, 113.2, 112.7, 111.5, 82.9, 81.8 (79), 81.8 (77), 62.6, 62.3, 56.2, 56.1, 55.9, 14.1. HRMS (ESI, m/z): calculated for $C_{31}H_{32}O_{12}$[M+Na]$^{+}$: 619.1786, found: 619.1786.

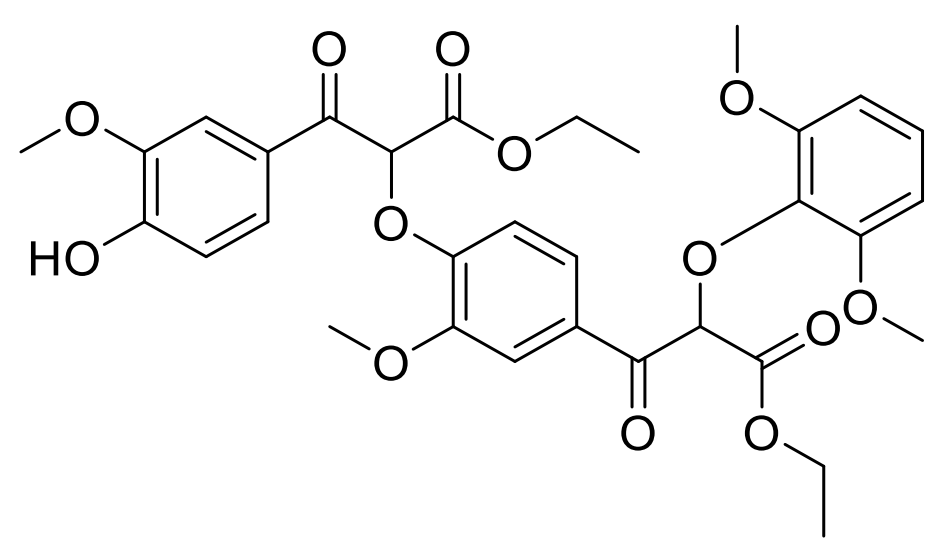

4e was synthesized following **the general procedure 6** using **3a** (39.0 mg, 0.100 mmol) and **2b** (70.8 mg, 0.15 mmol) as starting materials. The title compound was obtained as a colorless oil (41.0 mg, 0.065 mmol, 1:1 mixture of two diastereomers, 65% yield). ^{1}H NMR (400 MHz, $CDCl_3$) δ 7.81 (dd, J = 8.5, 1.9 Hz, 1H), 7.75 – 7.71 (m, 1H), 7.73 – 7.69 (m, 2H), 6.96 (dd, J = 8.4, 1.7 Hz, 2H), 6.91 (d, J = 8.4 Hz, 1H), 6.50 (d, J = 8.3 Hz, 2H), 6.16 (br s, 1H), 5.85 (s, 1H), 5.51 (s, 1H, overlapping two singlets from two diastereoisomers), 4.32 – 4.22 (m, 4H), 3.95 (s, 3H), 3.87 (s, 3H), 3.65 (s, 6H), 1.24 (t, J = 7.2 Hz, 3H), 1.20 (t, J = 7.1 Hz, 3H). ^{13}C NMR (101 MHz, $CDCl_3$) δ 190.7, 189.2, 167.5, 166.5, 152.9, 151.6, 150.6, 149.9, 146.7, 136.2, 130.1, 126.8, 125.6, 124.7, 124.2 (18), 124.2 (16), 115.6, 114.3, 113.2, 111.6, 85.3, 85.2, 82.0, 62.6, 62.0, 56.2, 56.1, 56.0, 14.2. HRMS (ESI, m/z): calculated for $C_{32}H_{34}O_{13}$[M+Na]$^{+}$: 649.1892, found: 649.1897.

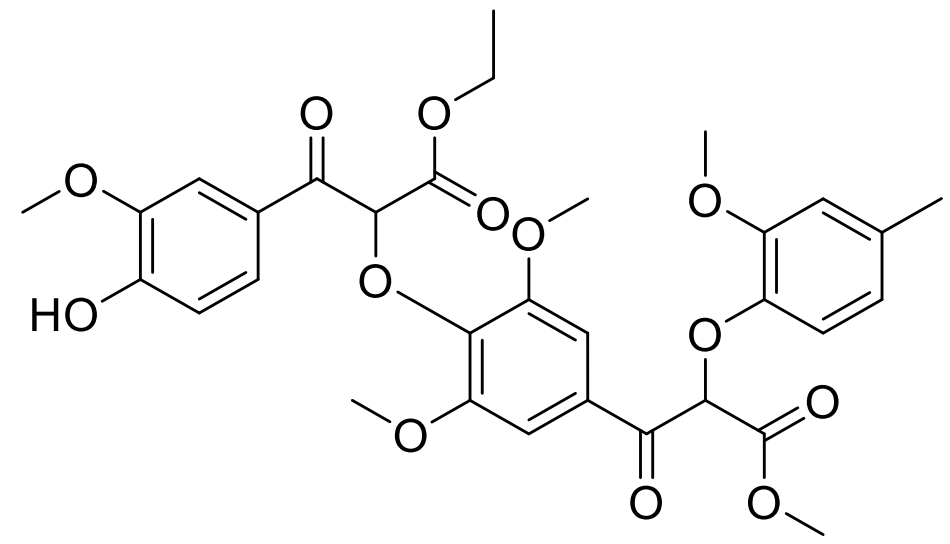

4f was synthesized following **the general procedure 6** using **3g** (39.0 mg, 0.100 mmol) and **2b** (70.8 mg, 0.15 mmol) as starting materials. The title compound was obtained as a dark yellow oil (40.1 mg, 0.064 mmol, 1:1 mixture of two diastereomers, 64% yield). ^{1}H NMR (400 MHz, $CDCl_3$) δ 7.78 – 7.75 (m, 1H), 7.67 (d, *J* = 2.0 Hz, 1H), 7.44 (s, 2H), 6.93 (d, *J* = 8.4 Hz, 1H), 6.82 (d, *J* = 8.1 Hz, 1H), 6.69 (d, *J* = 1.9 Hz, 1H), 6.62 –6.59 (m, 1H), 6.23 (br s, 1H), 5.82 (s, 1H), 5.67 (s, 1H), 4.24 (q, *J* = 7.1 Hz, 2H), 3.92 (s, 3H), 3.78 (s, 3H), 3.76 (s, 3H), 3.74 (s, 6H), 2.26 (s, 3H), 1.20 (t, *J* = 7.1 Hz, 3H). ^{13}C NMR (101 MHz, $CDCl_3$) δ 190.5, 189.6, 167.7, 167.1, 152.2, 151.1, 150.2, 146.6, 143.8, 140.9, 134.3, 129.6, 127.2, 125.5, 121.2, 118.8 (77), 118.7 (74), 114.0, 113.6, 111.5, 107.5, 84.1 (11), 84.1 (09), 83.4, 62.1, 56.2, 56.2, 55.7, 53.0, 21.2, 14.1. HRMS (ESI, m/z): calculated for $C_{32}H_{34}O_{13}[M+Na]^+$: 649.1892, found: 649.1896.

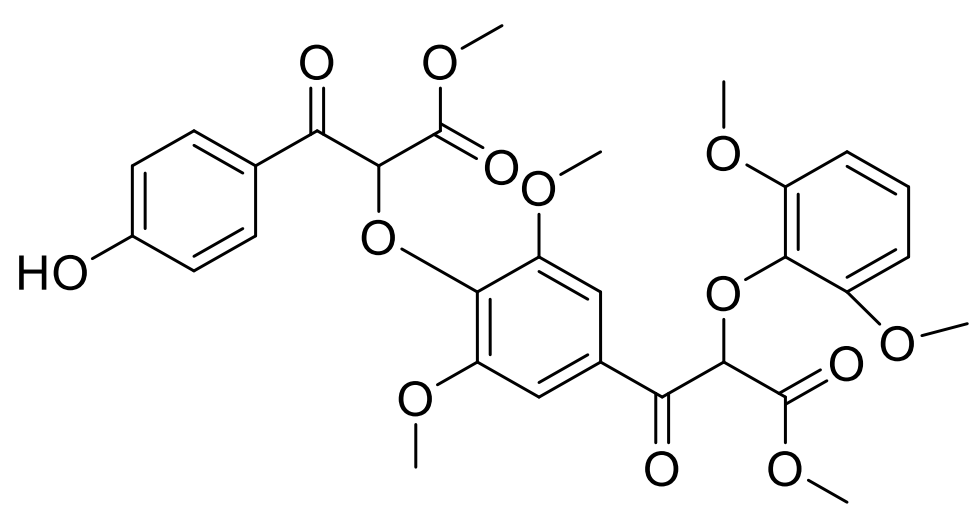

4g was synthesized following **the general procedure 6** using **3d** (40.6 mg, 0.100 mmol) and **2a** (64.2 mg, 0.15 mmol) as starting materials. The title compound was obtained as a colorless oil (34.0 mg, 0.057 mmol, 1:1 mixture of two diastereomers, 57% yield). In addition, 10 mg of unreacted **3d** were isolated (75% yield of **4g** after recovering the unreacted starting material). ^{1}H NMR (400 MHz, $CDCl_3$) δ 8.08 (d, *J* = 7.2 Hz, 2H), 7.41 (s, 2H), 6.99 (t, *J* = 8.4 Hz, 1H), 6.87 (d, *J* = 8.8 Hz, 2H), 6.51 (d, *J* = 8.4 Hz, 2H), 5.79 (s, overlapping two singlets from two diastereoisomers, 1H), 5.71 (br s, 1H), 5.53 (s, overlapping two singlets from two diastereoisomers, 1H), 3.79 (s, 6H), 3.72 (s, 6H), 3.66 (s, 6H). ^{13}C NMR (101 MHz, $CDCl_3$) δ 190.5, 189.6, 167.9, 167.5, 160.6, 152.8, 151.9, 140.3, 135.8, 132.4, 130.2 (17), 130.2 (16), 127.5, 124.7, 115.3, 107.2, 105.4, 85.0, 84.3 (32), 84.3 (28), 56.1, 56.0, 52.9, 52.8. HRMS (ESI, m/z): calculated for $C_{30}H_{30}O_{13}[M+Na]^+$: 621.1579, found: 621.1584.

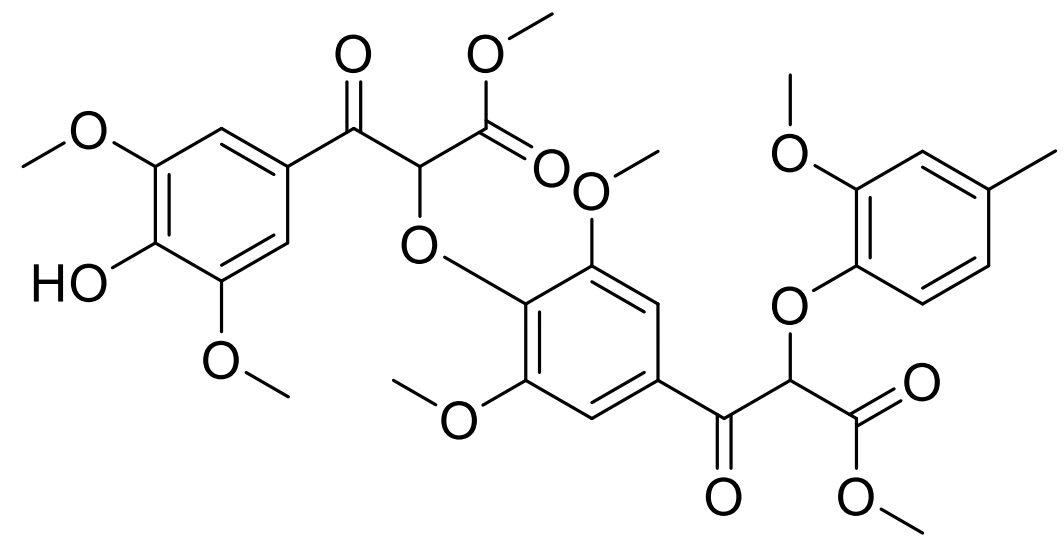

4h was synthesized following **the general procedure 7** using **3g** (39.0 mg, 0.100 mmol) and **2c** (73.2 mg, 0.15 mmol) as starting materials. The title compound was obtained as a brown oil (46.2 mg, 0.072 mmol, 1:1 mixture of two diastereomers, 72% yield). ^{1}H NMR (400 MHz, $CDCl_3$) δ 7.50 – 7.45 (m, 4H), 6.83 (d, J = 8.1 Hz, 1H), 6.69 (d, J = 2.0 Hz, 1H), 6.63 – 6.57 (m, 1H), 5.82 (s, 1H), 5.66 (s, 1H), 3.91 (s, 6H), 3.79 (s, 6H), 3.77 (s, 3H), 3.75 (s, 6H), 2.27 (s, 3H). ^{13}C NMR (101 MHz, $CDCl_3$) δ 190.4, 189.3, 167.6 (59), 167.6 (56), 167.6 (55), 152.1, 150.1, 146.7, 143.7, 140.5, 140.4, 134.3, 129.7, 121.1, 118.7 (71), 118.7 (67), 113.5, 107.4, 107.2, 84.3 (30), 84.3 (28), 83.4, 56.4, 56.2, 55.7, 52.9 (94), 52.9 (89), 21.2, 21.0. HRMS (ESI, m/z): calculated for $C_{32}H_{35}O_{14}$[M+H]$^+$: 643.2021, found: 643.2027.

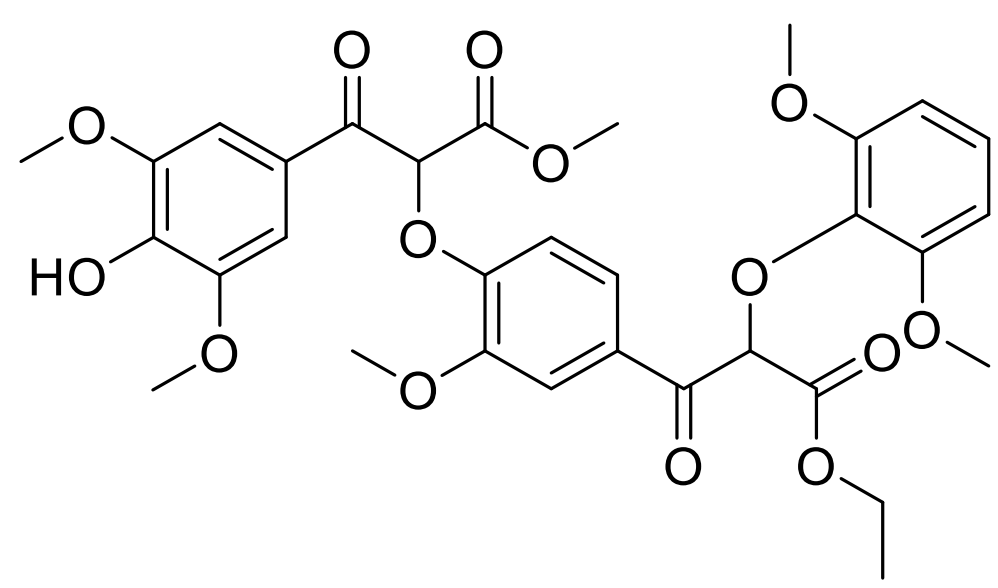

4i was synthesized following **the general procedure 7** using **3a** (39.0 mg, 0.100 mmol) and **2c** (73.2 mg, 0.15 mmol) as starting materials. The title compound was obtained as a brown oil (48.2 mg, 0.075 mmol, 1:1 mixture of two diastereomers, 75% yield). ^{1}H NMR (400 MHz, $CDCl_3$) δ 7.75 – 7.68 (m, 2H), 7.50 (s, 2H), 6.94 (t, J = 8.4 Hz, 1H), 6.88 (d, J = 8.4 Hz, 1H), 6.47 (d, J = 8.4 Hz, 2H), 5.82 (s, 1H), 5.48 (s, overlapping two singlets from two diastereoisomers, 1H), 4.21 (q, J = 7.1 Hz, 2H), 3.90 (s, 6H), 3.84 (s, overlapping two singlets, 3H), 3.79 (s, 3H), 3.62 (s, 6H), 1.18 – 1.14 (m, overlapping two triplets, 3H). ^{13}C NMR (101 MHz, $CDCl_3$) δ 190.6 (57), 190.6 (55), 188.92, 167.4, 166.9, 152.8, 150.2, 149.6, 146.9, 141.1, 136.1, 130.0, 124.9, 124.6, 124.1, 115.0 (03), 115.0 (99), 113.0 (04), 113.0 (02), 107.3, 105.4, 105.3, 85.1 (14), 85.1 (06), 82.3, 61.9, 56.4, 56.0, 55.9, 53.3, 14.1. HRMS (ESI, m/z): calculated for $C_{32}H_{34}O_{14}Na$[M+Na]$^+$: 665.1841, found: 665.1830.

Synthesis of tetramer 5

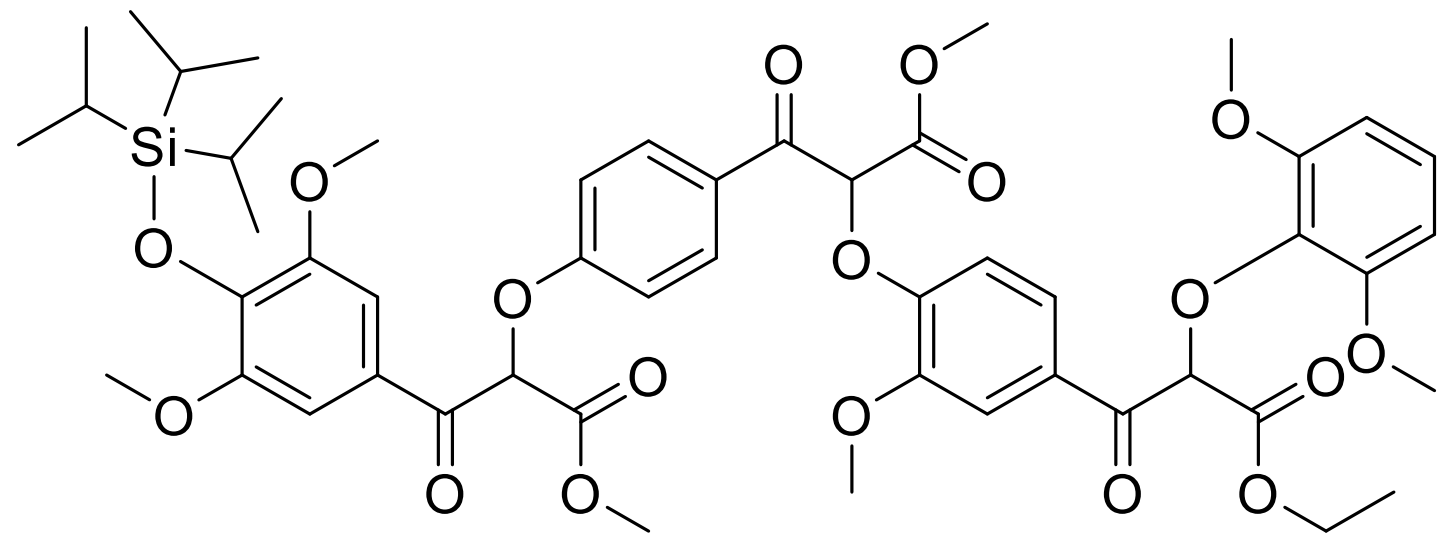

5-TIPS: unprotected trimer **4a** (291 mg, 0.50 mmol, 1.0 equiv.) and Cs_2CO_3 (488.7 mg, 1.50 mmol, 3.0 equiv.) were added to a 50 mL flask, which was subsequently purged with nitrogen for 15 minutes. A solvent mixture of THF (3.5 mL) and DMF (0.5 mL) was then added. The reaction mixture was stirred at room temperature for 30 min before a solution of brominated monomer **2c** (366.7 mg, 0.75 mmol, 1.5 equiv.) in dry THF (1.0 mL) was added dropwise. The reaction mixture was stirred at room temperature for 2 h and reaction progress was monitored by TLC (EtOAc/PE 4:6) until complete consumption of the monomer and the formation of the TIPS-protected tetramer was observed. Upon completion, the reaction mixture was treated with water (5 mL) and extracted with EtOAc (2 × 5 mL). The combined organic layers were dried over anhydrous $MgSO_4$, filtered, and concentrated under reduced pressure. The crude residue was adsorbed onto silica gel and purified by flash chromatography (solid loading, EtOAc/PE gradient, 10 mL/min flow rate). The desired product **5-TIPS** eluted at ~40% EtOAc. The title compound was obtained as a dark yellow oil (356.4 mg, 0.36 mmol, expected mixture of four diastereomers, NMR resolves two diastereomers, 72% yield). ^{1}H NMR (400 MHz, $CDCl_3$) δ 8.12 (d, *J* = 9.0 Hz, 2H), 7.77 – 7.69 (m, 2H), 7.34 (s, 2H), 7.03 – 6.99 (m, 2H), 6.95 (d, *J* = 8.6 Hz, 1H), 6.90 (d, *J* = 8.4 Hz, 1H), 6.50 (d, *J* = 1.2 Hz, 1H), 6.48 (d, *J* = 1.1 Hz, 1H), 5.84 (s, 1H), 5.81 (s, 1H), 5.50 (s, overlapping two singlets from diastereoisomers, 1H), 4.23 (q, *J* = 7.1 Hz, 2H), 3.83 (s, 3H), 3.82 (s, 3H), 3.81 (s, 6H), 3.79 (s, 3H), 3.64 (s, 6H), 1.25 (t, *J* = 7.2 Hz, 3H), 1.21– 1.17 (m, 3H), 1.06 (d, *J* = 7.4 Hz, 18H).^{13}C NMR (101 MHz, $CDCl_3$) δ 190.6 (63), 190.6 (61), 189.1, 188.6, 167.4, 166.8, 166.7, 161.3, 152.9, 151.3, 150.4 (37), 150.4 (35), 149.9, 141.9, 136.2 (17), 136.2 (15), 132.5, 130.3 (31), 130.3 (29), 128.4, 125.4 (36), 125.4 (35), 124.7, 124.6, 124.1 (08), 124.1 (06), 116.0 (02), 116.0 (00), 115.2, 113.2 (22), 113.2 (20), 106.9, 105.4, 85.2, 85.1, 81.9, 81.0 (01), 81.0 (99), 61.9, 56.1, 56.0, 55.8, 53.4, 53.3, 18.0, 14.1, 13.5. HRMS (ESI, m/z): calculated for $C_{51}H_{61}O_{18}Si[M-H]^+$: 989.3622, found: 989.3631.

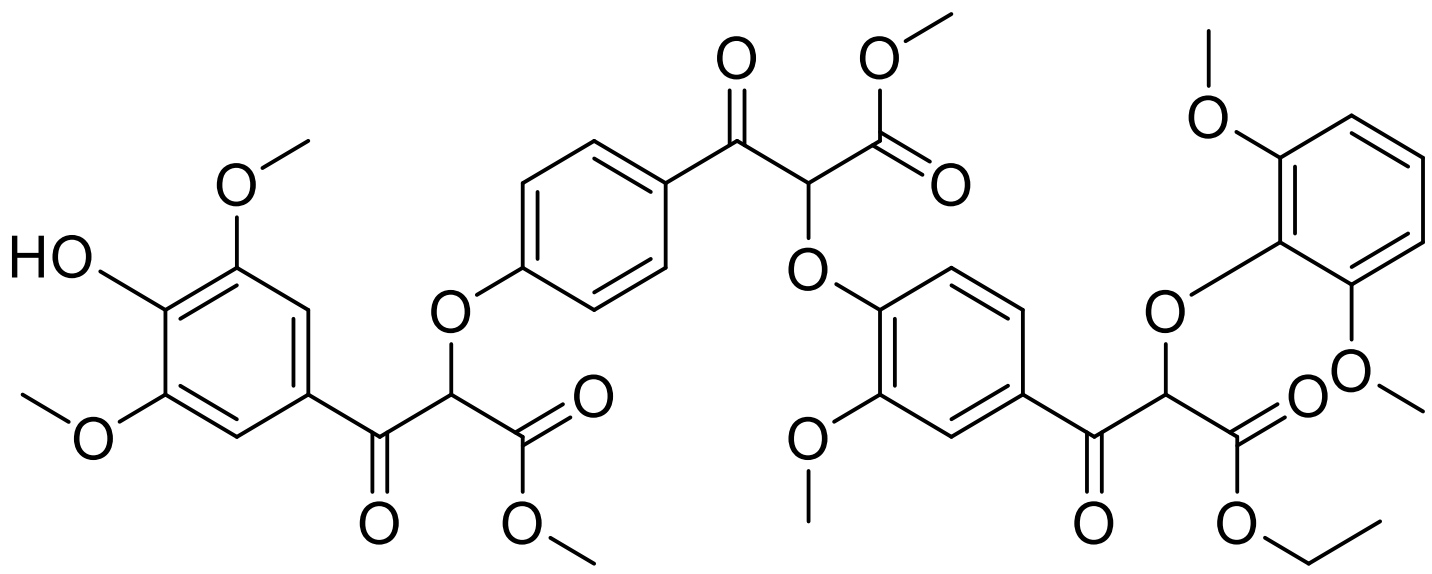

5: TIPS-protected tetramer **5-TIPS** (198 mg, 0.200 mmol, 1.0 equiv.) was placed in a reaction flask, flushed with nitrogen for 10 min, and dissolved in dry THF (1.5 mL). A solution of TBAF·xH_2O (52.5 mg, ~15 wt% H_2O, 0.17 mmol) in THF (500 µL) was added dropwise. The reaction mixture was stirred under nitrogen with a stopper at room temperature for 30 min. The reaction progress was monitored by TLC (EtOAc/PE 3:2). Upon completion, the reaction mixture was quenched with saturated aqueous NH_4Cl (2 mL) and extracted with EtOAc (2 ×2 mL). The combined organic layers were dried over anhydrous $MgSO_4$, filtered, and concentrated onto silica gel. Purification was performed by flash chromatography (solid loading, EtOAc/PE gradient, 5 mL/min flow rate), with the desired product **5** eluting at ~65-70% EtOAc. The title compound was obtained as a brown oil (142 mg, 0.170 mmol, 85% yield, expected mixture of four diastereomers, NMR resolves two diastereomers). Obtained tetramer 5 was used in the next step to prepare **LMC8** without further purification. ^{1}H NMR (400 MHz, $CDCl_3$) δ 8.12 (d, *J* = 8.9 Hz, 2H), 7.75 – 7.68 (m, 2H), 7.42 (s, 2H), 7.03 – 6.93 (m, 3H), 6.89 (d, *J* = 8.6 Hz, 1H), 6.49 (d, *J* = 8.5 Hz, 2H), 6.16 (br s, 1H), 5.84 (s, 1H), 5.79 (s, 1H), 5.49 (s, overlapping four singlets from four diastereomers, 1H), 4.23 (q, J = 7.1 Hz, 2H), 3.91 (s, 6H), 3.82 (s, 6H), 3.79 (s, 3H), 3.63 (s, 6H), 1.18 (t, *J* = 7.1Hz, 3H). ^{13}C NMR (101 MHz, $CDCl_3$) δ 190.7, 190.6, 189.1 (12), 189.1 (10), 188.5, 167.4, 166.8, 166.6 (61), 166.6 (59), 161.2, 152.8, 150.3 (34), 150.3 (32), 149.8, 147.0, 141.4, 136.1, 132.5, 130.3 (30), 130.3 (27), 128.5 (49), 128.5 (48), 124.7 (74), 124.7 (66), 124.7 (65), 124.1 (09), 124.1 (07), 116.0 (00), 116.0 (97), 115.2, 113.2 (21), 113.2 (19), 107.2, 107.1, 105.4, 85.2 (20), 85.2 (15), 82.0, 81.2, 62.0, 56.6, 56.1, 56.0 (01), 56.0 (00), 53.5, 53.3, 14.1. HRMS (ESI, m/z): calculated for $C_{42}H_{41}O_{18}[M-H]^+$: 833.2287, found: 833.2295.

Synthesis of Oligomers with β-5 Linkage

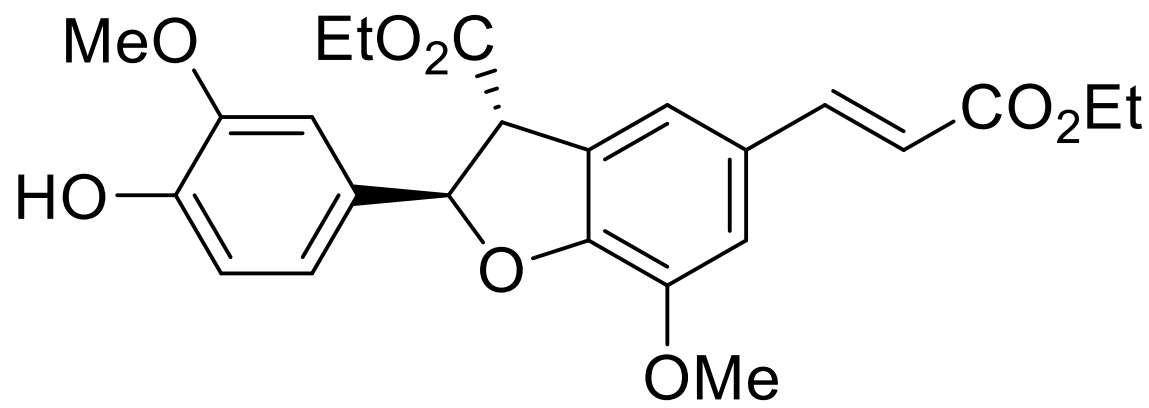

6:[35] Silver (I) oxide (83 mg, 0.36 mmol, 1.5 equiv.) and ethyl (*E*)-3-(4-hydroxy-3-methoxyphenyl)acrylate (107 mg, 0.48 mmol, 2.0 equiv.) were added to an oven-dried 25 mL round-bottom flask and purged with nitrogen for 15 min. Dry DCM (4.8 mL) was added, and the reaction mixture was stirred at room temperature. Reaction was monitored by TLC. After 18 h, prior to full consumption of starting material to minimize byproduct formation, the reaction mixture was filtered through a pad of celite, which was washed with DCM. The filtrate was subsequently washed with brine (4 mL) and water (4 mL), and the organic phase was dried over anhydrous $MgSO_4$. The solvent was evaporated in the presence of silica gel, and the crude residue was purified by flash chromatography (solid loading, EtOAc/PE gradient, 3 mL/min flow rate), with the desired product **6** eluting at ~45% EtOAc. The title compound was obtained as a white solid (60.3 mg, 0.136 mmol, single trans-diastereomer, 57% yield). This compound has been previously characterized.[35] Crystals of **6** suitable for X-ray diffraction were obtained by crystallization from hexane/ethyl acetate. ^{1}H NMR (400 MHz, $CDCl_3$) δ 7.64 (d, *J* = 15.9 Hz, 1H), 7.19 (s, 1H), 7.03 (s, 1H), 6.91 (m, 3H), 6.31 (d, *J* = 15.9 Hz, 1H), 6.11 (d, *J* = 8.3 Hz, 1H), 5.65 (s, 1H), 4.33 – 4.23 (m, 5H, two overlapping quartets + CH furan), 3.92 (s, 3H), 3.88 (s, 3H), 1.34 (t, *J* = 7.1 Hz, 6H). HRMS (ESI, *m/z*): calculated for $C_{24}H_{26}O_8Na$ $[M+Na]^+$: 465.1519, found: 465.1523.

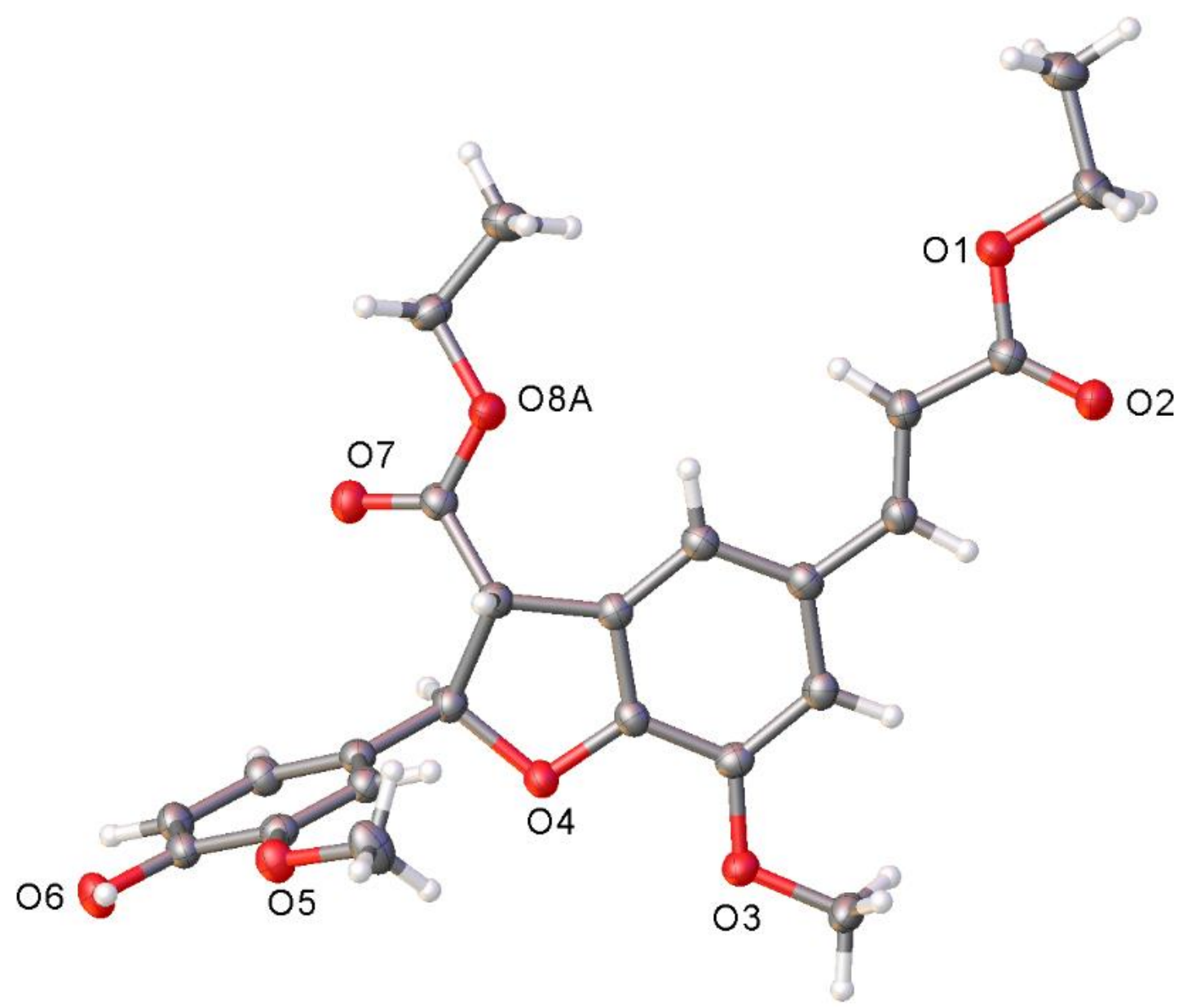

Crystal structure of **6**. Thermal ellipsoids are set to a 50% probability level. One ethyl group is disordered over two orientations, only one disordered fragment is shown for clarity.

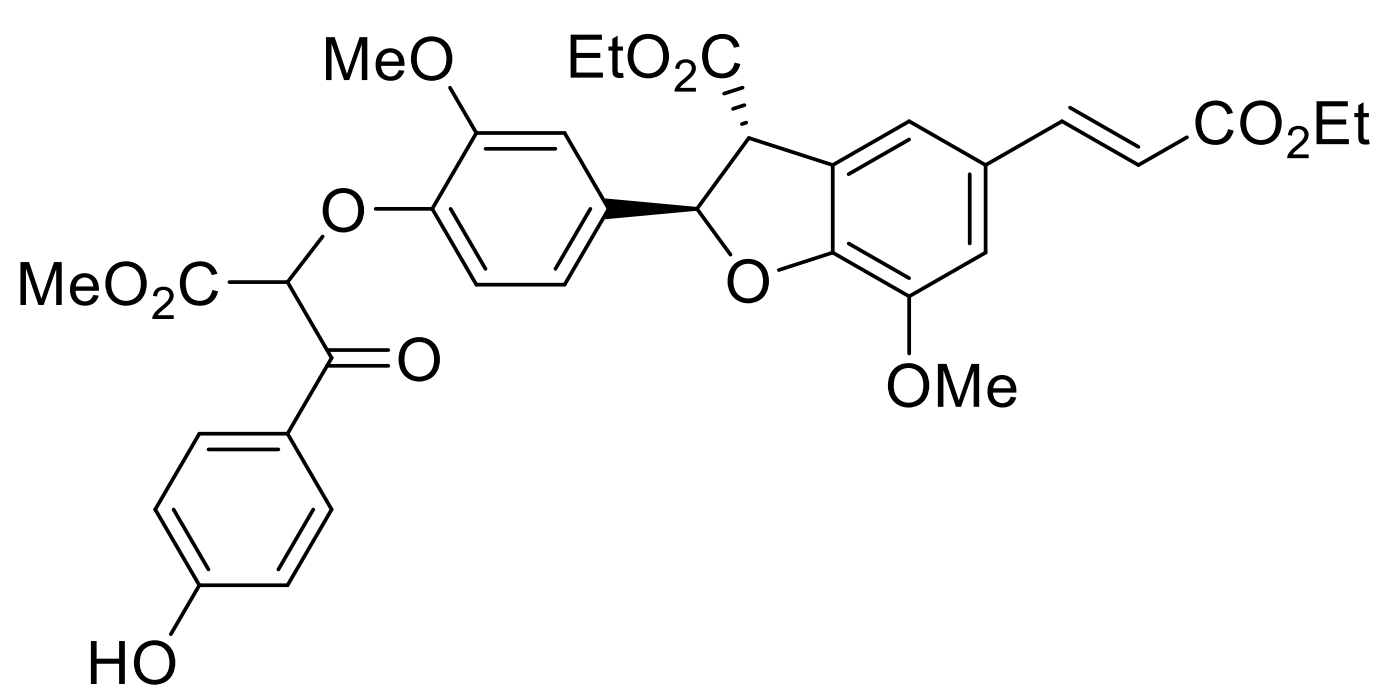

7a was synthesized following **the general procedure 6** on 0.3 mmol scale without addition DMF co-solvent, using compound **6** (132.6 mg, 0.3 mmol, 1.0 equiv.) and **2a** (192.6 mg, 0.45 mmol, 1.5 equiv.) as starting materials. The title compound was obtained as a brown oil (171.2 mg, 0.27 mmol, ~1:1 mixture of two diastereomers, 90% yield). ^{1}H NMR (400 MHz, $CDCl_3$) δ 8.03 (d, *J* = 8.1 Hz, 2H), 7.63 (d, *J* = 15.9 Hz, 1H), 7.17 (s, 1H), 7.01 (s, 1H), 6.92- 6.87 (m, 5H), 6.30 (d, *J* = 15.9 Hz, 1H), 6.10 (d, *J* = 8.1 Hz, 1H), 5.79 (s, 1H), 4.30 – 4.24 (m, 5H, two overlapping quartets + CH furan), 3.90 (s, 3H), 3.77 (s, 6H), 1.33 (m, 6H, two overlapping triplets). ^{13}C NMR (101 MHz, $CDCl_3$) δ 189.9, 170.4, 167.7, 162.0, 150.6, 149.9, 146.4, 144.8, 135.6, 132.6, 128.8, 126.7, 125.7, 118.7, 118.5, 118.1, 116.0, 115.8, 112.1, 110.6, 87.0, 82.3, 62.2, 60.8, 56.2, 56.1, 55.6, 53.2, 14.4, 14.3. HRMS (ESI, *m/z*): calculated for $C_{34}H_{34}O_{12}Na$ $[M+Na]^+$: 657.1942, found: 657.1936.

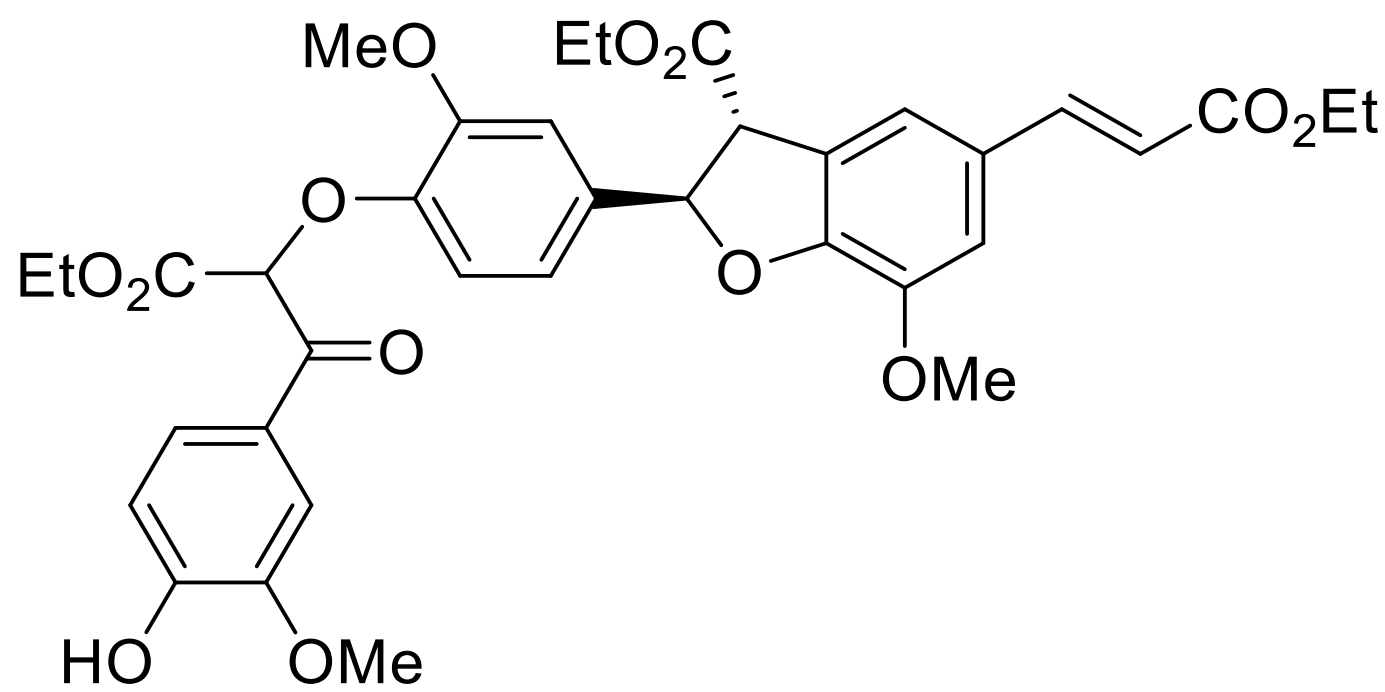

7b was synthesized following **the general procedure 6** on 0.3 mmol scale without addition DMF co-solvent, using compound **6** (132.6 mg, 0.3 mmol, 1.0 equiv.) and **2b** (212.4 mg, 0.45 mmol, 1.5 equiv.) as starting materials. The title compound was obtained as a light brown oil (185.2 mg, 0.279 mmol, ~1:1 mixture of two diastereomers, 93% yield). ^{1}H NMR (400 MHz, $CDCl_3$) δ 7.80 – 7.77 (m, 1H), 7.67 (d, *J* = 2.0 Hz, 1H), 7.62 (d, *J* = 15.9 Hz, 1H), 7.17 (s, 1H), 7.01 (s, 1H), 6.95 – 6.91 (m, 3H), 6.86 (d, *J* = 8.4 Hz, 1H), 6.37 (br s, 1H), 6.29 (d, *J* = 15.9 Hz, 1H), 6.10 (d, *J* = 8.0 Hz, 1H), 5.75 (s, 1H), 4.29 – 4.22 (m, 7H, three overlapping quartets + CH furan), 3.91 (s, 3H), 3.89 (s, 3H), 3.79 (s, 3H), 1.32 (t, *J* = 7.2 Hz, 6H), 1.24 – 1.19 (m, 3H). ^{13}C NMR (101 MHz, $CDCl_3$) δ 189.9, 170.21, 167.3, 167.0, 151.5, 150.6, 149.9, 146.7, 146.4, 144.8, 144.6, 135.4, 128.8, 126.9, 125.7, 125.5, 118.7, 118.3, 118.0, 116.1, 114.2, 112.0, 111.5, 110.6, 87.0, 82.5, 62.3, 62.0, 60.5, 56.2, 56.1, 56.1, 55.6, 14.4, 14.3, 14.1. HRMS (ESI, *m/z*): calculated for $C_{35}H_{36}O_{13}Na$ $[M+Na]^+$: 687.1933, found: 687.1931.

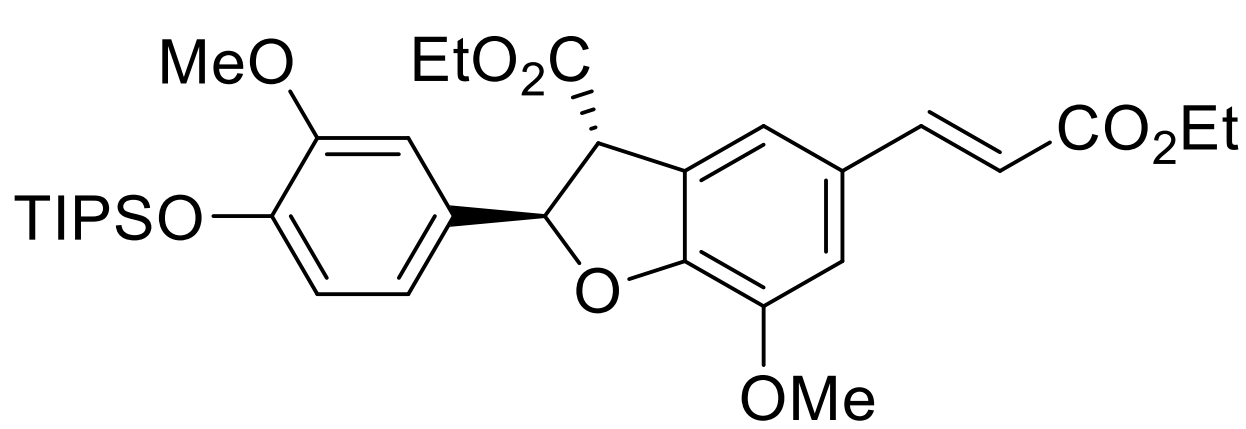

6-TIPS: compound **6** (673 mg, 1.52 mmol, 1.0 equiv.) and imidazole (204 mg, 3 mmol, 2.0 equiv.) were dissolved in dry DCM (9 mL), giving a clear solution. The mixture was cooled to 0 °C, and TIPSCl (353 µL, 1.65 mmol, 1.1 equiv.) was added portion-wise under stirring. The reaction mixture was allowed to warm up to room temperature, and the reaction was monitored by TLC (EtOAc/PE 1:9). After 3 h, complete consumption of the starting material was observed, and the reaction was quenched with water (5 mL). Organics were extracted with DCM (2 × 5 mL). The

combined organic layers were dried over anhydrous $MgSO_4$, filtered, and concentrated onto silicasilica gel. Purification with was performed with flash chromatography (solid loading, EtOAc/PE gradient, 7 mL/min flow rate) with product eluting at ~12% EtOAc. The title compound was obtained as a white solid (753.5 mg, 1.26 mmol, single trans-diastereomer, 83% yield).

^{1}H NMR (400 MHz, $CDCl_3$) δ 7.63 (d, *J* = 15.9 Hz, 1H), 7.18 (s, 1H), 7.02 (s, 1H), 6.88 (s, 1H), 6.83 (m, 2H), 6.30 (d, *J* = 15.9 Hz, 1H), 6.10 (d, *J* = 8.2 Hz, 1H), 4.32 – 4.23 (m, 5H, two overlapping quartets + CH furan), 3.91 (s, 3H), 3.78 (s, 3H), 1.35-1.31 (m, 6H, two overlapping triplets), 1.28 – 1.18 (m, 3H), 1.07 (d, *J* = 7.4 Hz, 18H).^{13}C NMR (101 MHz, $CDCl_3$) δ 170.4, 167.3, 151.1, 150.1, 146.1, 144.8, 144.7, 132.7, 128.7, 126.1, 120.5, 118.8, 118.0, 116.0, 112.0, 110.2, 87.7, 61.9, 60.5, 56.2, 55.7, 55.6, 18.0, 14.5, 14.4, 13.0. HRMS (ESI, *m/z*): calculated for $C_{33}H_{47}O_8Si$ $[M+H]^+$: 599.3034, found: 599.3035.

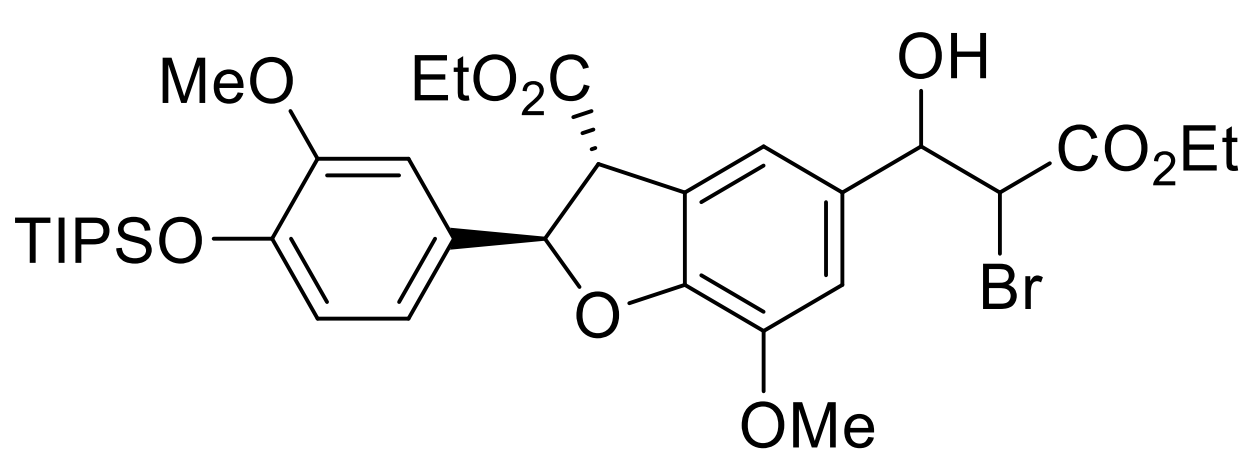

6-TIPS-Br-OH: In a 100 mL flask equipped with a stir bar, **6-TIPS** (1.00 g, 1.67 mmol, 1.0 equiv.) was suspended in 25 mL of *t*-BuOH/H_2O (4:1 v/v). To the resulting suspension, N-bromosuccinimide (NBS, 605 mg, 3.4 mmol, 2.0 equiv.) was added, followed by diphenyl thiourea (DPTU, 193.8 mg, 0.85 mmol, 0.5 equiv.). Upon addition of DPTU, the reaction mixture turned yellow. The mixture was stirred at room temperature and monitored by TLC (EtOAc/PE 1:3). After 12 h, the reaction mixture was quenched with water (10 mL) and extracted with EtOAc (3 × 15 mL). The combined organic layers were washed with brine (5 mL) and water (5 mL), dried over anhydrous $MgSO_4$, filtered, and concentrated under reduced pressure. The crude residue was adsorbed onto silica and purified by flash chromatography (solid loading, EtOAc/PE gradient, 7 mL/min flow rate), with the bromohydrin product eluting at ~30% EtOAc. The title compound was obtained as a yellow oil (1.00 g, 1.44 mmol, ~1:1 mixture of two diastereomers, 86% yield).

^{1}H NMR (400 MHz, $CDCl_3$) δ 7.02 (s, 1H), 6.90 (m, 2H), 6.83 (s, 2H), 6.07 (dd, *J* = 8.4, 4.3 Hz, 1H), 5.06 – 5.02 (m, 1H), 4.36 – 4.20 (m, 5H, two overlapping quartets + CH furan), 3.90 (s, 3H), 3.78 (s, 3H), 3.20 (dd, *J* = 14.0, 5.1 Hz, 1H), 1.35 – 1.27 (m, 6H), 1.28 – 1.18 (m, 3H), 1.07 (d, *J* = 7.3 Hz, 18H). ^{13}C NMR (101 MHz, $CDCl_3$) δ 170.3, 170.3, 169.3, 169.2, 150.8, 148.0, 148.0,

145.6, 145.6, 144.3, 144.2, 132.7, 132.6, 132.4, 125.2, 125.1, 120.1, 118.5, 118.4, 115.8, 115.6, 111.0, 110.9, 110.0 109.9, 87.0, 86.9, 75.1, 75.0, 62.2, 61.5, 55.9, 55.8, 55.4, 47.8, 47.7, 17.7, 14.1, 13.7, 12.7. HRMS (ESI, *m/z*): calculated for $C_{33}H_{47}O_9BrNaSi$ $[M+Na]^+$: 717.2064, found: 717.2055.

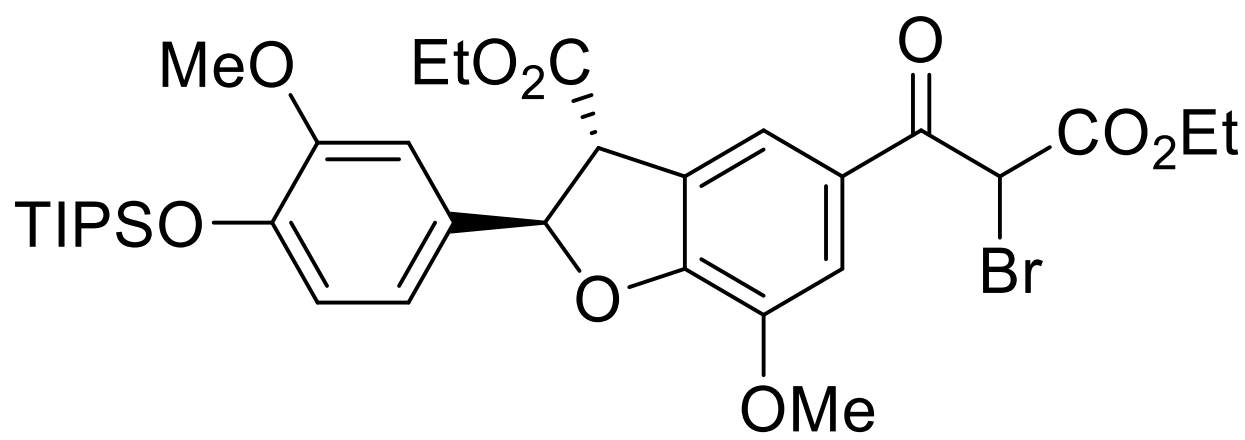

8: In a 100 mL flask equipped with a stir bar, **6-TIPS-Br-OH** (834 mg, 1.20 mmol, 1.0 equiv.) was dissolved in dry DCM (12 mL) and Dess-Martin periodinane (DMP, 560 mg, 1.32 mmol, 1.1 equiv.) was added in one portion. The reaction was stirred at room temperature and monitored by TLC (EtOAc/PE 1:4). After 1 h, the reaction mixture was concentrated under reduced pressure, and the residue was treated with diethyl ether (10 mL). The resulting suspension was washed successively with a 1:1 mixture of 10% $Na_2S_2O_3$ and saturated (20 mL), followed by brine (10 mL) and water (10 mL). The organic layer was dried over anhydrous $MgSO_4$, filtered, and concentrated onto silica gel. Purification was performed with flash chromatography (solid loading, EtOAc/PE gradient, 10 mL/min flow rate) with the desired bromoketoester **8** eluting at ~30-40% EtOAc. The title compound was obtained as a brown oil (757 mg, 1.09 mmol, ~1:1 mixture of two diastereomers, 91% yield). ^{1}H NMR (400 MHz, $CDCl_3$) δ 7.71 (s, 1H, overlapping two singlets), 7.57 (s, 1H), 6.86 (s, 1H), 6.84 (s, 2H), 6.19 (two overlapping doublets from two diastereomers with J = 7.9 Hz that appear as a triplet, 1H), 5.63 (s, 1H, CH-Br, overlapping two singlets), 4.37-4.26 (m, 5H, two overlapping quartets + CH furan), 3.94 (s, 3H), 3.78 (s, 3H), 1.36 (t, J = 7.1 Hz, 3H), 1.30 – 1.19 (m, 6H), 1.07 (d, J = 7.4 Hz, 18H). ^{13}C NMR (101 MHz, $CDCl_3$) δ 186.6, 186.5, 170.0 (04), 170.0 (98), 165.4 (41), 165.4 (38), 153.5, 151.2, 146.3, 145.0 (01), 145.0 (97), 132.2, 132.1, 127.6 (60), 127.6 (57), 125.7, 125.6, 120.6, 120.2, 120.1, 118.8 (80), 118.8 (76), 113.7 (72), 113.7, 110.2, 88.4, 88.3, 63.4, 62.2 (23), 62.2 (20), 56.4, 55.7, 55.3 (28), 55.3 (25), 46.6, 46.2, 18.0, 14.4 (40), 14.4 (36), 14.1, 14.0, 13.0. HRMS (ESI, *m/z*): calculated for $C_{33}H_{46}O_9BrSi$ $[M+H]^+$: 693.2089, found: 693.2084.

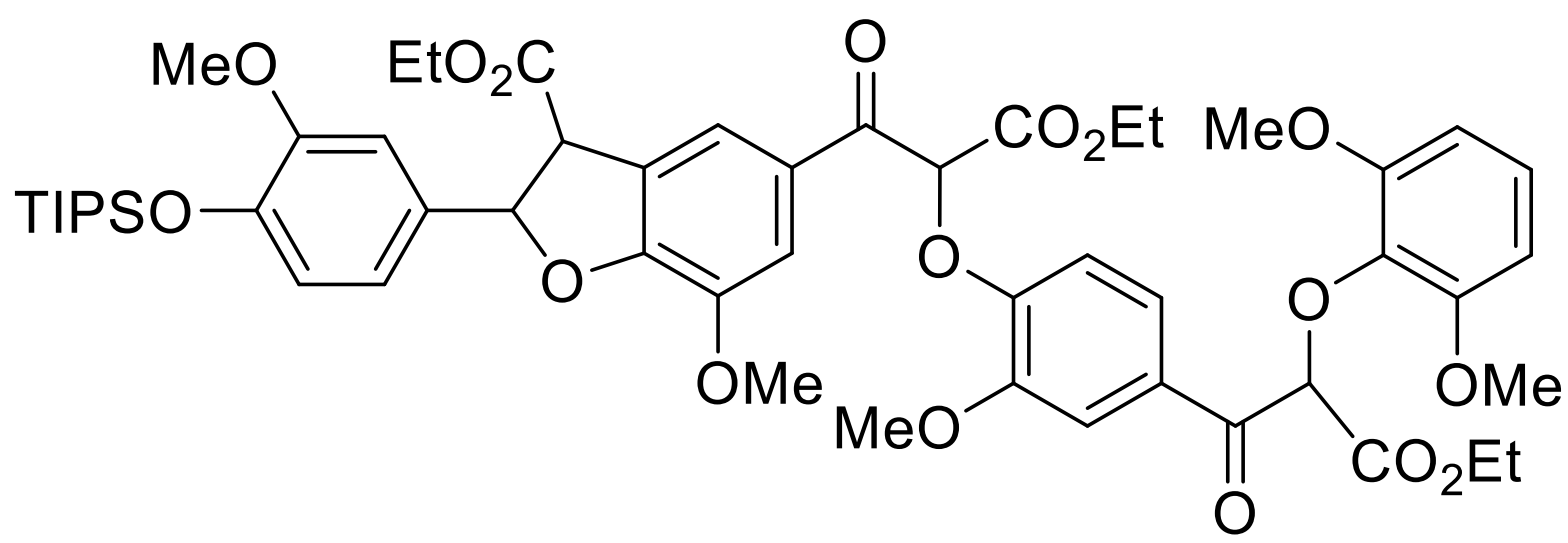

9-TIPS: unprotected dimer **3a** (56.2 mg, 0.144 mmol, 1.0 equiv.) and Cs_2CO_3 (140.4 mg, 0.432 mmol, 3.0 equiv.) were added to a 10 mL flask, which was subsequently purged with nitrogen for 15 minutes. A solvent THF (0.4 mL) was then added. The reaction mixture was stirred at room temperature for 30 min before a solution of bromoketoester **8** (150.0 mg, 0.216 mmol, 1.5 equiv) in dry THF (1.0 mL) was added dropwise. The reaction mixture was stirred at room temperature for 3h and reaction progress was monitored by TLC (EtOAc/PE 4:6) until complete consumption of the monomer and the formation of the TIPS-protected tetramer was observed. Upon completion, the reaction mixture was treated with water (3 mL) and extracted with EtOAc (2 × 3 mL). The combined organic layers were dried over anhydrous $MgSO_4$, filtered, and concentrated under reduced pressure. The crude residue was adsorbed onto silica gel and purified by flash chromatography (solid loading, EtOAc/PE gradient, 5 mL/min flow rate). The desired product **9-TIPS** eluted at 65% EtOAc. The title compound was obtained as a yellow oil (133 mg; 0.133 mmol; ~1:1:1:1 mixture of four diastereomers, 92% yield). ^{1}H NMR (400 MHz, $CDCl_3$) δ 7.94 – 7.89 (m, 1H, overlapping two triplets), 7.78 – 7.72 (m, 3H), 6.99 – 6.92 (m, 2H), 6.86 (s, 1H), 6.83 (s, 2H), 6.50 (d, J = 8.5 Hz, 2H), 6.14 (m, 1H, overlapping four singlets from diastereoisomers), 5.84 (d, J = 4.8 Hz, 1H), 5.52-5.48 (m, 1H, overlapping four singlets from diastereoisomers), 4.33 (d, J = 8.4 Hz, 1H), 4.29 – 4.21 (m, 6H), 3.93 (s, 3H), 3.89 – 3.84 (m, 3H), 3.77 (s, 3H), 3.65 (s, 6H), 1.31 – 1.19 (m, 12H),1.07 (d, J = 7.3 Hz, 18H). ^{13}C NMR (101 MHz, $CDCl_3$) δ 190.7, 190.6, 188.9 (90), 188.9 (87), 188.9 (85), 188.8, 170.1, 167.4, 166.4 (43), 166.4 (37), 153.4, 152.9, 152.8, 151.2, 150.5 (52), 150.5 (50), 150.5 (48), 149.9, 149.8, 146.3, 144.7, 136. 2 (21), 136.2 (19), 132.2, 132.1, 130.1 (13), 130.1 (12), 130.1 (09), 130.1 (07), 128.2, 128.1 (13), 128.1 (10), 128.1 (09), 125.8, 124.7, 124.6, 124.2 (18), 124.1 (16), 120.7, 120.6, 120.5, 118.8 (82), 118.8 (79), 115.7 (69), 115.7 (66), 115.5, 114.2, 113.2 (18), 113.2 (15), 110. 3 (31), 110.3 (30), 105.4, 88.4 (41), 88.4 (38), 85.3 (32), 85.3 (28), 85.2 (24), 85.2 (20), 82.3, 82.2, 62.6 (58), 62.6 (55), 62.2, 62.1, 62.0, 56.2, 56.1 (10), 56.1 (07), 56.0, 55.7, 55.4, 55.3, 18.0, 14.3, 14.2, 13.0. HRMS (ESI, *m/z*): calculated for $C_{53}H_{65}O_{17}Si$ $[M-H]^+$: 1001.3986, found: 1001.3985.

Reduction of Ketoesters into LMC1-9

General procedure 8: Ketoester **3a**, **4a, 4d, 4i** or **5** (1.0 equiv.) was dissolved in $CHCl_3$: MeOH (1:1, 1 mL total volume) and cooled to 0 °C. $NaBH_4$ was added portion-wise at 0 °C. The reaction progress was monitored by TLC (5% methanol in DCM). Upon completion (5-20 h), the reaction mixture was treated with saturated aqueous NH_4Cl and stirred for an additional 15 min at room temperature. The mixture was extracted with EtOAc, and the combined organic layers were dried over anhydrous $MgSO_4$, filtered, and concentrated under reduced pressure. The crude product was purified by flash chromatography using MeOH/DCM gradient to afford corresponding LMCs.

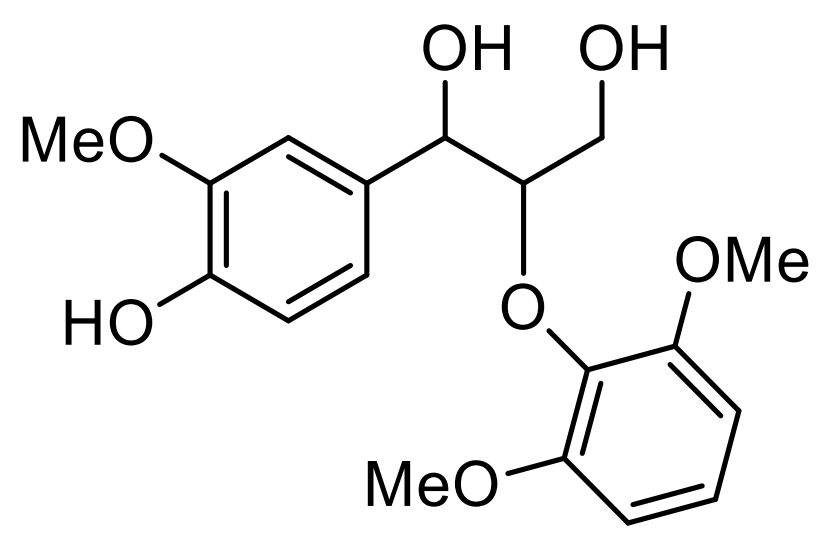

LMC1 was synthesized following **the general procedure 8** using **3a** (70.2 mg, 0.180 mmol) as the starting material and $NaBH_4$ (136.2 mg, 3.6 mmol, 20 equiv.). The title compound was obtained as a colorless oil after flash chromatography (solid loading, MeOH/DCM gradient, 3 mL/min flow rate, product eluted at 5% MeOH); (55.3 mg, 0.158 mmol, mixture of diastereomers, 88% yield). ^{1}H NMR (400 MHz, $CDCl_3$) δ 7.09 – 7.03 (m, 1H), 6.98-6.95 (m, 1H), 6.89 – 6.84 (m, 1H), 6.76 – 6.74 (m, 1H), 6.64 (dd, J = 8.4, 4.5 Hz, 2H), 5.71 (br s, 1H), 5.05 – 5.01 (m, 1H, overlapping two doublets from diastereoisomers), 4.17 – 4.13 (m, 1H), 3.90 (s, 3H), 3.88 (s, 6H), 3.83 – 3.79 (m, 1H), 3.60 – 3.49 (m, 1H), 3.36 – 3.30 (m, 1H), 3.17 (br s, 1H). ^{13}C NMR (101 MHz, $CDCl_3$) δ153.7, 153.4, 146.8, 146.6, 145.5, 145.0, 135.5, 135.1, 132.1, 131.4, 124.6 (62), 124.6 (60), 120.5, 118.9, 114.4, 114.3, 110.0, 108.5, 105.4 (44), 105.4 (42), 89.2, 87.2, 74.2, 72.6, 60.7, 60.6, 56.3, 56.1(09), 56.1(07). HRMS (ESI, m/z): calculated for $C_{18}H_{22}O_7$[M+Na]$^+$: 373.1258, found: 373.1260.

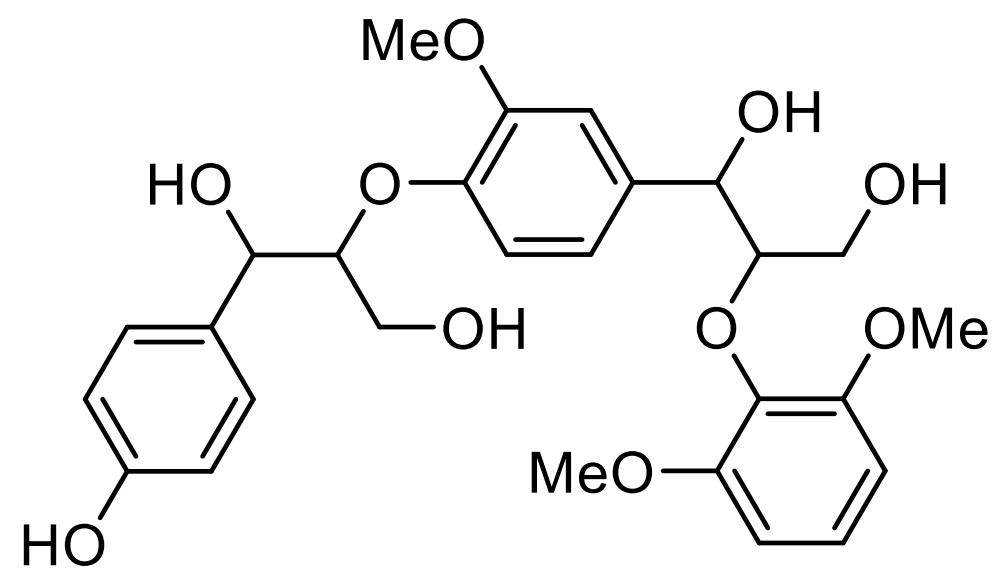

LMC2 was synthesized following **the general procedure 8** using compound **4a** (104.8 mg, 0.180 mmol) as the starting material and $NaBH_4$ (272.4 mg, 7.2 mmol, 40 equiv.). The title compound was obtained as a yellow oil after flash chromatography (solid loading, MeOH/DCM gradient, 3 mL/min flow rate, product eluted at 5% MeOH); (69.7 mg, 0.135 mmol, mixture of diastereomers, 75% yield). ^{1}H NMR (400 MHz, acetone-d_6) δ 8.19 (s, 1H), 7.28 (d, J = 8.6 Hz, 2H), 7.11 – 6.98 (m, 2H), 6.95 – 6.83 (m, 2H), 6.79 – 6.76 (m, 2H), 6.70 (d, J = 8.4 Hz, 2H), 5.02 – 4.97 (m, 1H), 4.88 (t, J = 5.0 Hz, 1H), 4.49 (s, 1H, overlapping two singlets), 4.45 – 4.36 (m, 1H), 4.23 – 4.20 (m, 1H), 4.17 – 4.14 (m, 1H), 3.86 – 3.80 (m, 9H, overlapping three methoxy groups), 3.72 – 3.61 (m, 2H), 3.48 – 3.38 (m, 1H), 3.30 – 3.29 (m, 1H). ^{13}C NMR (101 MHz, acetone-d_6) δ 157.3, 154.3, 154.0, 151.5, 151.4, 148.0, 147.7, 137.0, 136.9, 136.4, 133.6, 128.7, 124.7, 124.6, 120.3, 119.8, 119.4 (43), 119.4 (38), 119.3 115.4, 112.2, 112.1, 111.7 (74), 111.7 (72), 106.4, 106.3, 89.0, 87.5, 87.4, 87.1 (13), 87.1 (10), 87.1 (06), 73.6, 73.5, 73.0, 61.6 (62), 61.6 (60), 61.6 (56), 61.2, 60.7, 56.4, 56.2, 56.1. HRMS (ESI, m/z): calculated for $C_{27}H_{31}O_{10}[M-H]^{+}$: 515.1912, found: 515.1920.

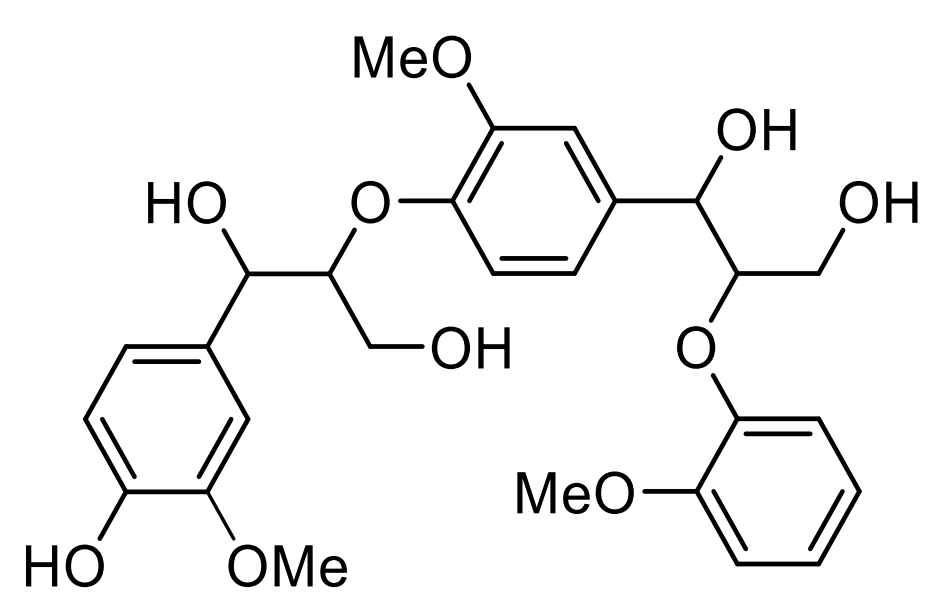

LMC3 was synthesized following **the general procedure 8** using compound **4d** (356.0 mg, 0.59 mmol, 1.0 equiv.) as the starting material and $NaBH_4$ (893.0 mg, 23.6 mmol, 40 equiv.). The title compound was obtained as a yellow oil after flash chromatography (solid loading, MeOH/DCM gradient, 7 mL/min flow rate, product eluted at ~5-7% MeOH); (228.0 mg, 0.44 mmol, mixture of diastereomers, 75% yield). ^{1}H NMR (400 MHz, acetone-d_6) δ 7.44 (m, 1H), 7.18 – 7.07 (m, 3H),

7.02 – 6.81 (m, 6H), 6.79 – 6.74 (m, 1H), 4.97 – 4.84 (m, 2H), 4.54 – 4.44 (m, 1H), 4.43 – 4.37 (m, 1H), 4.35 – 4.12 (m, 2H), 3.87– 3.76 (m, 11H, overlapping three methoxy groups + CH_2), 3.75 – 3.65 (m, 3H), 3.56 – 3.43 (m, 1H). ^{13}C NMR (101 MHz, acetone-d_6) δ 151.1, 150.9, 150.7 (67), 150.7 (65), 150.6, 150.5 (49), 150.5 (46), 150.4, 148.7, 148.2, 148.00, 147.9, 147.4, 147.2 (24), 147.2 (16), 147.1, 146.00, 145.8, 136.6 (64), 136.6 (62), 136.5, 136.2, 136.0, 133.5, 133.0, 122.6, 122.5, 121.1, 121.0, 119.7, 119.6 (61), 119.6 (57), 119.5 (52), 119.5 (49), 119.5 (47), 119.0 (04), 119.0 (00), 118.8 (80), 118.8 (78), 118.5, 118.4, 118.2, 118.1, 114.4, 114.3, 112.6, 112.5, 111.5, 111.4 (43), 111.4 (39), 111.4 (36), 111.3, 110.5, 87.8 (82), 87.8 (79), 87.7, 87.3 (32), 87.3 (25), 86.0, 85.9, 85.7 (72), 85.7 (70), 85.7 (67), 85.7 (65), 73.0, 72.9, 72.8 (84), 72.8 (81), 61.0 (99), 61.0 (96), 60.9, 55.4 (41), 55.4 (38), 55.3 (34), 55.3 (32). HRMS (ESI, m/z): calculated for $C_{27}H_{31}O_{10}$[M-H]$^+$: 515.1912, found: 515.1919.

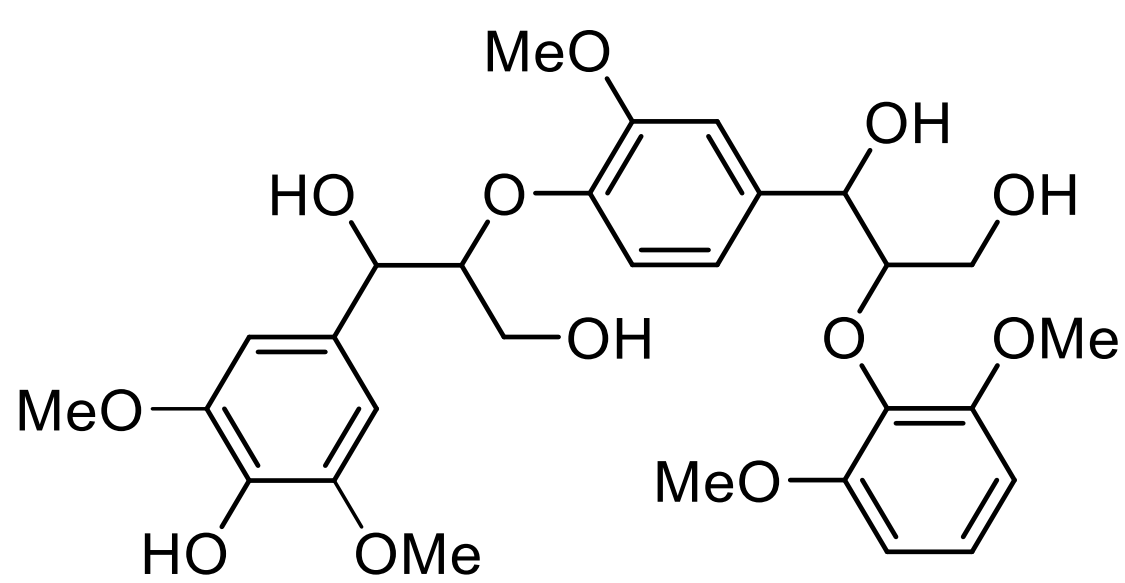

LMC4 was synthesized following **the general procedure 8** using compound **4i** (25.0 mg, 0.039 mmol) as the starting material and $NaBH_4$ (59.0 mg, 1.56 mmol, 40 equiv.). The title compound was obtained as a yellow oil after flash chromatography (solid loading, MeOH/DCM gradient, 3 mL/min flow rate, product eluted at 7% MeOH); (15.5 mg, 0.027 mmol, mixture of diastereomers, 69% yield). ^{1}H NMR (400 MHz, acetone-d_6) δ 7.11 – 6.99 (m, 3H), 6.94 – 6.89 (m, 1H), 6.88 – 6.83 (m, 1H), 6.77 (d, J = 2.8 Hz, 1H), 6.70 (d, J = 8.5 Hz, 2H), 5.03 – 4.94 (m, 1H), 4.89 – 4.86 (m, 1H), 4.55 – 4.53 (m, 1H), 4.47 – 4.39 (m, 1H), 4.32 – 4.27 (m, 1H), 4.20 – 4.14 (m, 1H), 4.01 – 3.96 (m, 1H), 3.87 – 3.78 (m, 15H, overlapping five methoxy groups), 3.77 – 3.64 (m, 2H), 3.48 – 3.32 (m, 2H). ^{13}C NMR (101 MHz, acetone-d_6) δ 154.5, 154.1, 151.4 (43), 151.4 (41), 151. 4 (38), 151.4 (36), 148.4, 147.9 (93), 147.9 (91), 137.0, 136.9, 136.8, 136.7, 136.6, 136.0, 133.3 (30), 133.3 (26), 124.9, 124.8, 120.5, 120.0, 118.9, 118.8, 112.3, 112.2, 112.1 (14), 112.1 (11), 111.9, 111.8, 106.5, 106.4, 105.4, 89.3, 87.6, 87.6, 87.5, 86.8, 86.7, 86.6 (58), 86.6 (55), 86.5, 74.1, 74.0 (04), 74.0 (02), 73.8, 73.2 (23), 73.2 (21), 73.2 (16), 62.1, 61.9 (90), 61.9 (86), 61.4,

60.9, 56.6 (60), 56.6 (59), 56.5, 56.3 (31), 56.3 (31), 56.3 (27). HRMS (ESI, m/z): calculated for $C_{29}H_{36}O_{12}Na[M+Na]^+$: 599.2099, found: 599.2095.

General procedure 9: Ketoester **6**, **7a**, or **7b** (1.0 equiv.) was dissolved in dry THF (1 mL) under a nitrogen atmosphere and cooled to 0 °C. DIBAL-H (1 M toluene, 3.0 equiv. per carbonyl group) was added dropwise with stirring at 0°C. The reaction progress was monitored by TLC (EtOAc/PE 9.5:0.5 and EtOAc/MeOH 9.5:0.5). Upon complete consumption of the starting material, the reaction was quenched by slowly adding EtOAc (2-4 mL) followed by dropwise addition of saturated Rochelle salt solution (3-6 mL) at cooling with ice bath. The mixture was stirred for 30 min, then allowed to stand at room temperature for 15 min. to facilitate phase separation. The aqueous layer was extracted with EtOAc (2 × 1 mL). The organic layer was dried with $MgSO_4$, filtered, and concentrated under reduced pressure. The crude product was purified by flash chromatography using a MeOH/ DCM gradient as a mobile phase.

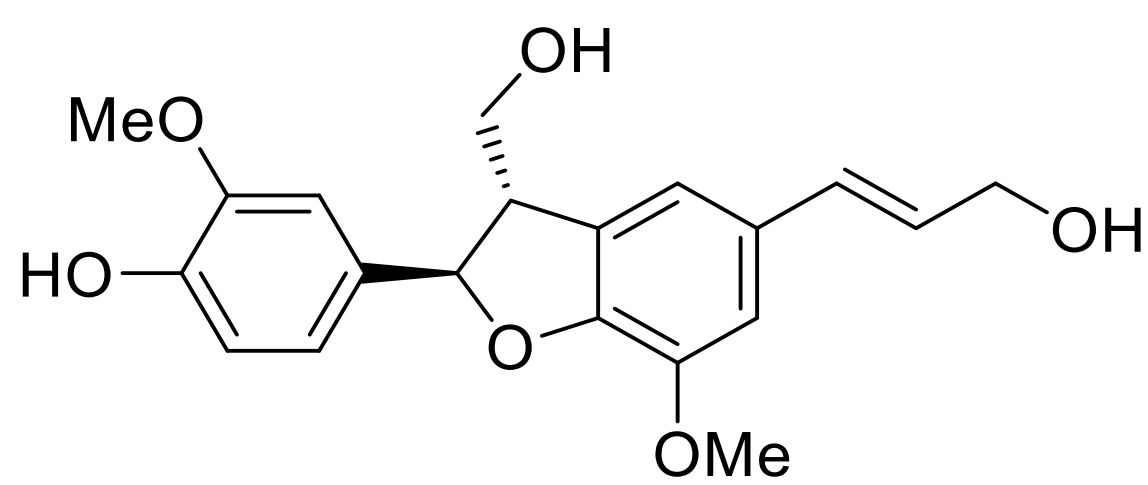

LMC5 was synthesized following **the general procedure 9** using compound **6** (48.6 mg, 0.110 mmol,) as the starting material and 6.0 equiv. of DIBAL-H. The title compound was obtained as a white foam after flash chromatography (solid loading, MeOH/DCM gradient, 5 mL/min flow rate, product eluted at 9% MeOH); (37.4 mg, 0.104 mmol, single trans-diastereomer, 95% yield). This compound has been previously characterized.[35] Crystals of **LMC5** suitable for X-ray diffraction were prepared by dissolving the solid in hot hexane, slowly cooling the solution, then storing it at −20 °C to promote crystal formation. ^{1}H NMR (400 MHz, acetone-d$_6$) δ 7.60 (s, 1H), 7.04 (d, J = 2.0 Hz, 1H), 6.98 (s, 1H), 6.95 (s, 1H), 6.89 (dd, J = 8.2, 2.0 Hz, 1H), 6.81 (d, J = 8.1 Hz, 1H), 6.56 – 6.49 (m, 1H), 6.28 – 6.21 (m, 1H), 5.57 (d, J = 6.6 Hz, 1H), 4.20 (d, J = 5.6 Hz, 2H), 3.89 (m, 1H), 3.86 (s, 3H), 3.83 (m, 1H), 3.81 (s, 3H), 3.54 (m, 1H), 2.99 (br s, 1H).

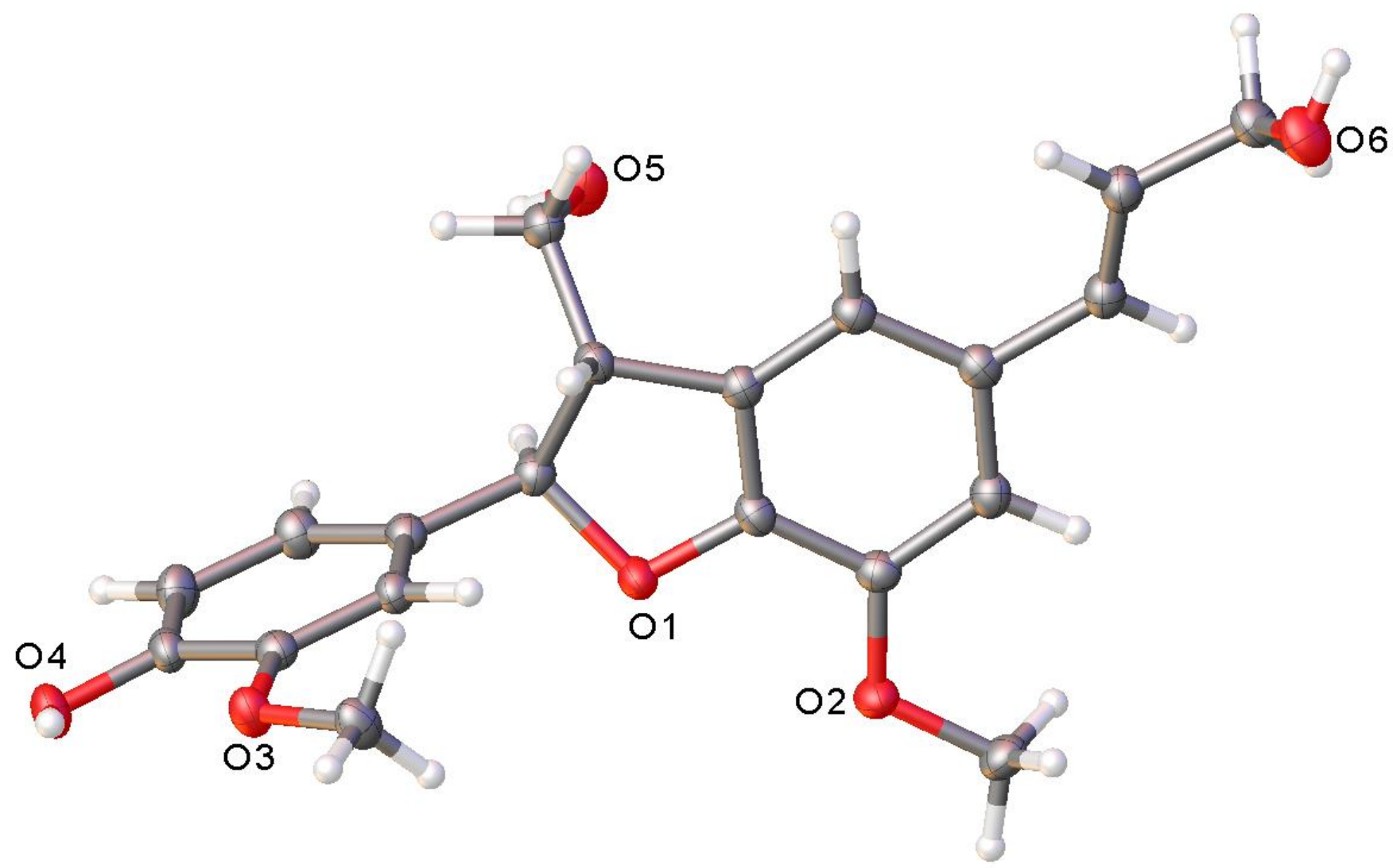

Crystal structure of **LMC5**. Thermal ellipsoids are set to a 50% probability level.

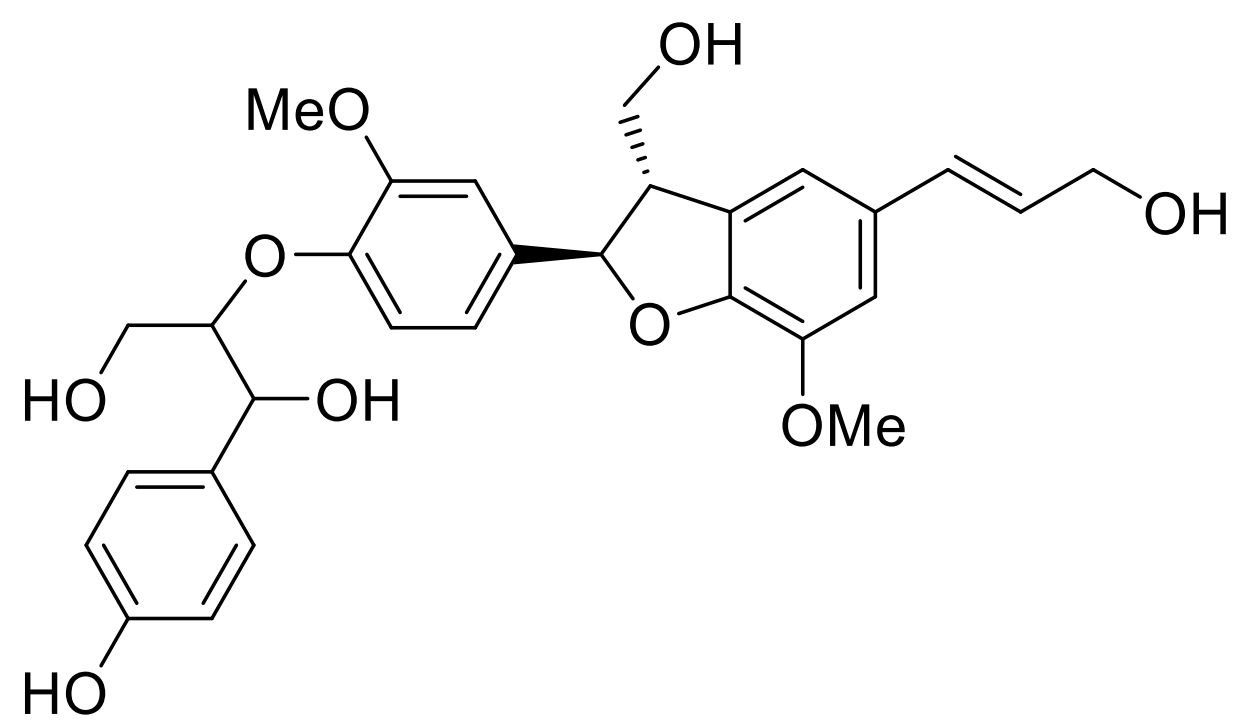

LMC6 was synthesized according to **the general procedure 9** using compound **7a** (0.110 mmol, 69.7 mg) as the starting material and 12.0 equiv. of DIBAL-H. The title compound was obtained as white oil after flash chromatography (solid loading, MeOH/DCM gradient, 5 mL/min flow rate, product eluted at 9% MeOH); (52.4 mg; 0.10 mmol; 91% yield). ^{1}H NMR (400 MHz, acetone-d$_6$) δ 8.26 (d, J = 16.9 Hz, 1H), 7.29 (d, J = 8.5 Hz, 2H), 7.16 (d, J = 8.3 Hz, 1H), 7.07 – 7.03 (m, 1H), 6.97 – 6.92 (m, 2H), 6.90 – 6.87 (m, 1H), 6.80 – 6.77 (m, 2H), 6.53 (d, J = 15.0 Hz, 1H), 6.28 – 6.21 (m, 1H), 5.59 (d, J = 6.7 Hz, 1H), 4.90 – 4.87 (m, 1H), 4.38 (d, J = 3.6 Hz, 1H), 4.28 – 4.24 (m, 1H), 4.22 – 4.17 (m, 3H), 3.92 – 3.88 (m, 1H), 3.87 (s, 3H, overlapping two singlets), 3.85 (s, 2H), 3.83 – 3.79 (m, 3H), 3.76 – 3.65 (m, 2H), 3.56 – 3.44 (m, 2H), ^{13}C NMR (101 MHz, acetone-d$_6$) δ 157.7, 157.5, 151.9, 151.8, 149.4, 148.9, 148.7 (73), 148.7 (71), 145.2, 137.5 (52), 137.5 (45), 137.4, 133.7, 133.2, 132.1, 130.5, 130.2, 129.1, 128.9, 128.5, 119.9, 119.6, 119.5, 119.3 (32), 119.3 (28), 119.2 (22), 119.2 (16), 116.1, 115.7, 115.5, 111.7 (75), 111.7 (73), 111.4 (37), 111.4

(35), 111.2 (22), 111.2 (20), 88.8 (79), 88.8 (77), 88.3, 88.2, 87.0, 73.8, 73.7, 64.7, 63.4, 61.8 (84), 61.8 (79), 56.4, 55.0, 54.9. HRMS (ESI, *m/z*): calculated for $C_{29}H_{32}O_9Na$ $[M+Na]^+$: 547.1939, found: 547.1932.

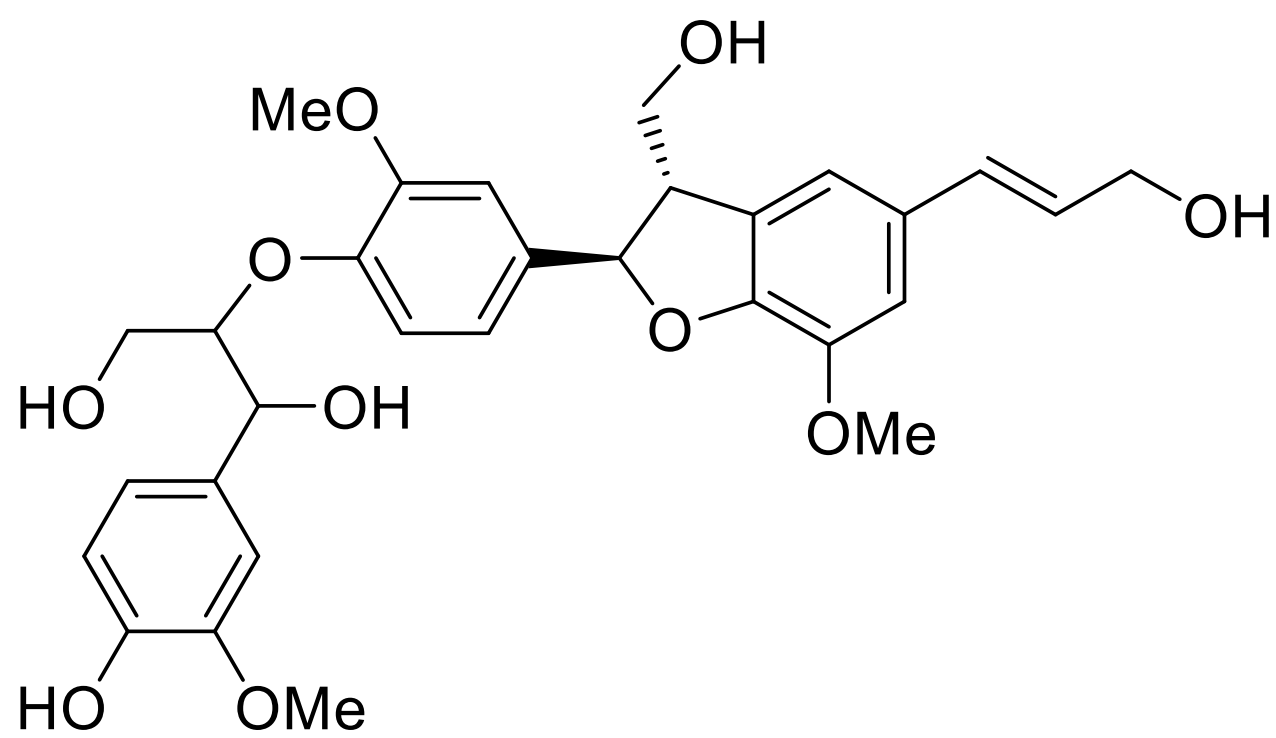

LMC7 was synthesized according to **the general procedure 9** using compound **7b** (0.052 mmol, 35.3 mg) as the starting material and 12.0 equiv. of DIBAL-H. Title compound was obtained as a light-yellow oil after flash chromatography (solid loading, MeOH/DCM gradient , 3 mL/min flow rate, product eluted at 9% MeOH); (25.5 mg; 0.046 mmol; 89% yield). ^{1}H NMR (400 MHz, acetone-d_6) δ 7.45 (d, *J* = 15.1 Hz, 1H), 7.15 (d, *J* = 8.3 Hz, 1H), 7.11 – 7.03 (m, 2H), 6.98 – 6.93 (m, 2H), 6.93 – 6.86 (m, 2H), 6.78 – 6.75 (m, 1H, overlapping four singlets), 6.55 – 6.50 (m, 1H), 6.28 – 6.21 (m, 1H), 5.63 – 5.55 (m, 1H), 4.90 – 4.86 (m, 1H), 4.42 – 4.41 (m, 1H), 4.23 – 4.17 (m, 3H), 4.04 (s, 1H), 3.93 – 3.89 (m, 2H), 3.87 (s, 3H, overlapping two singlets), 3.85 – 3.82 (m, 3H), 3.80 (s, 3H, overlapping two singlets), 3.74 – 3.66 (m, 2H), 3.56 – 3.46 (m, 2H). ^{13}C NMR (101 MHz, acetone-d_6) δ 151.8 (81), 151.8 (79), 151.7, 149.4, 148.9, 148.8 (76), 148.8 (75), 148.0 (04), 148.0 (97), 146.8, 146.7, 145.2 (20), 145.2 (19), 137.4, 134.3, 133.9, 132.1 (07), 132.1 (05), 130.7, 130.5, 130.2, 128.5 (46), 128.5 (45), 120.6, 120.5, 119.5, 119.3 (29), 119.3 (27), 119.2 (19), 119.2 (15), 119.1 (12), 119.1 (10), 116.1, 115.2, 115.1, 111.7, 111.4 (41), 111.4 (38), 111.3 (30), 111.3 (26), 111.2 (17), 111.2 (15), 88.4, 88.3, 86.6 (61), 86.6 (59), 73.8, 64.7, 63.7, 63.4, 61.9 (92), 61.9 (89), 56.4 (41), 56.4 (38), 56.2 (21), 56.2 (20), 54.9. HRMS (ESI, *m/z*): calculated for $C_{30}H_{34}O_{10}Na$ $[M+Na]^+$: 577.2044, found: 577.2037.

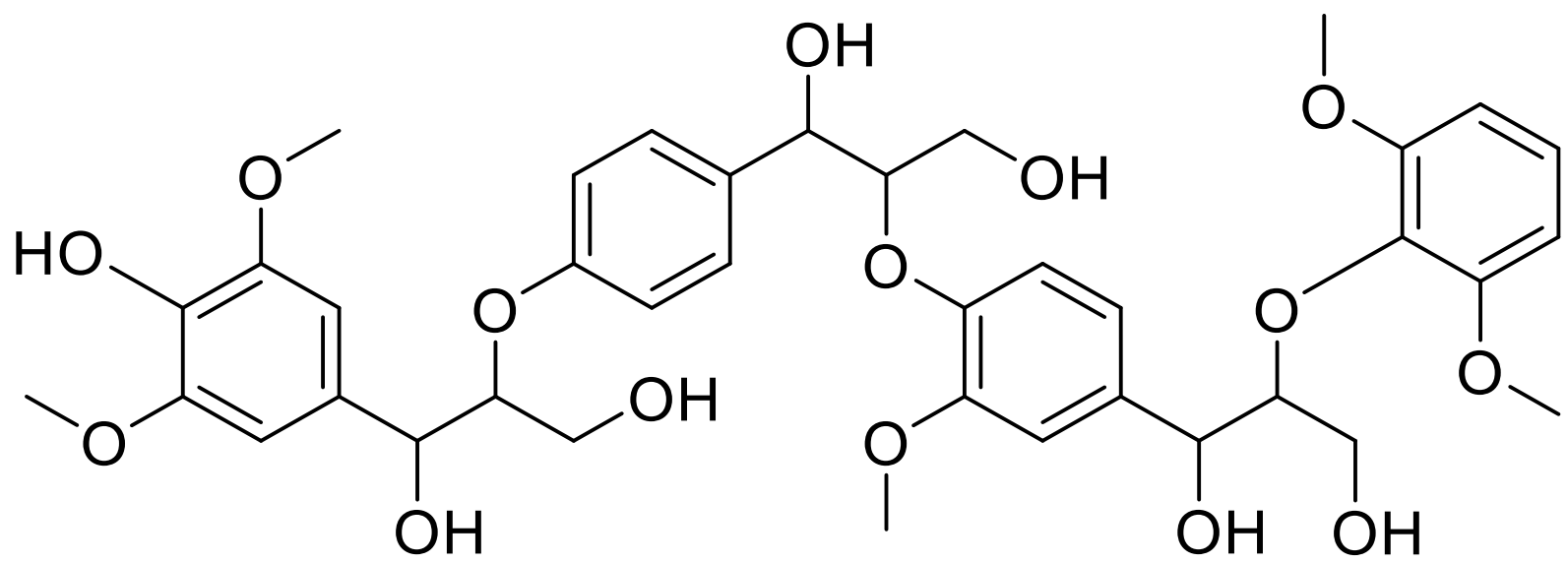

LMC8 was synthesized according to **the general procedure 8** using compound **5** (56 mg, 0.067 mmol) as the starting material and $NaBH_4$ (152.1 mg, 4.02 mmol, 60 equiv.). The title compound was obtained as dark yellow oil after flash chromatography (solid loading, MeOH/DCM gradient ,3 mL/min flow rate, product eluted at 14% MeOH); (37.6 mg, 0.051 mmol, mixture of diastereomers, 76% yield). ^{1}H NMR (400 MHz, acetone-d$_6$) δ 7.33– 7.27 (m, 2H), 7.14 – 7.00 (m, 3H), 6.97 – 6.90 (m, 2H), 6.88– 6.84 (m, 2H), 6.76– 6.74 (m, 2H), 6.72 (d, *J* = 2.7 Hz, 1H), 6.70 (d, *J* = 2.7 Hz, 1H), 5.05 – 4.97 (m, 1H), 4.93– 4.85 (m, 2H), 4.54 – 4.36 (m, 3H), 4.25 – 4.10 (m, 1H), 4.01 – 3.94 (m, 1H), 3.89 – 3.85 (m, 6H, overlapping two singlet methoxy groups), 3.85 – 3.83 (m, 2H), 3.82 – 3.76 (m, 9H, overlapping three singlet methoxy groups), 3.72 – 3.63 (m, 2H), 3.60 – 3.51 (m, 1H), 3.50– 3.39 (m, 1H), 3.34– 3.28 (m, 1H). ^{13}C NMR (101 MHz, acetone-d$_6$) δ 159.6, 159.4, 159.2, 159.0, 154.5, 154.2, 151.6, 151.4, 148.8, 148.4 (37), 148.4 (35), 137.1, 136.0, 135.3, 135.2, 134.7 (73), 134.7 (68), 133.5, 133.3, 128.9, 128.8 (82), 128.8 (79), 128.8 (77), 128.7 (73), 128.7 (70), 124.9 (93), 124.9 (85), 120.6, 120.5 (50), 120.5 (47), 119.7, 119.6, 119.4 (44), 119.4 (40), 119.4 (38), 116.7, 116.6 (62), 116.6 (56), 116.5 (54), 116.5 (48), 112.3 (33), 112.3 (28), 112.2 (21), 112.2 (16), 106.5 (55), 106.5 (46), 105.4 (43), 105.4 (40), 105.3, 89.3, 89.2, 88.7, 87.6, 87.0, 84.2, 84.1, 83.7, 74.2, 74.1, 73.9, 73.6, 73.5, 62.1, 61.8, 61.4 (41), 61.4 (37), 60.9, 56.6 (63), 56.6 (60), 56.6 (56), 56.3 (31), 56.3 (30). HRMS (ESI, m/z): calculated for $C_{38}H_{46}O_{15}Na[M+Na]^+$:765.2729, found: 765.2713.

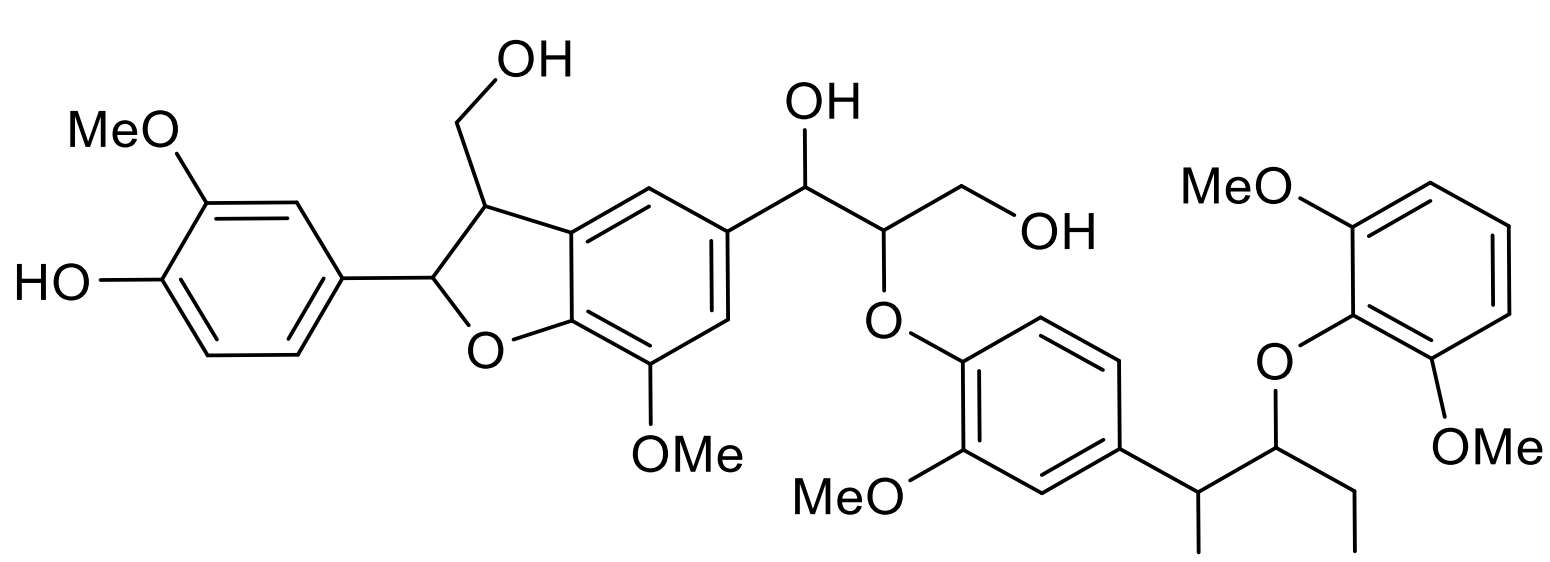

LMC9: Ketoster **9-TIPS** (50.1 mg, 0.05 mmol, 1.0 equiv.) was dissolved in $CHCl_3$: MeOH (1:1, 1 mL total volume) and cooled to 0 °C. $NaBH_4$ (94.6 mg, 2.5 mmol, 50 equiv.) was added portion-

wise at 0 °C. The reaction progress was monitored by TLC (5% methanol in DCM). Upon completion, the reaction mixture was treated with saturated aqueous NH_4Cl (2 mL) and stirred for an additional 15 min at room temperature. The mixture was extracted with EtOAc (2 × 1 mL), and the combined organic layers were dried over anhydrous $MgSO_4$, filtered, and concentrated under reduced pressure. The crude product was purified by flash chromatography using MeOH/DCM gradient to afford corresponding **LMC9-TIPS**. Subsequently, **LMC9-TIPS** (27.0 mg, 0.031 mmol, 1.0 equiv.) was dissolved in 0.2 mL THF, then TBAF·xH_2O (8.09 mg, 0.0269 mmol, 15 wt% H_2O) was added in 100 µL of THF. The mixture was stirred at room temperature and monitored by TLC (MeOH/DCM = 1:9). Upon completion, the reaction was quenched with saturated aqueous NH_4Cl (1 mL) and extracted with EtOAc (2 × 1 mL). The combined organic layers were dried over anhydrous $MgSO_4$, filtered and concentrated. The crude product was purified using preparative TLC (MeOH/DCM = 0.4:9.6). The title compound was obtained as a light-yellow oil (15.7 mg, 0.022 mmol, mixture of diastereomers, 70% yield). ^{1}H NMR (400 MHz, acetone-d_6) δ 7.56 (s, 1H), 7.15 – 7.06 (m, 2H), 7.05 – 6.97 (m, 4H), 6.93 – 6.85 (m, 2H), 6.80 (d, *J* = 8.1 Hz, 1H), 6.71 (d, *J* = 8.4 Hz, 2H), 5.56 – 5.53 (m, 1H), 5.05 – 5.00 (m, 1H), 4.89 – 4.85 (m, 1H), 4.52 (d, *J* = 5.5 Hz, 1H), 4.47 – 4.34 (m, 2H), 4.30 – 4.14 (m, 2H), 4.10 – 4.02 (m, 1H), 4.02 – 3.96 (m, 2H), 3.88 – 3.82 (m, 12H, overlapping four singlet methoxy groups), 3.80 (s, 3H, overlapping two singlet), 3.75 – 3.63 (m, 4H), 3.58 – 3.51 (m, 3H), 3.35 – 3.28 (m, 1H). ^{13}C NMR (101 MHz, acetone-d_6) δ 154.5, 154.2, 151.3 (31), 151.3 (28), 148.8, 148.6, 148.5, 148.4, 148.2, 147.2, 144.8, 144.7, 137.1, 137.0, 136.1, 135.6, 134.6, 129.7, 129.6, 124.9 (93), 124.9 (85), 120.6 (61), 120.6 (59), 120.5, 120.2, 119.9, 119.6, 119.4, 116.6, 116.4, 115.7, 112.6, 112.4, 112.3, 112.2, 110.5, 106.6, 106.5, 89.3, 88.7, 88.4 (44), 88.4 (41), 74.0, 73.9, 64.8, 64.7, 62.0, 61.9, 61.4, 56.6, 56.4, 56.4, 56.3 (34), 56.3 (29), 56.3, 55.0. HRMS (ESI, m/z): calculated for $C_{38}H_{44}O_{14}Na[M+Na]^+$: 747.2623, found: 747.2615.

NMR Spectra of Synthesized Compounds

NMR Spectra of Building Blocks 2a-c and Synthetic Intermediates:

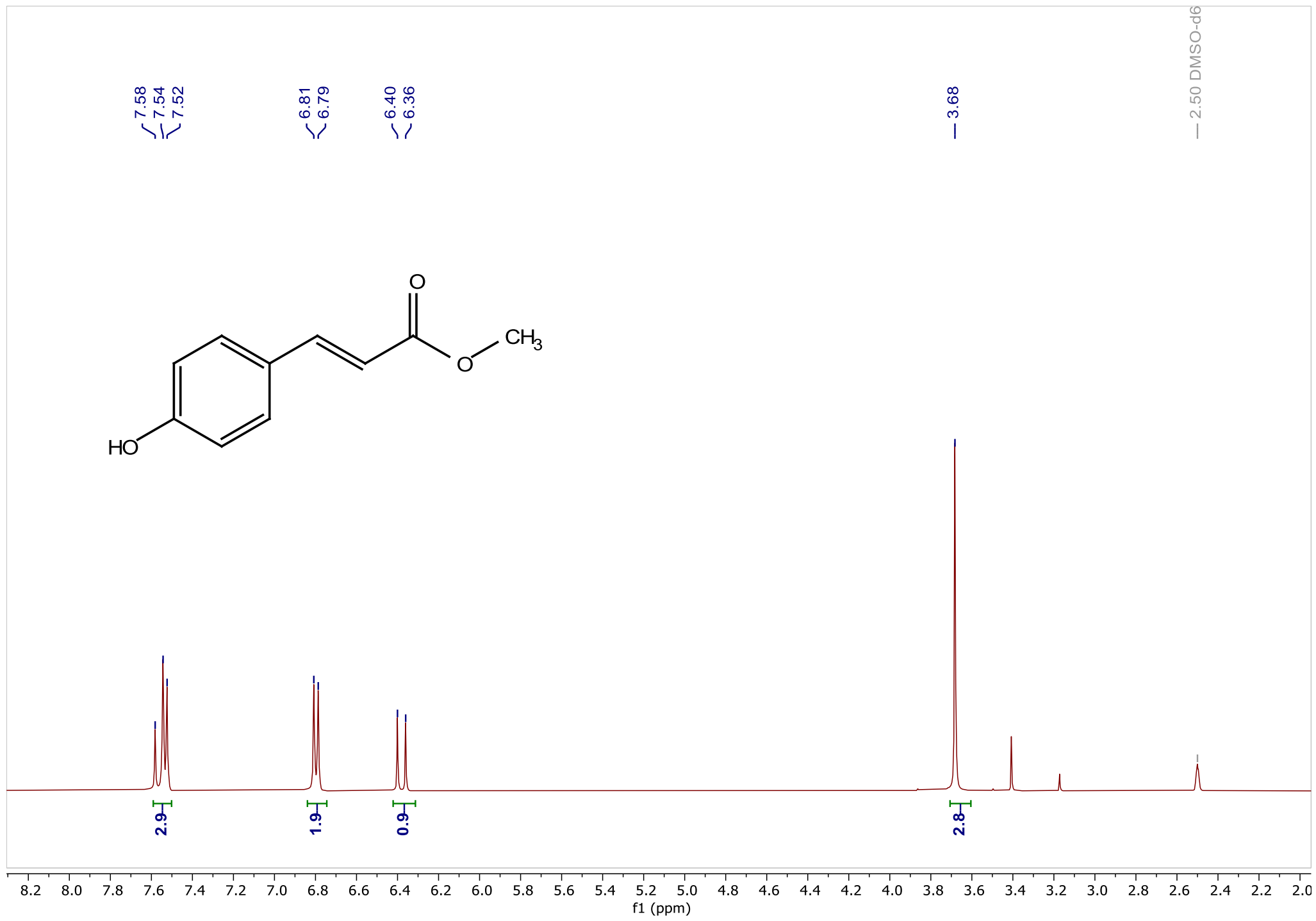

Figure S1. ^{1}H NMR (400 MHz, DMSO-d$_6$) spectrum of methyl (E)-3-(4-hydroxyphenyl)acrylate.

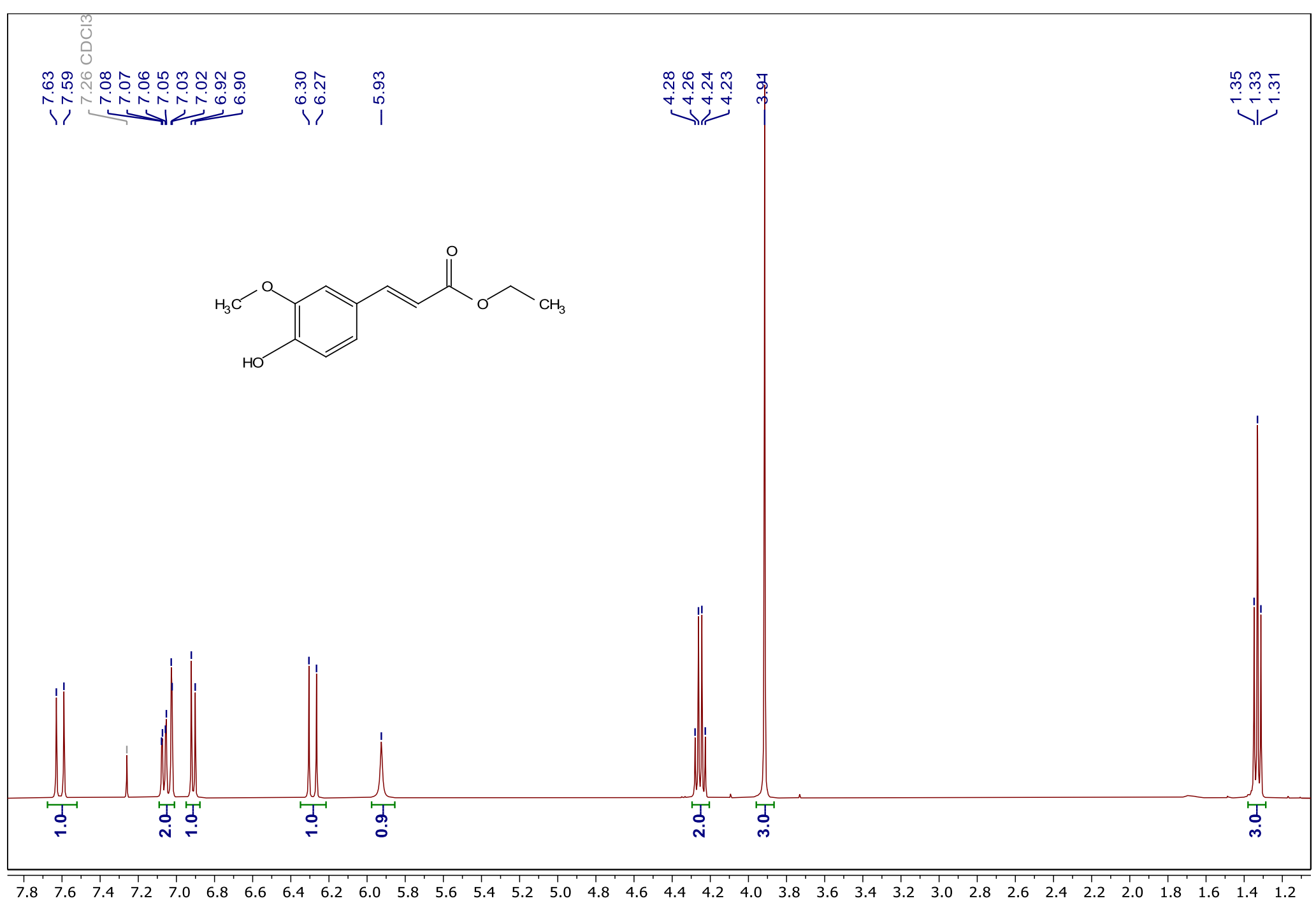

Figure S2. ^{1}H NMR (400 MHz, $CDCl_3$) spectrum of ethyl (E)-3-(4-hydroxy-3-methoxyphenyl) acrylate.

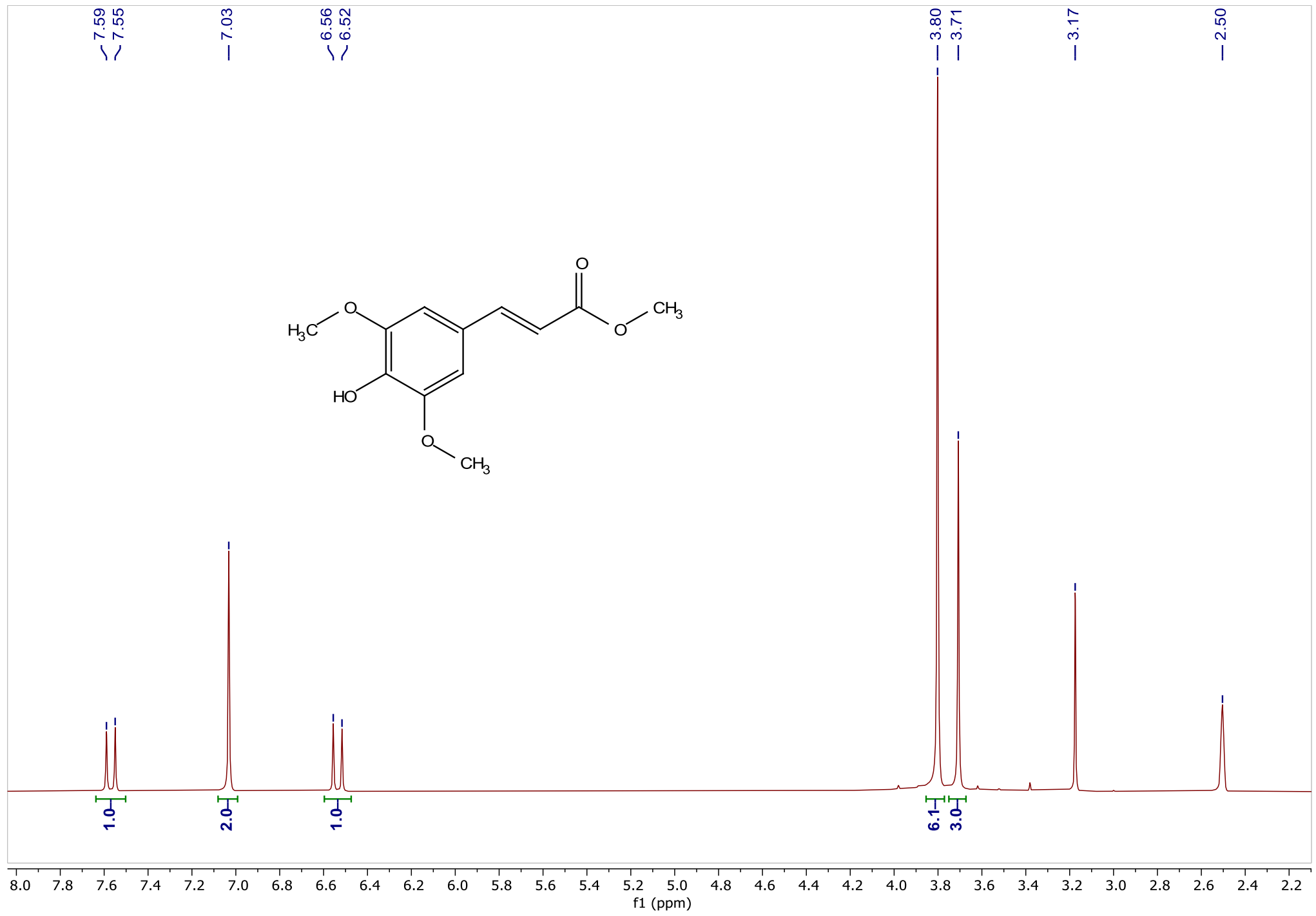

Figure S3. ^{1}H NMR (400 MHz, DMSO-d_6) spectrum of methyl (E)-3-(4-hydroxy-3,5-dimethoxyphenyl)acrylate.

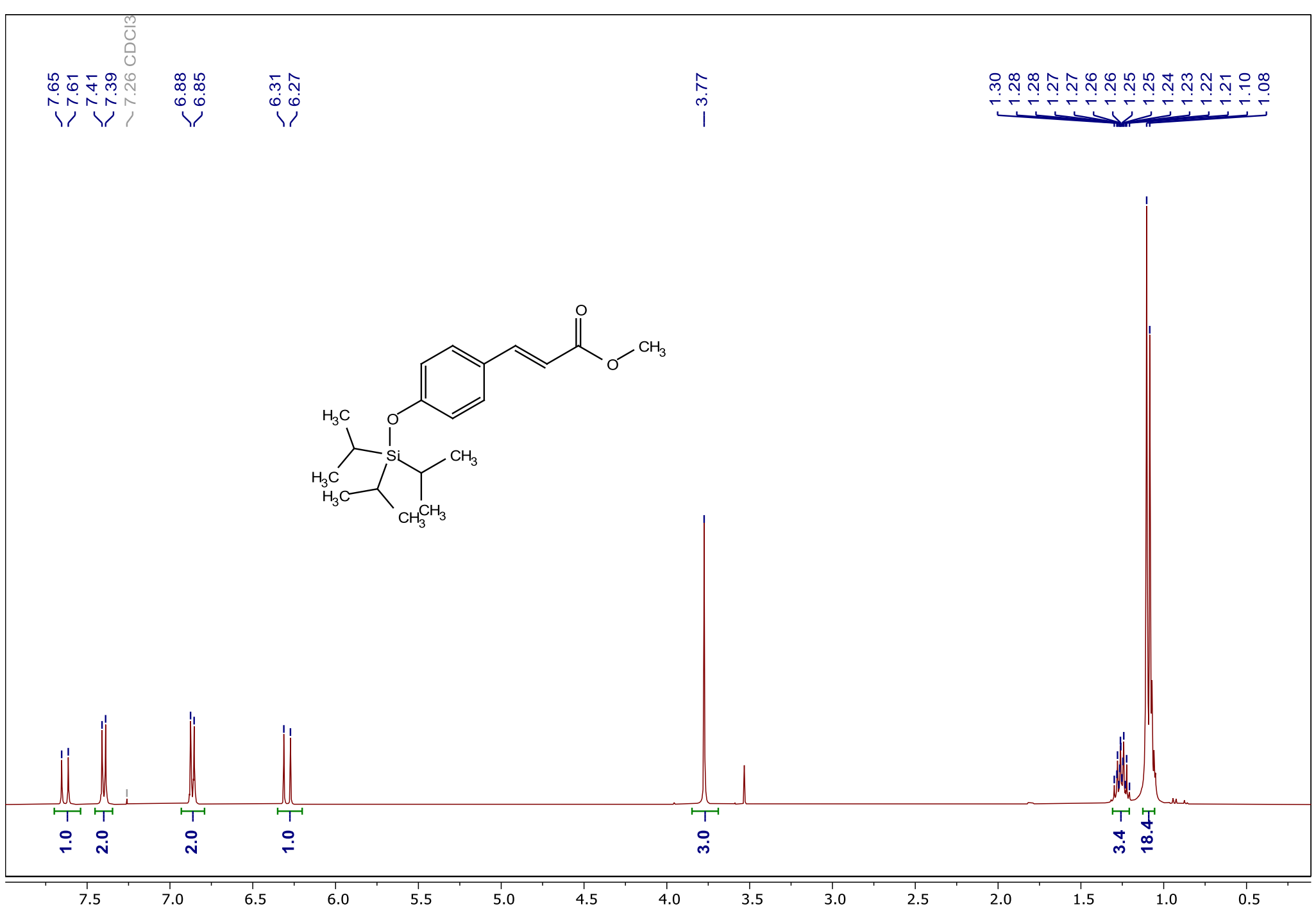

Figure S4. ^{1}H NMR (400 MHz, $CDCl_3$) spectrum of methyl (*E*)-3-(4-((triisopropylsilyl)oxy) phenyl)acrylate.

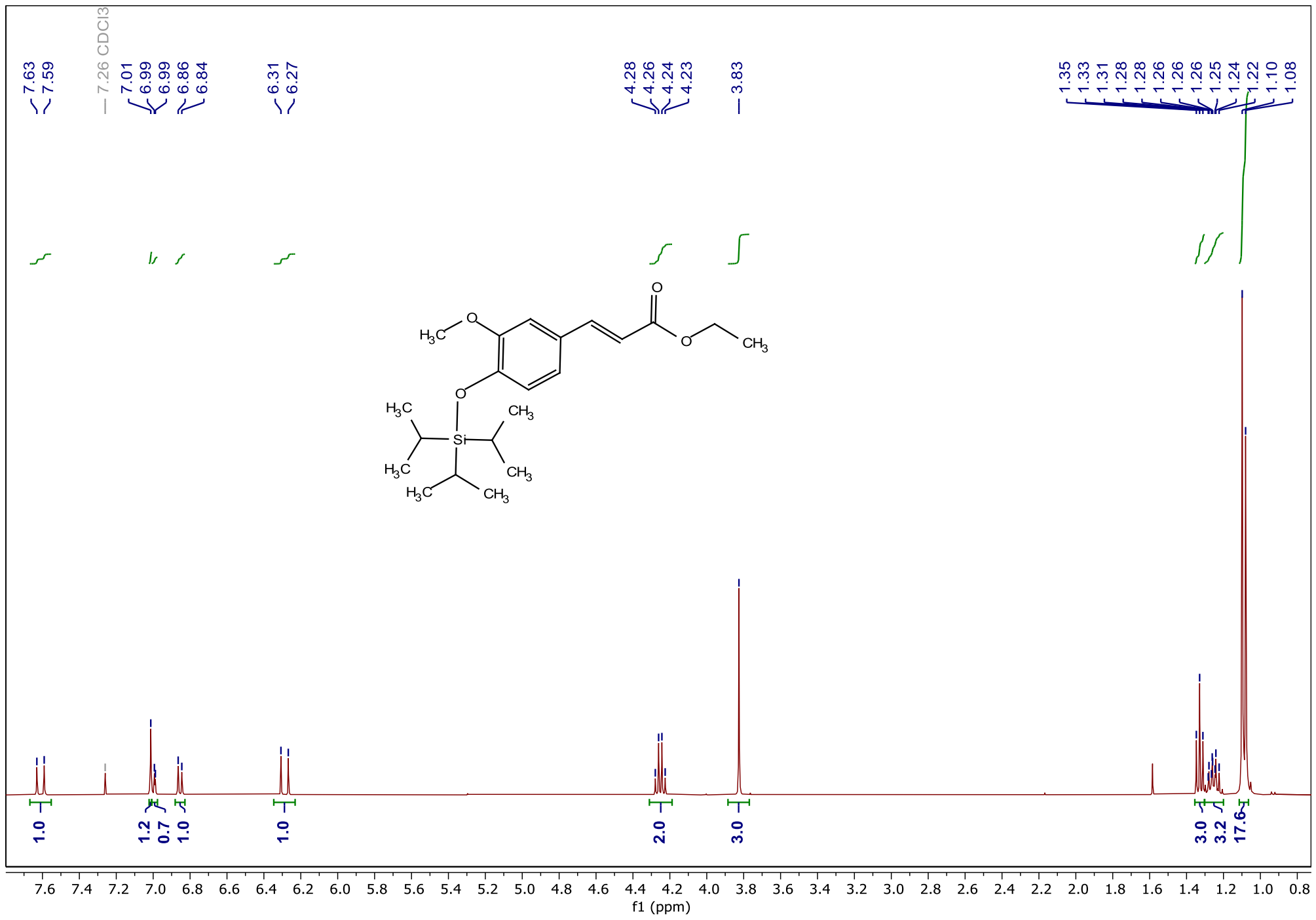

Figure S5. ^{1}H NMR (400 MHz, $CDCl_3$) spectrum of ethyl (*E*)-3-(3-methoxy-4-((triisopropylsilyl)oxy) phenyl) acrylate.

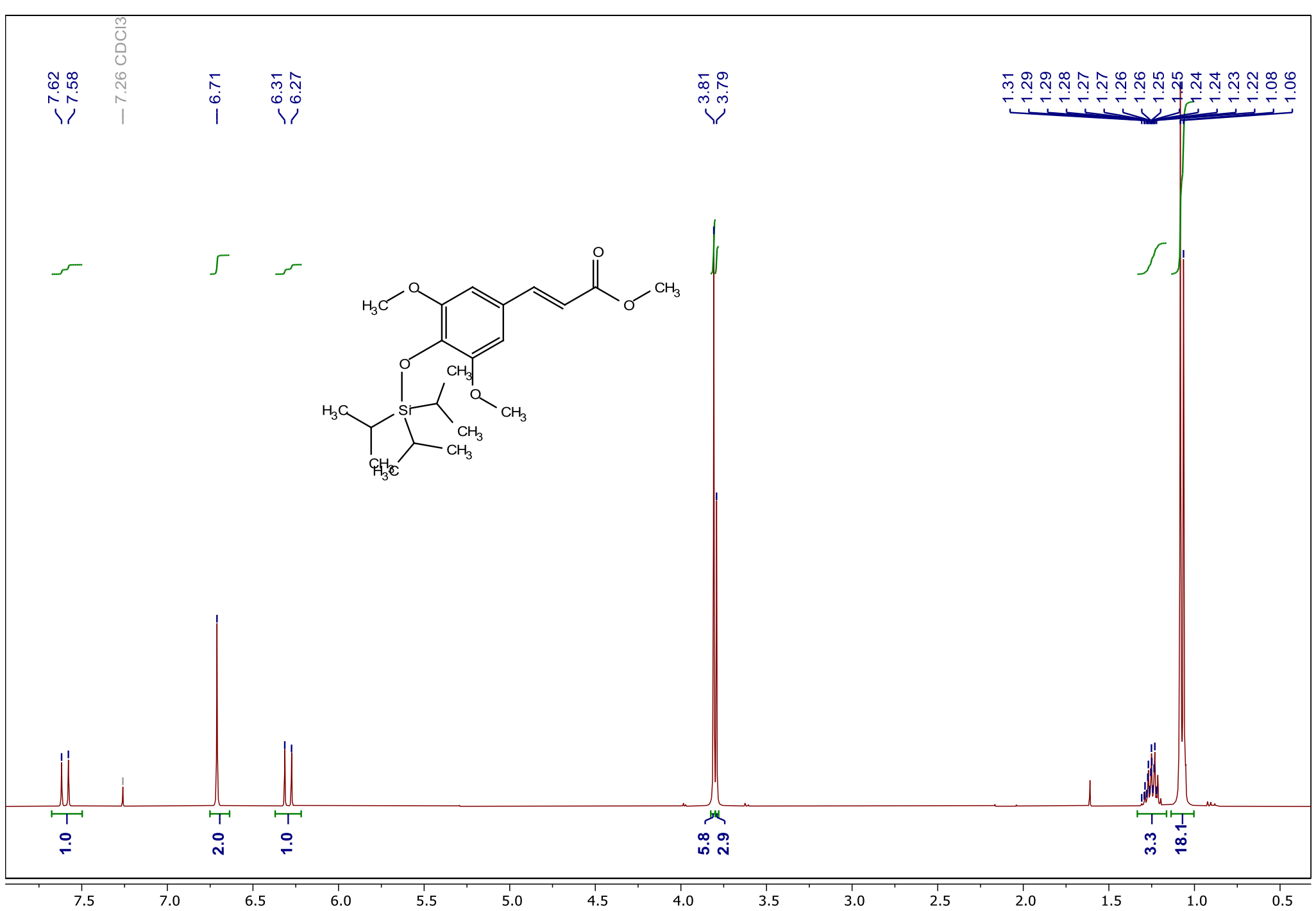

Figure S6. ^{1}H NMR (400 MHz, $CDCl_3$) spectrum of methyl (*E*)-3-(3,5-dimethoxy-4-((triisopropylsilyl)oxy)phenyl)acrylate.

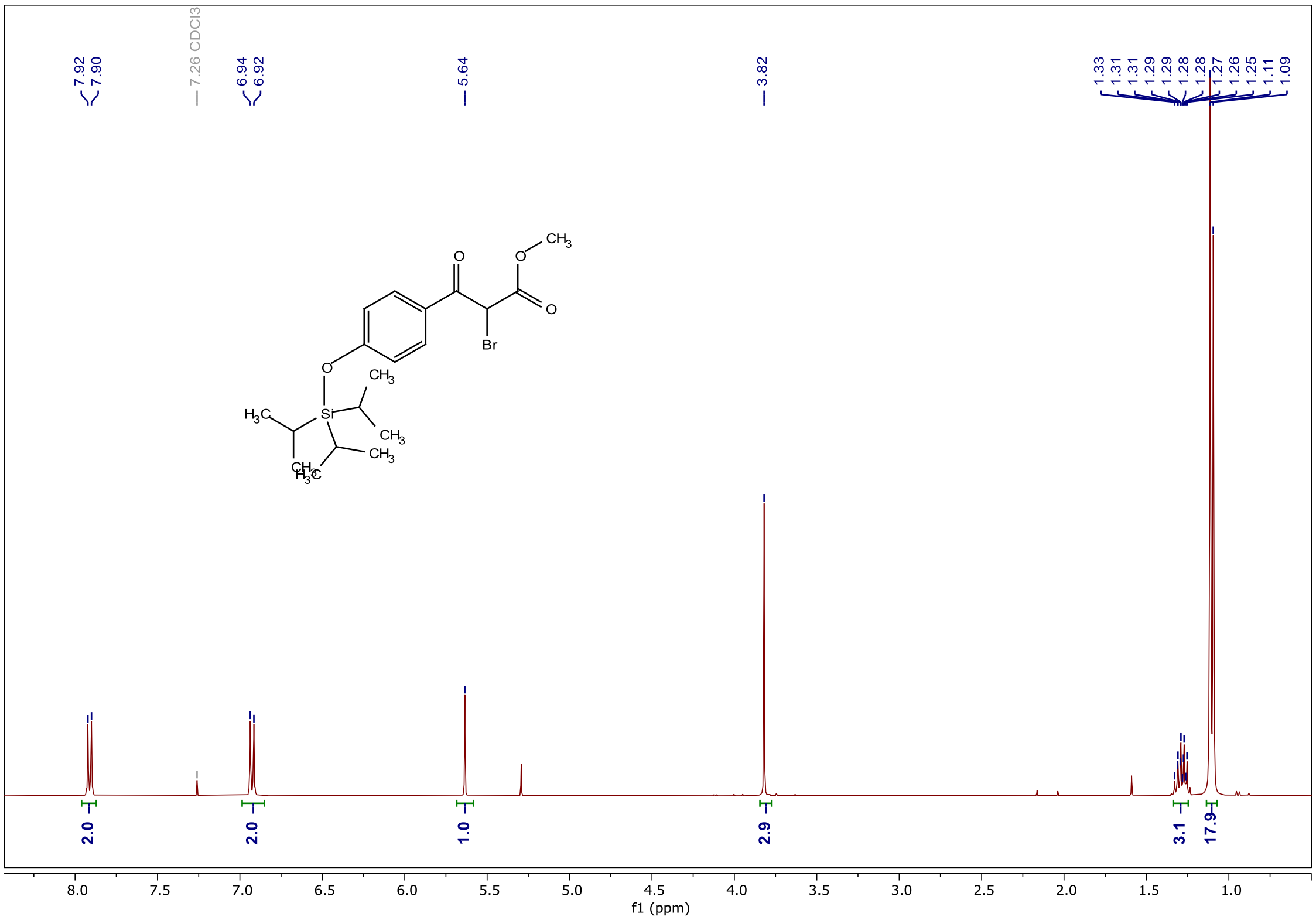

Figure S7. ^{1}H NMR (400 MHz, $CDCl_3$) spectrum of compound **2a.**

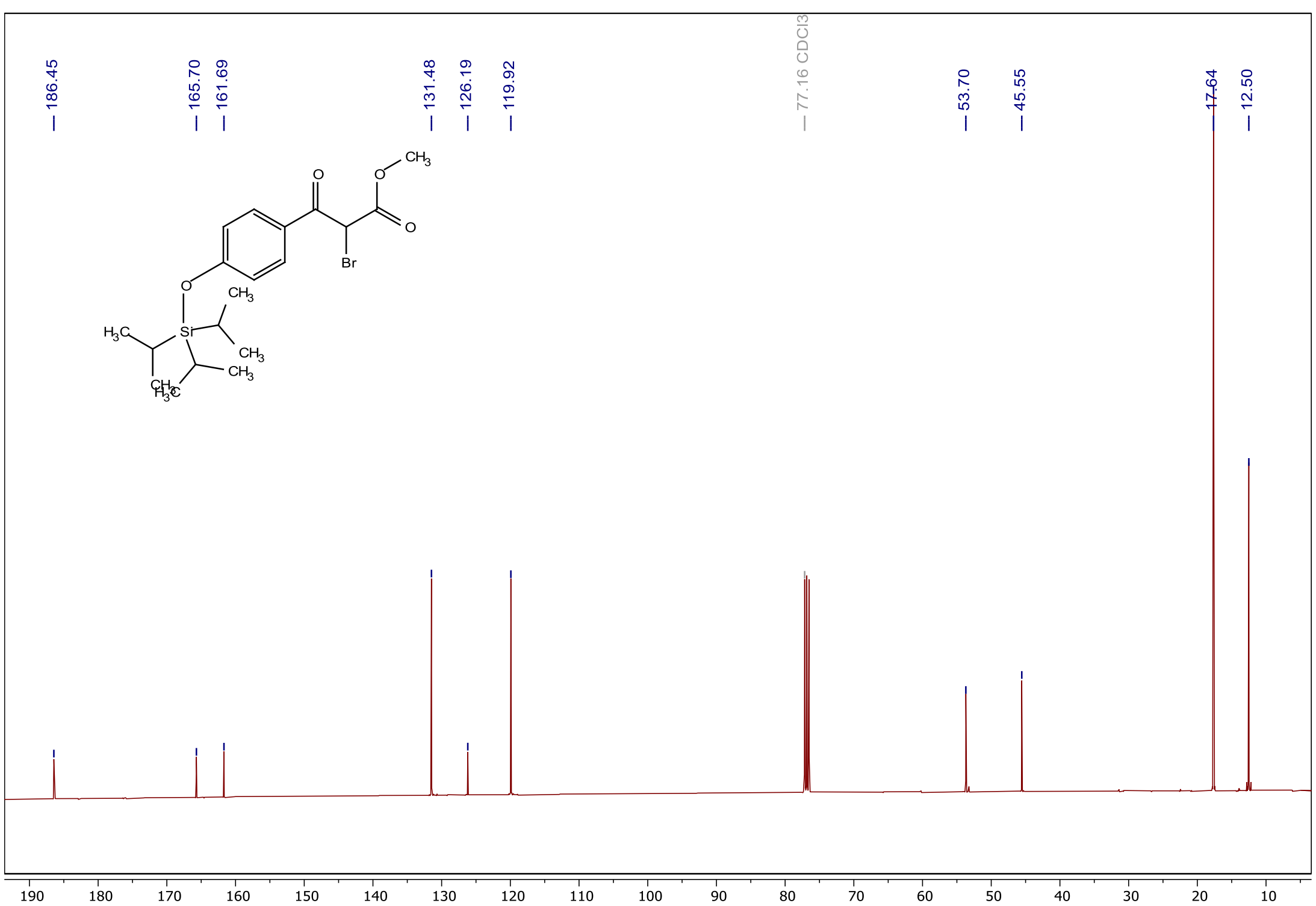

Figure S8. ^{13}C NMR (101 MHz, $CDCl_3$) spectrum of compound **2a.**

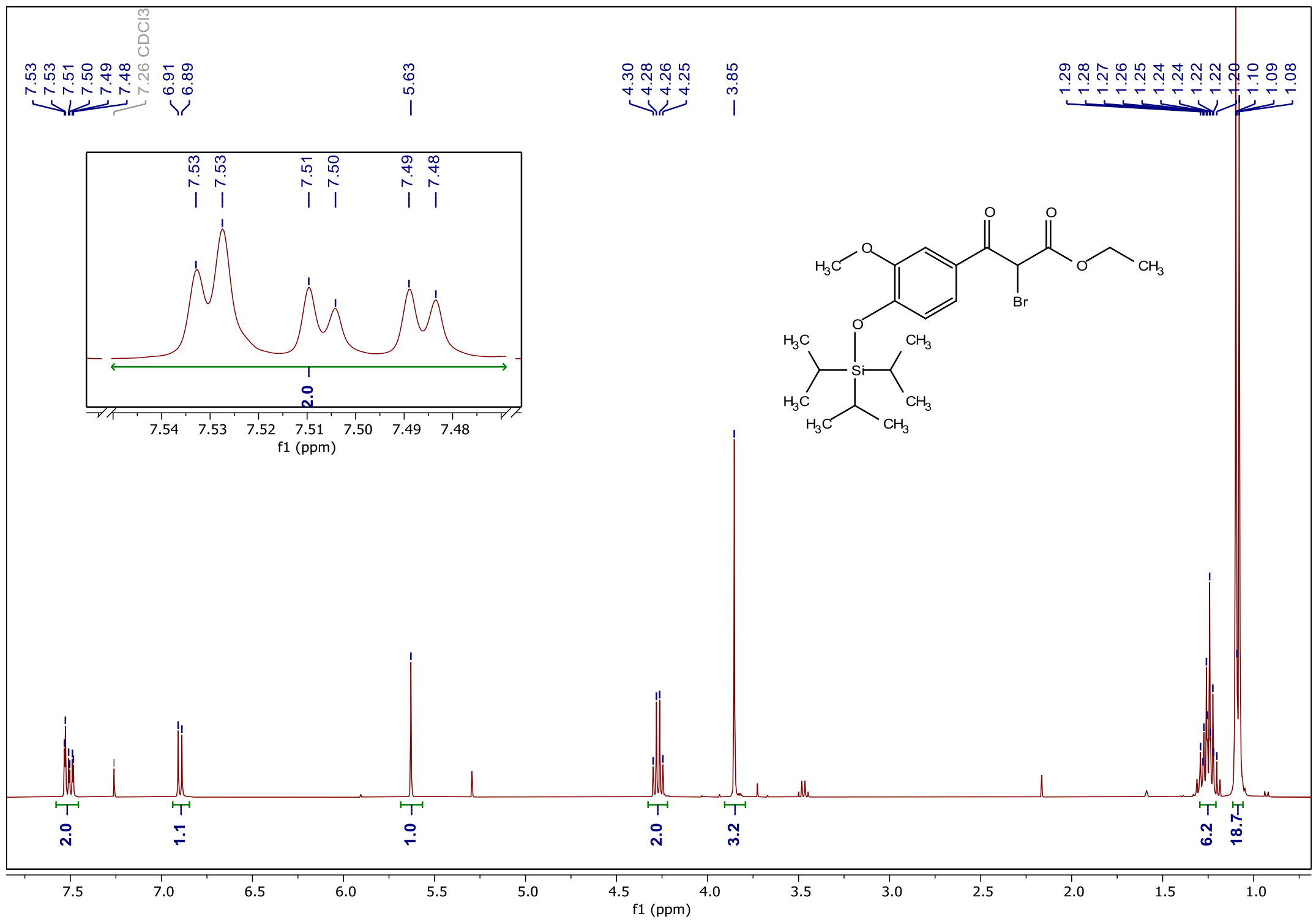

Figure S9. ^{1}H NMR (400 MHz, $CDCl_3$) spectrum of compound **2b.**

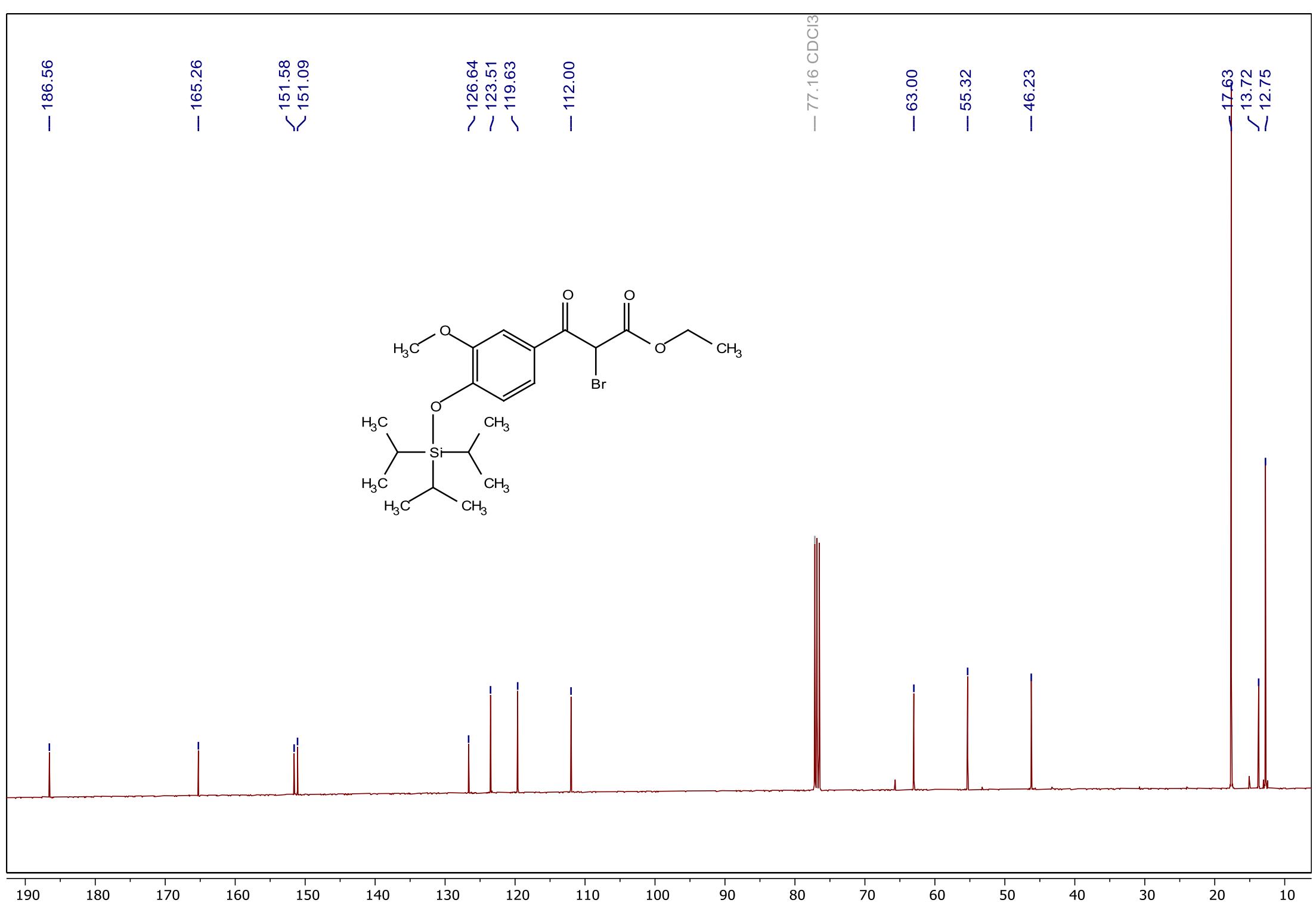

Figure S10. ^{13}C NMR (101 MHz, $CDCl_3$) spectrum of compound **2b.**

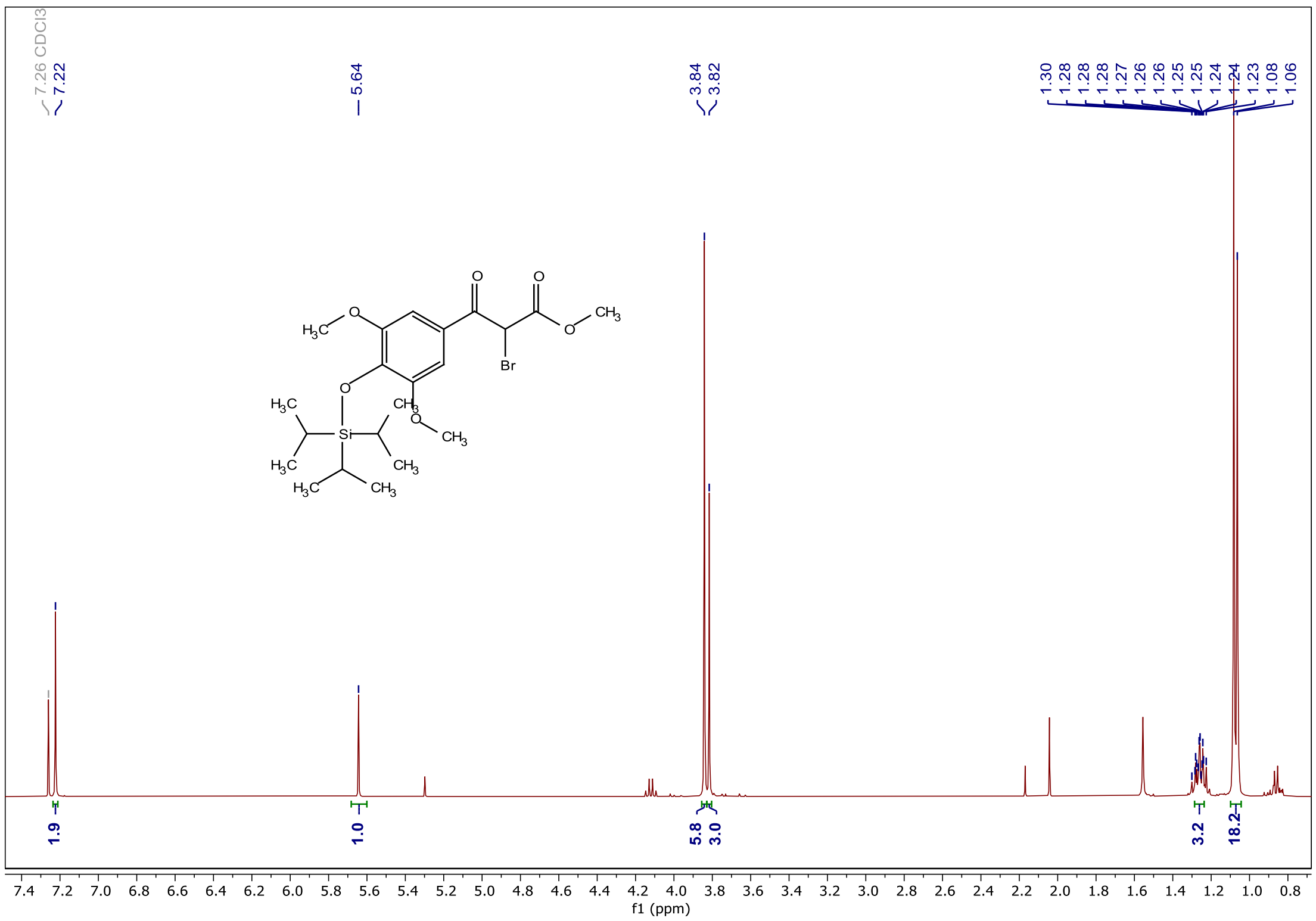

Figure S11. ^{1}H NMR (400 MHz, $CDCl_3$) spectrum of compound **2c.**

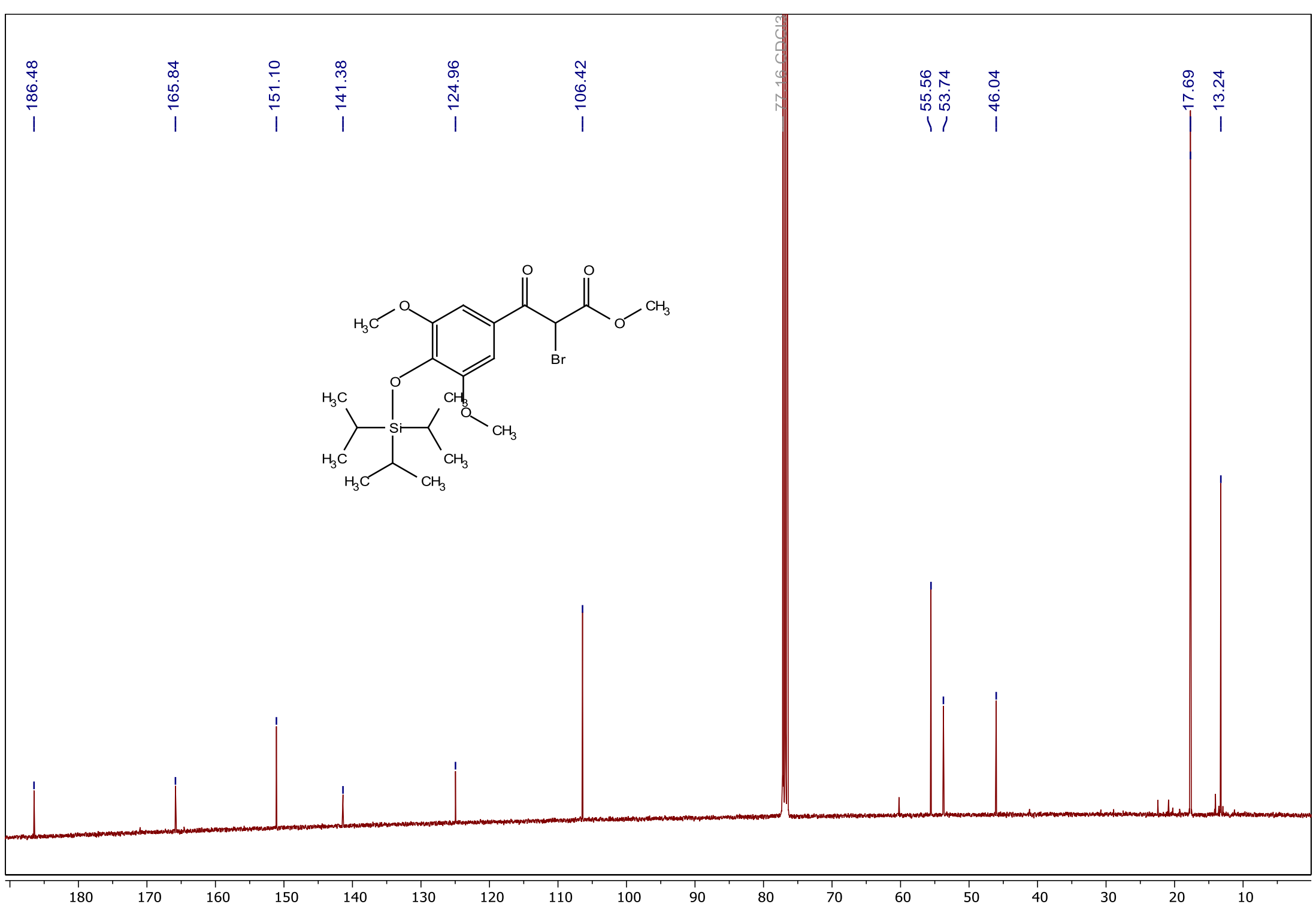

Figure S12. ^{13}C NMR (101 MHz, $CDCl_3$) spectrum of compound **2c.**

NMR Spectra of Dimers 3a-k:

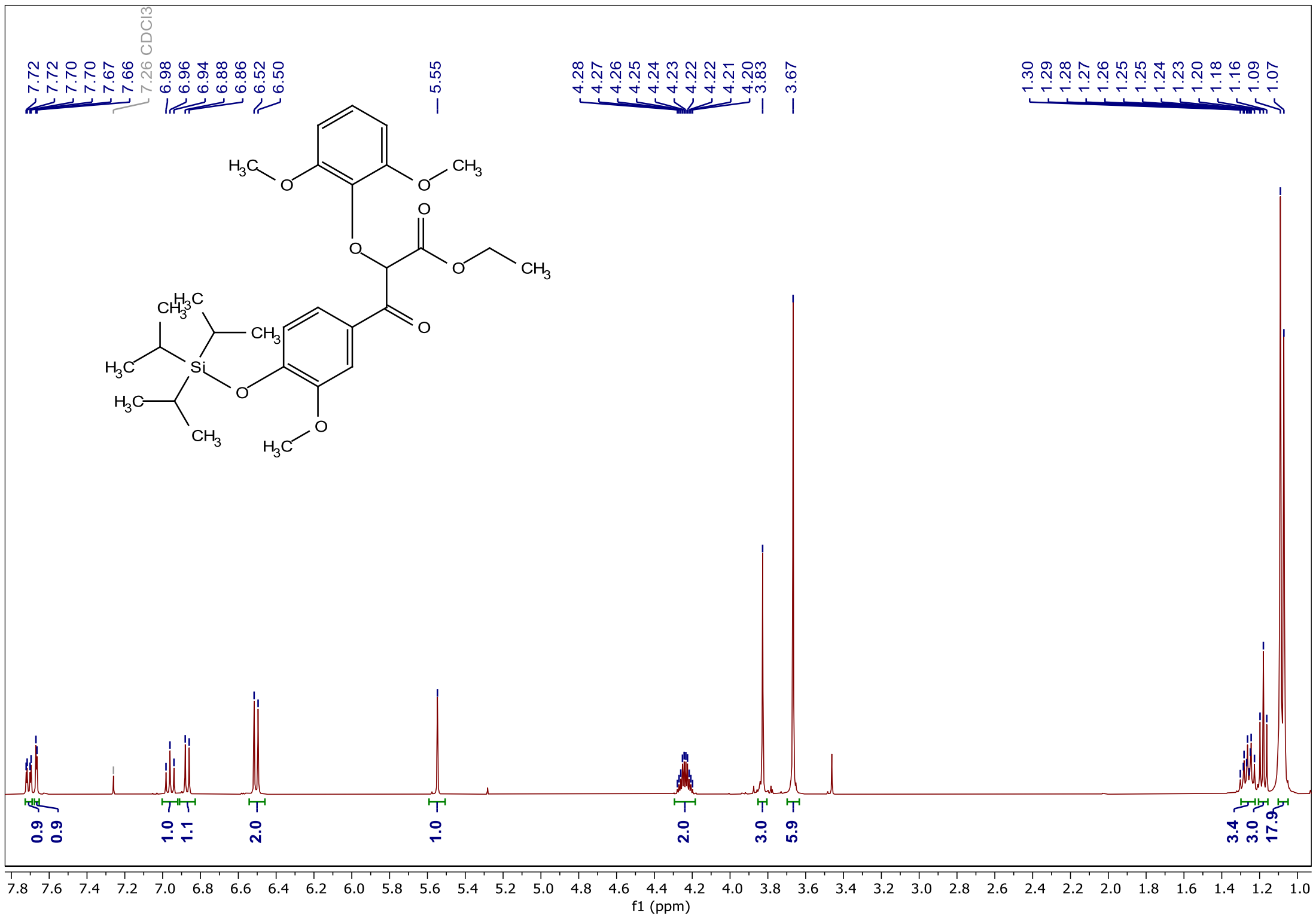

Figure S13. ^{1}H NMR (400 MHz, $CDCl_3$) spectrum of compound **3a-TIPS.**

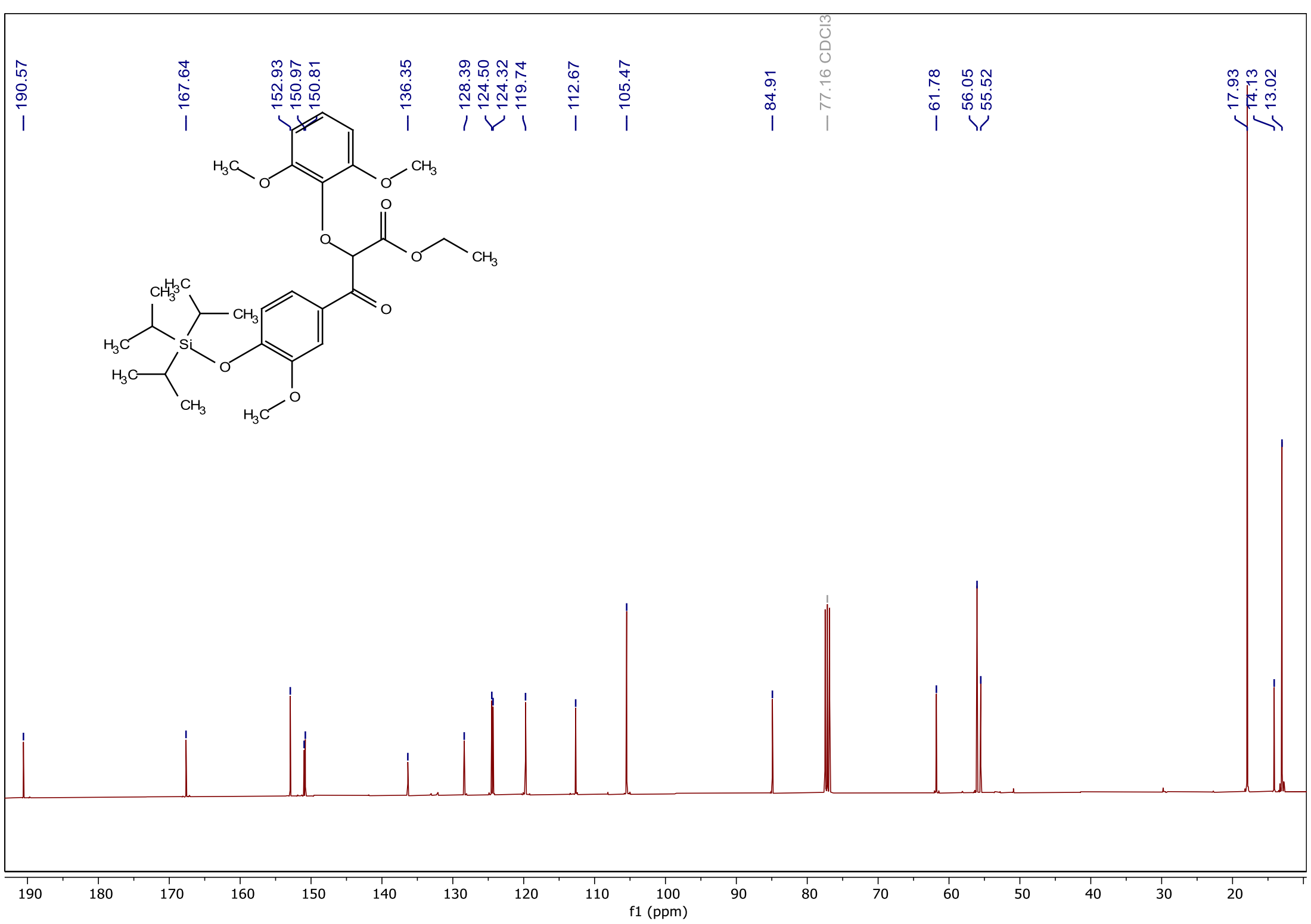

Figure S14. ^{13}C NMR (101 MHz, $CDCl_3$) spectrum of compound **3a-TIPS.**

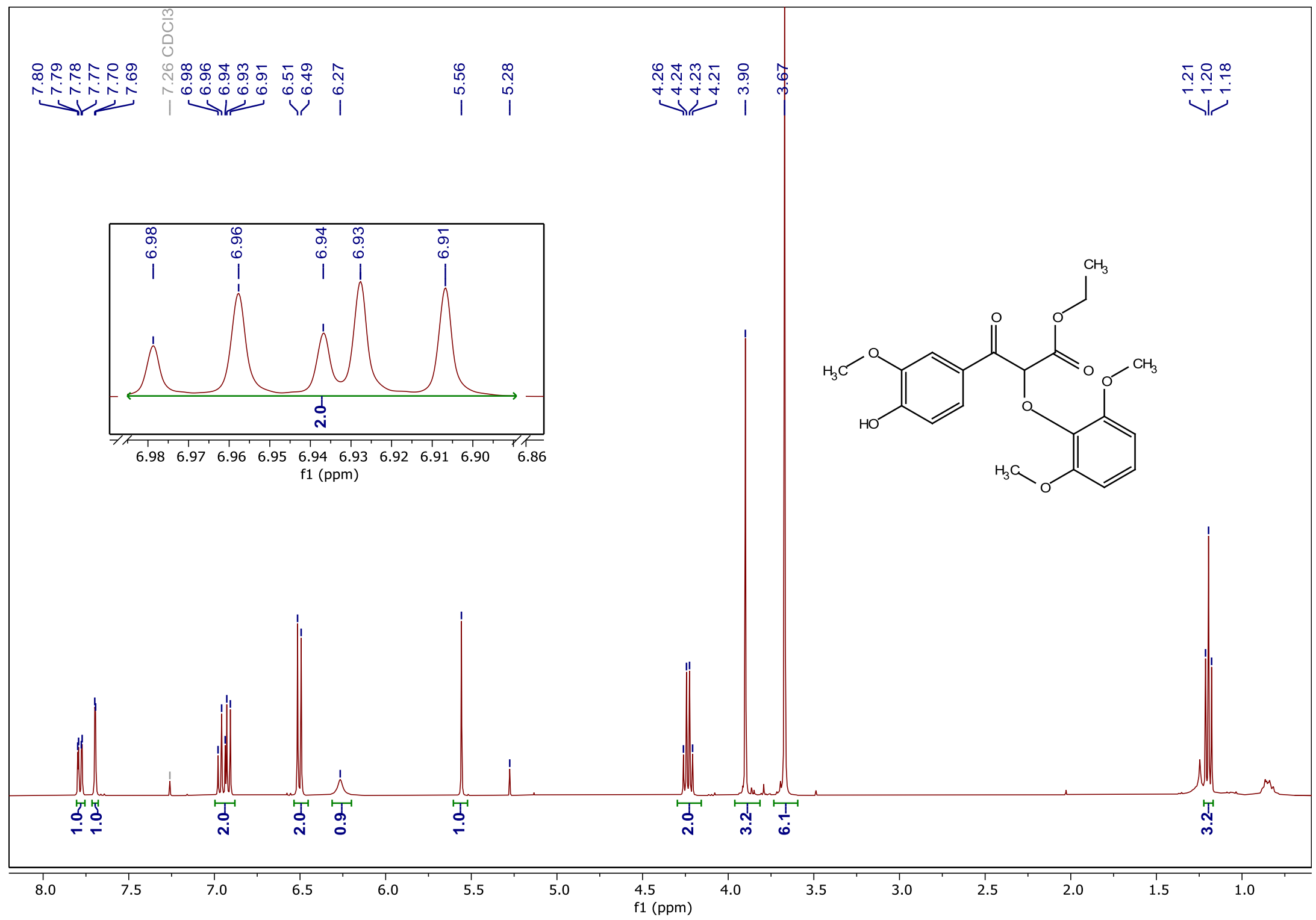

Figure S15. ^{1}H NMR (400 MHz, $CDCl_3$) spectrum of compound **3a.**

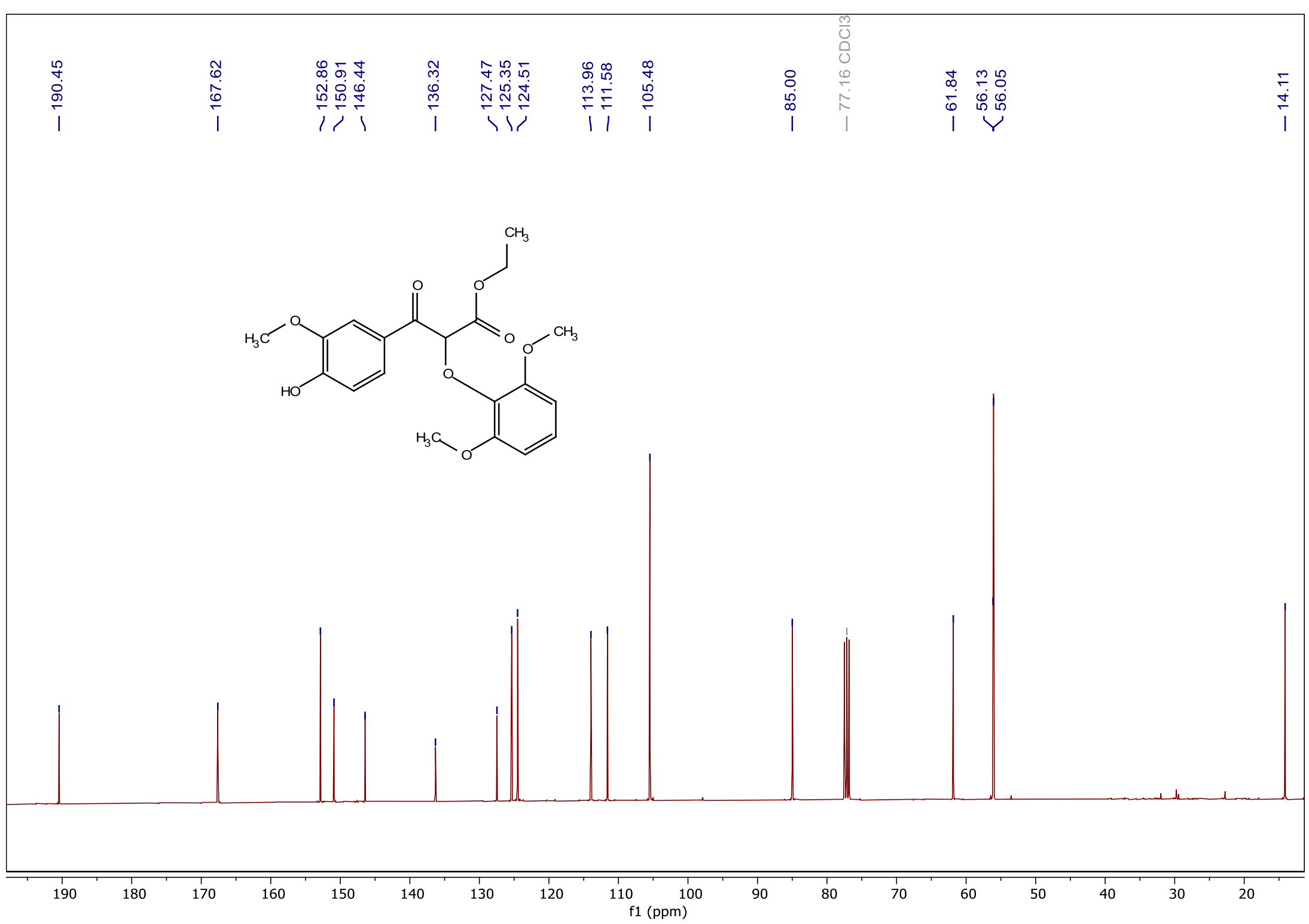

Figure S16. ^{13}C NMR (101 MHz, $CDCl_3$) spectrum of compound **3a.**

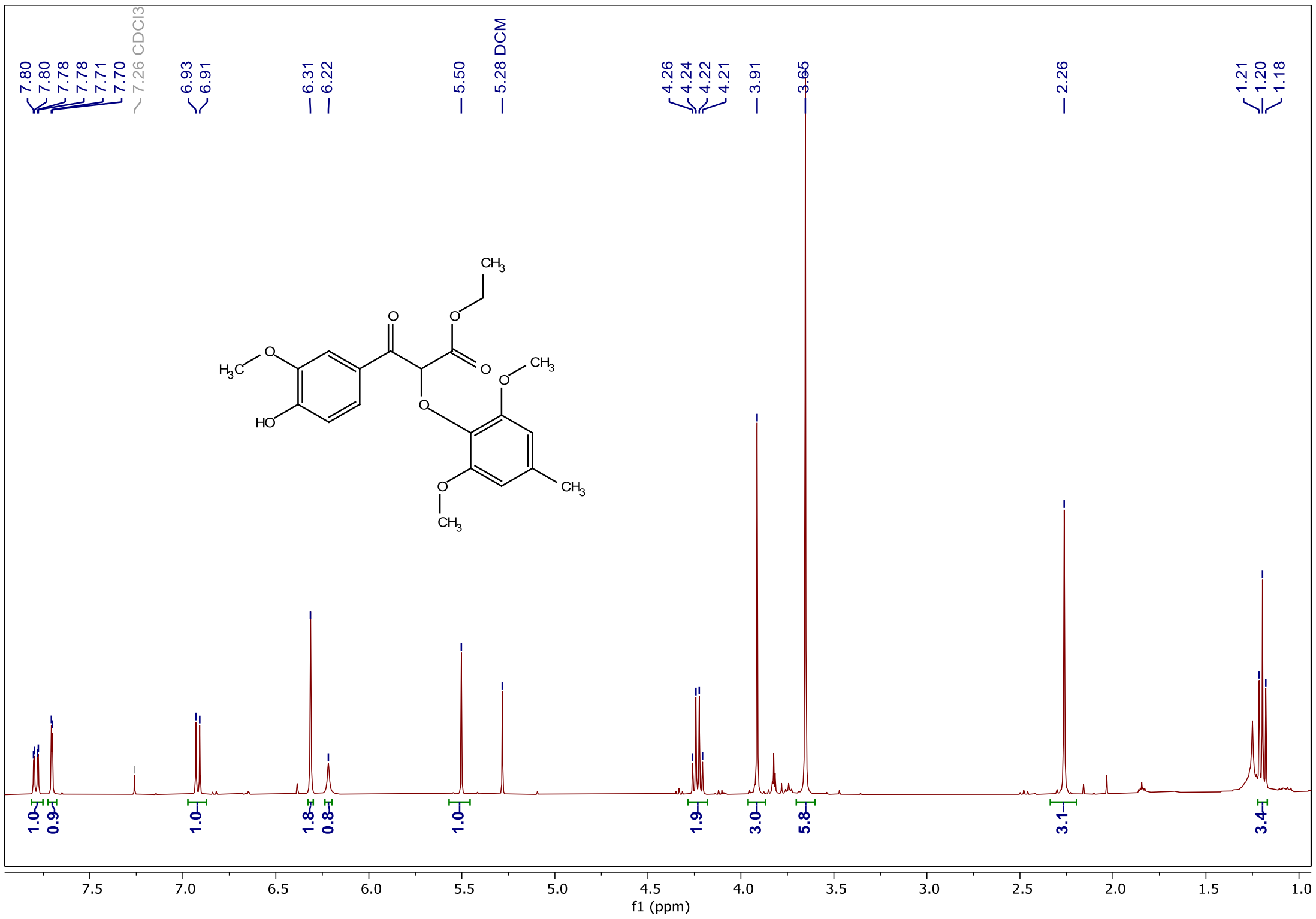

Figure S17. ^{1}H NMR (400 MHz, $CDCl_3$) spectrum of compound **3b.**

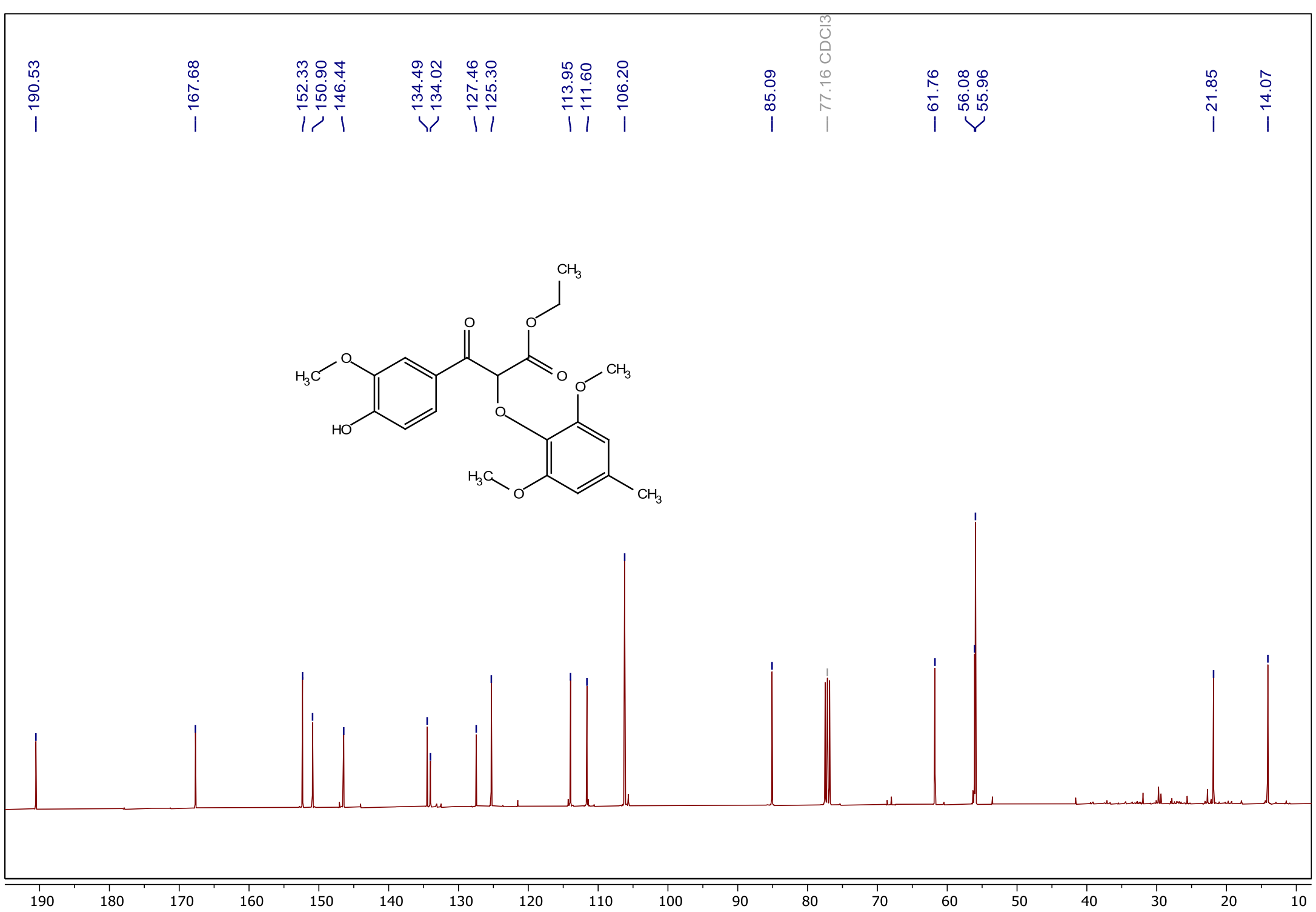

Figure S18. ^{13}C NMR (101 MHz, $CDCl_3$) spectrum of compound **3b.**

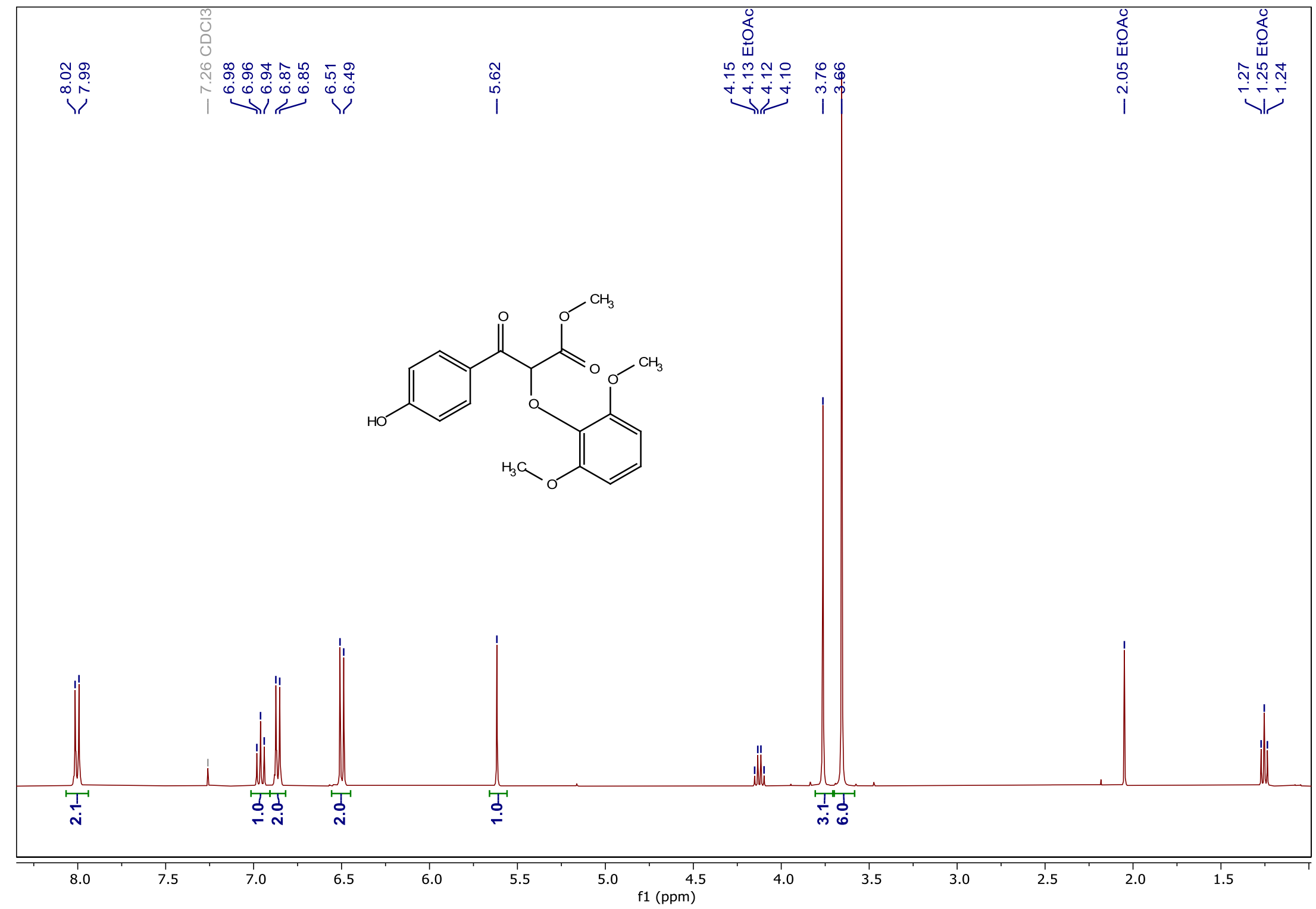

Figure S19. ^{1}H NMR (400 MHz, $CDCl_3$) spectrum of compound **3c.**

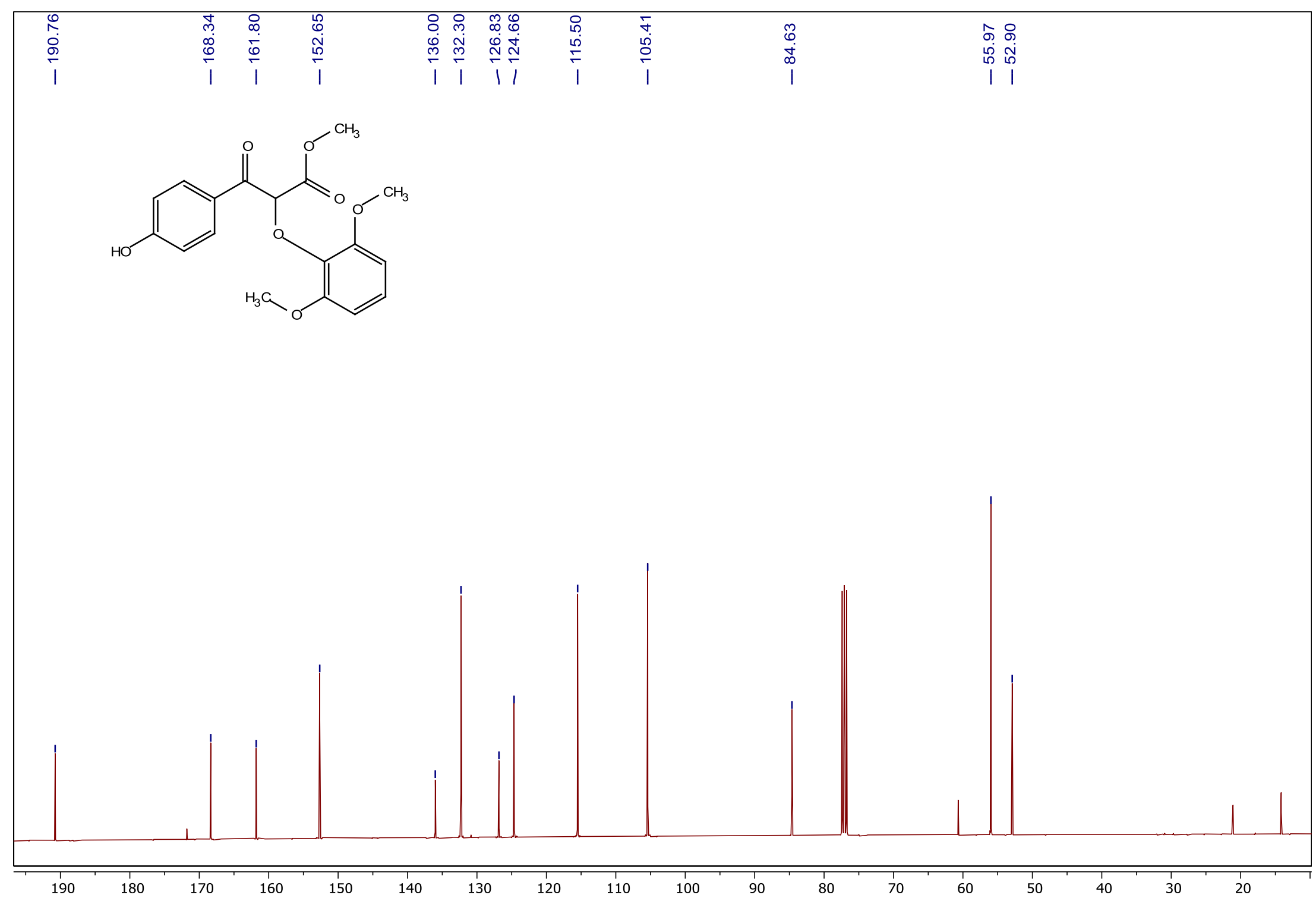

Figure S20. ^{13}C NMR (101 MHz, $CDCl_3$) spectrum of compound **3c.**

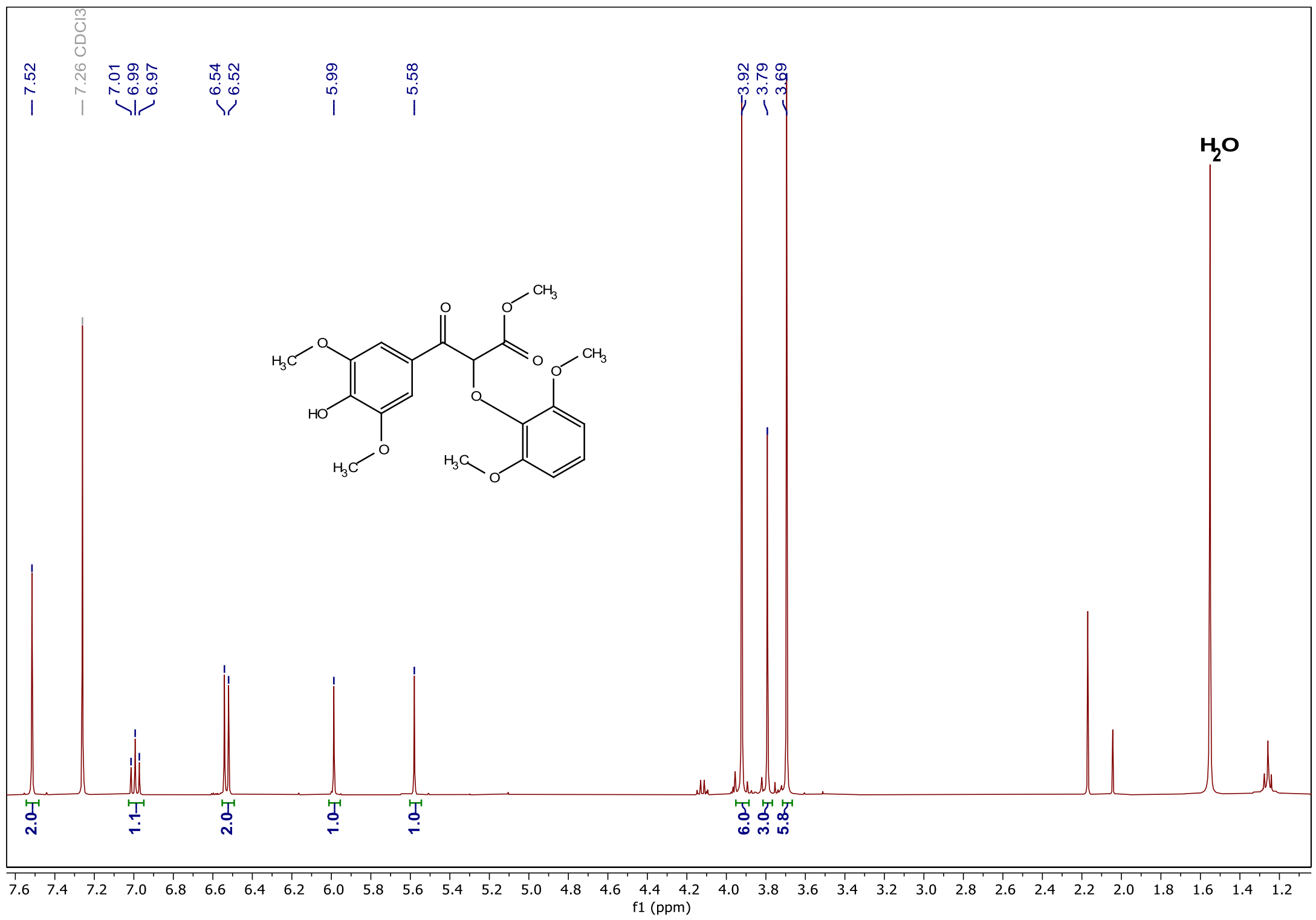

Figure S21. ^{1}H NMR (400 MHz, $CDCl_3$) spectrum of compound **3d.**

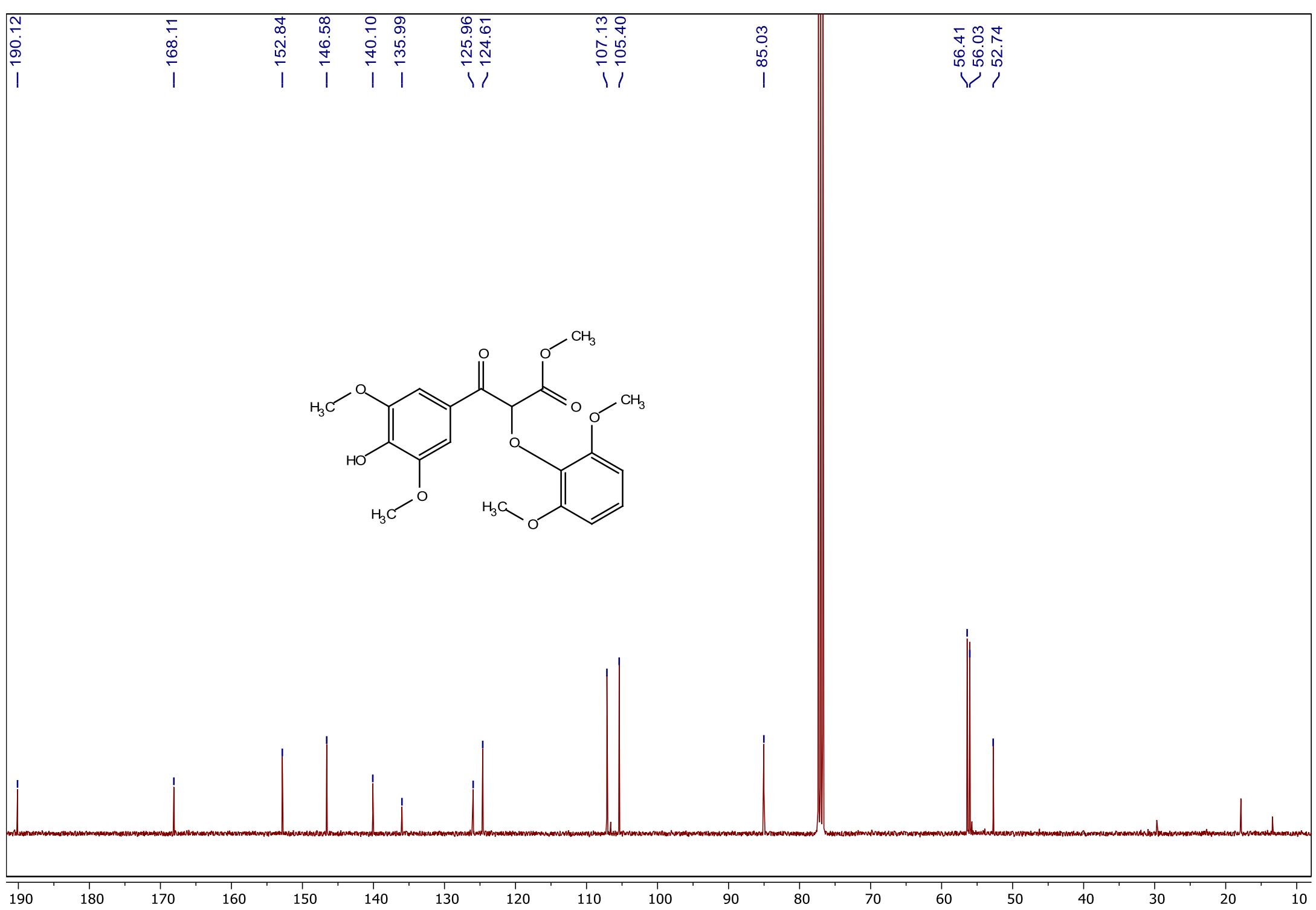

Figure S22. ^{13}C NMR (101 MHz, $CDCl_3$) spectrum of compound **3d.**

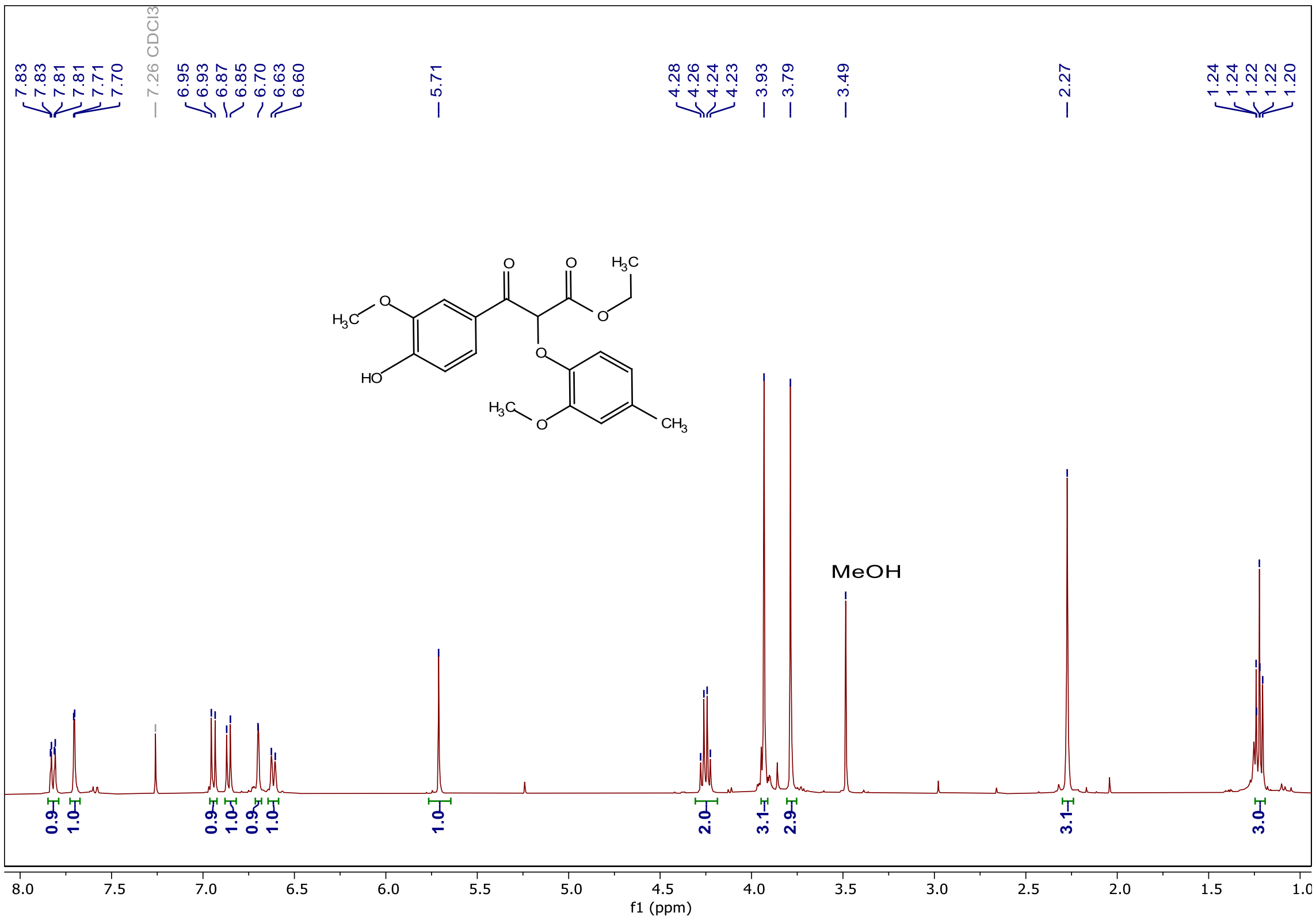

Figure S23. ^{1}H NMR (400 MHz, $CDCl_3$) spectrum of compound **3e.**

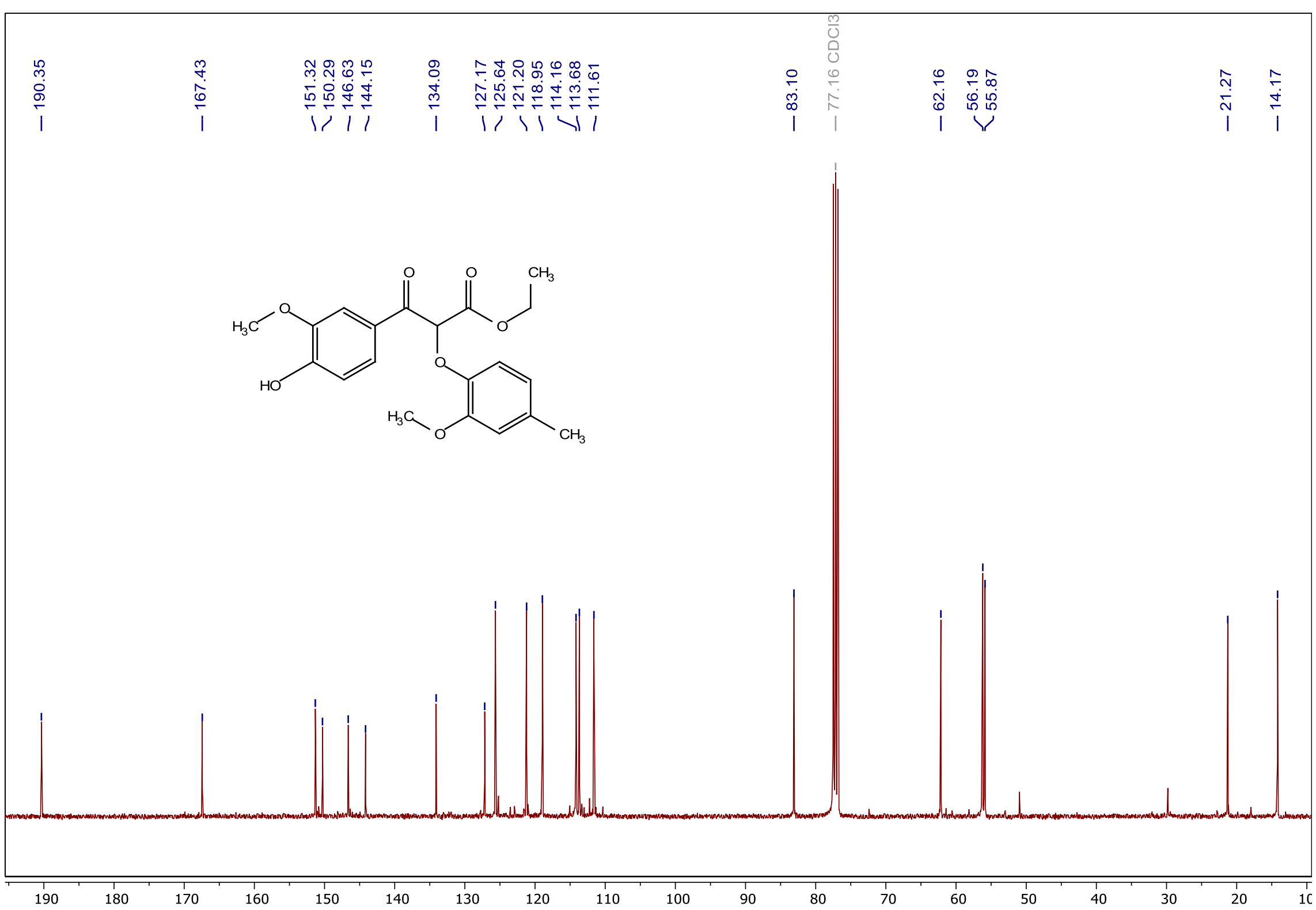

Figure S24. ^{13}C NMR (101 MHz, $CDCl_3$) spectrum of compound **3e.**

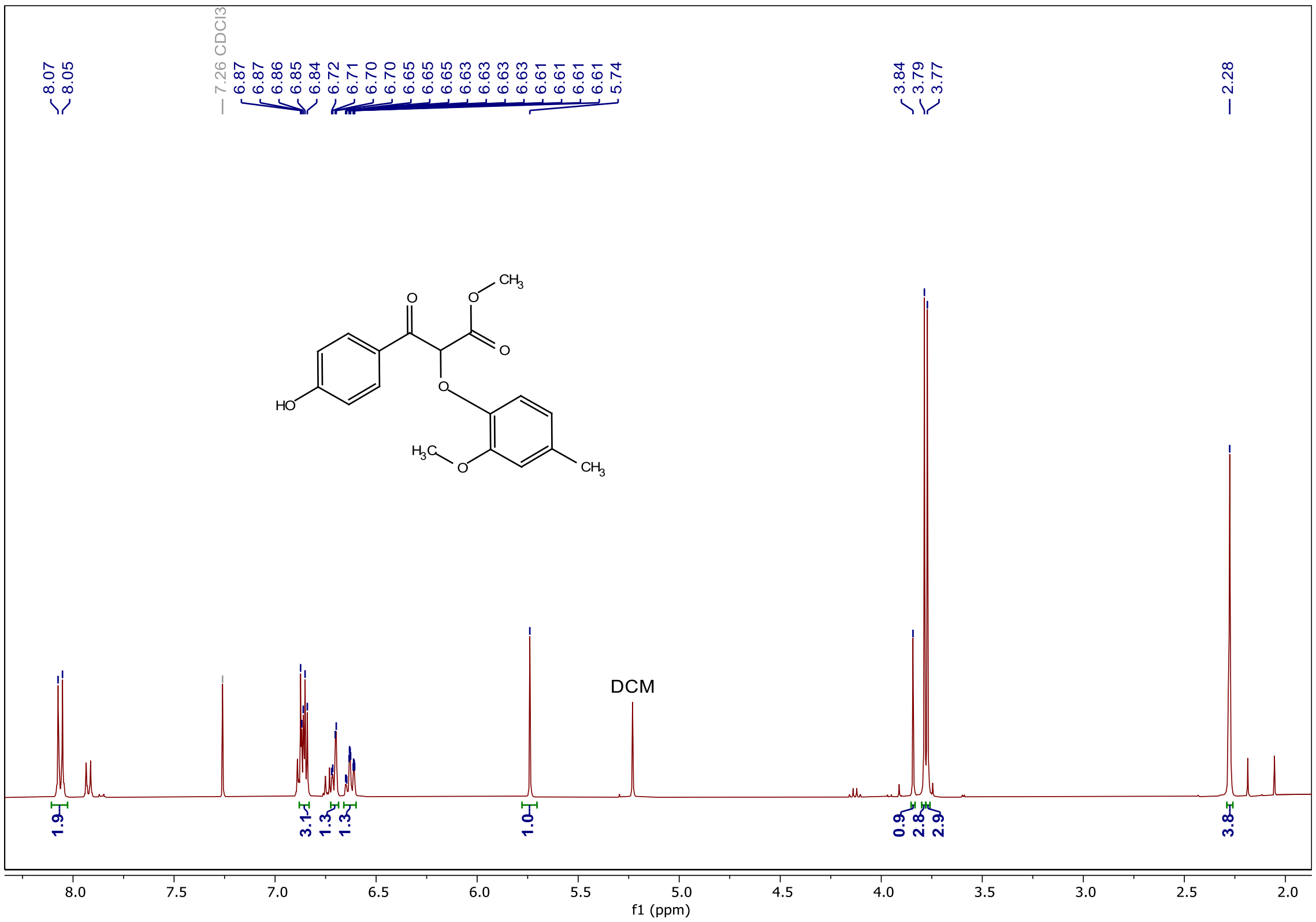

Figure S25. ^{1}H NMR (400 MHz, $CDCl_3$) spectrum of compound **3f.**

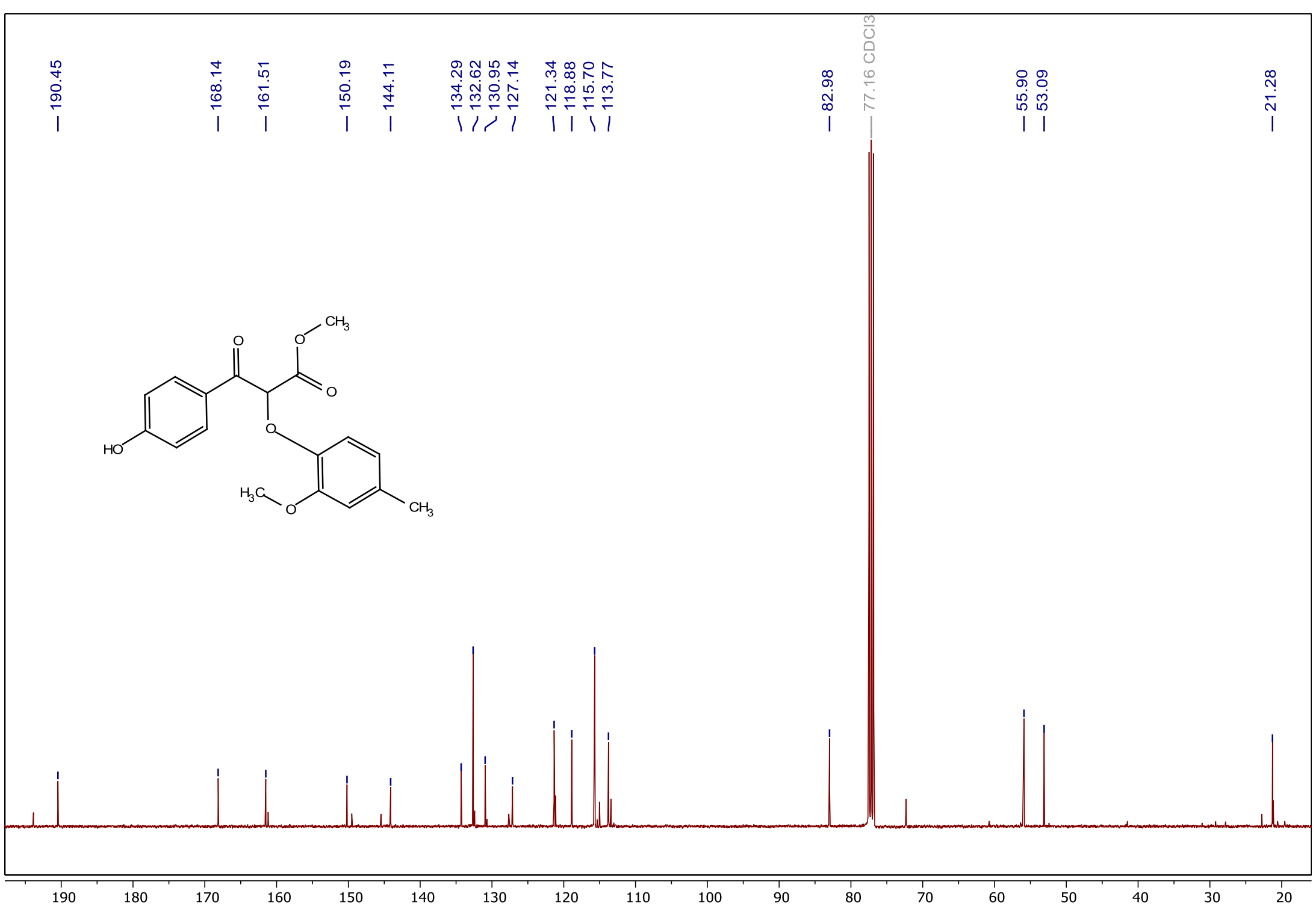

Figure S26. ^{13}C NMR (101 MHz, $CDCl_3$) spectrum of compound **3f.**

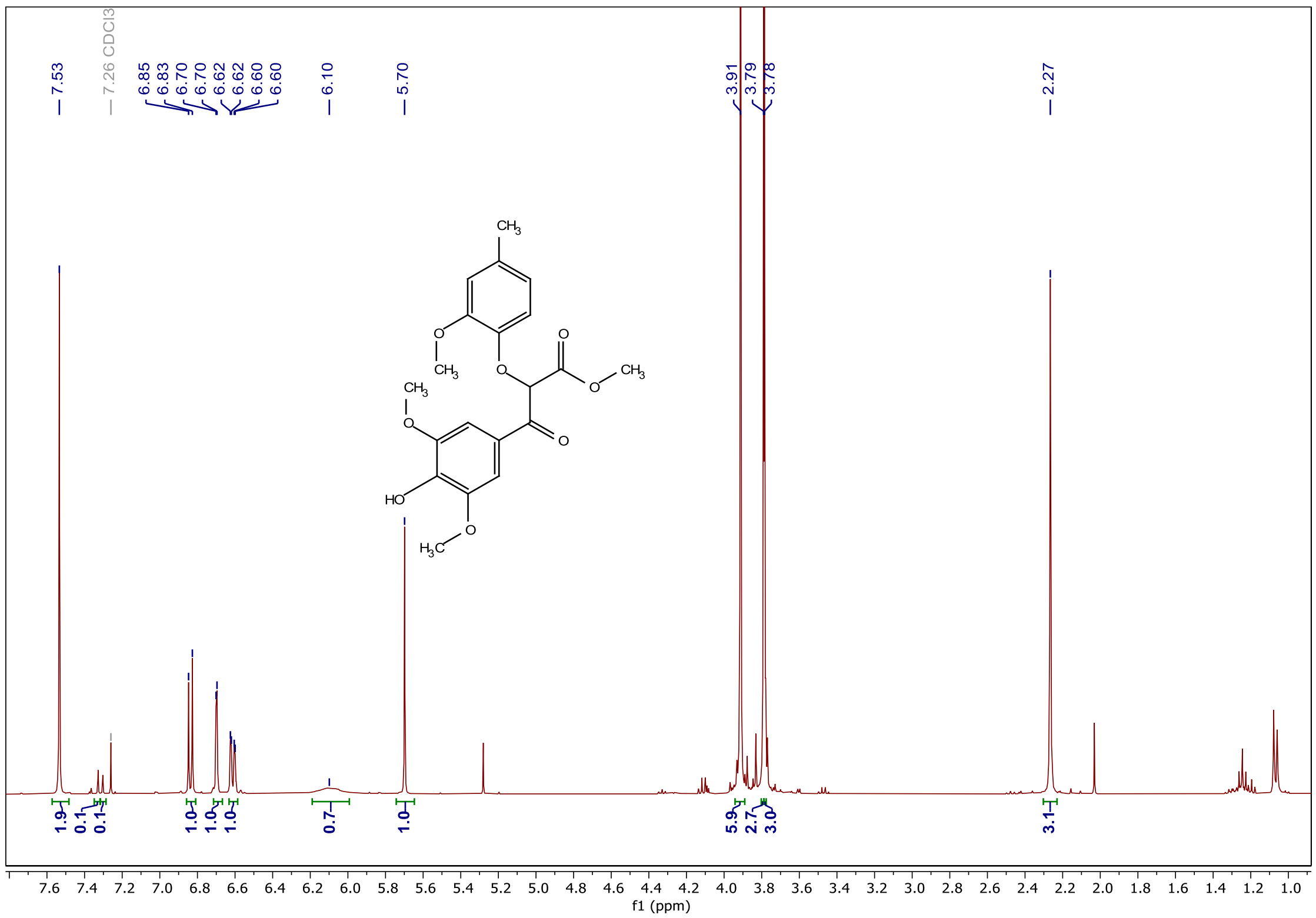

Figure S27. ^{1}H NMR (400 MHz, $CDCl_3$) spectrum of compound **3g.**

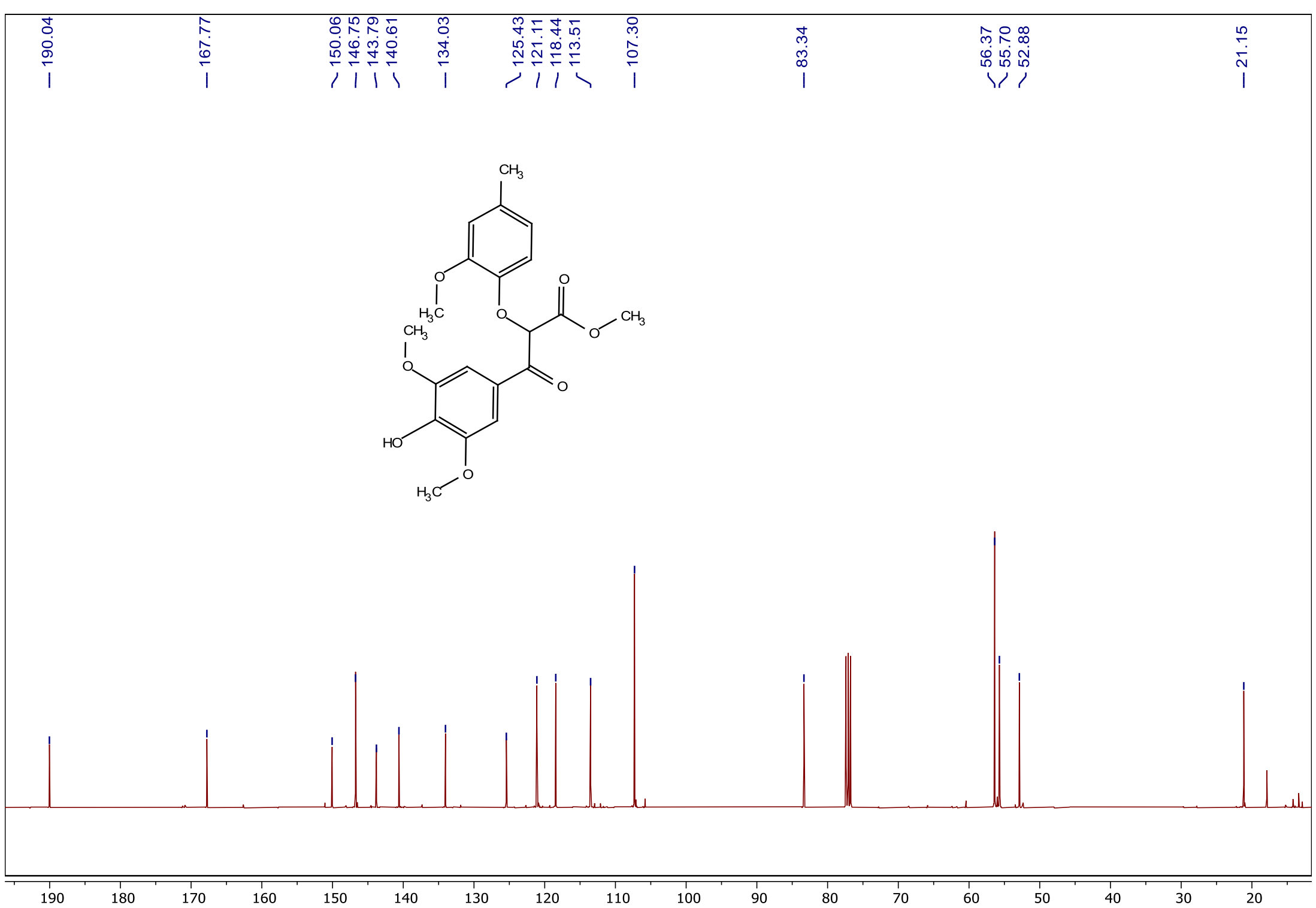

Figure S28. ^{13}C NMR (101 MHz, $CDCl_3$) spectrum of compound **3g.**

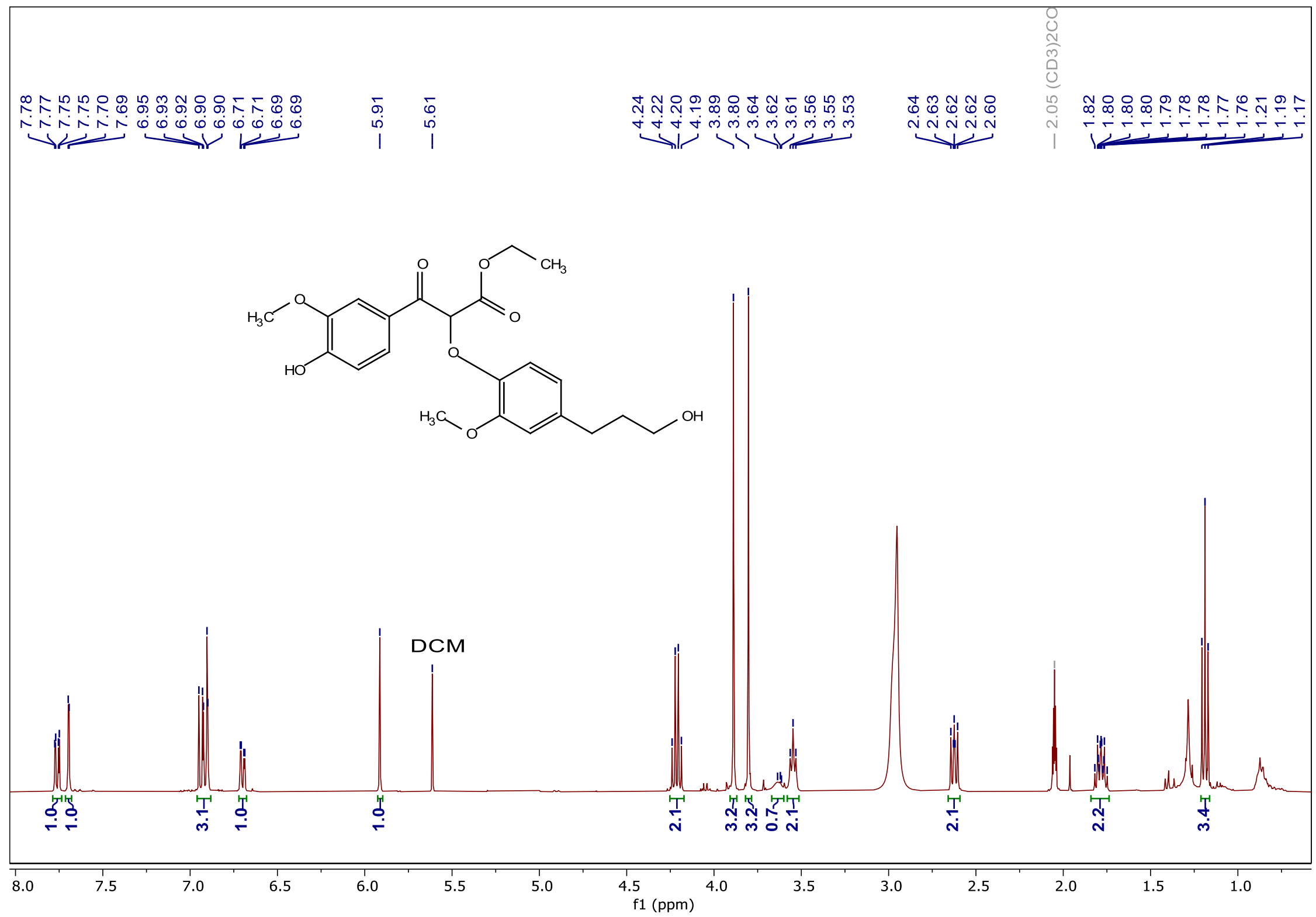

Figure S29. ^{1}H NMR (400 MHz, acetone-d_6) spectrum of compound **3h.**

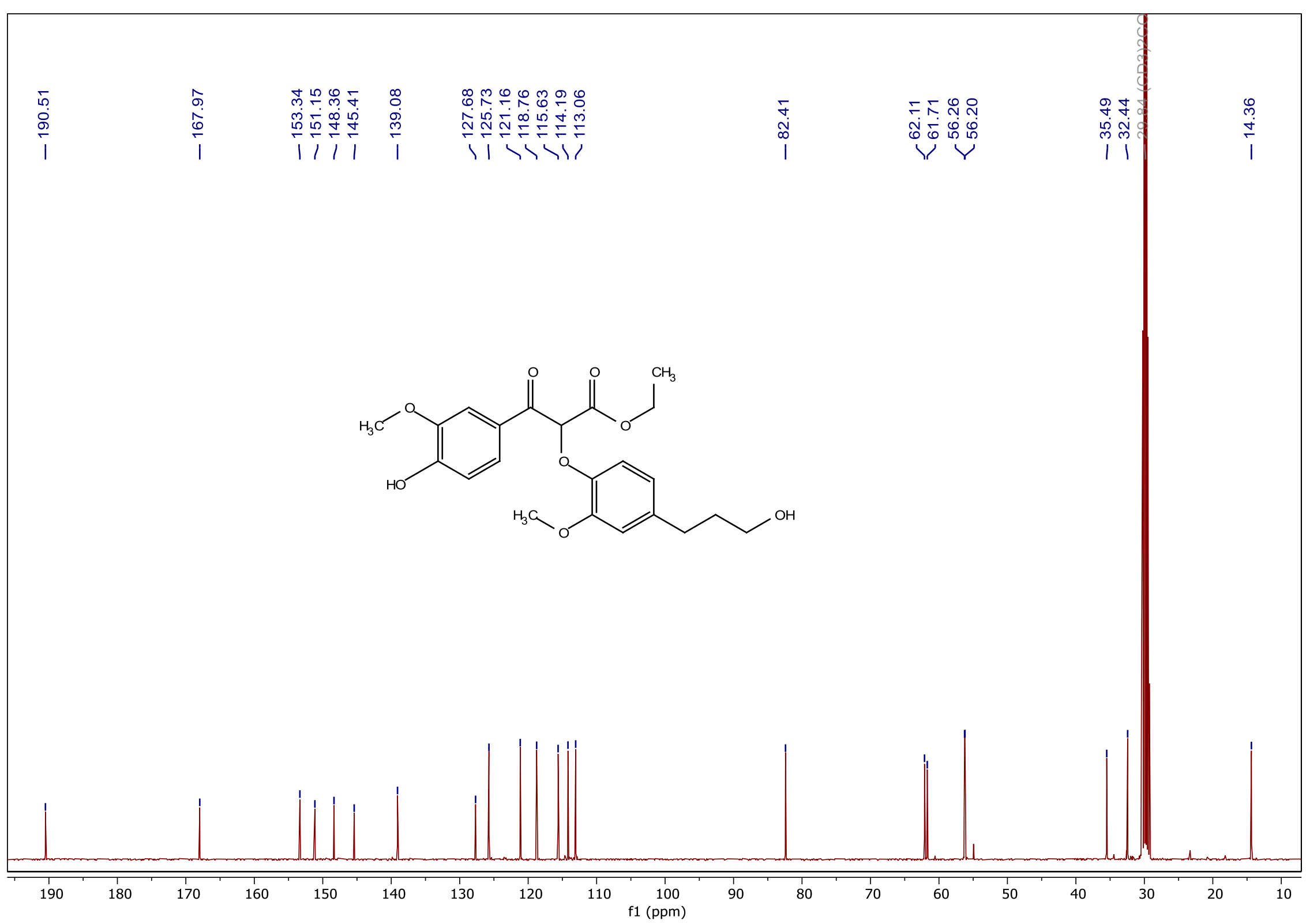

Figure S30. ^{13}C NMR (101 MHz, acetone-d_6) spectrum of compound **3h.**

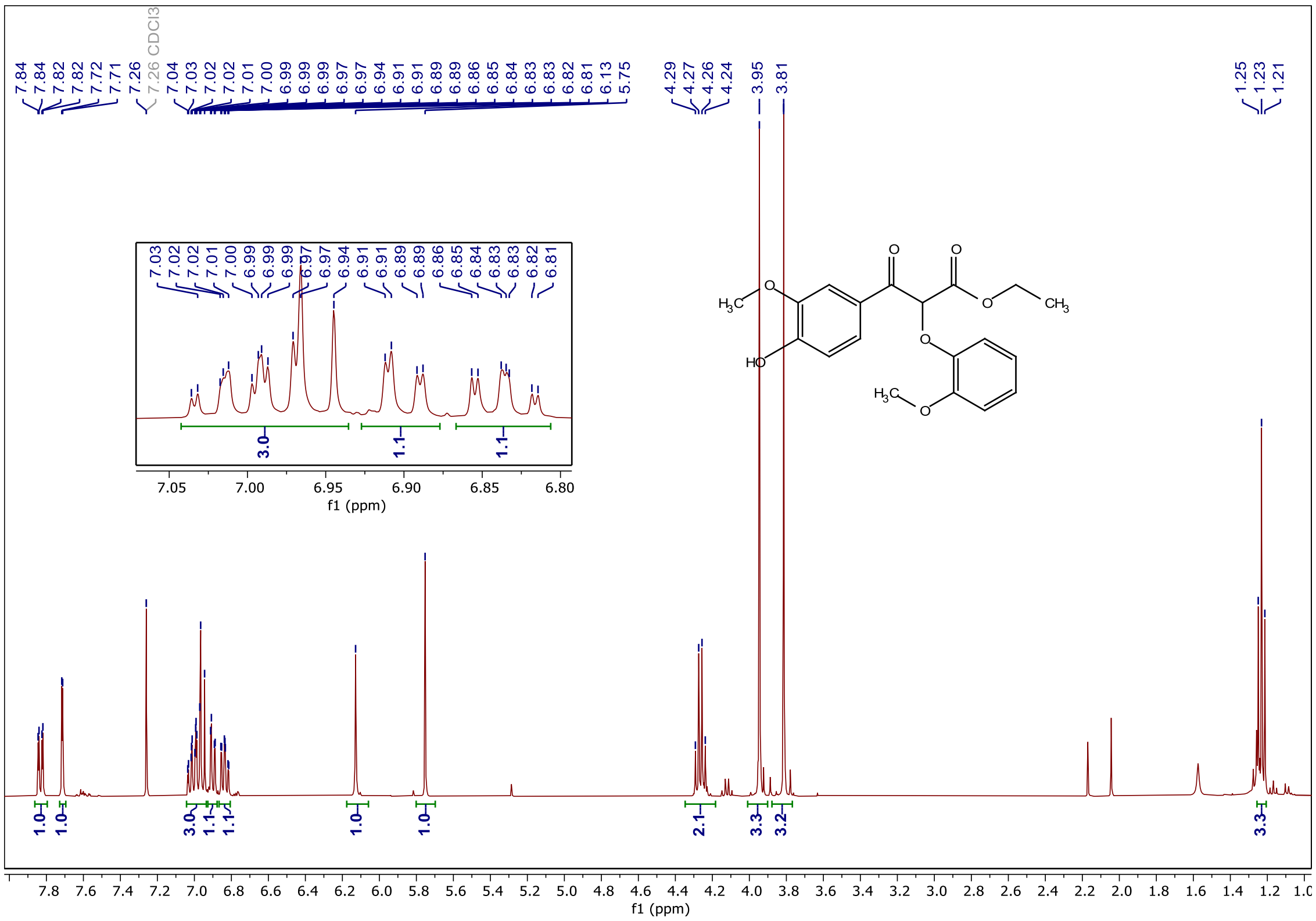

Figure S31. ^{1}H NMR (400 MHz, $CDCl_3$) spectrum of compound **3i.**

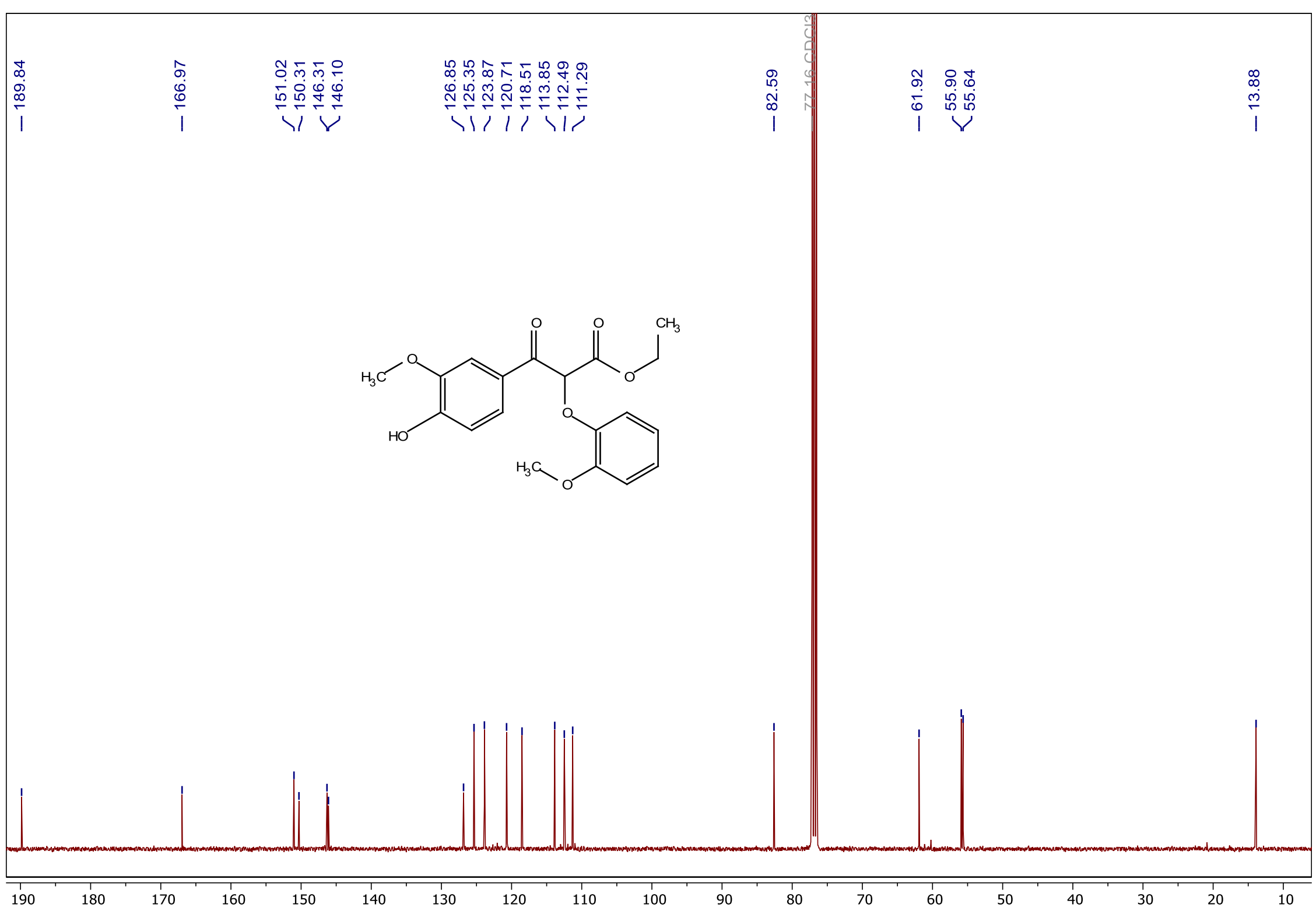

Figure S32. ^{13}C NMR (101 MHz, $CDCl_3$) spectrum of compound **3i.**

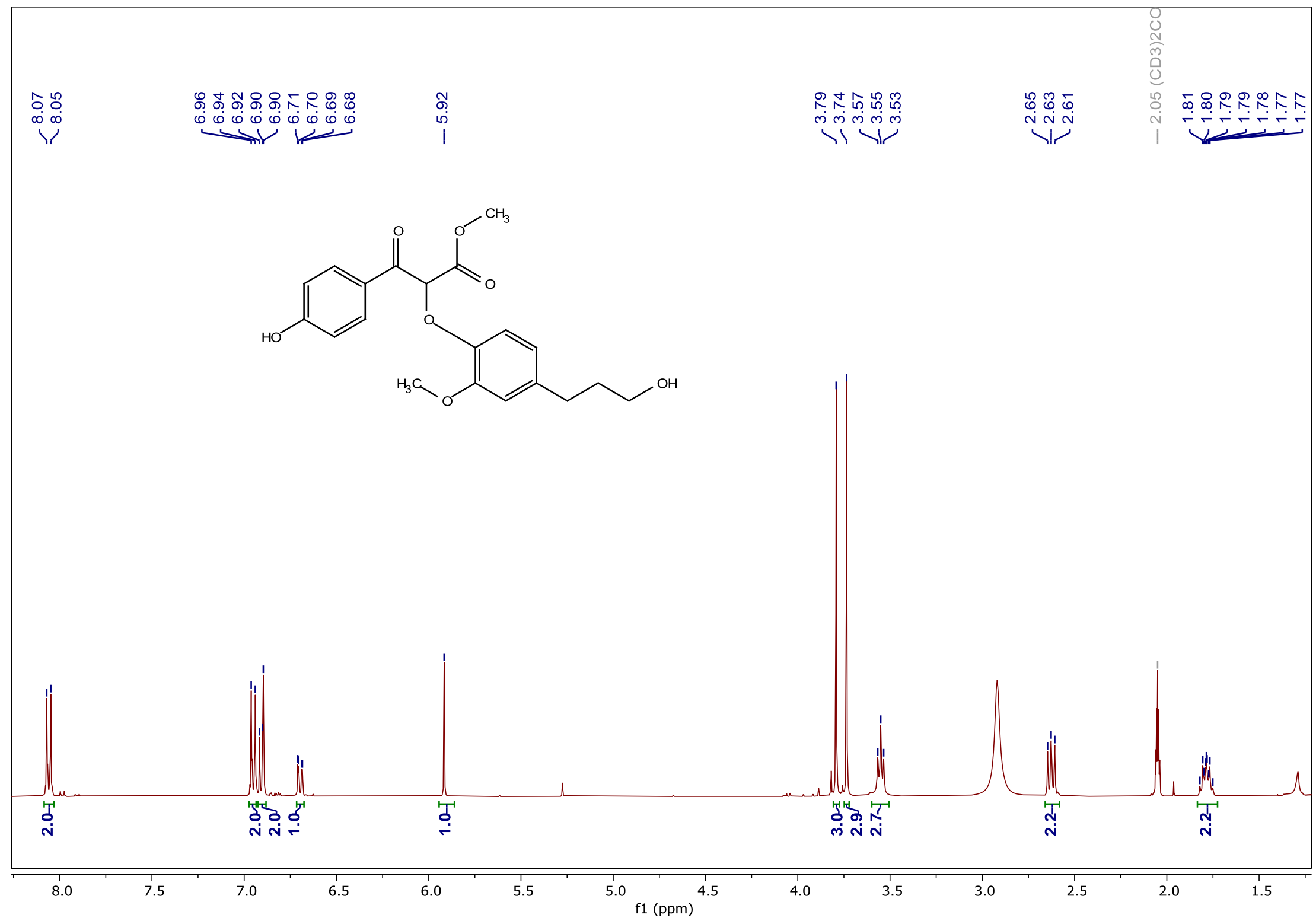

Figure S33. ^{1}H NMR (400 MHz, acetone-d_6) spectrum of compound **3j.**

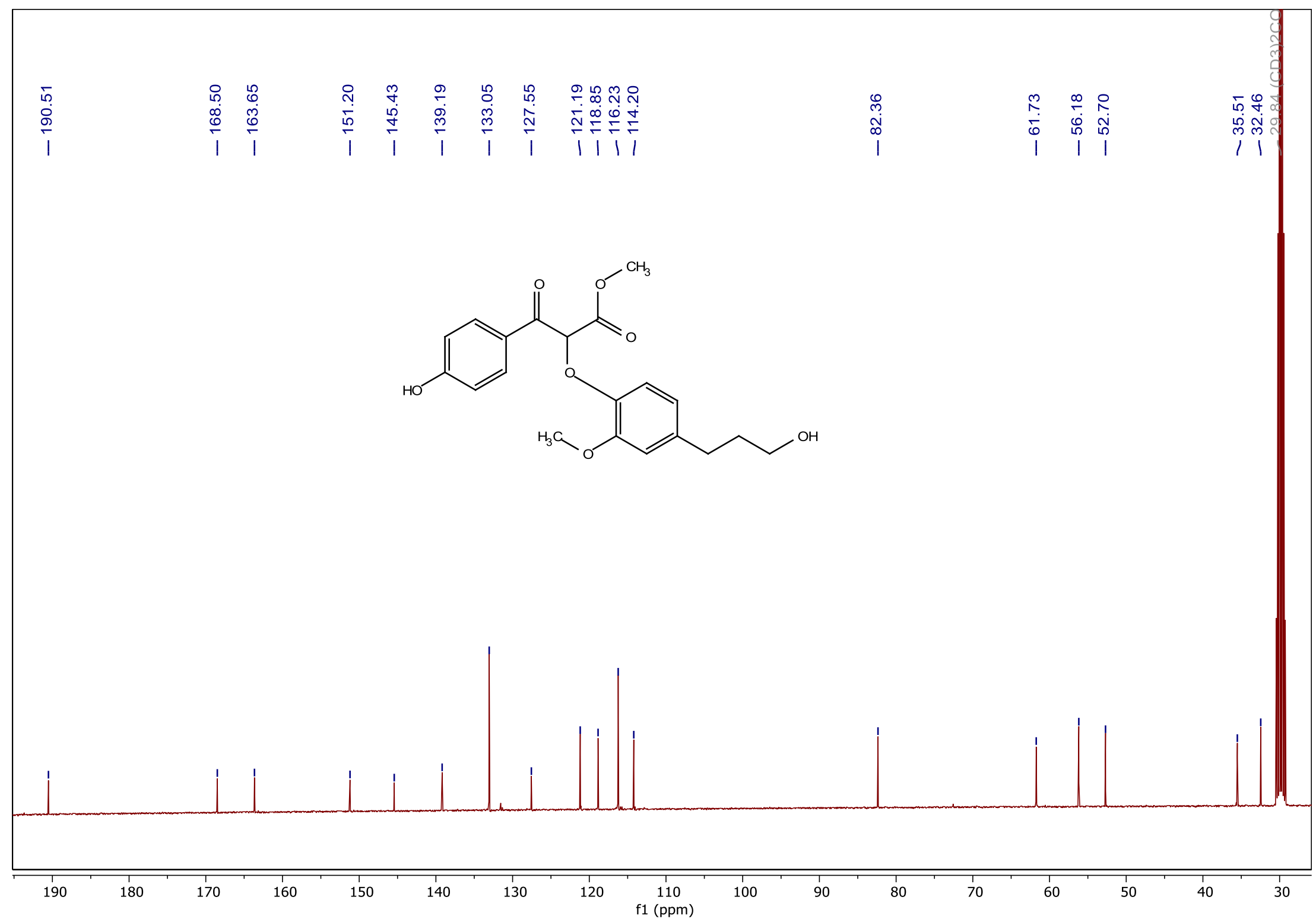

Figure S34. ^{13}C NMR (101 MHz, acetone-d_6) spectrum of compound **3j.**

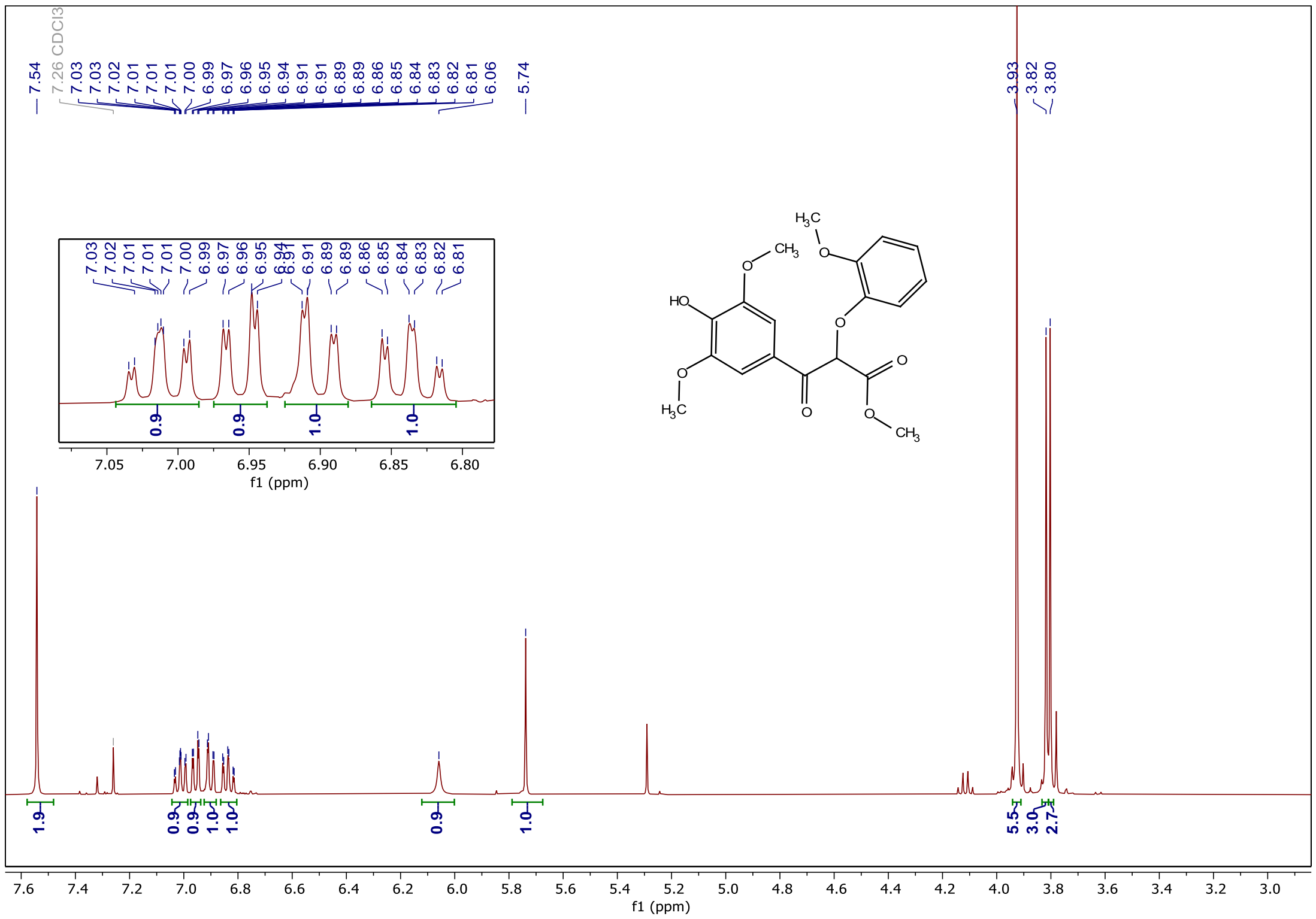

Figure S35. ^{1}H NMR (400 MHz, $CDCl_3$) spectrum of compound **3k.**

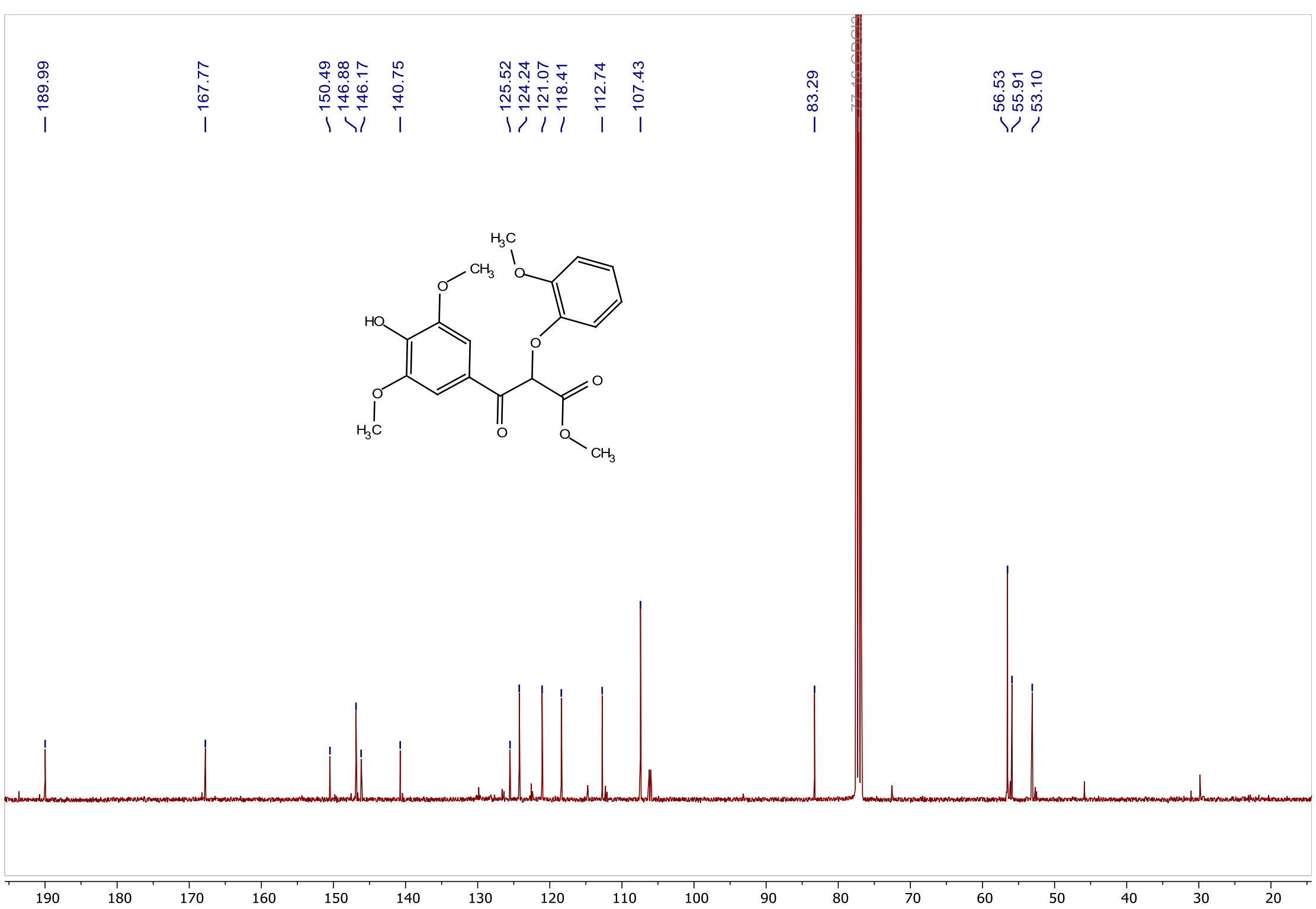

Figure S36. ^{13}C NMR (101 MHz, $CDCl_3$) spectrum of compound **3k.**

NMR Spectra of Trimers 4a-i:

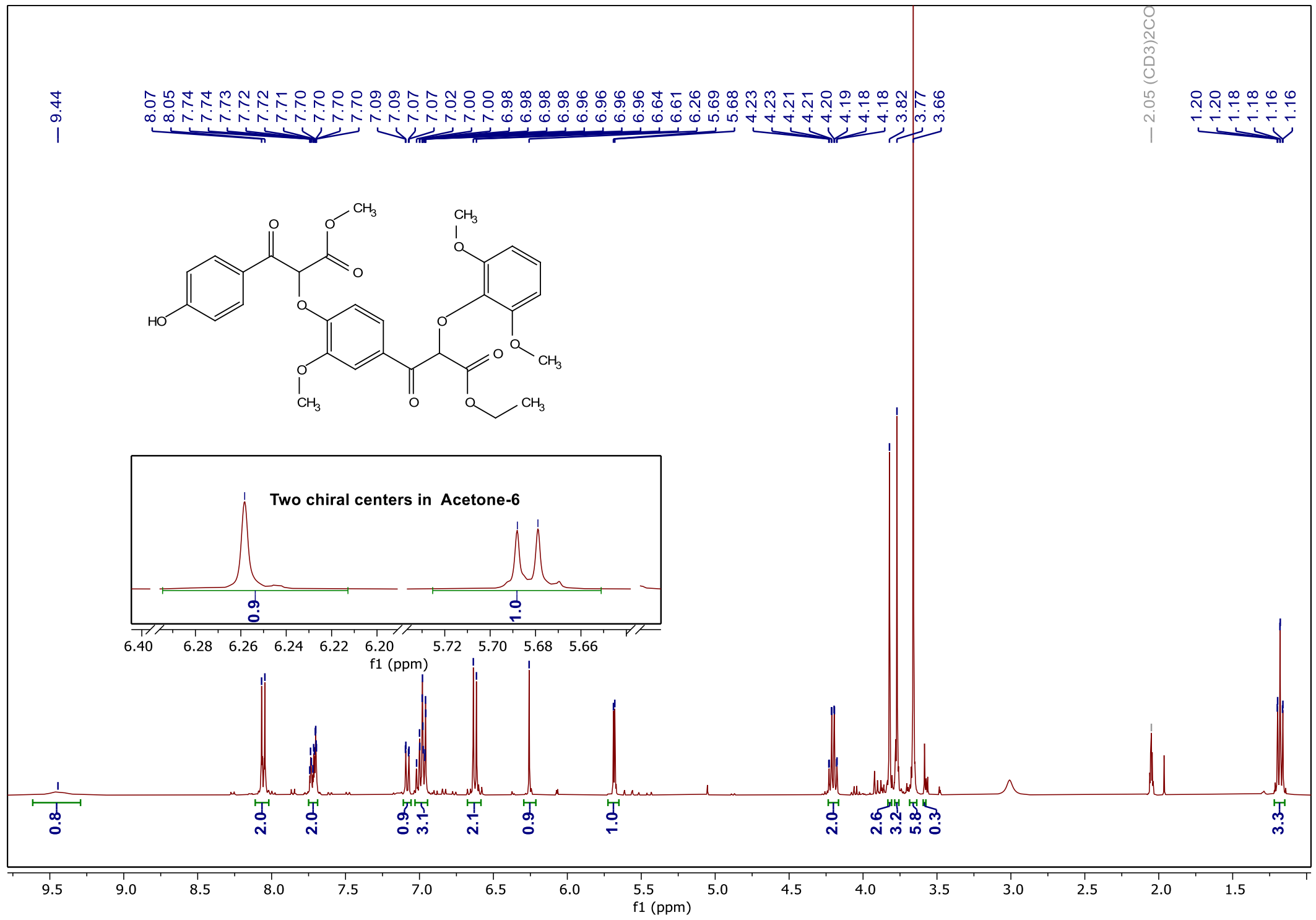

Figure S37. ^{1}H NMR (400 MHz, acetone-d$_6$) spectrum of compound **4a.**

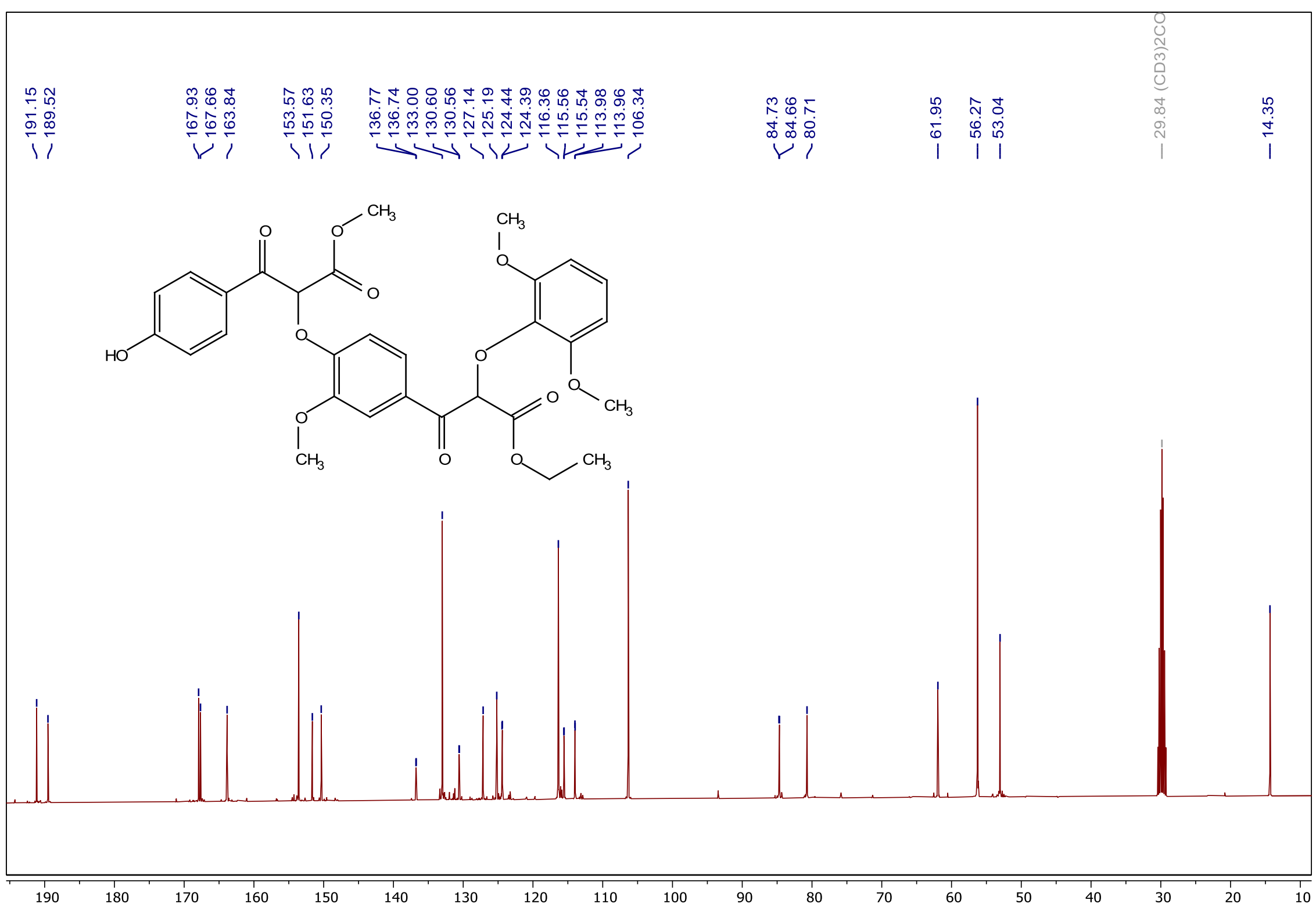

Figure S38. ^{13}C NMR (101 MHz, acetone-d$_6$) spectrum of compound **4a.**

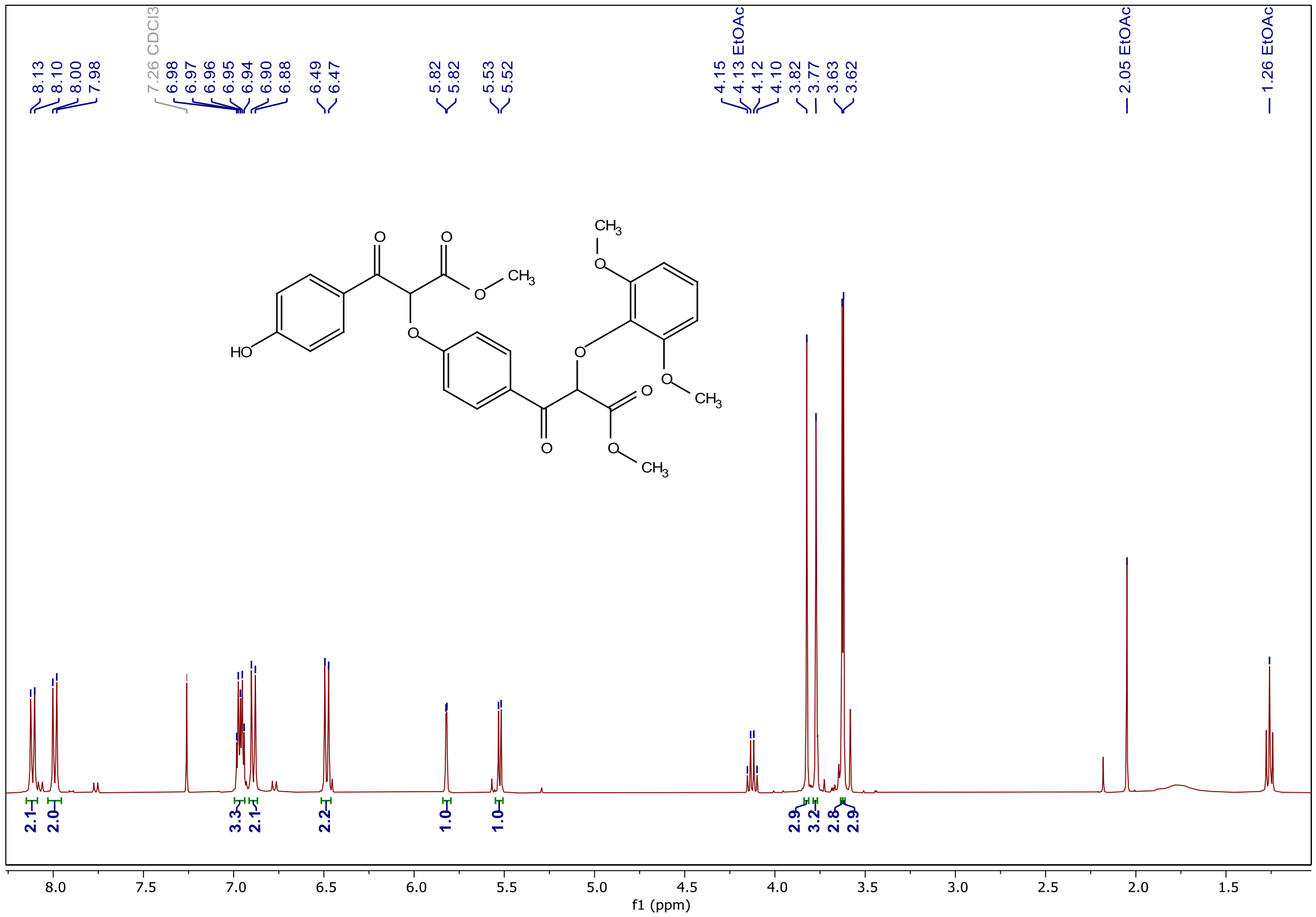

Figure S39. ^{1}H NMR (400 MHz, $CDCl_3$) spectrum of compound **4b.**

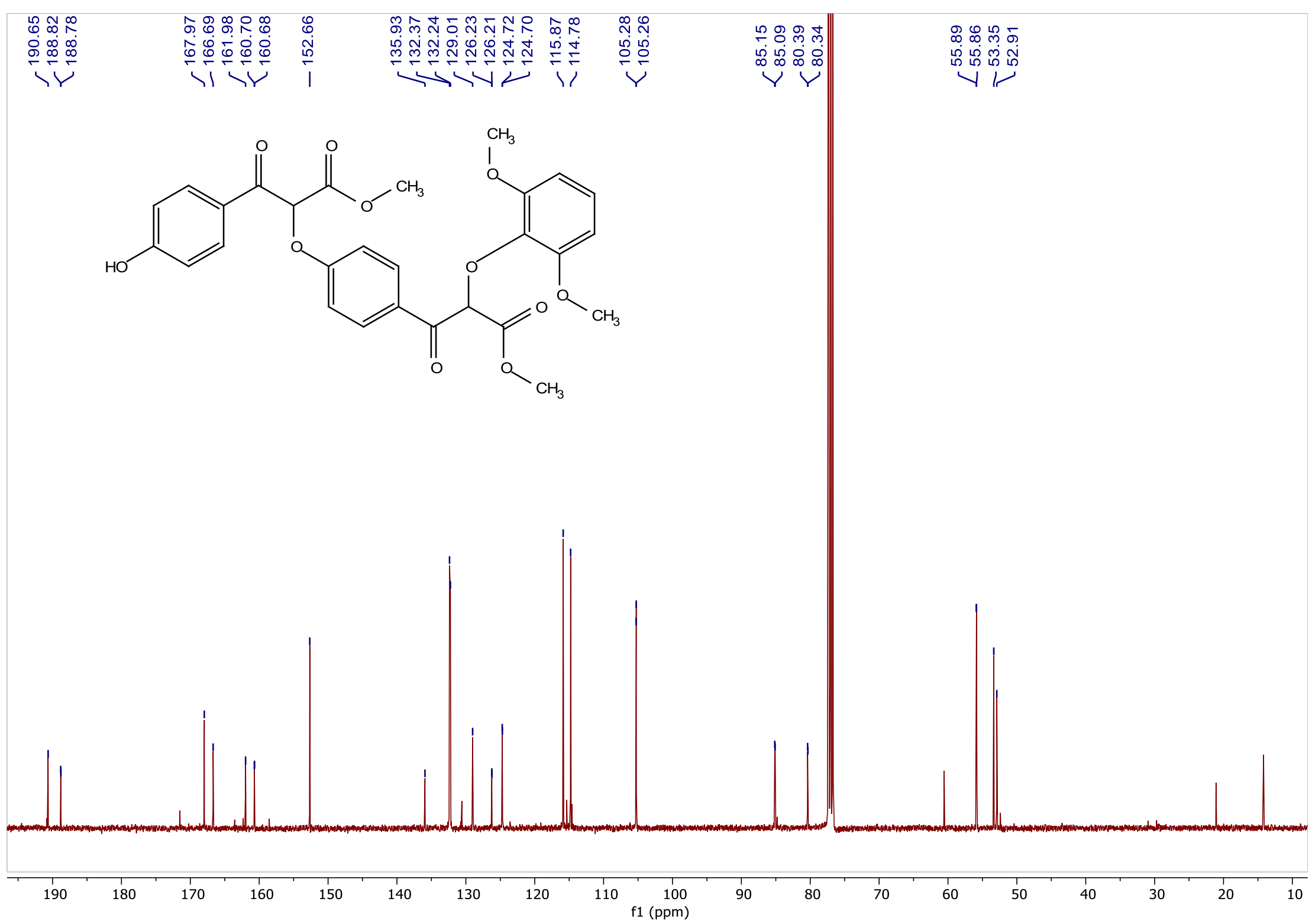

Figure S40. ^{13}C NMR (101 MHz, $CDCl_3$) spectrum of compound **4b.**

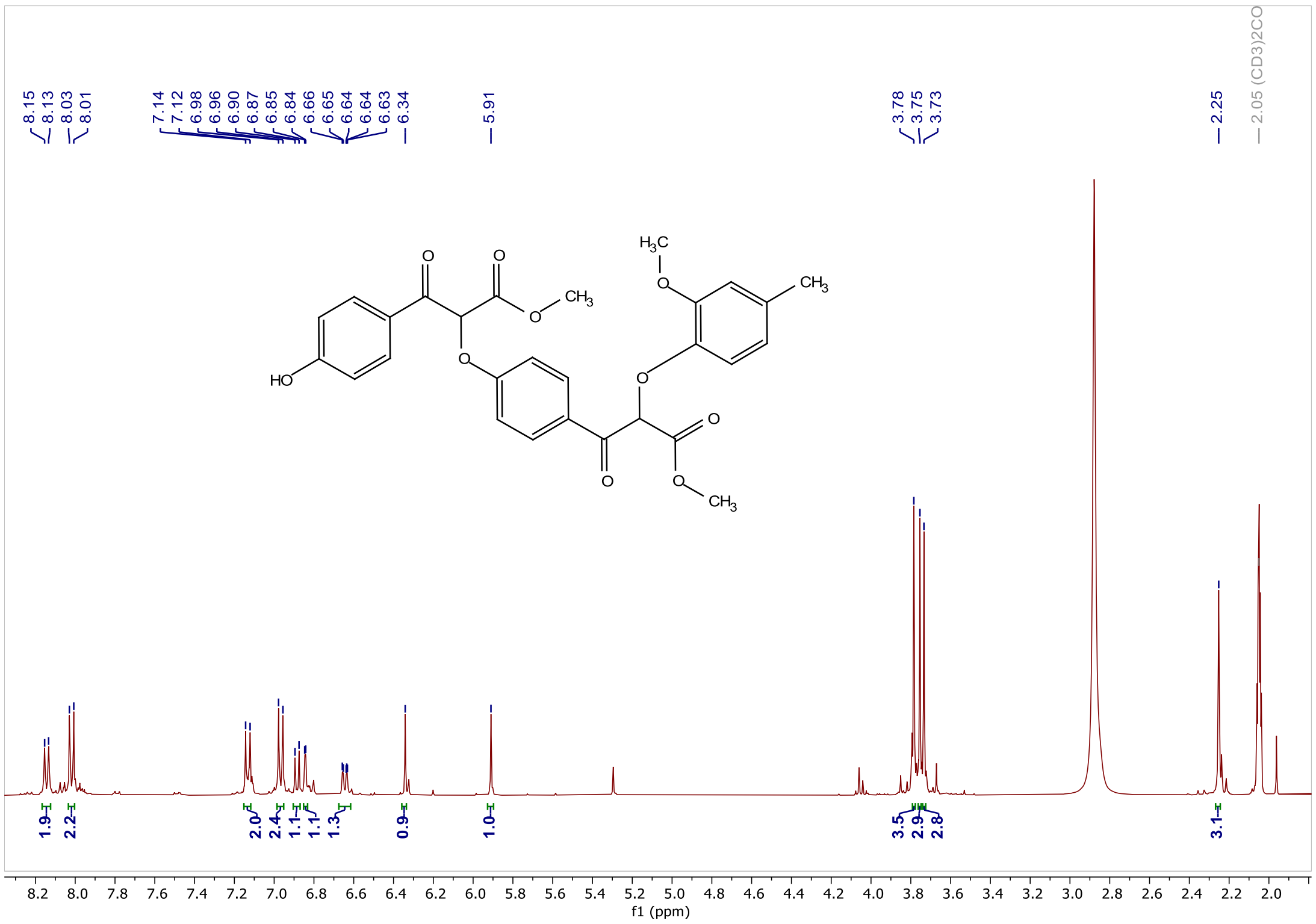

Figure S41. ^{1}H NMR (400 MHz, acetone-d_6) spectrum of compound **4c.**

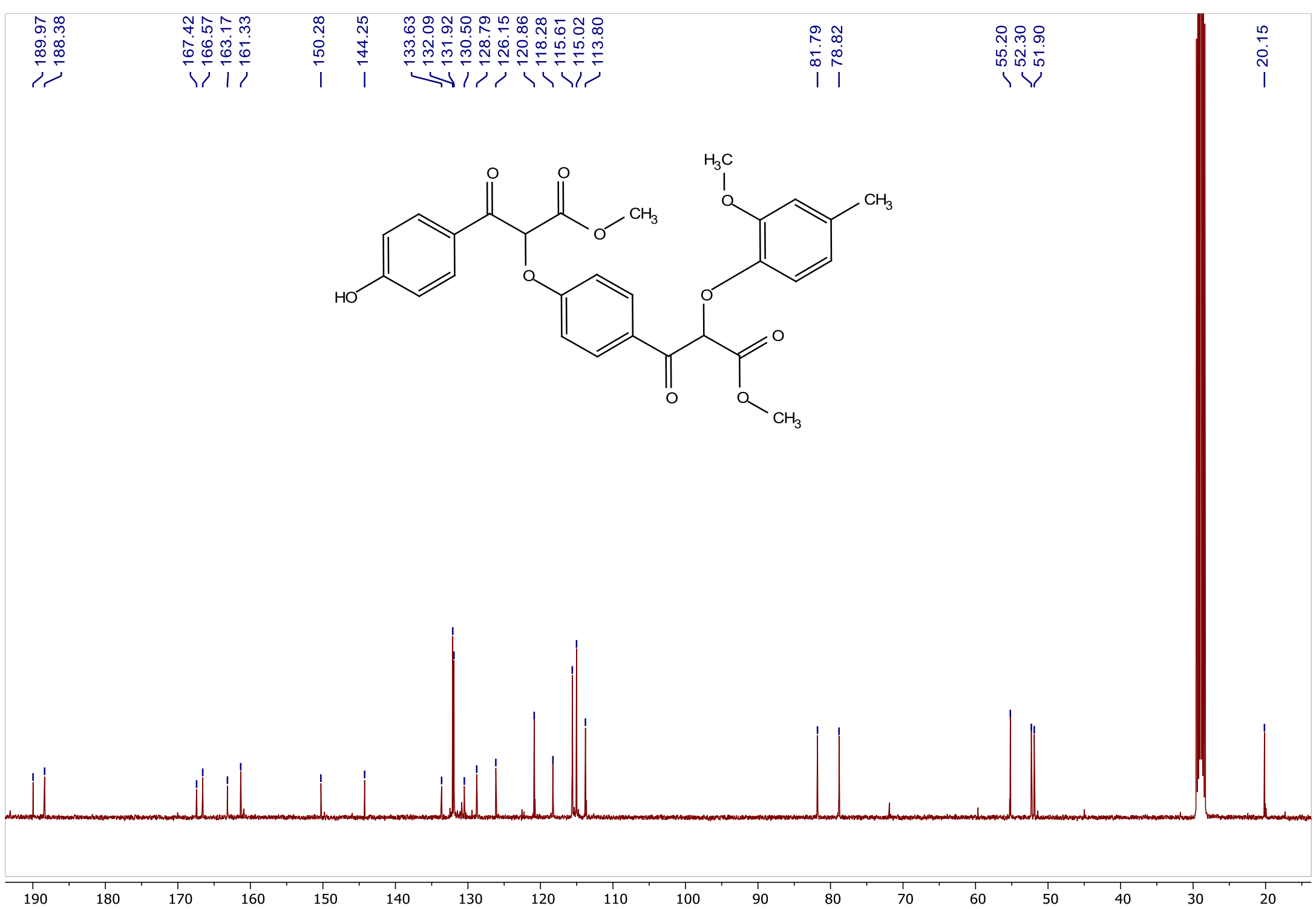

Figure S42. ^{13}C NMR (101 MHz, acetone-d_6) spectrum of compound **4c.**

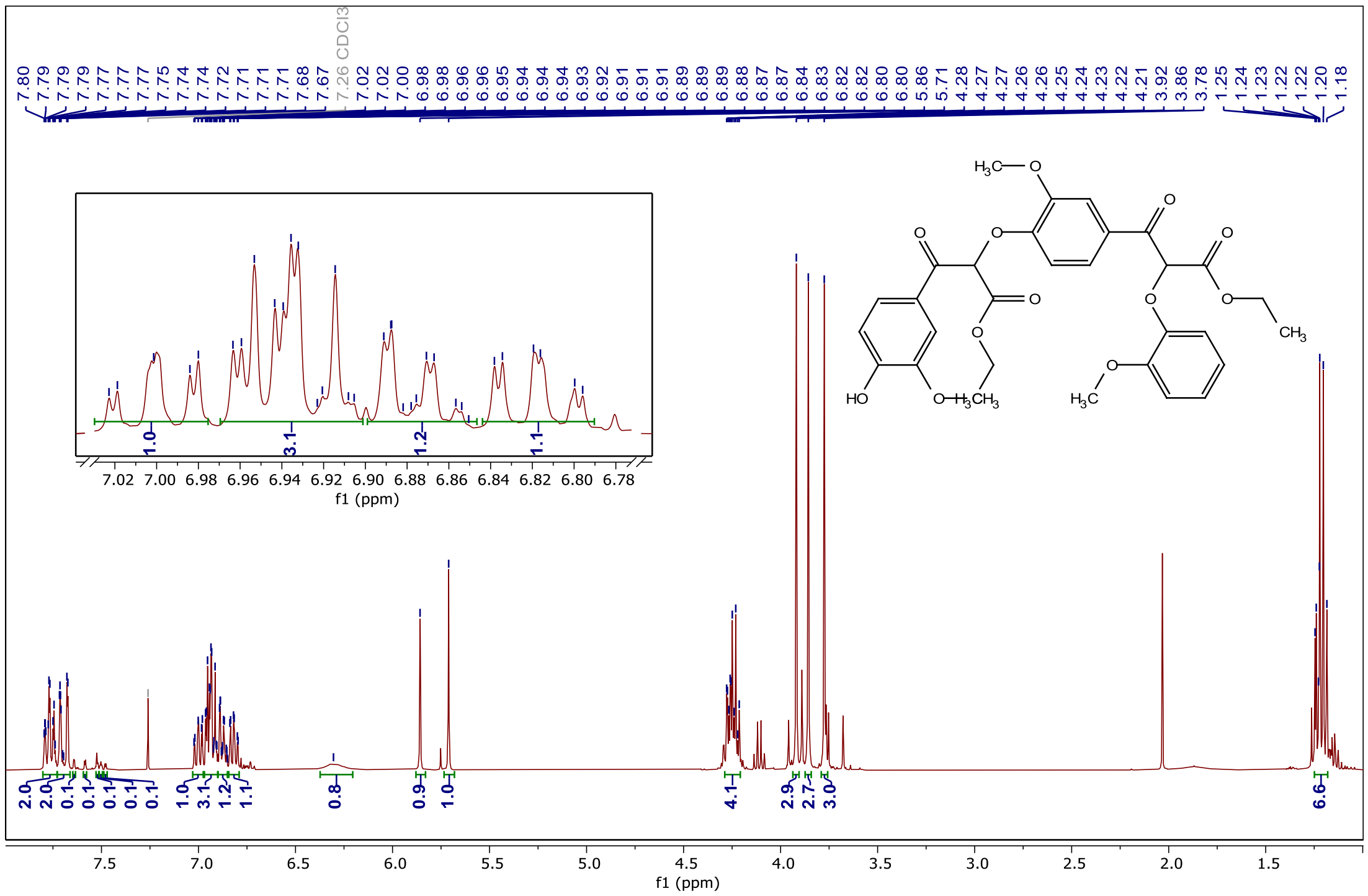

Figure S43. ^{1}H NMR (400 MHz, $CDCl_3$) spectrum of compound **4d**.

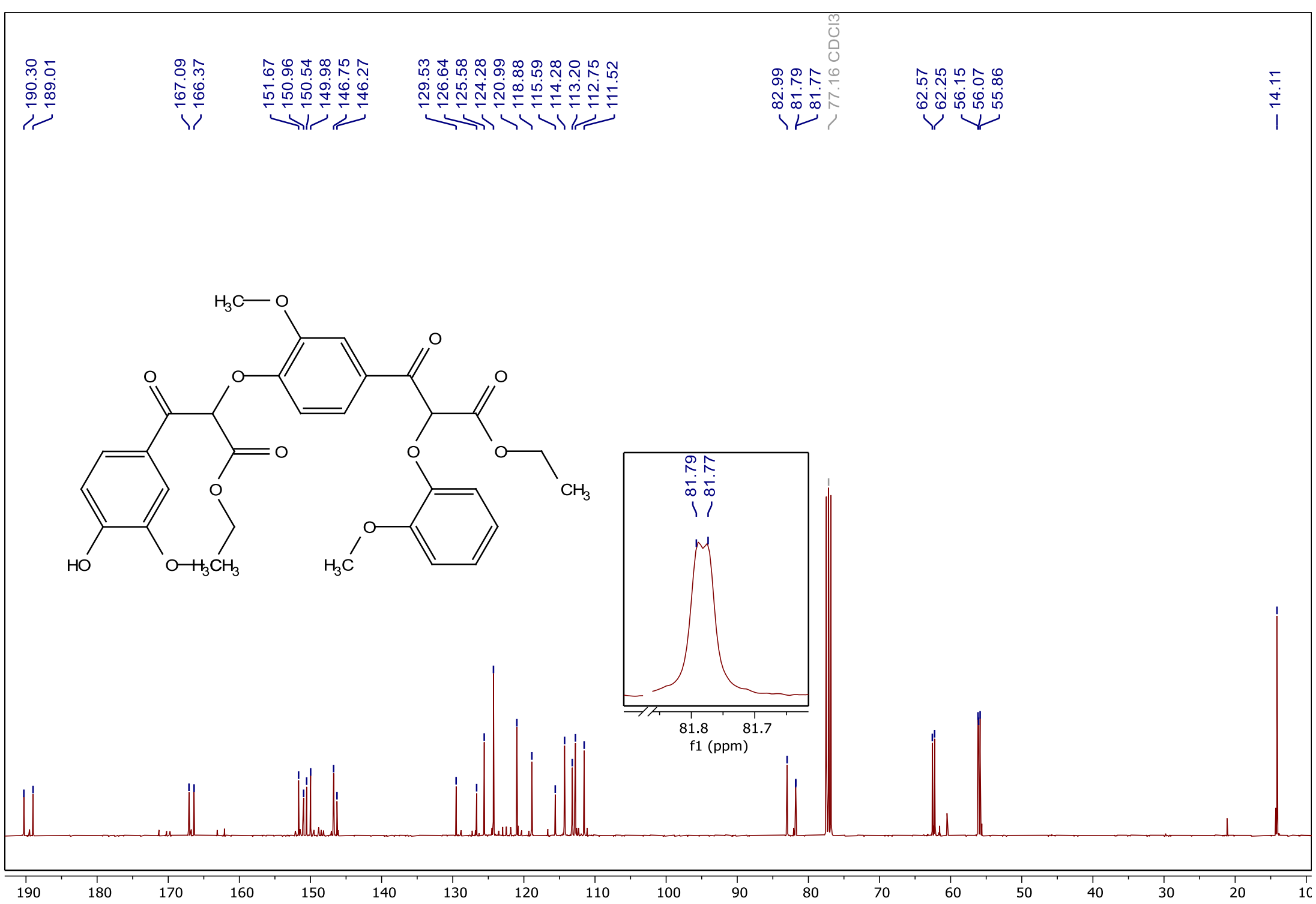

Figure S44. ^{13}C NMR (101 MHz, $CDCl_3$) spectrum of compound **4d**.

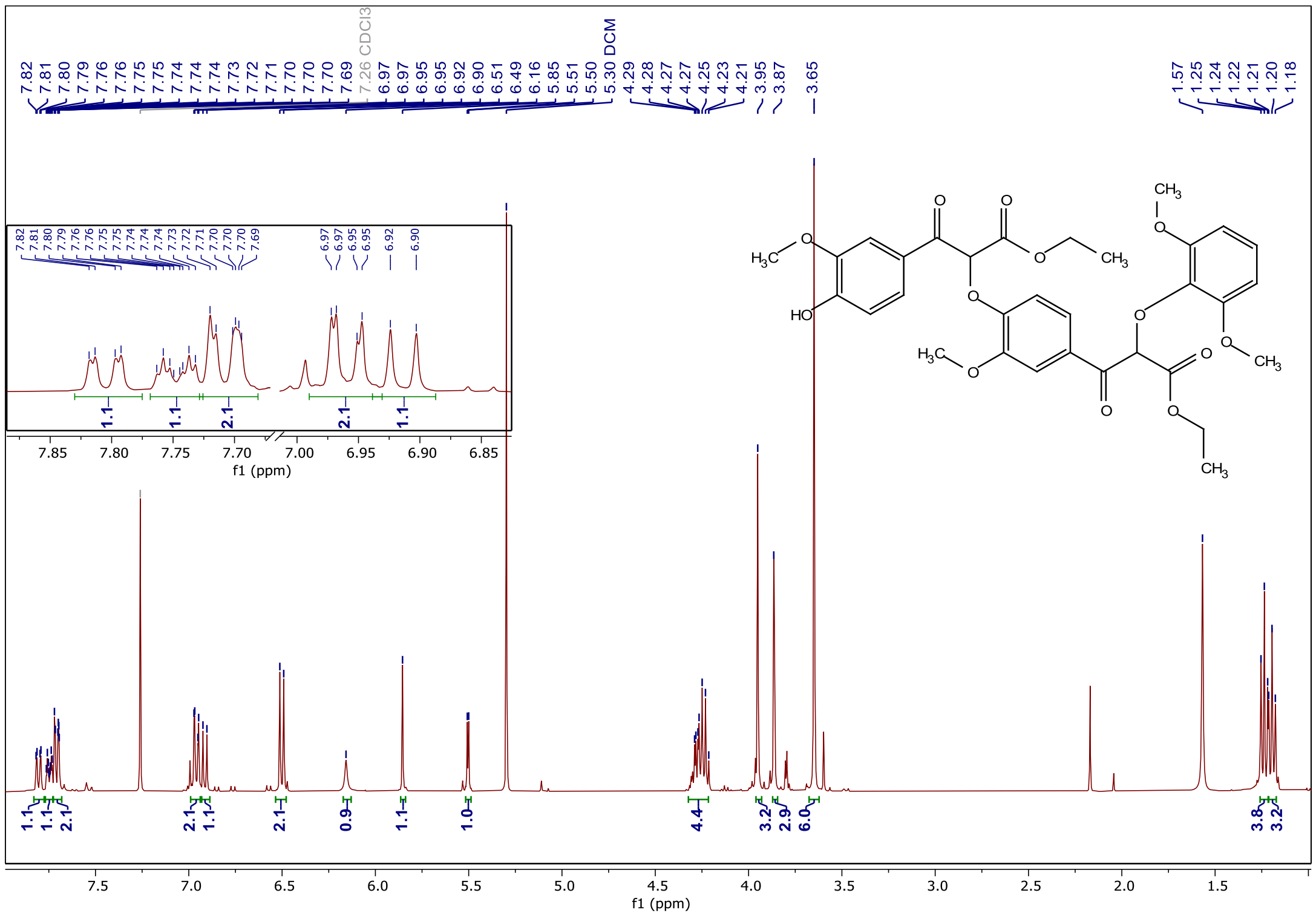

Figure S45. ^{1}H NMR (400 MHz, $CDCl_3$) spectrum of compound **4e.**

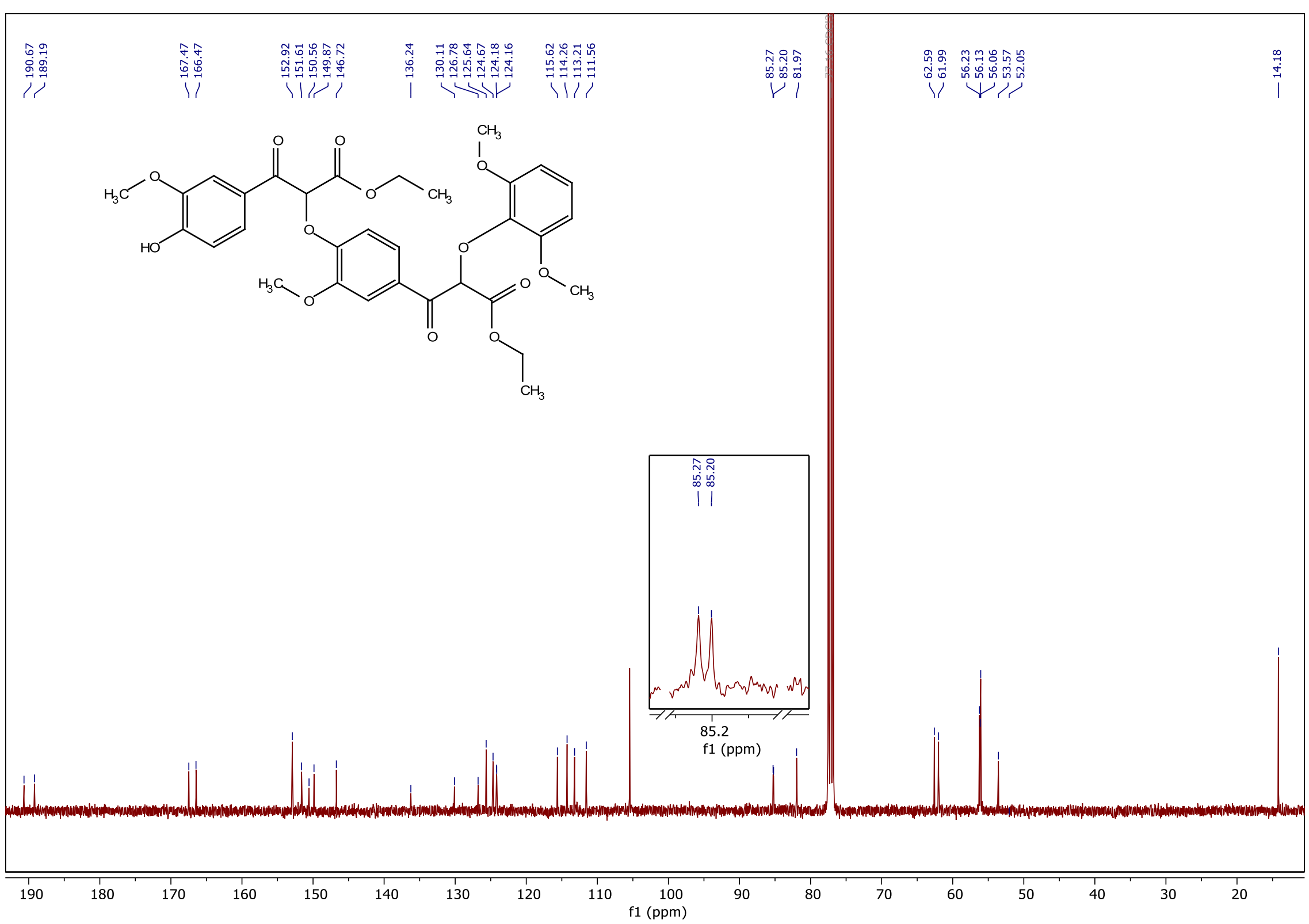

Figure S46. ^{13}C NMR (101 MHz, $CDCl_3$) spectrum of compound **4e.**

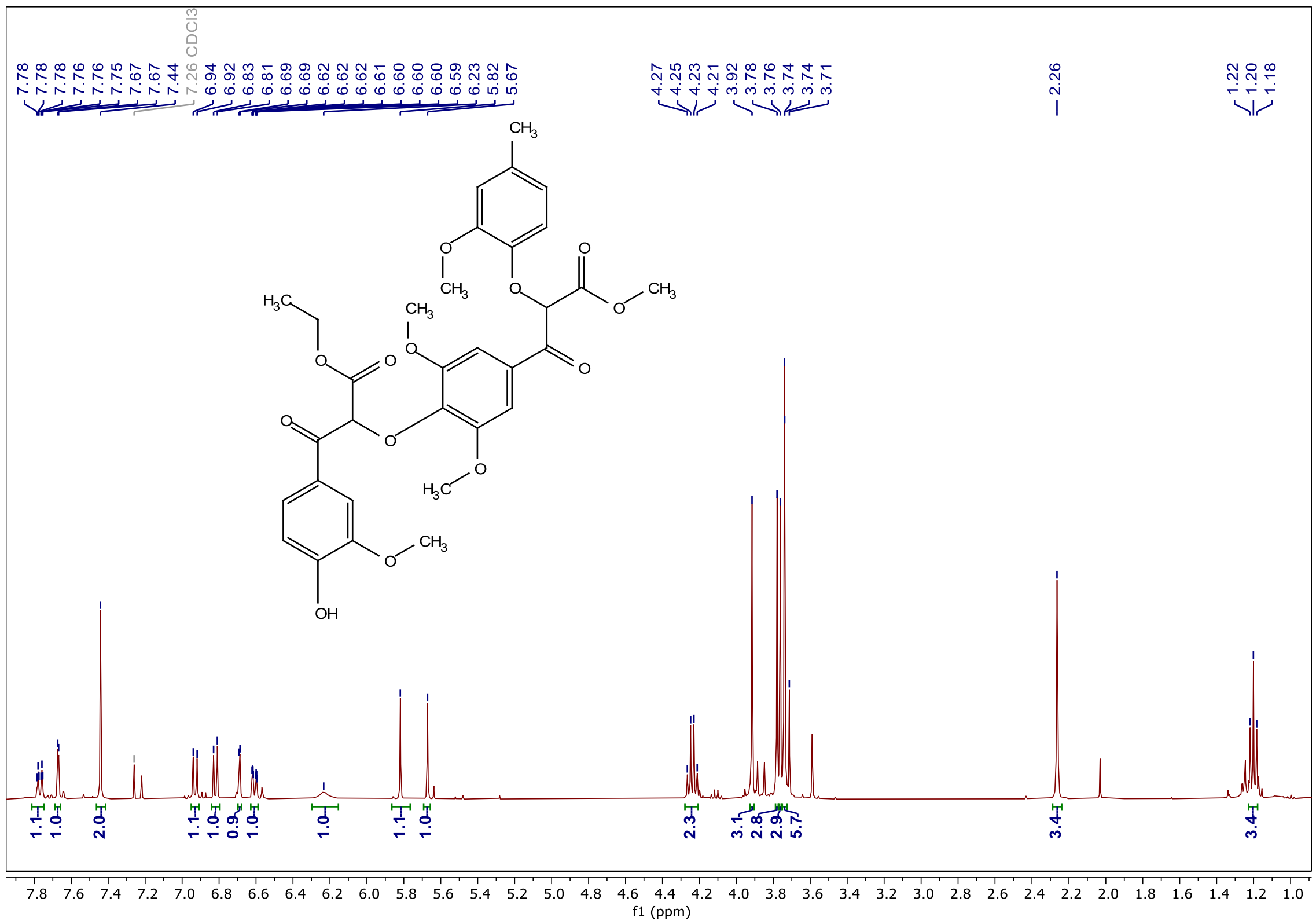

Figure S47. ^{1}H NMR (400 MHz, $CDCl_3$) spectrum of compound **4f.**

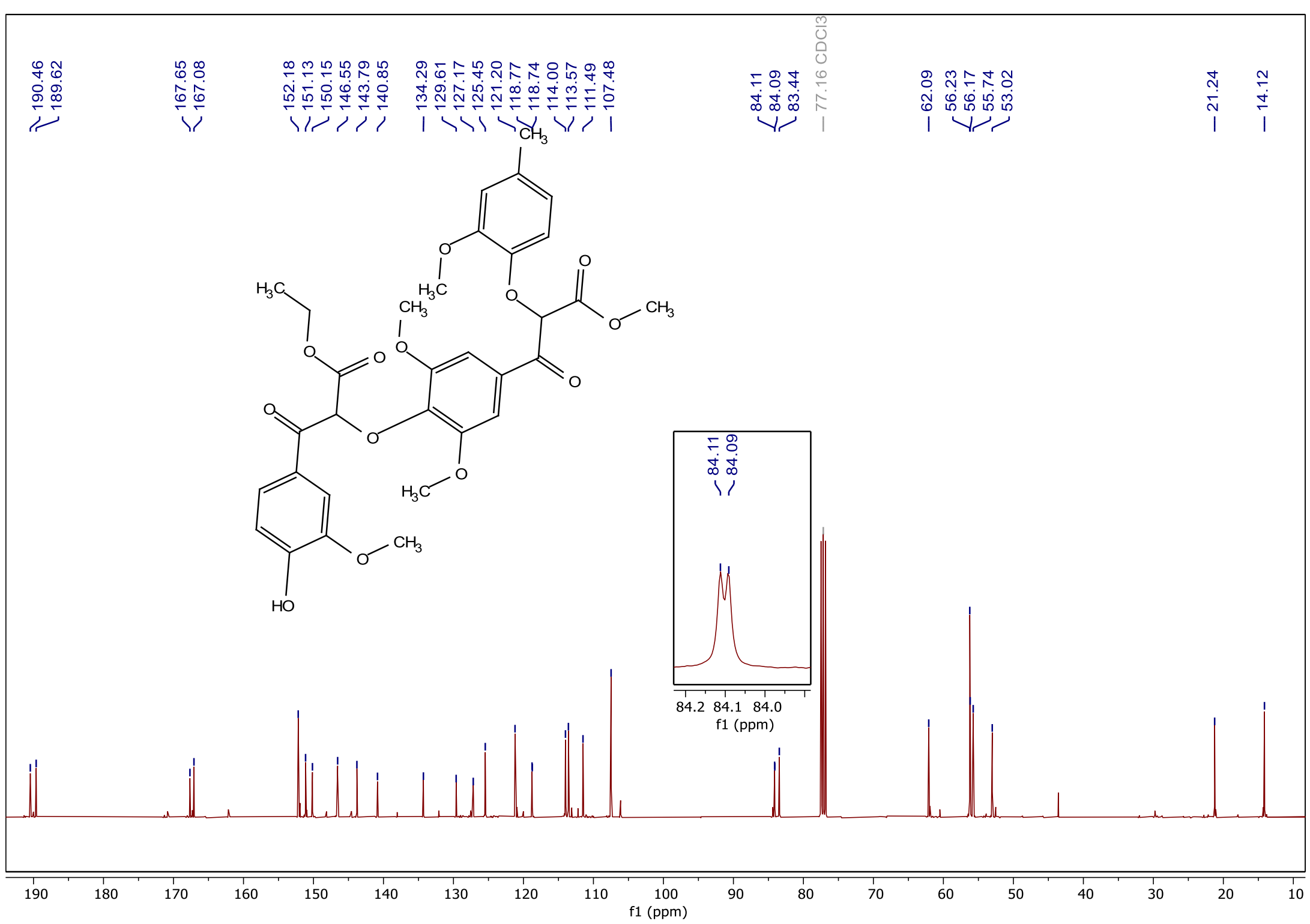

Figure S48. ^{13}C NMR (101 MHz, $CDCl_3$) spectrum of compound **4f.**

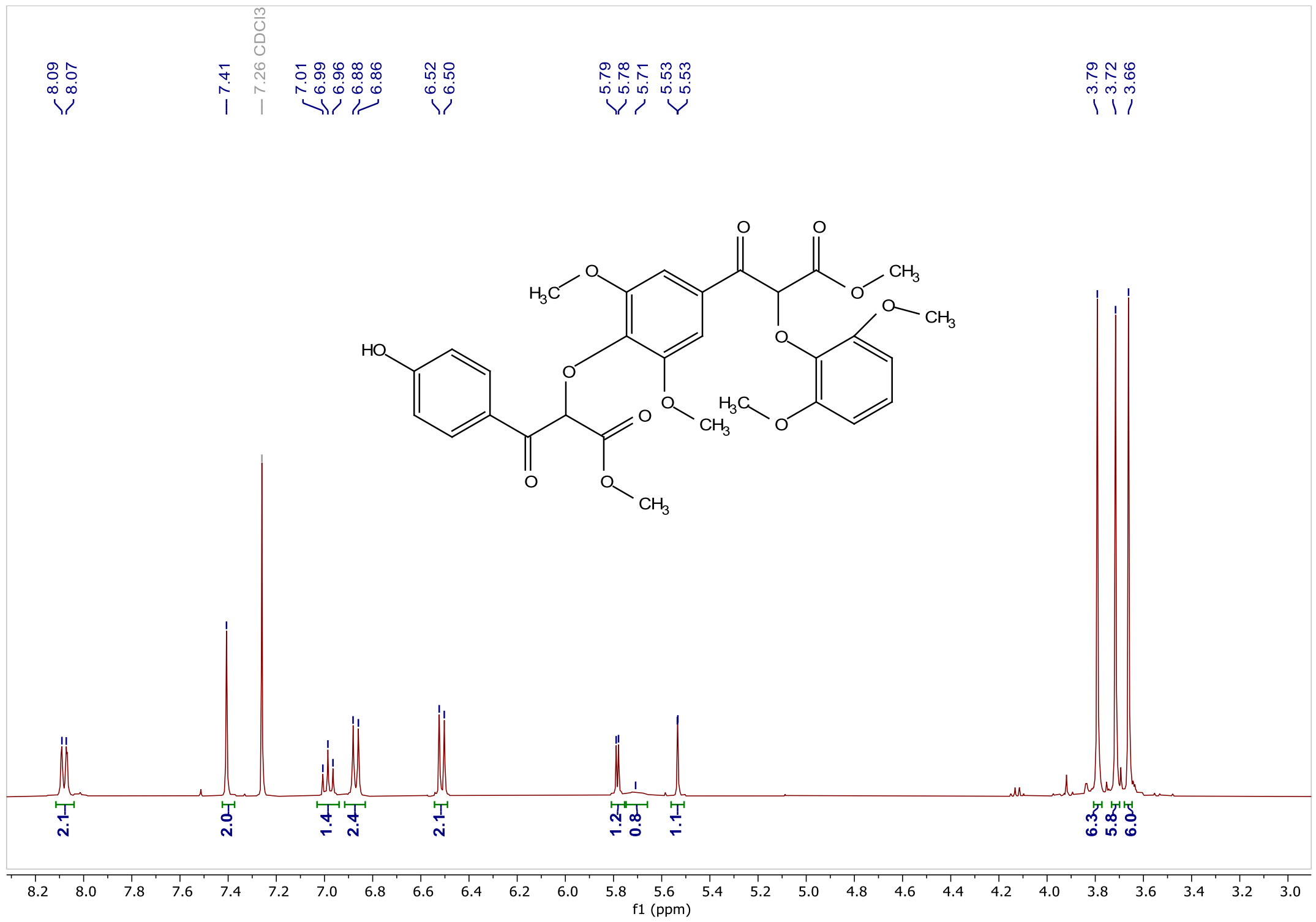

Figure S49. ^{1}H NMR (400 MHz, $CDCl_3$) spectrum of compound **4g.**

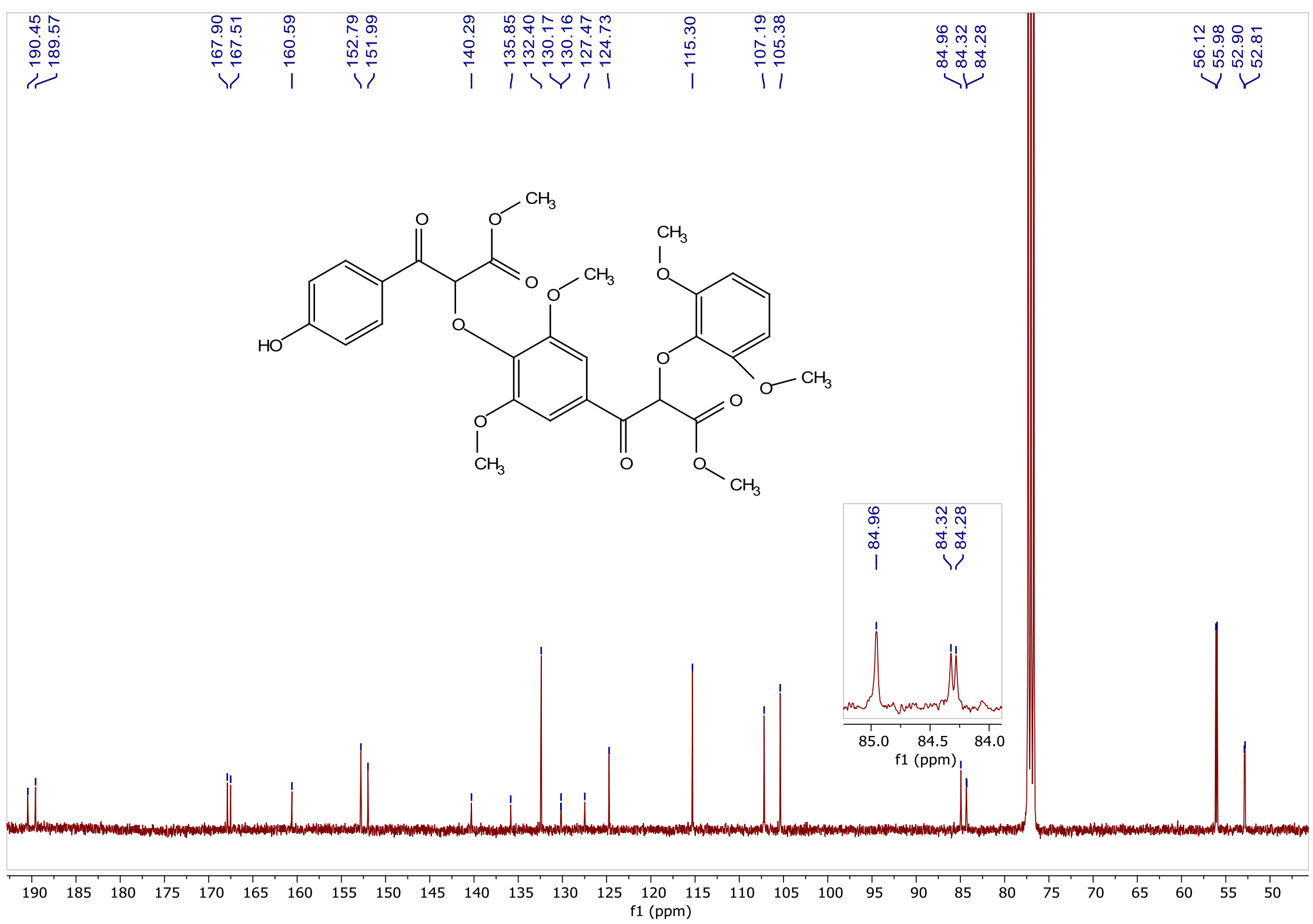

Figure S50. ^{13}C NMR (101 MHz, $CDCl_3$) spectrum of compound **4g.**

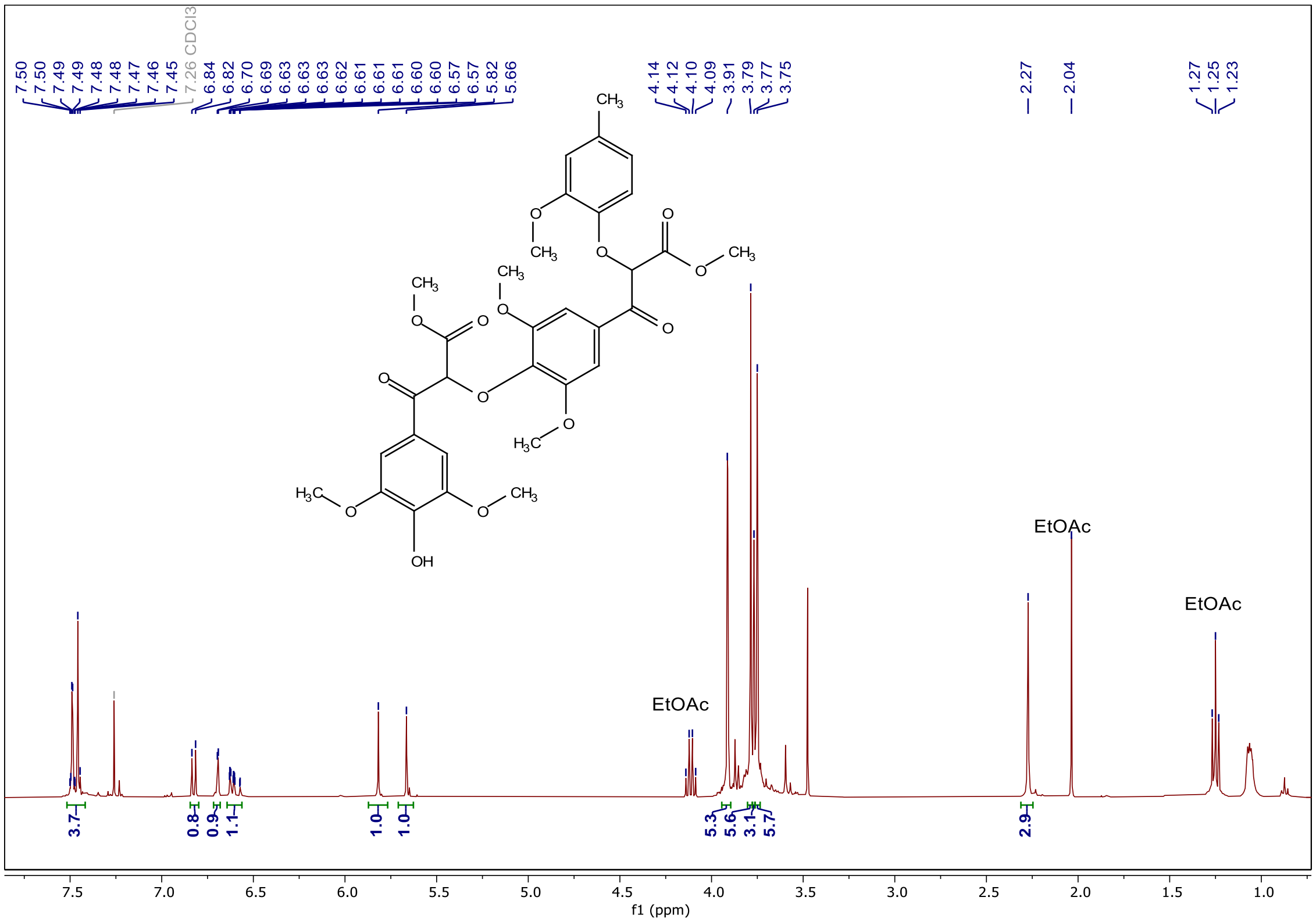

Figure S51. ^{1}H NMR (400 MHz, $CDCl_3$) spectrum of compound **4h.**

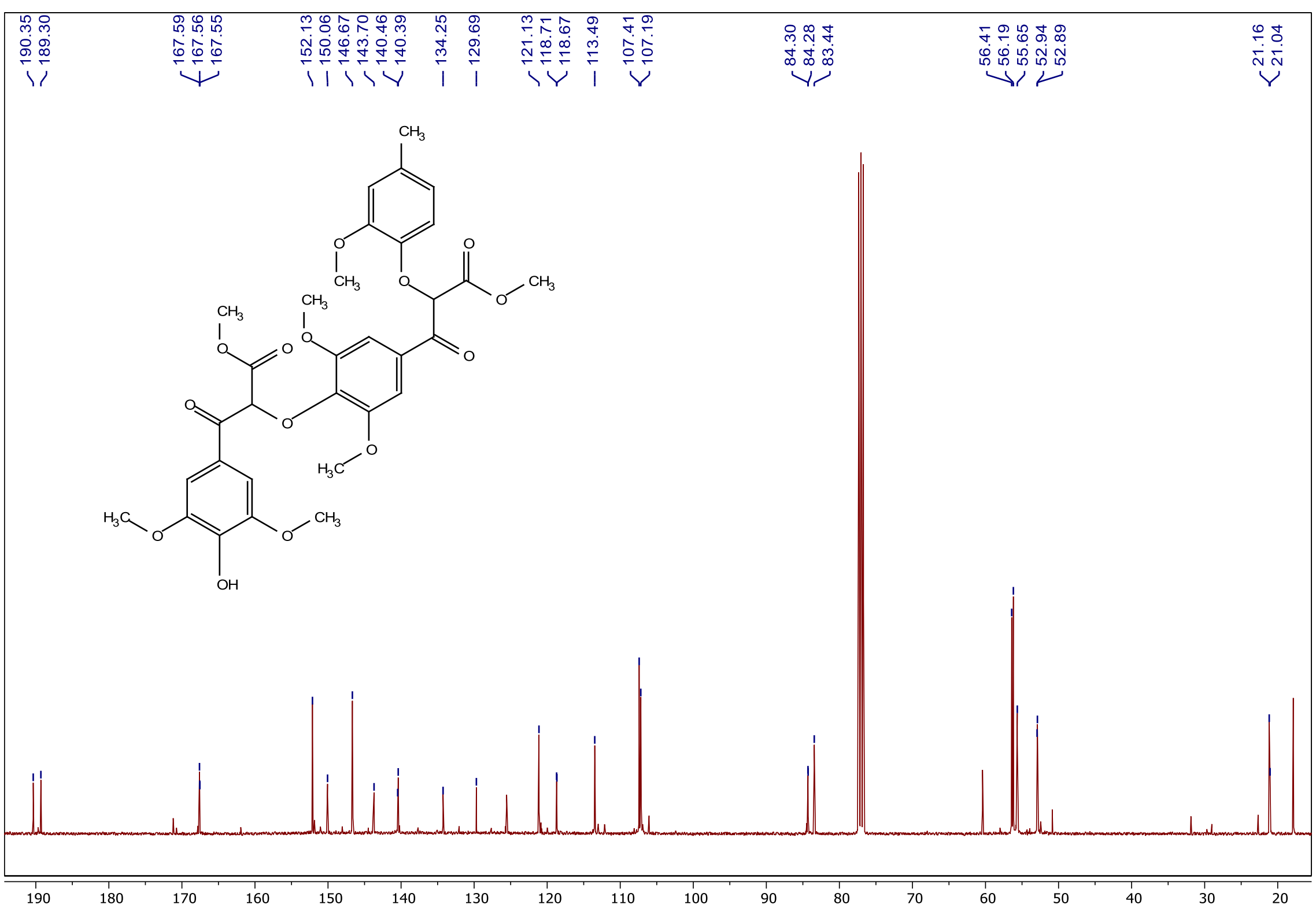

Figure S52. ^{13}C NMR (101 MHz, $CDCl_3$) spectrum of compound **4h.**

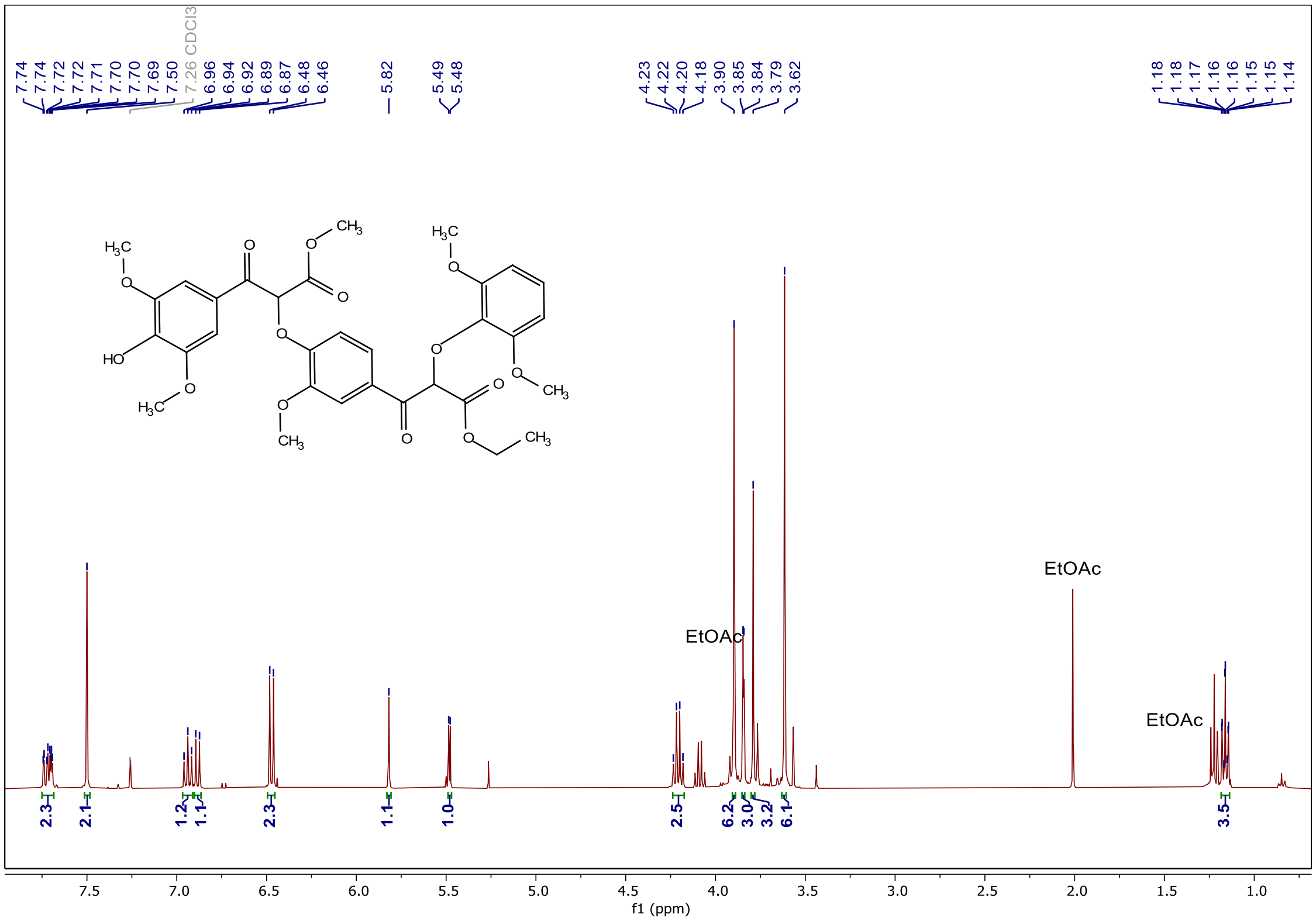

Figure S53. ^{1}H NMR (400 MHz, $CDCl_3$) spectrum of compound **4i.**

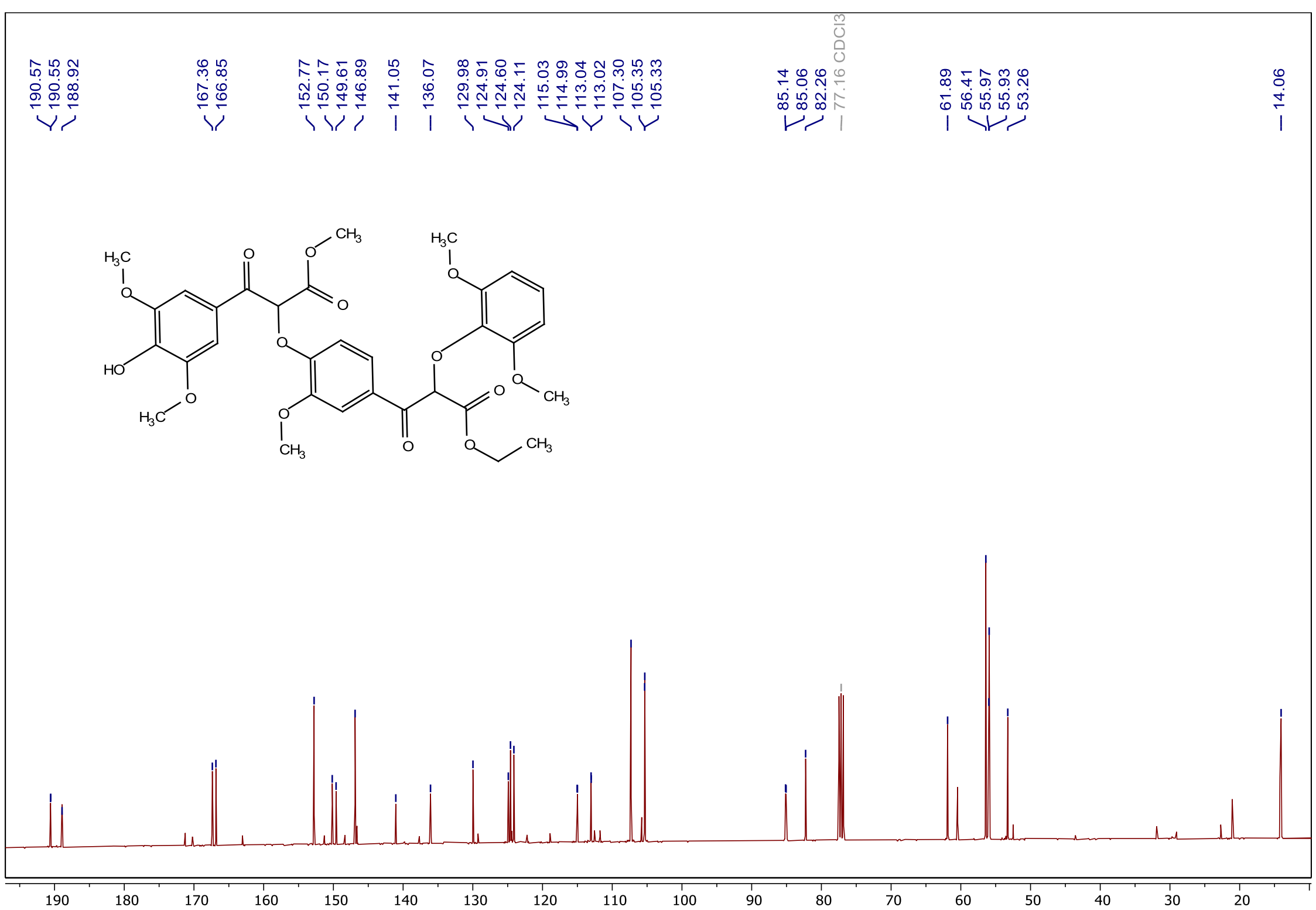

Figure S54. ^{13}C NMR (101 MHz, $CDCl_3$) spectrum of compound **4i.**

NMR Spectra of Tetramers 5:

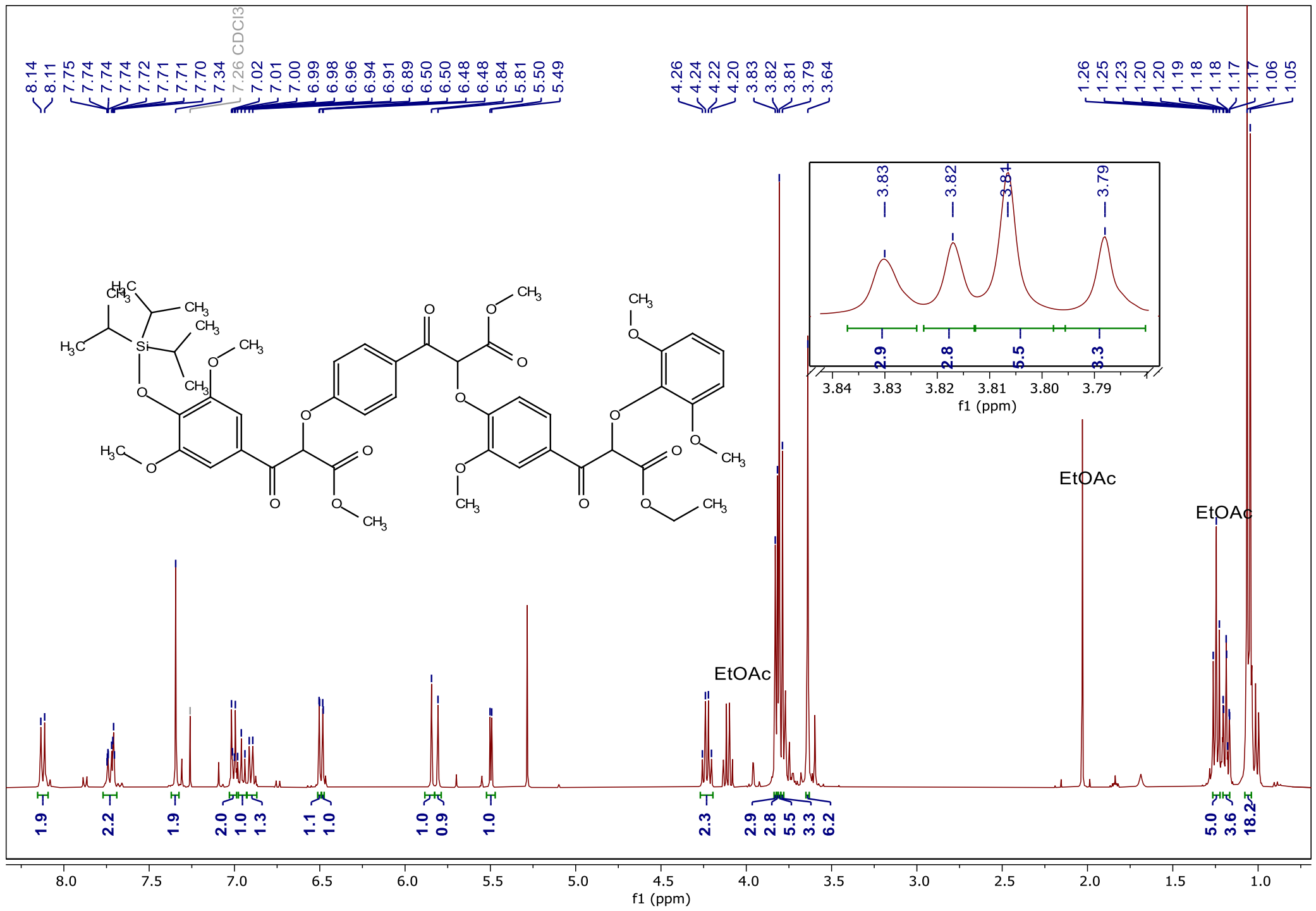

Figure S55. ^{1}H NMR (400 MHz, $CDCl_3$) spectrum of **5-TIPS.**

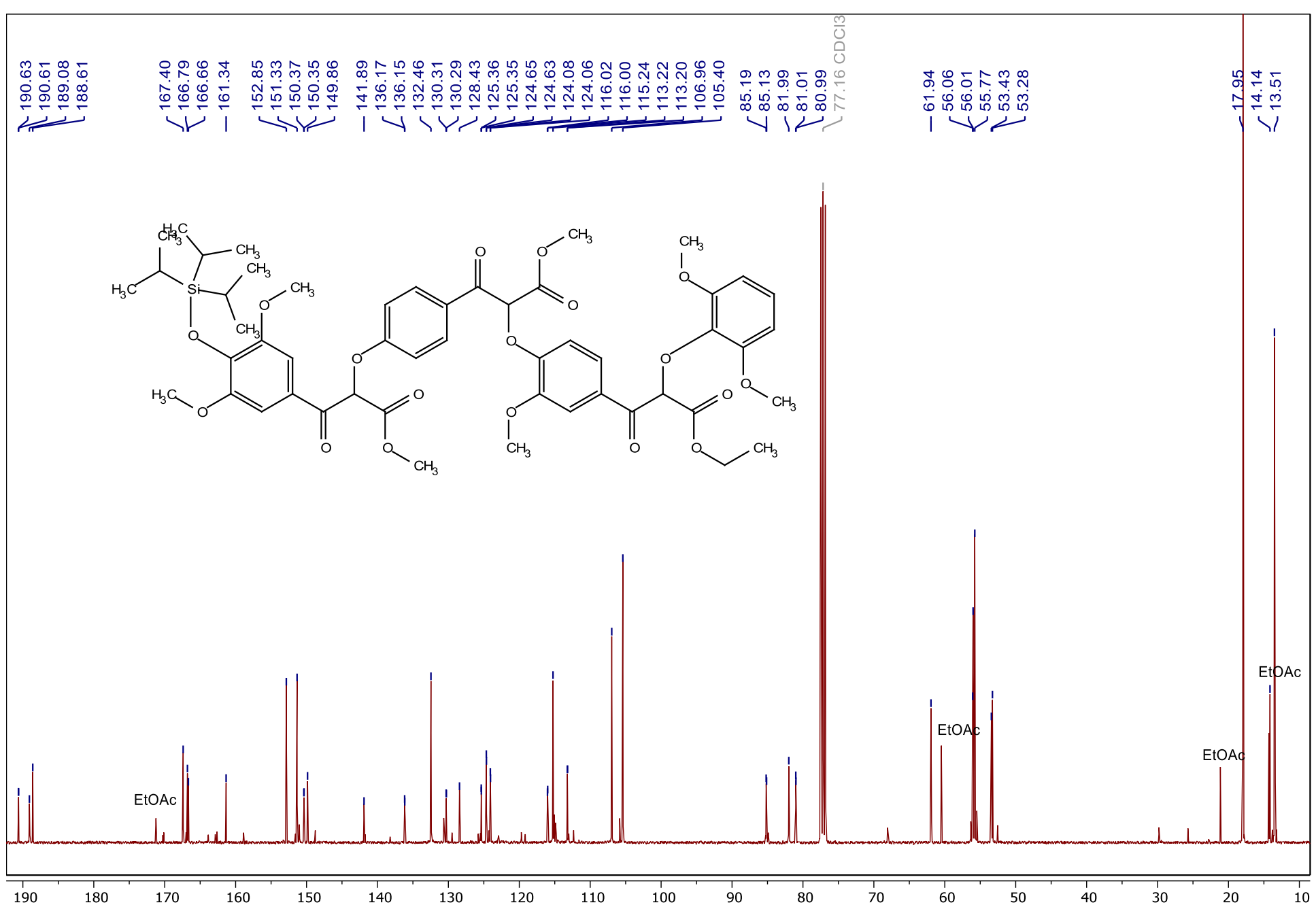

Figure S56. ^{13}C NMR (101 MHz, $CDCl_3$) spectrum of **5-TIP**

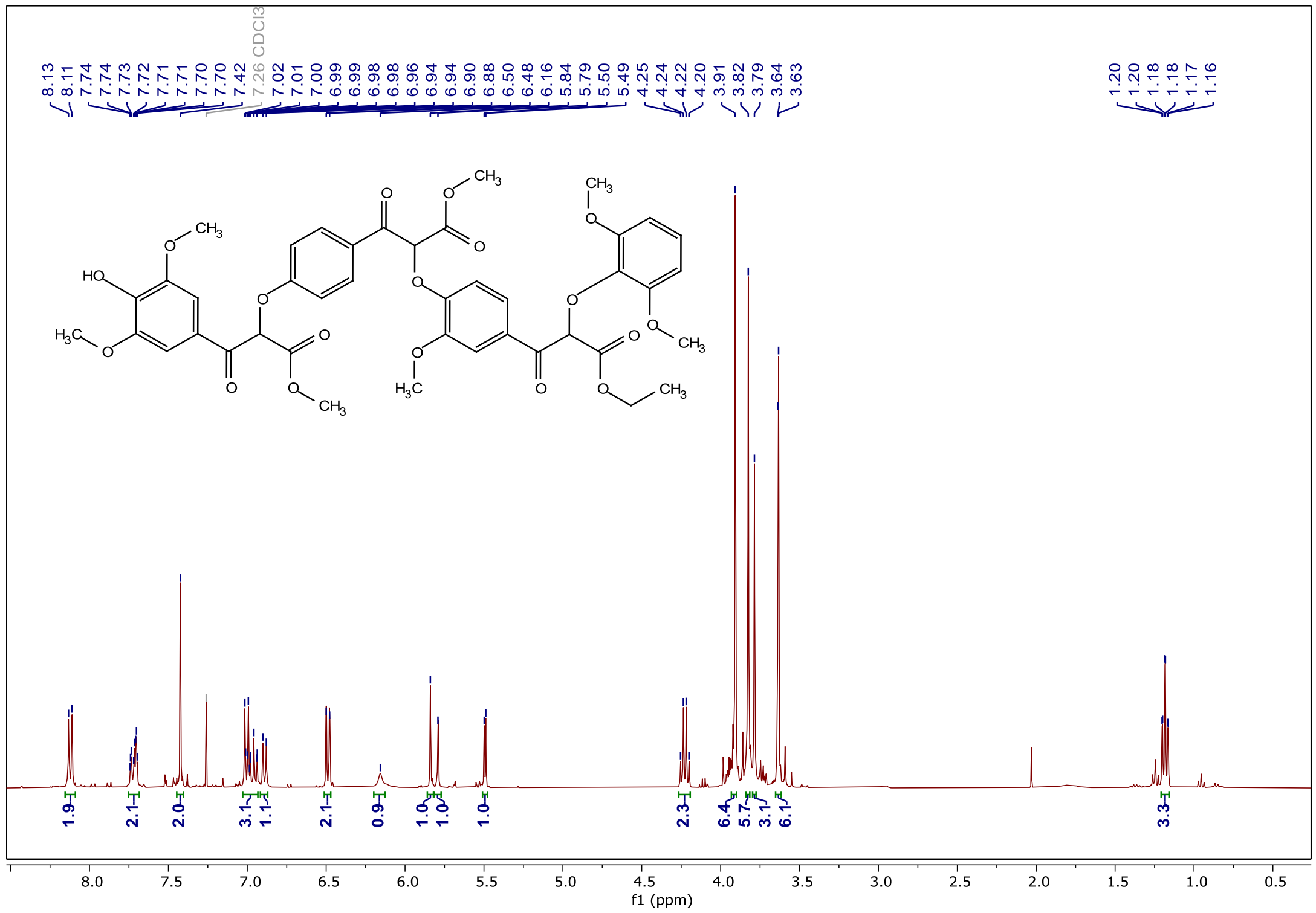

Figure S57. ^{1}H NMR (400 MHz, $CDCl_3$) spectrum of **5**.

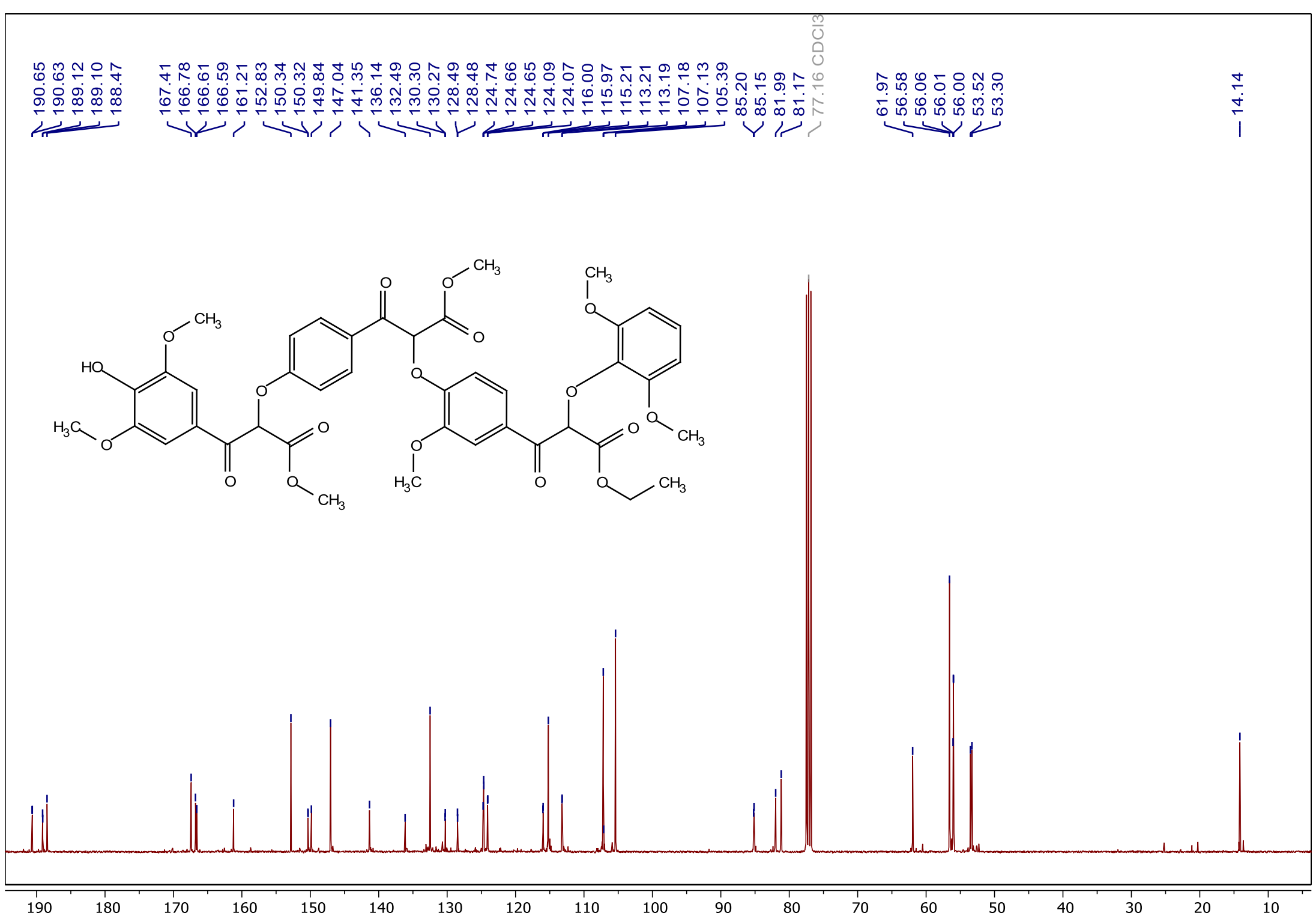

Figure S58. ^{13}C NMR (101 MHz, $CDCl_3$) spectrum of compound **5**.

Synthesis Oligomers with β-5 Linkage:

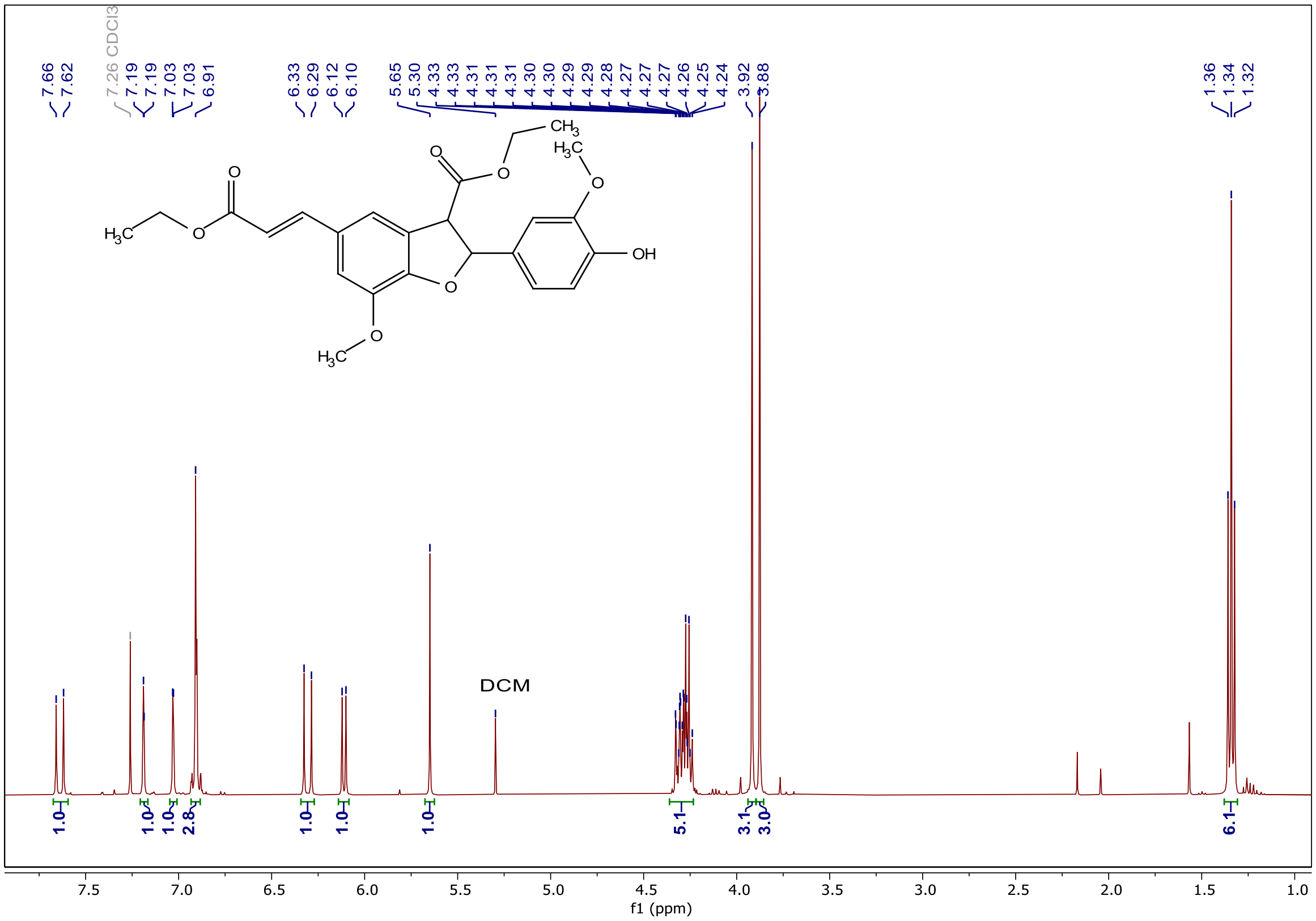

Figure S59. ^{1}H NMR (400 MHz, $CDCl_3$) spectrum of compound **6.**

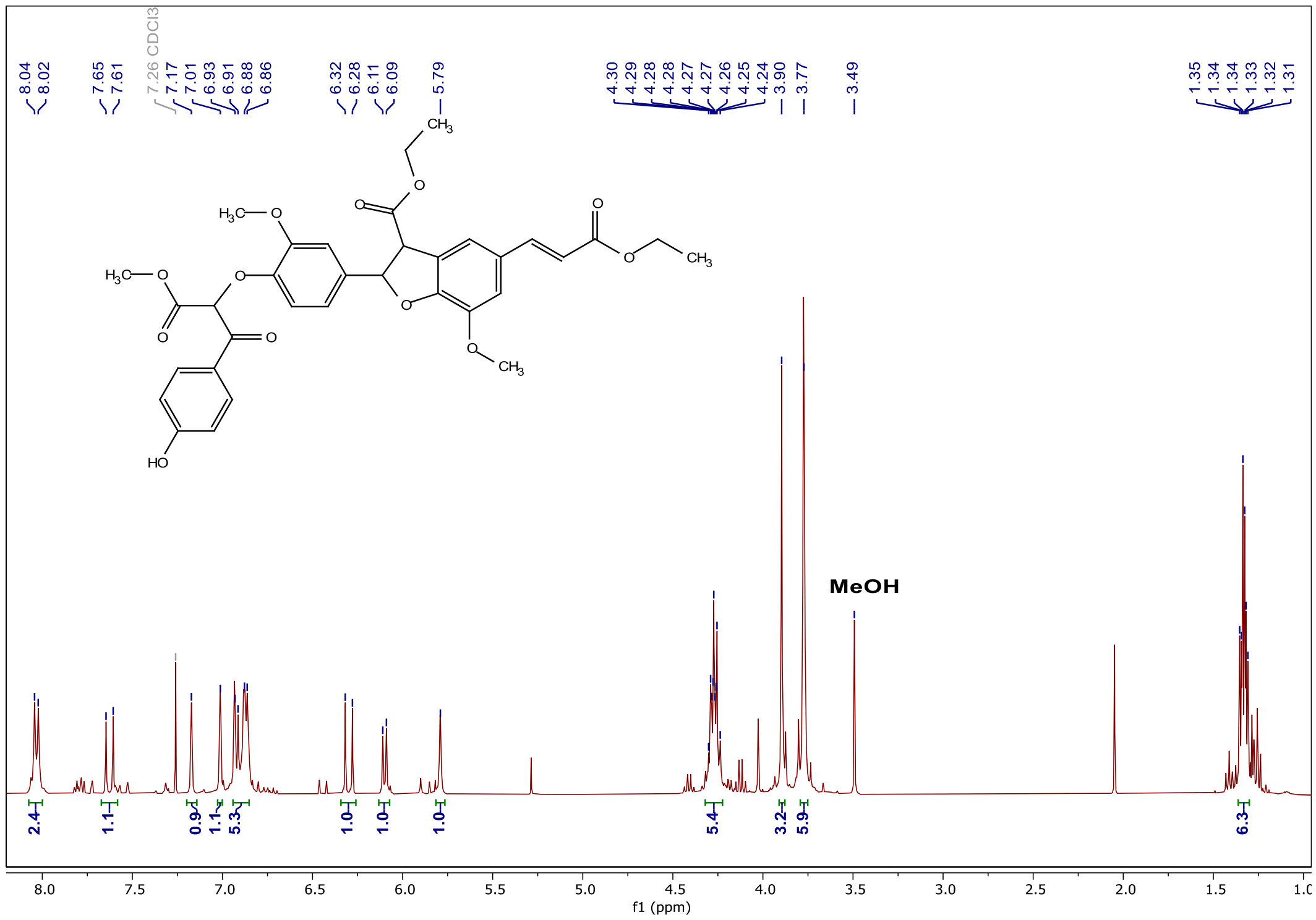

Figure S60. ^{1}H NMR (400 MHz, $CDCl_3$) spectrum of **7a.**

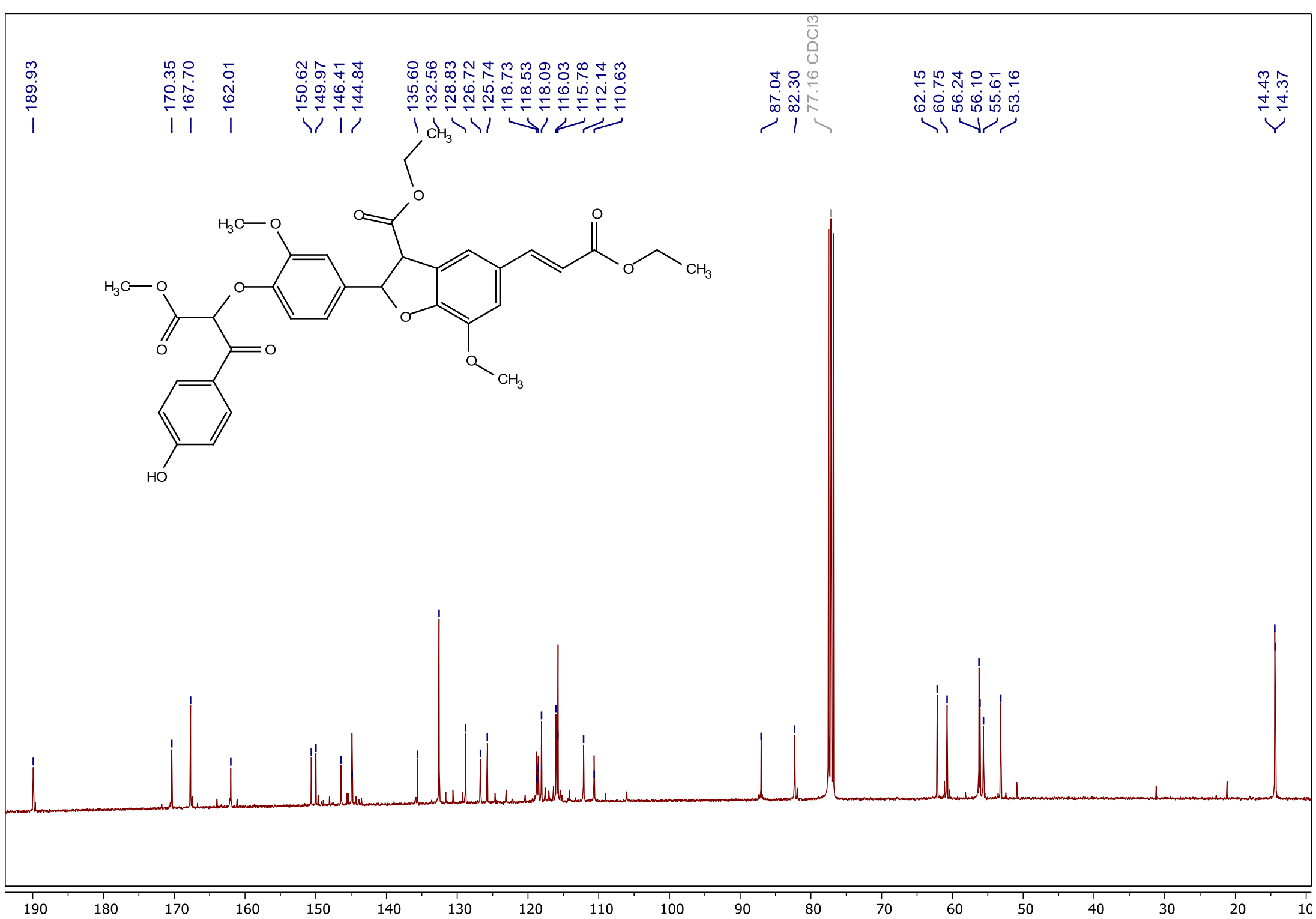

Figure S61. ^{13}C NMR (101 MHz, $CDCl_3$) spectrum of **7a.**

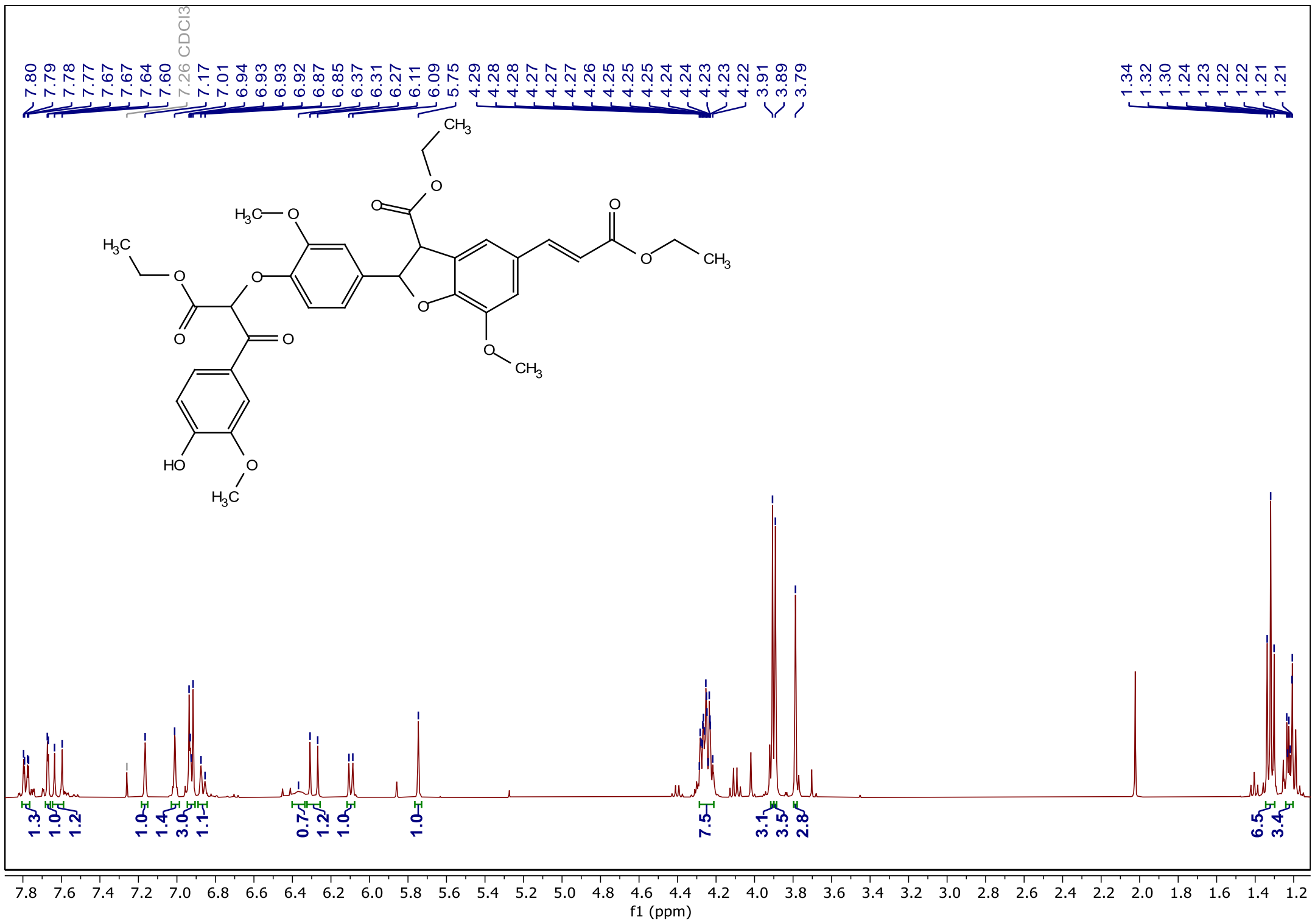

Figure S62. ^{1}H NMR (400 MHz, $CDCl_3$) spectrum of **7b.**

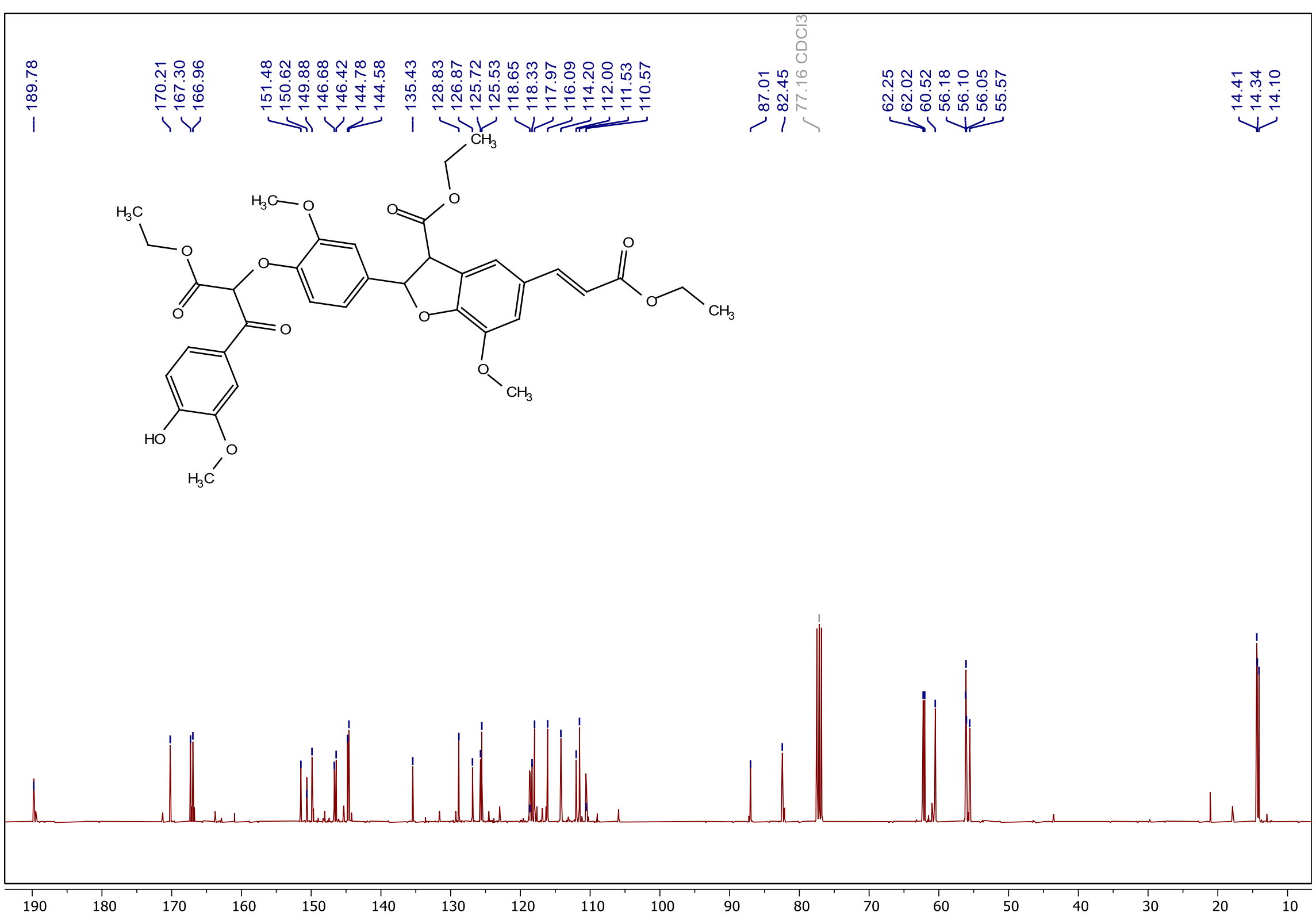

Figure S63. ^{13}C NMR (101 MHz, $CDCl_3$) spectrum of **7b.**

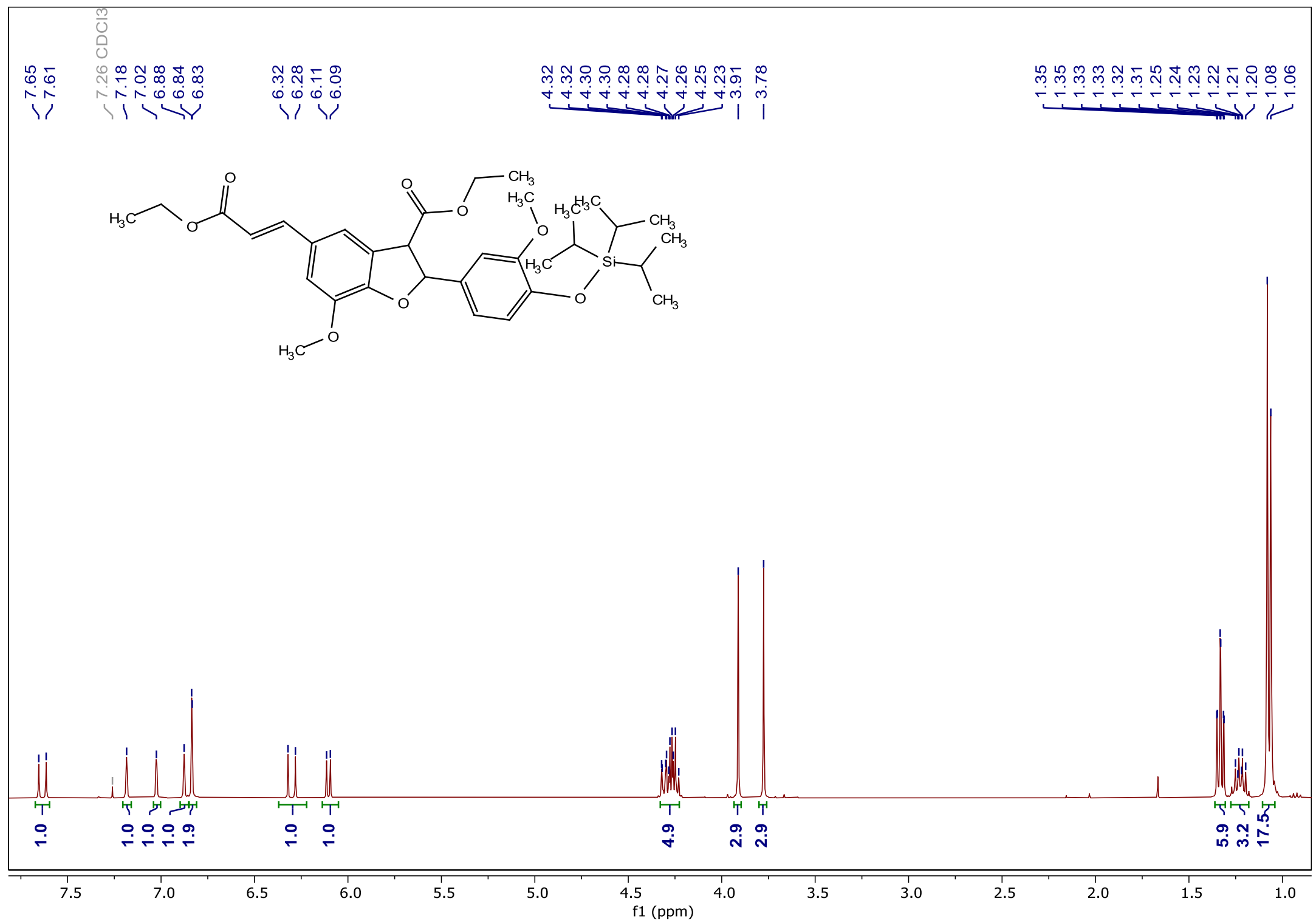

Figure S64. ^{1}H NMR (400 MHz, $CDCl_3$) spectrum of **6-TIPS.**

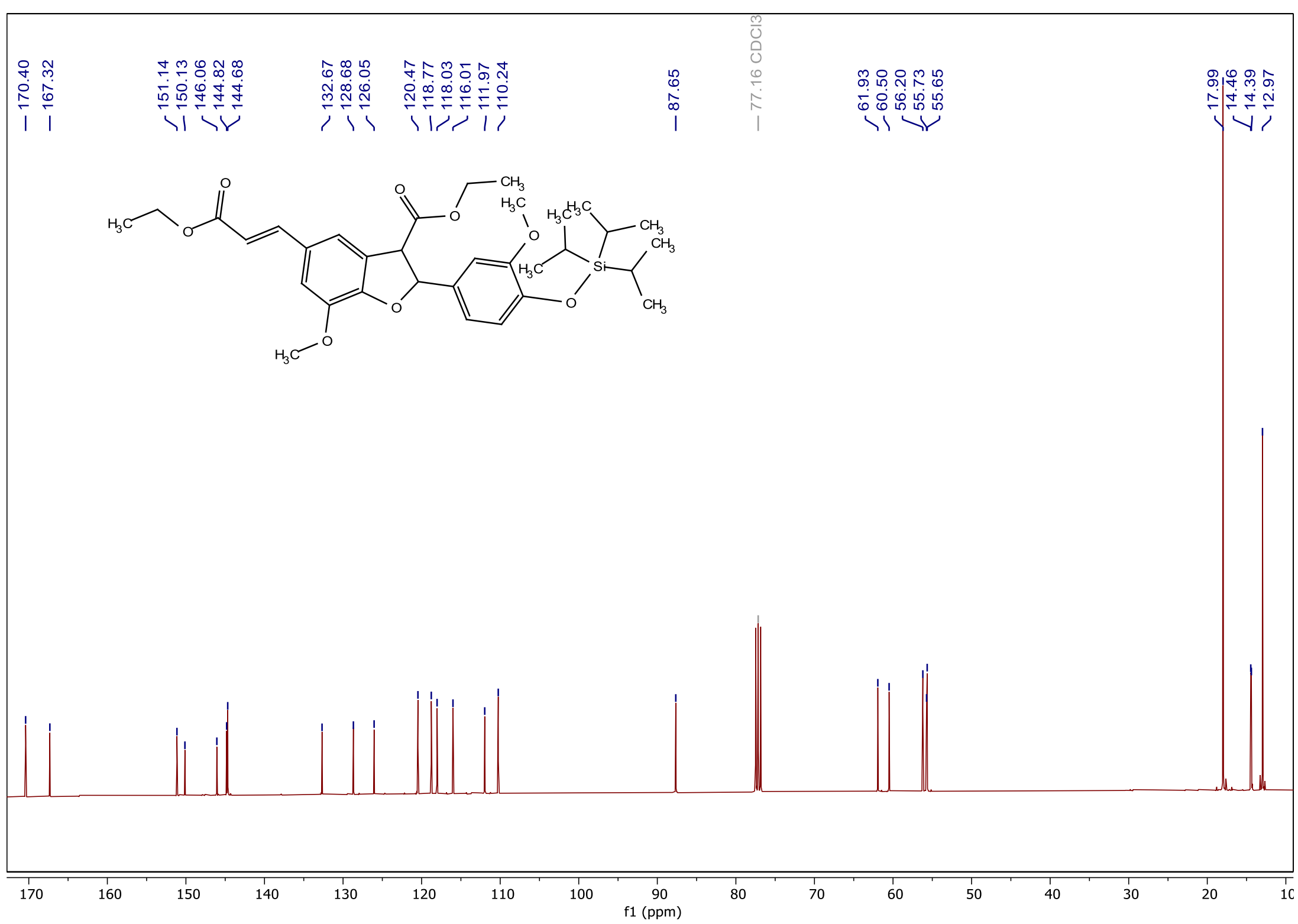

Figure S65. ^{13}C NMR (101 MHz, $CDCl_3$) spectrum of **6-TIPS.**

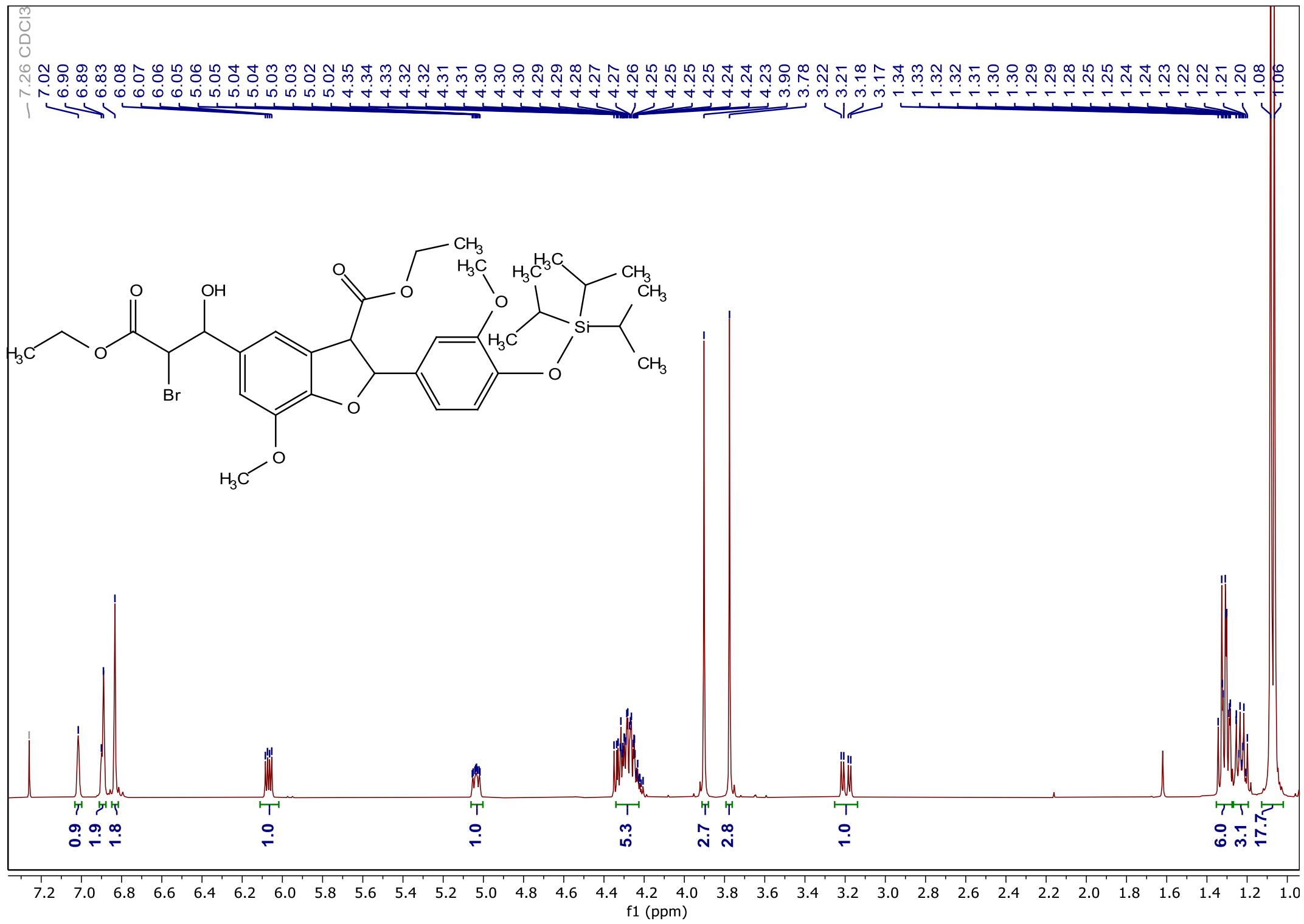

Figure S66. ^{1}H NMR (400 MHz, $CDCl_3$) spectrum of **6-TIPS-Br-OH.**

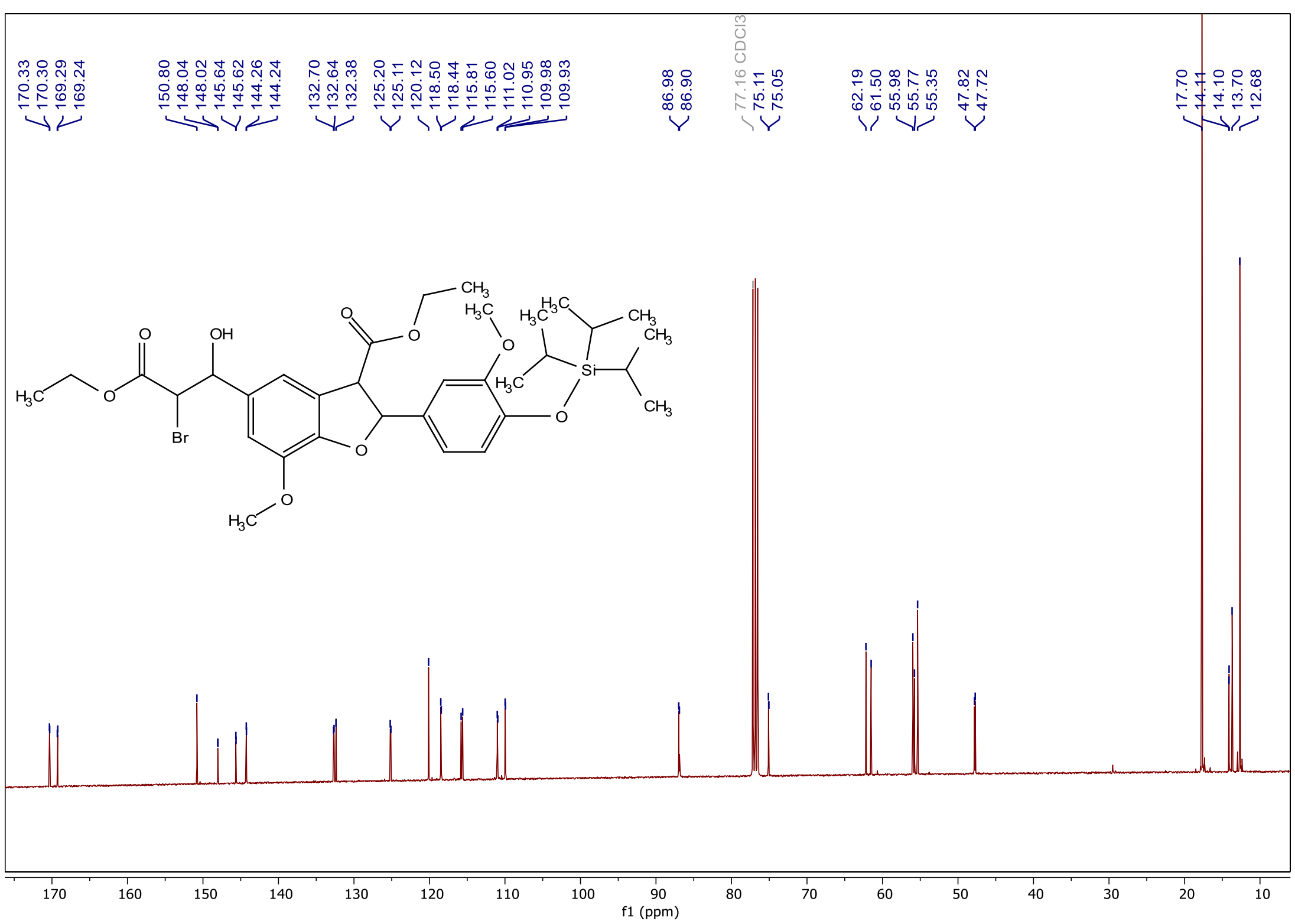

Figure S67. ^{13}C NMR (101 MHz, $CDCl_3$) spectrum of **6-TIPS-Br-OH.**

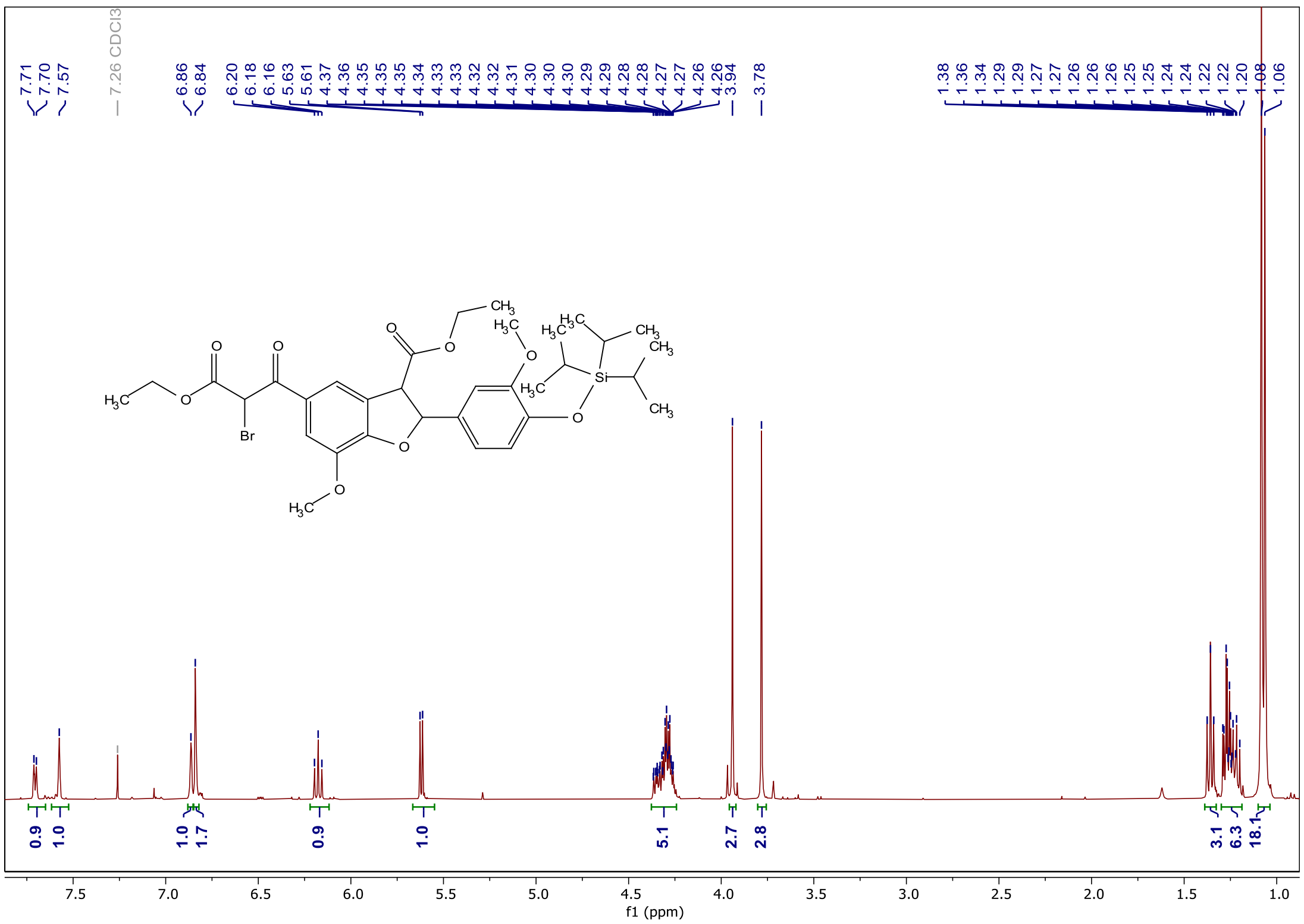

Figure S68. ^{1}H NMR (400 MHz, $CDCl_3$) spectrum of **8.**

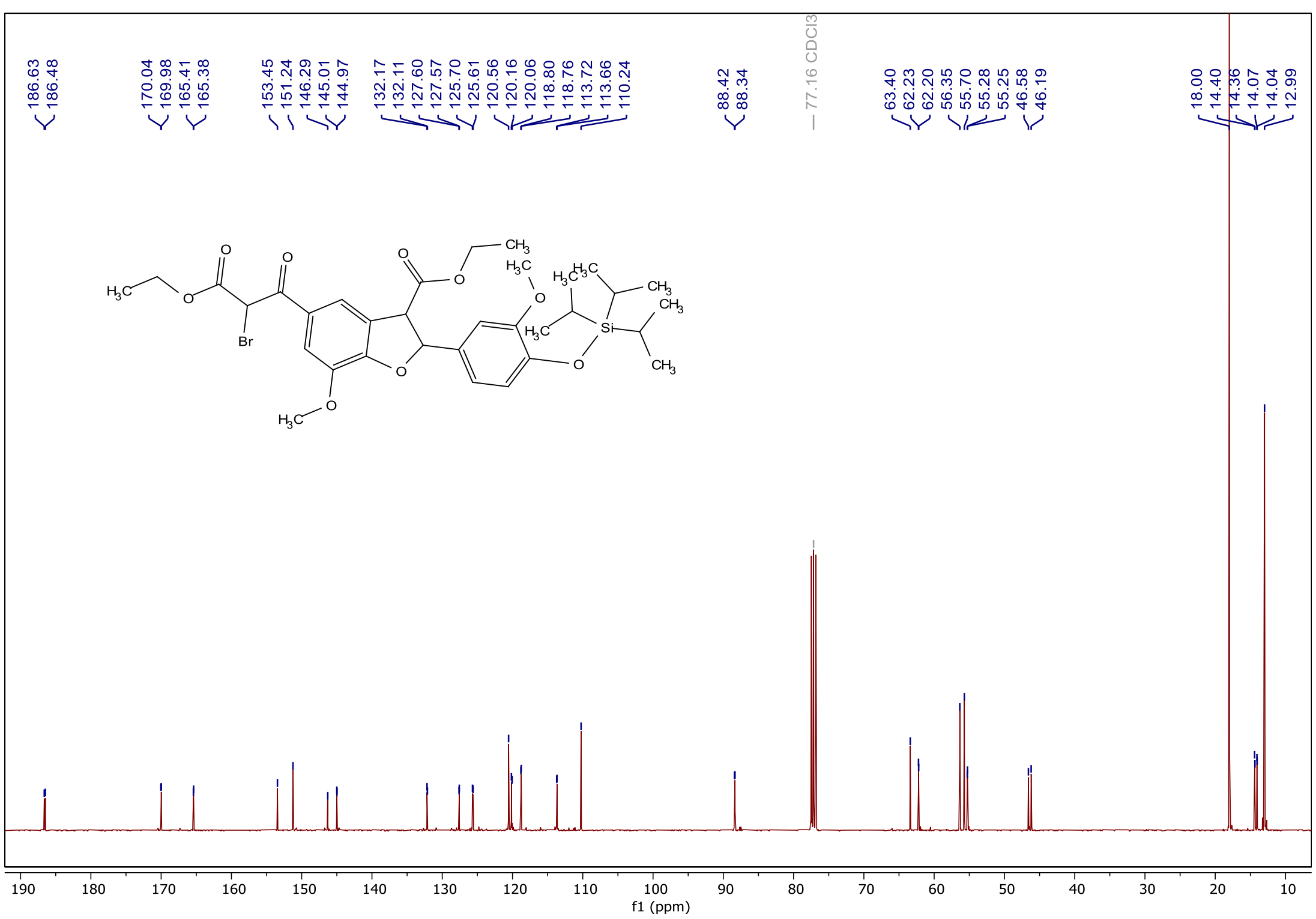

Figure S69. ^{13}C NMR (101 MHz, $CDCl_3$) spectrum of **8.**

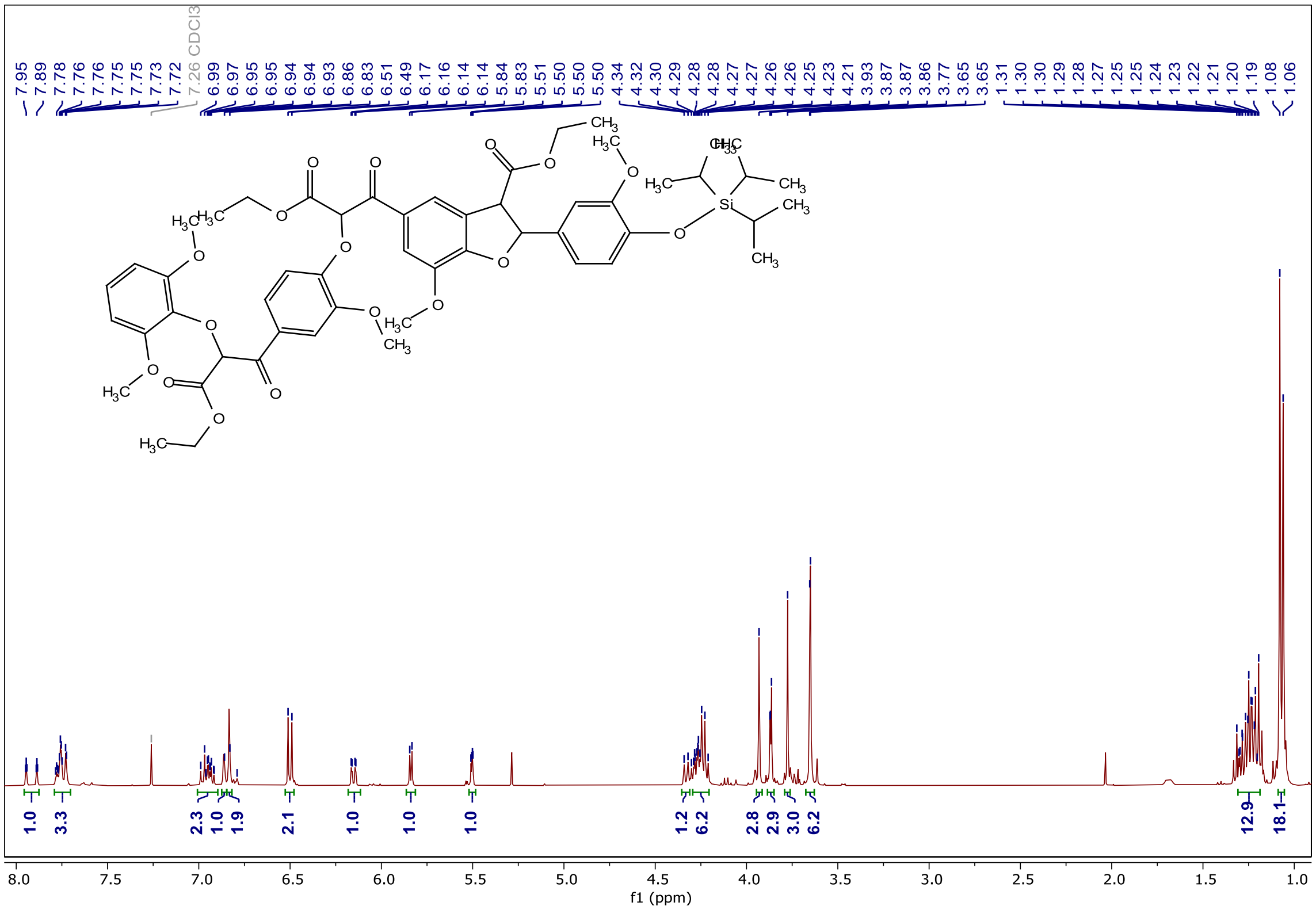

Figure S70. ^{1}H NMR (400 MHz, $CDCl_3$) spectrum of **9-TIPS.**

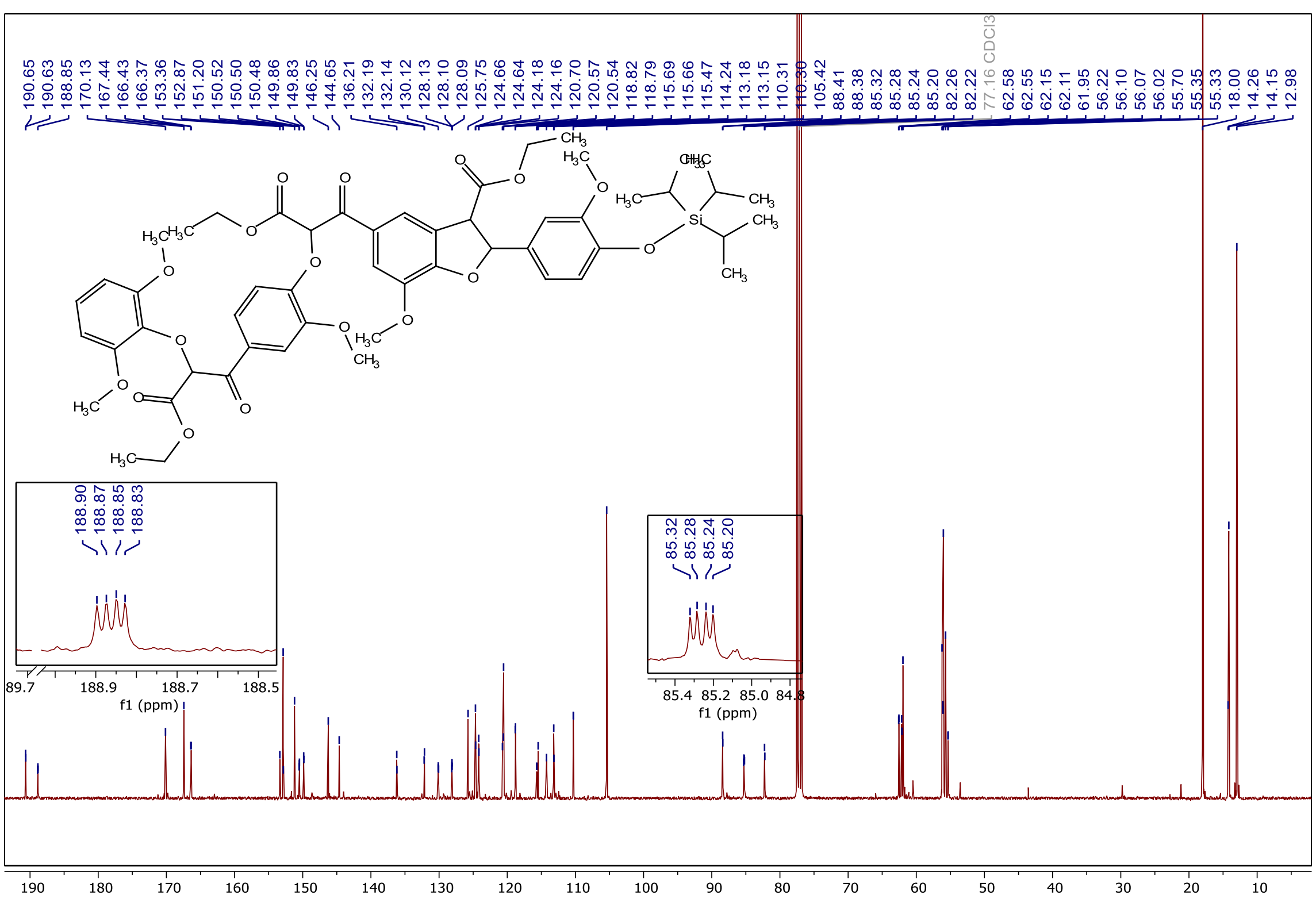

Figure S71. ^{13}C NMR (101 MHz, $CDCl_3$) spectrum of **9-TIPS.**

NMR Spectra of LMCs:

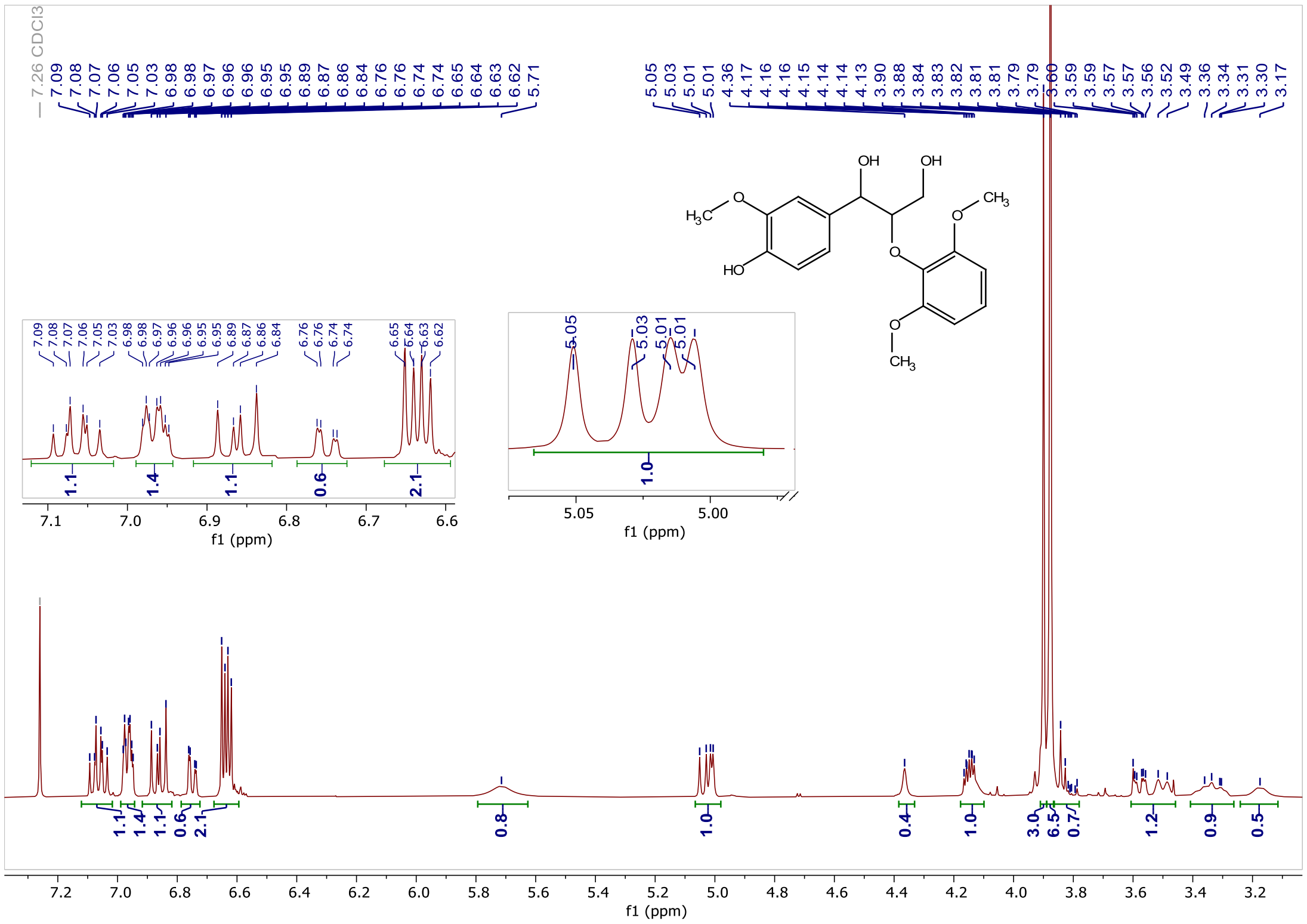

Figure S72. ^{1}H NMR (400 MHz, $CDCl_3$) spectrum of **LMC1**.

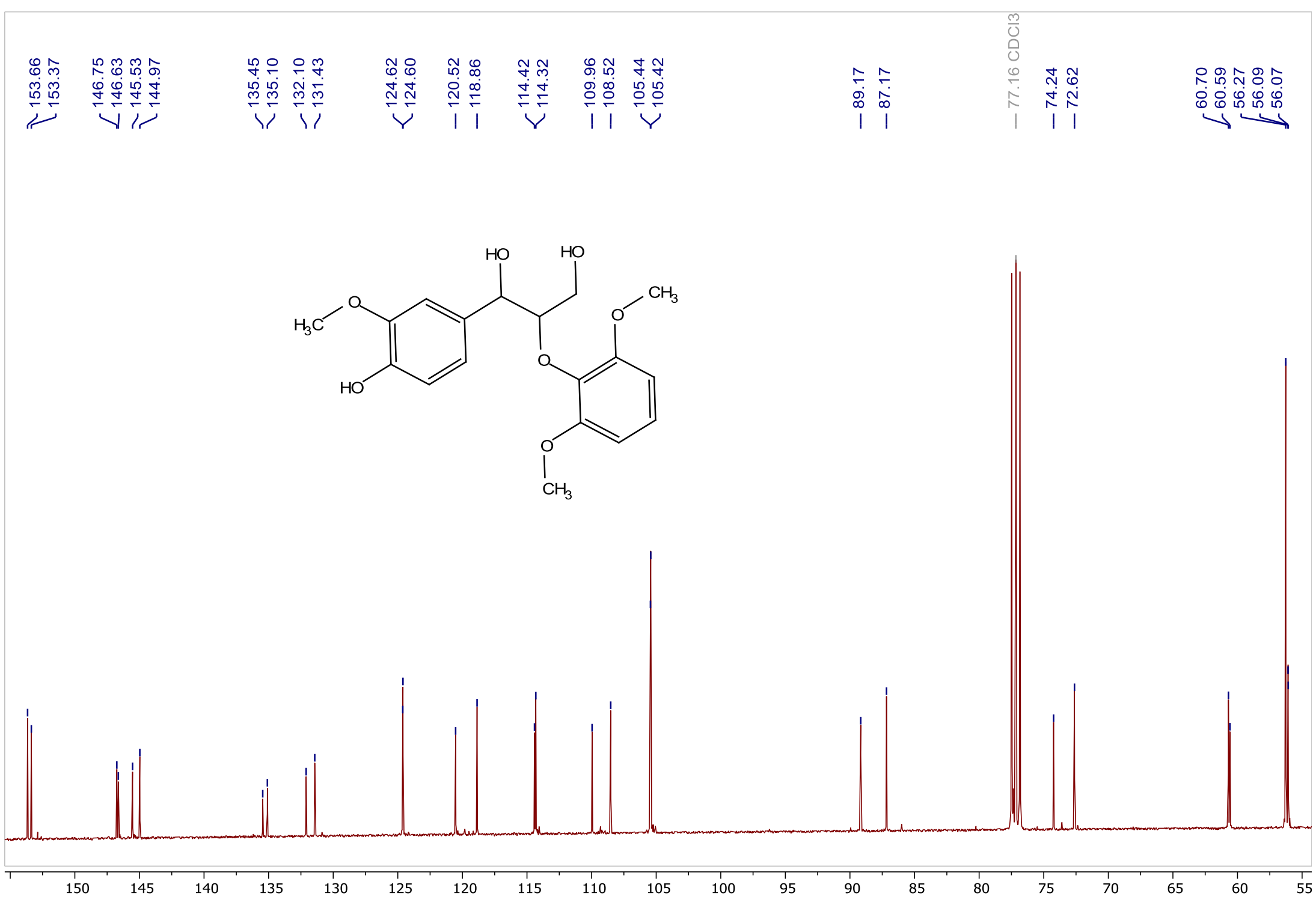

Figure S73. ^{13}C NMR (101 MHz, $CDCl_3$) spectrum of compound **LMC1**.

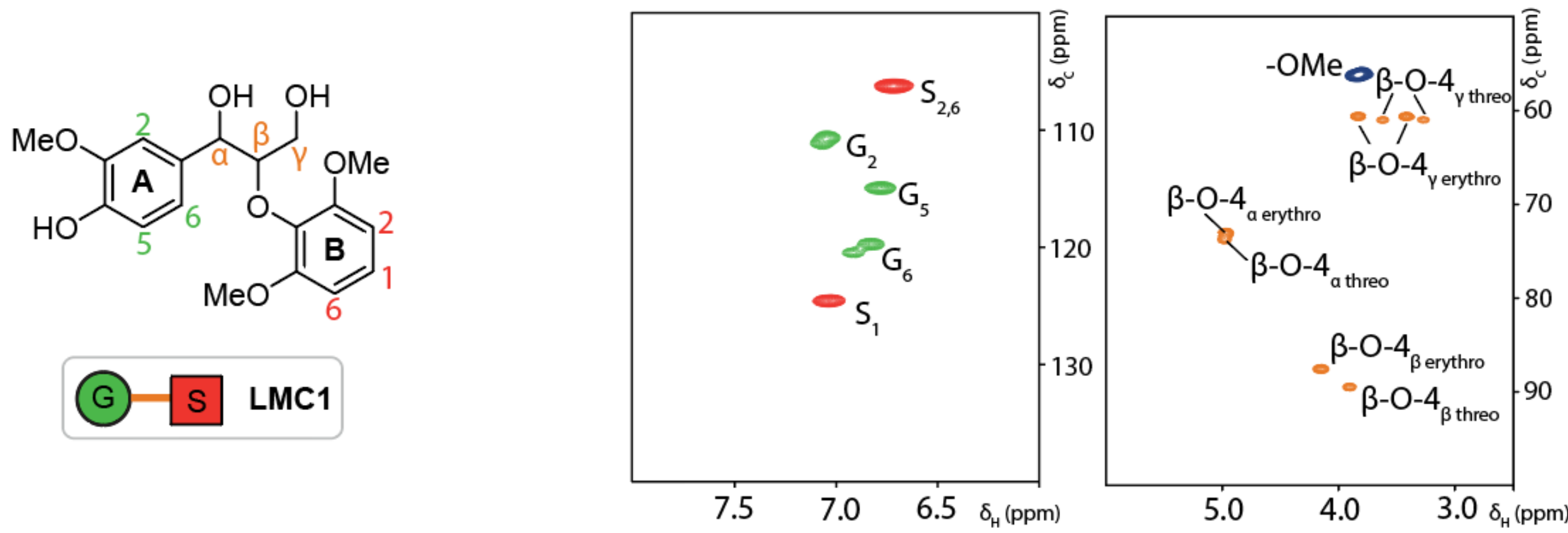

Figure S74. Aromatic and aliphatic regions of ^{1}H-^{13}C HSQC NMR spectra of **LMC1**.

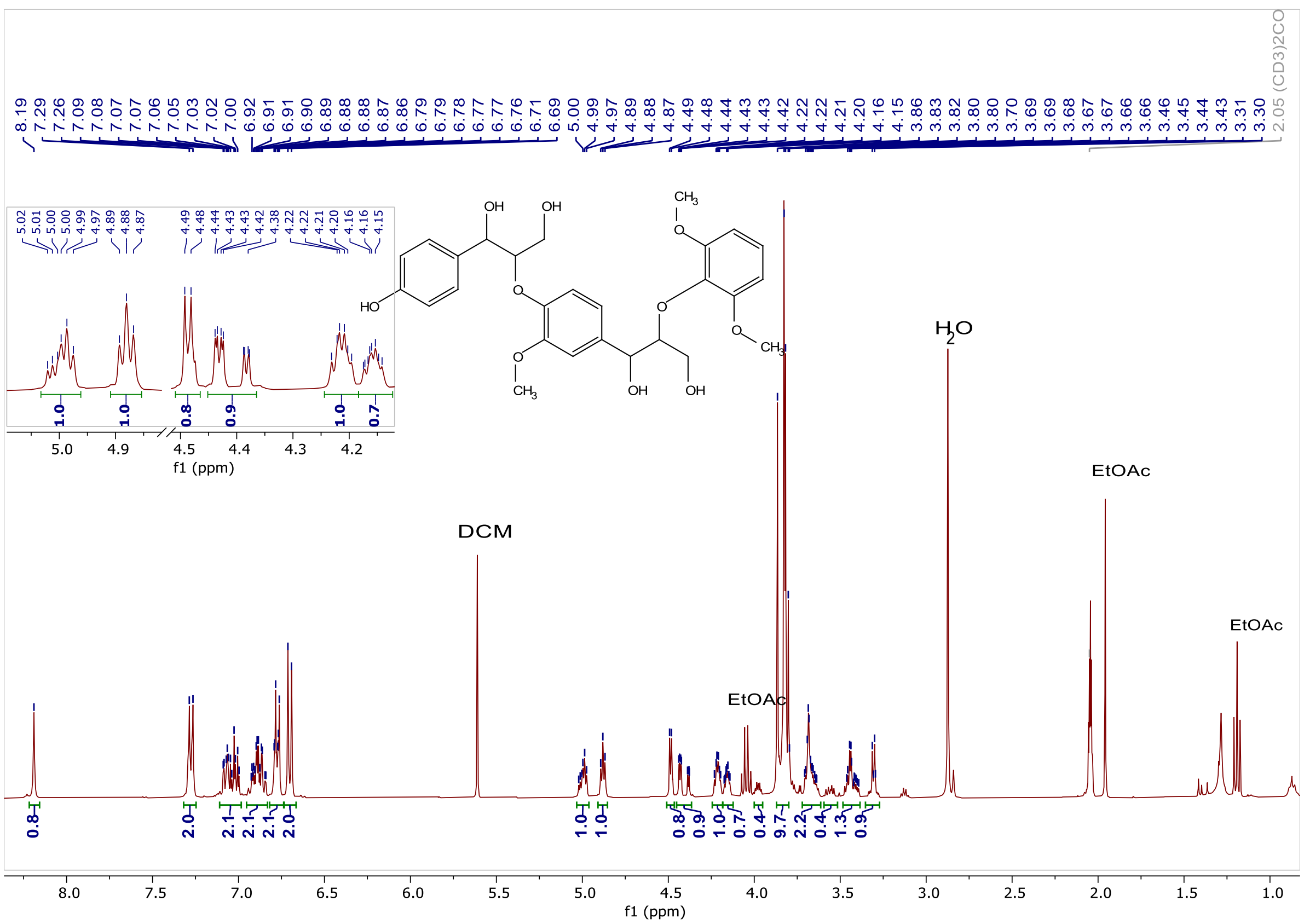

Figure S75. ^{1}H NMR (400 MHz, acetone-d_6) spectrum of **LMC2**.

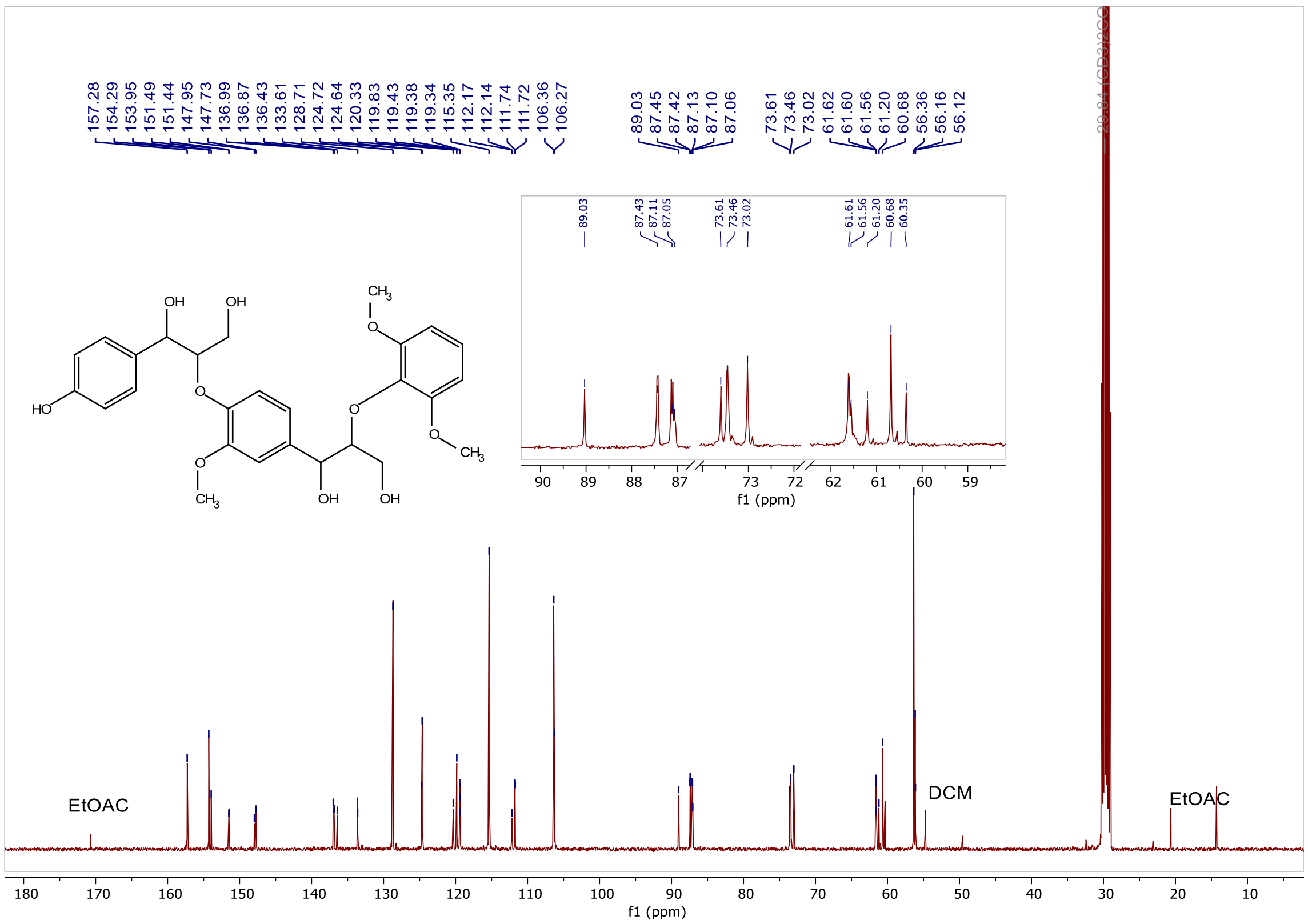

Figure S76. ^{13}C NMR (101 MHz, acetone-d$_6$) spectrum of compound **LMC2**.

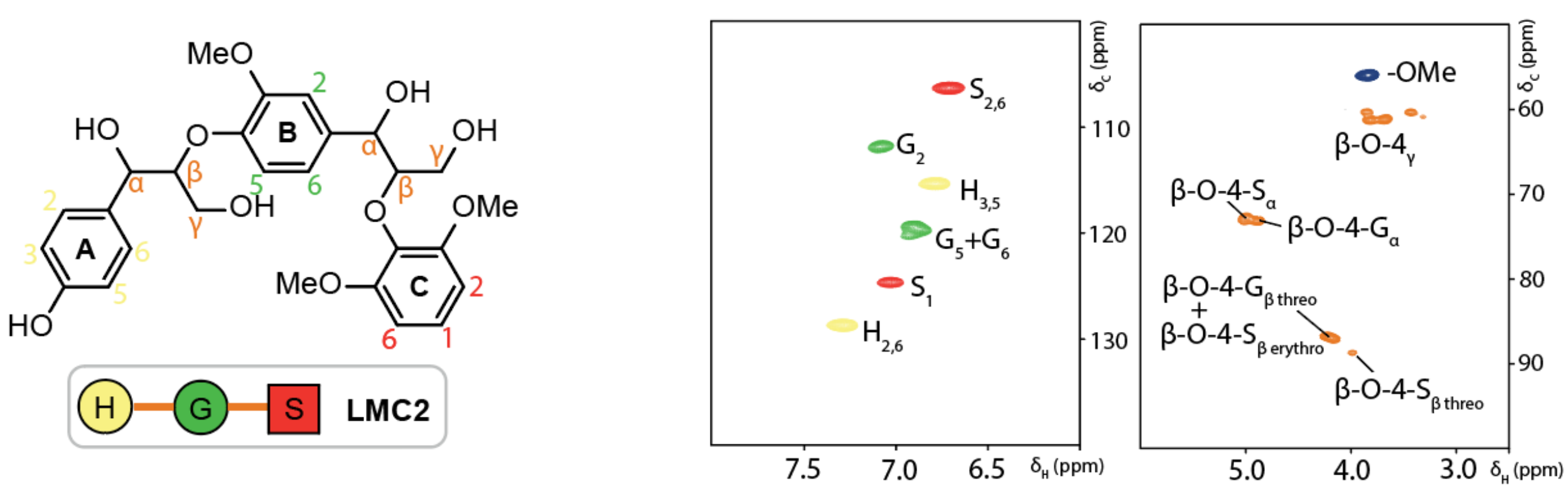

Figure S77. Aromatic and aliphatic regions of ^{1}H-^{13}C HSQC NMR spectra of **LMC2**. Also displayed in Figure 1 of the main manuscript.

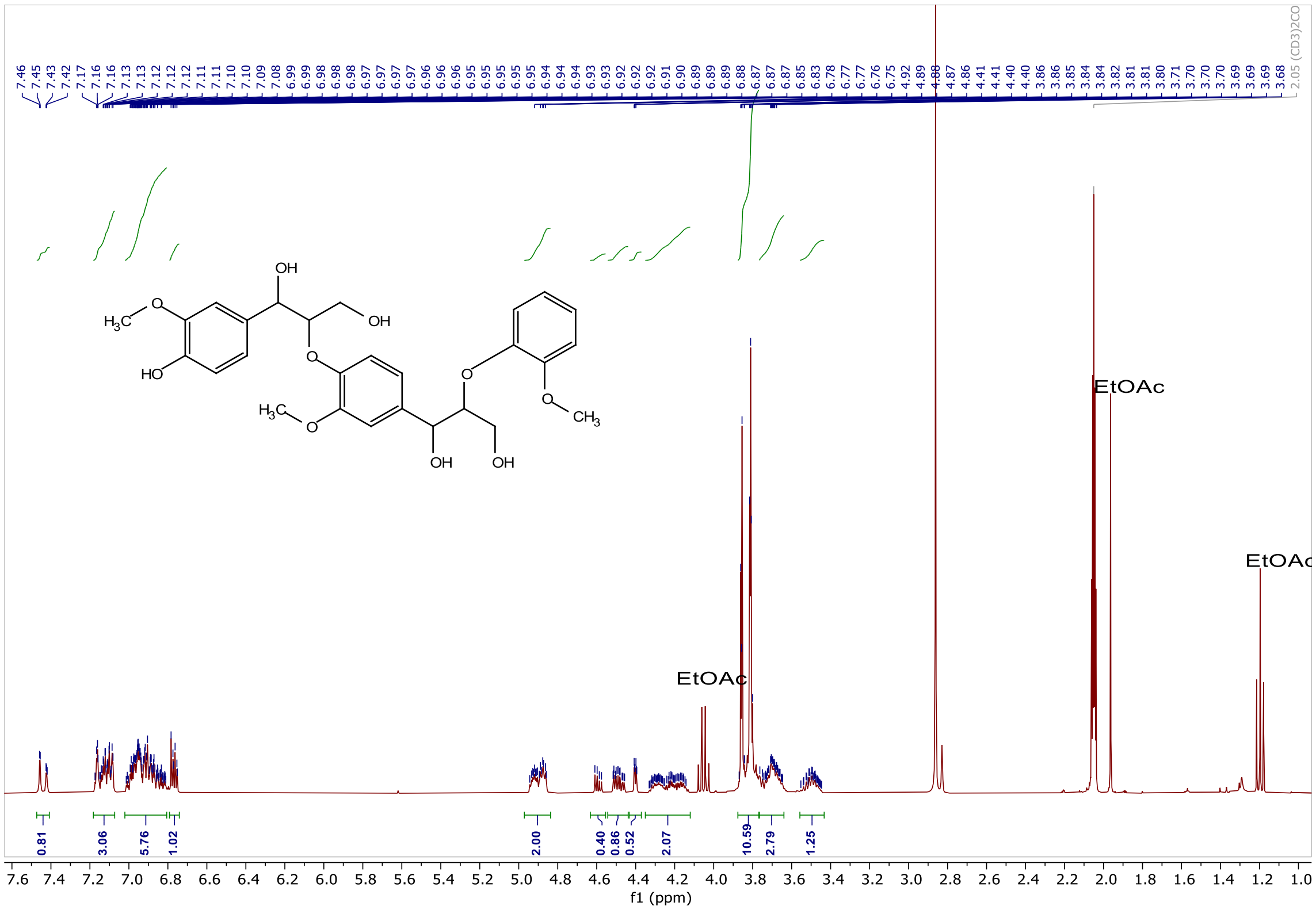

Figure S78. ^{1}H NMR (400 MHz, acetone-d$_6$) spectrum of **LMC3**.

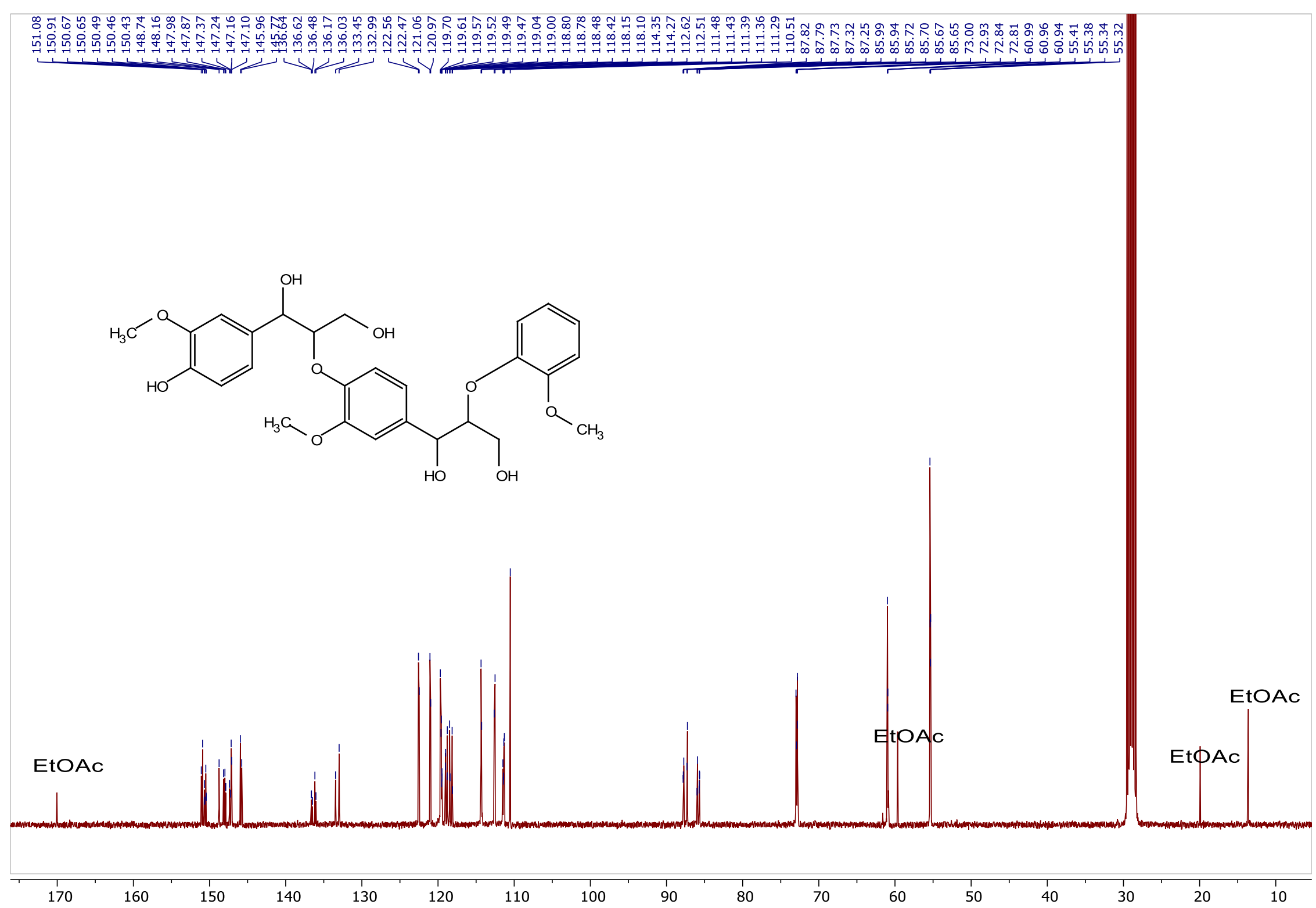

Figure S79. ^{13}C NMR (101 MHz, acetone-d$_6$) spectrum of **LMC3**.

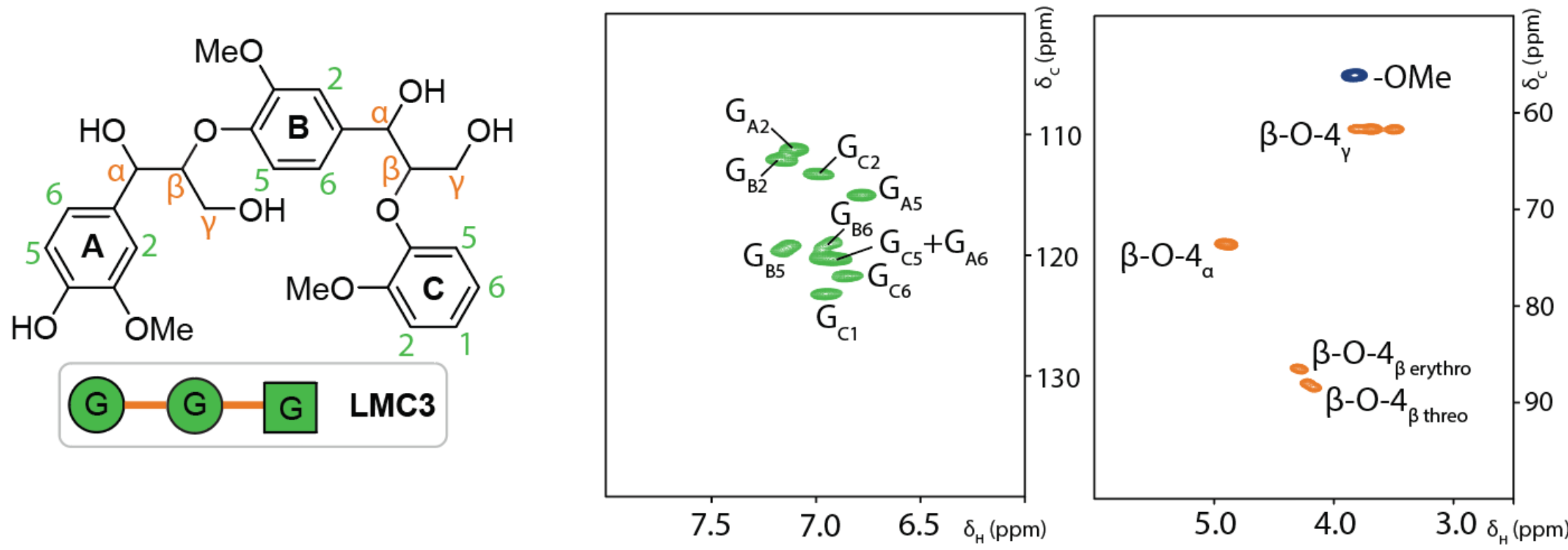

Figure S80. Aromatic and aliphatic regions of ^{1}H-^{13}C HSQC NMR spectra of **LMC3**.

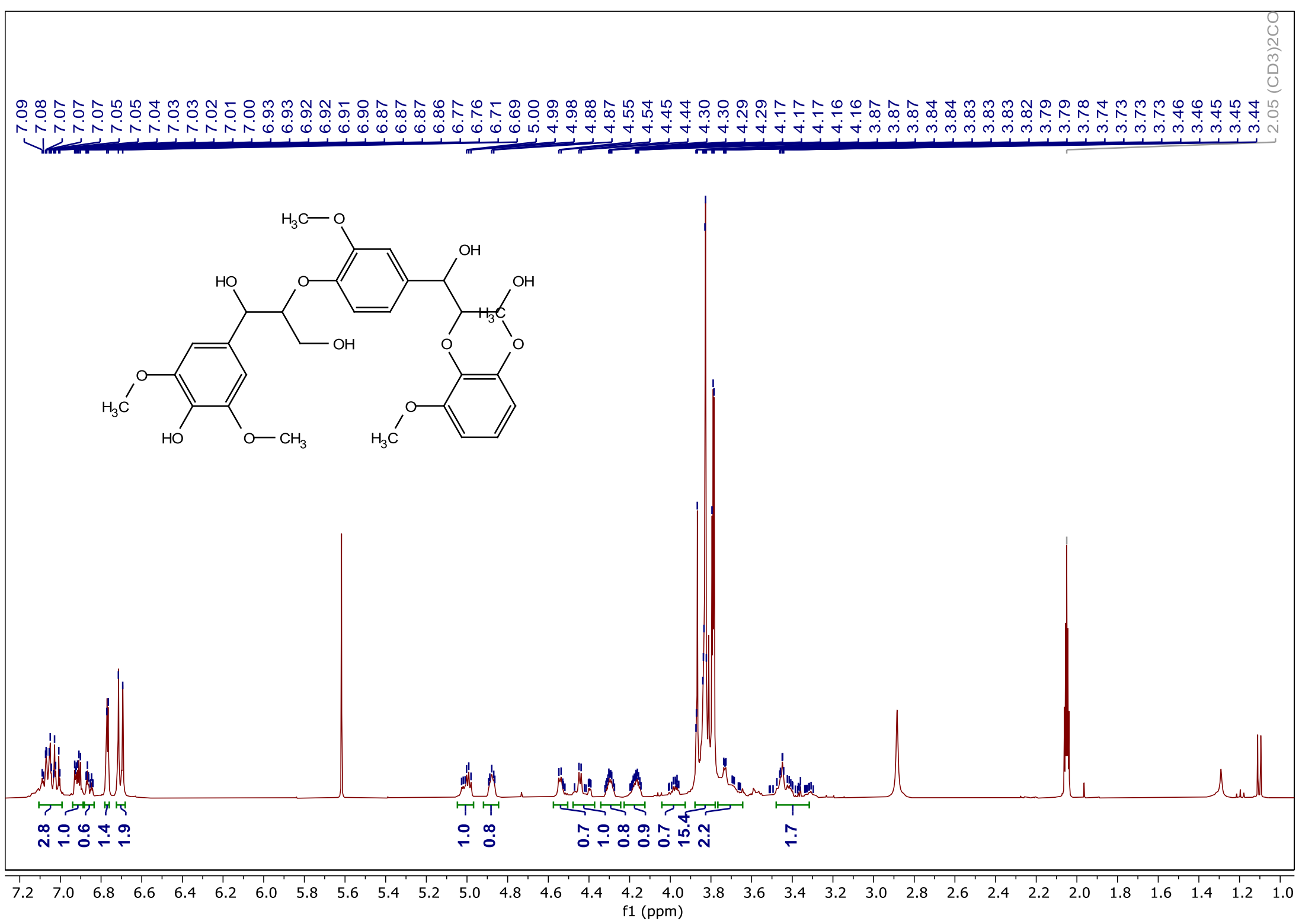

Figure S81. ^{1}H NMR (400 MHz, acetone-d_6) spectrum of **LMC4**.

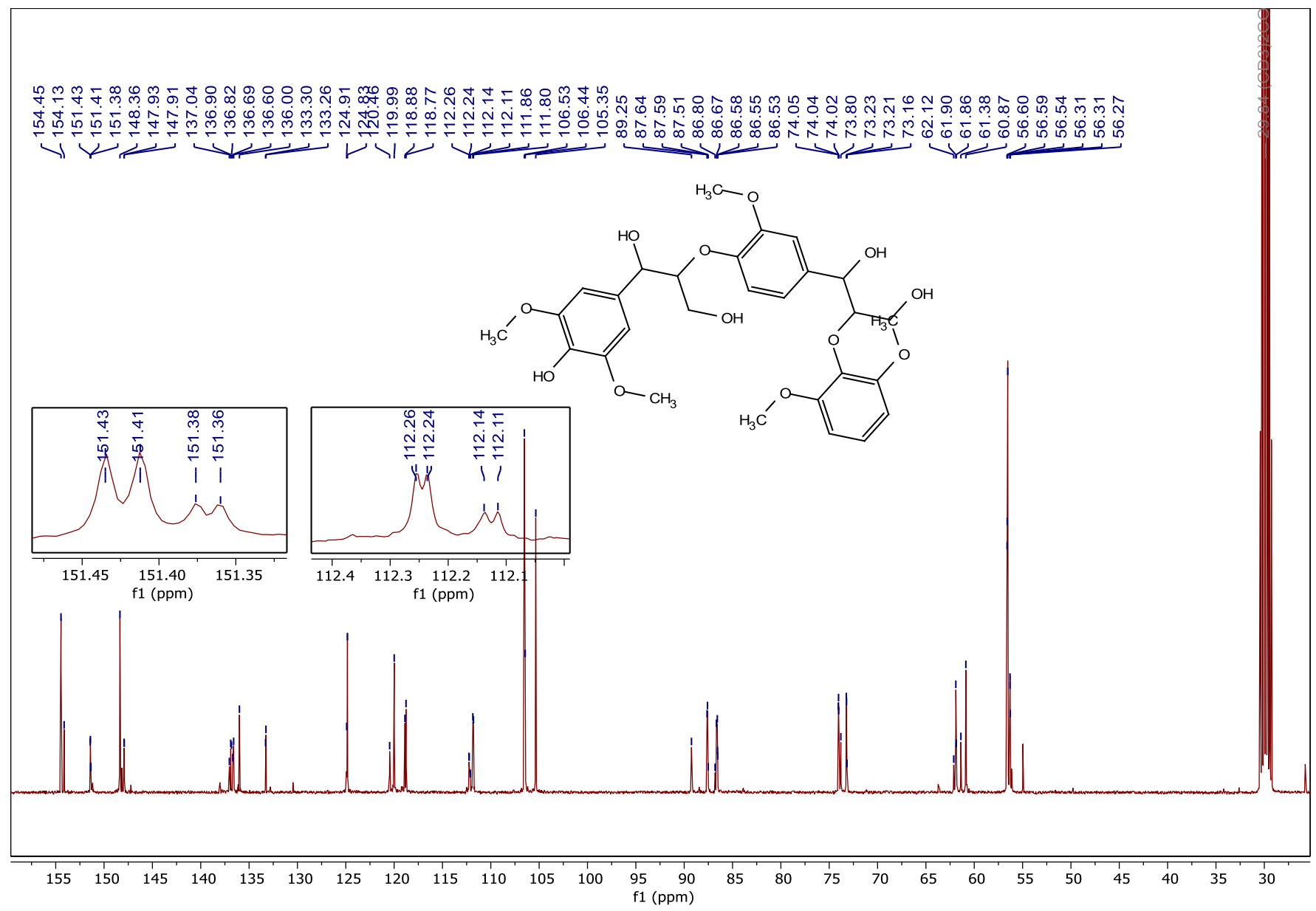

Figure S82. ^{13}C NMR (101 MHz, acetone-d_6) spectrum of **LMC4**.

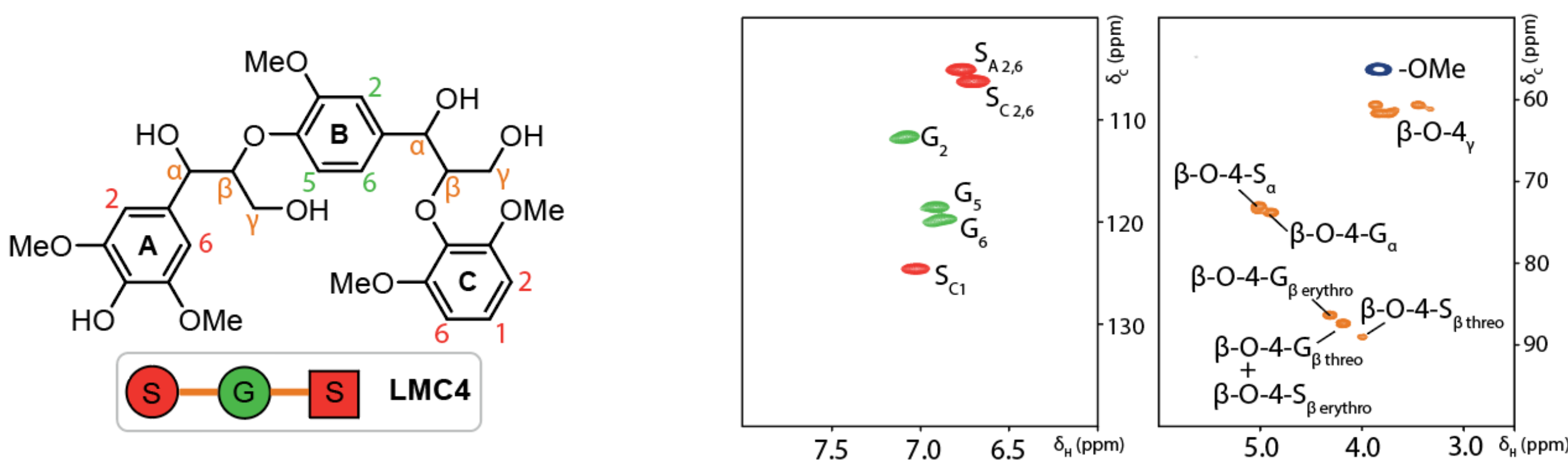

Figure S83. Aromatic and aliphatic regions of ^{1}H-^{13}C HSQC NMR spectra of **LMC4**. Also displayed in Figure 1 of the main manuscript.

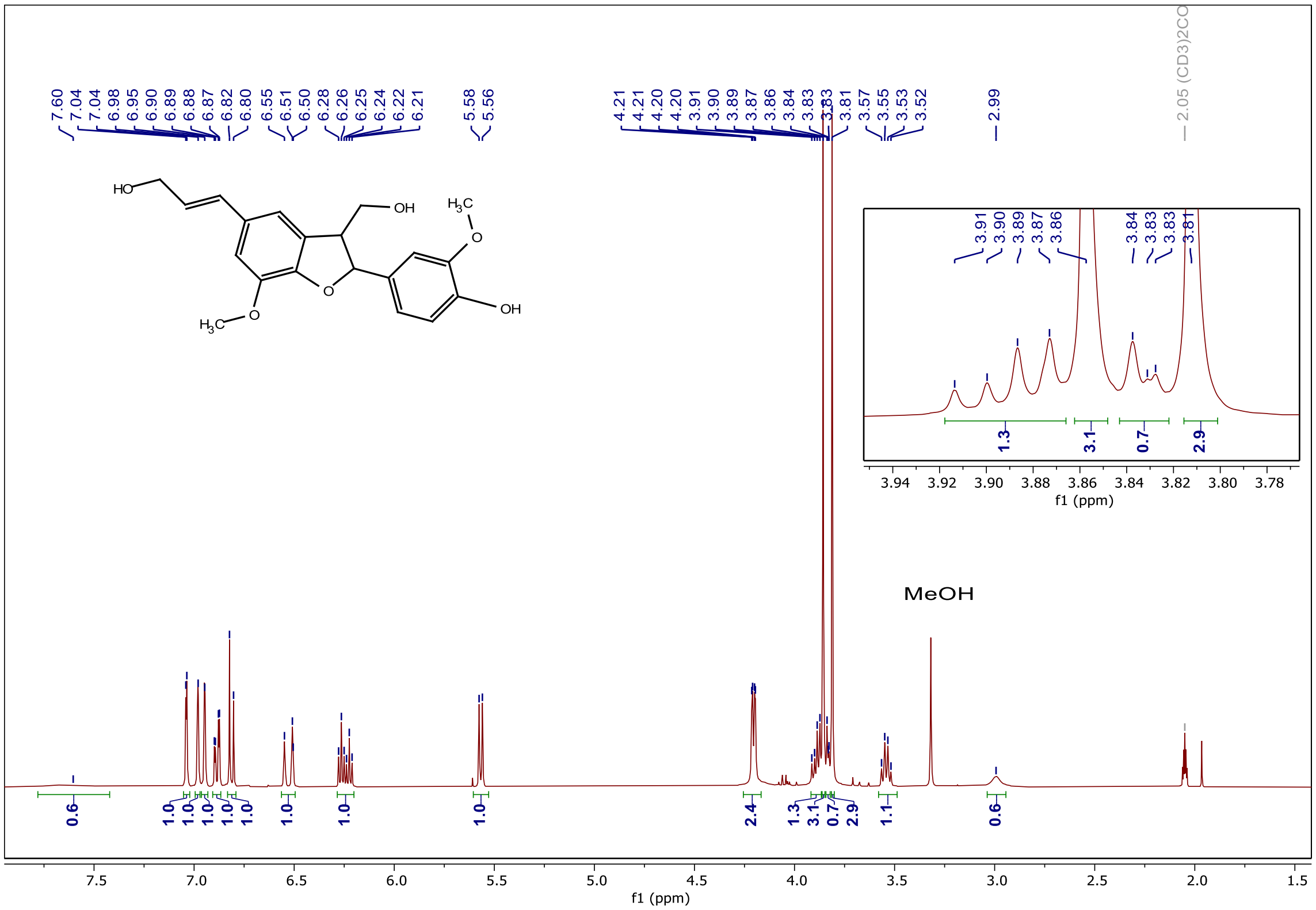

Figure S84. ^{1}H NMR (400 MHz, acetone-d$_6$) spectrum of **LMC5.**

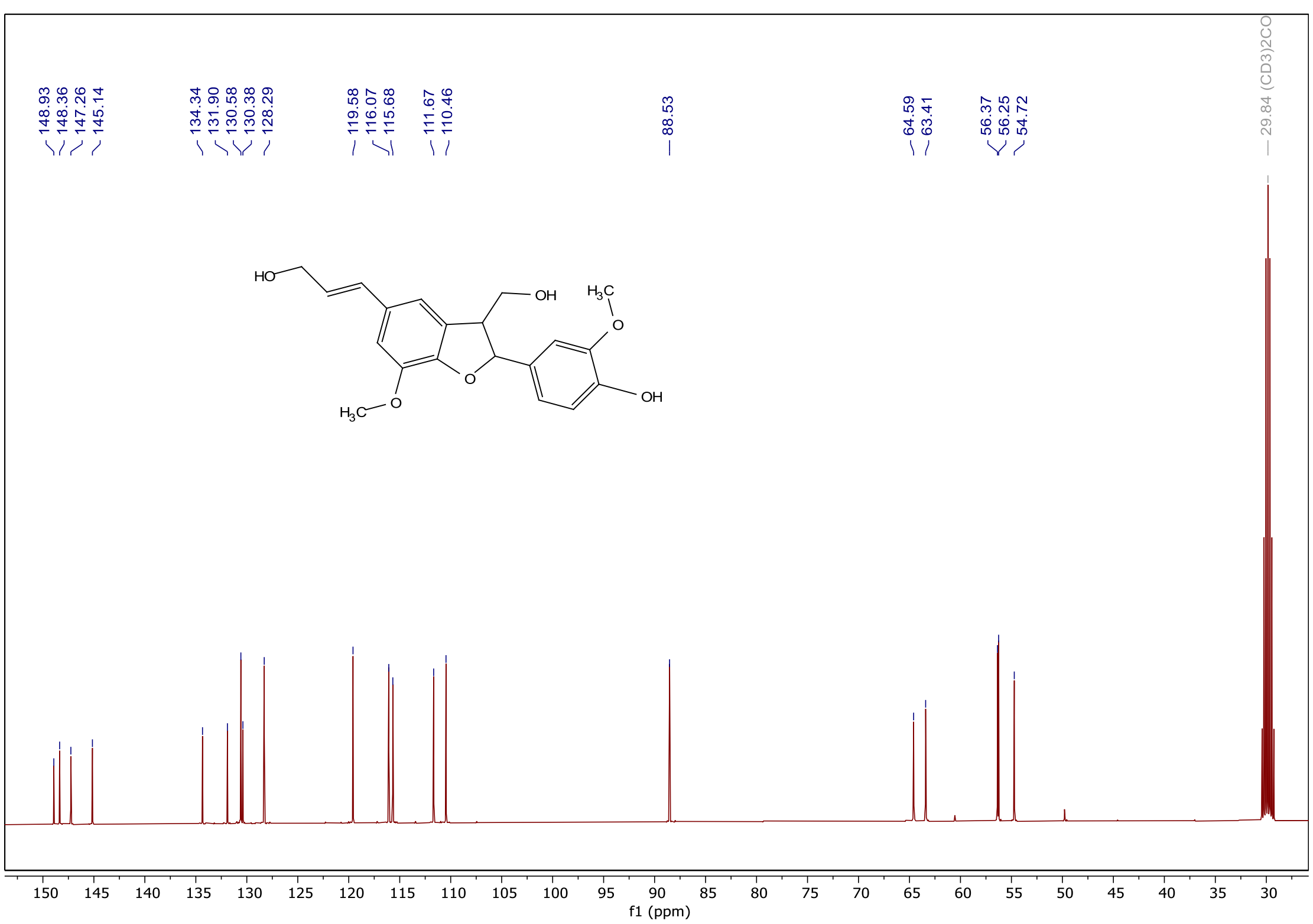

Figure S85. ^{13}C NMR (101 MHz, acetone-d$_6$) spectrum of **LMC5**.

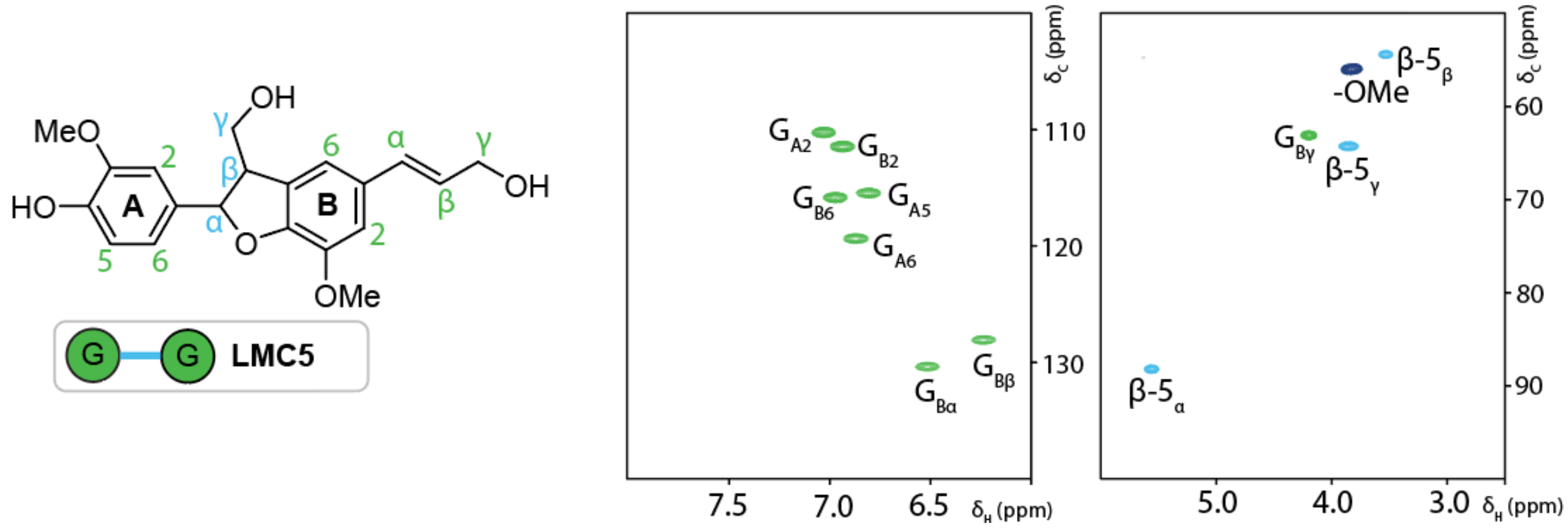

Figure S86. Aromatic and aliphatic regions of ^{1}H-^{13}C HSQC NMR spectra of **LMC5.**

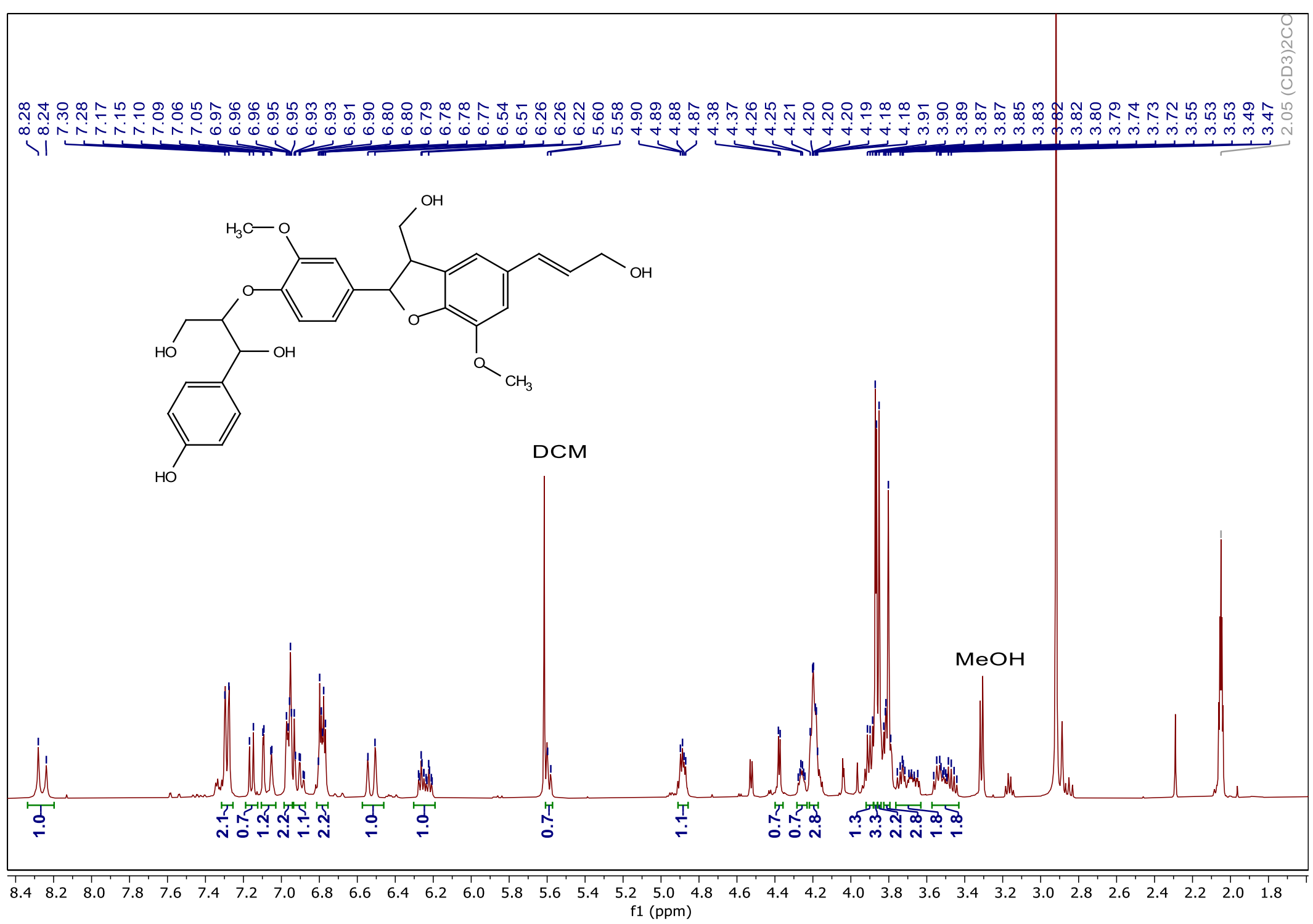

Figure S87. ^{1}H NMR (400 MHz, acetone-d_6) spectrum of **LMC6.**

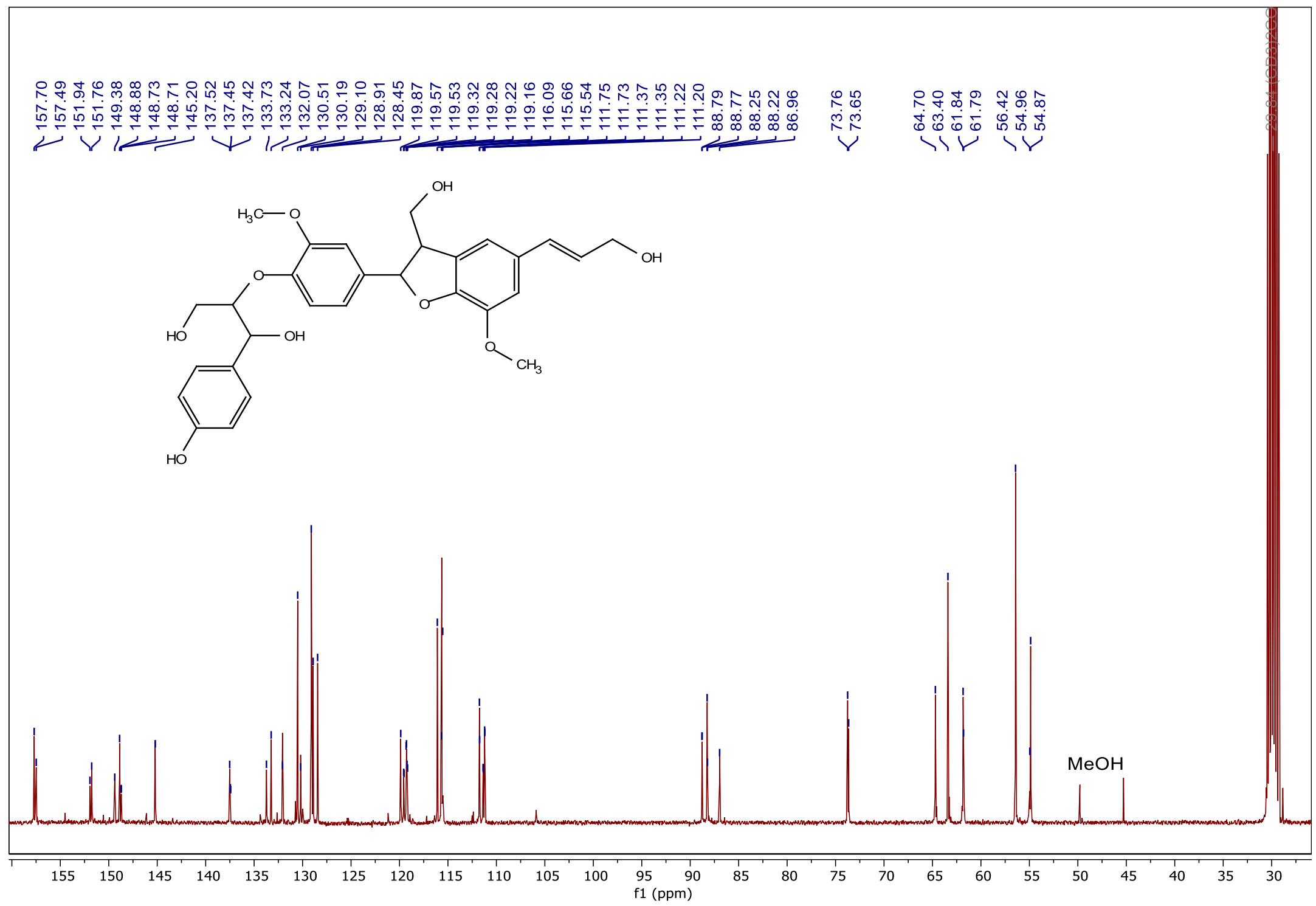

Figure S88. ^{13}C NMR (101 MHz, acetone-d_6) spectrum of **LMC6.**

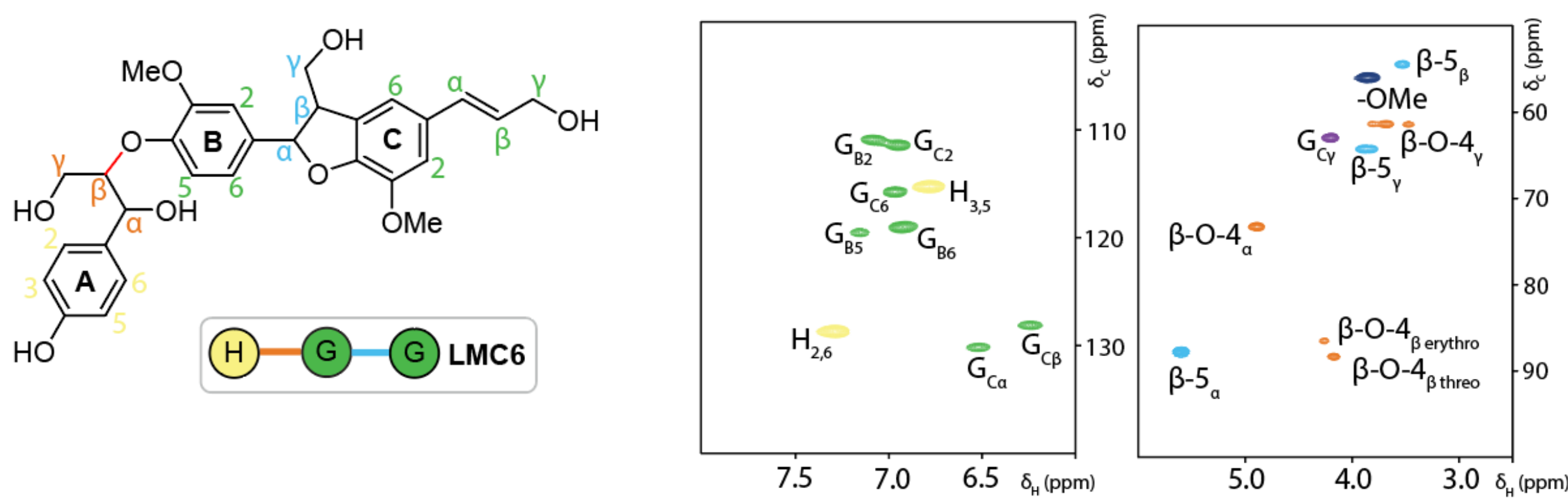

Figure S89. Aromatic and aliphatic regions of ^1H-^{13}C HSQC NMR spectra of **LMC6.**

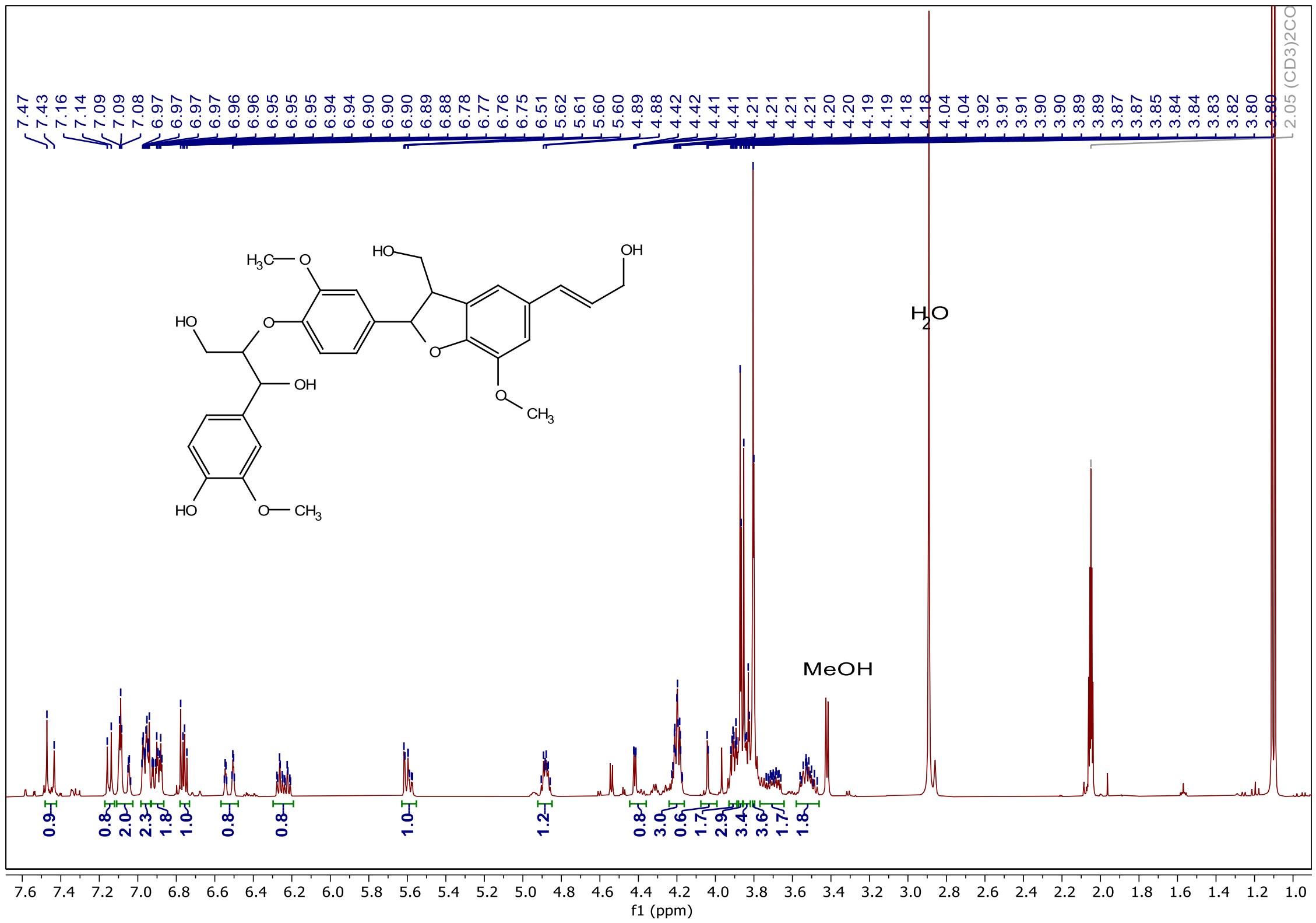

Figure S90. ^{1}H NMR (400 MHz, acetone-d_6) spectrum of **LMC7.**

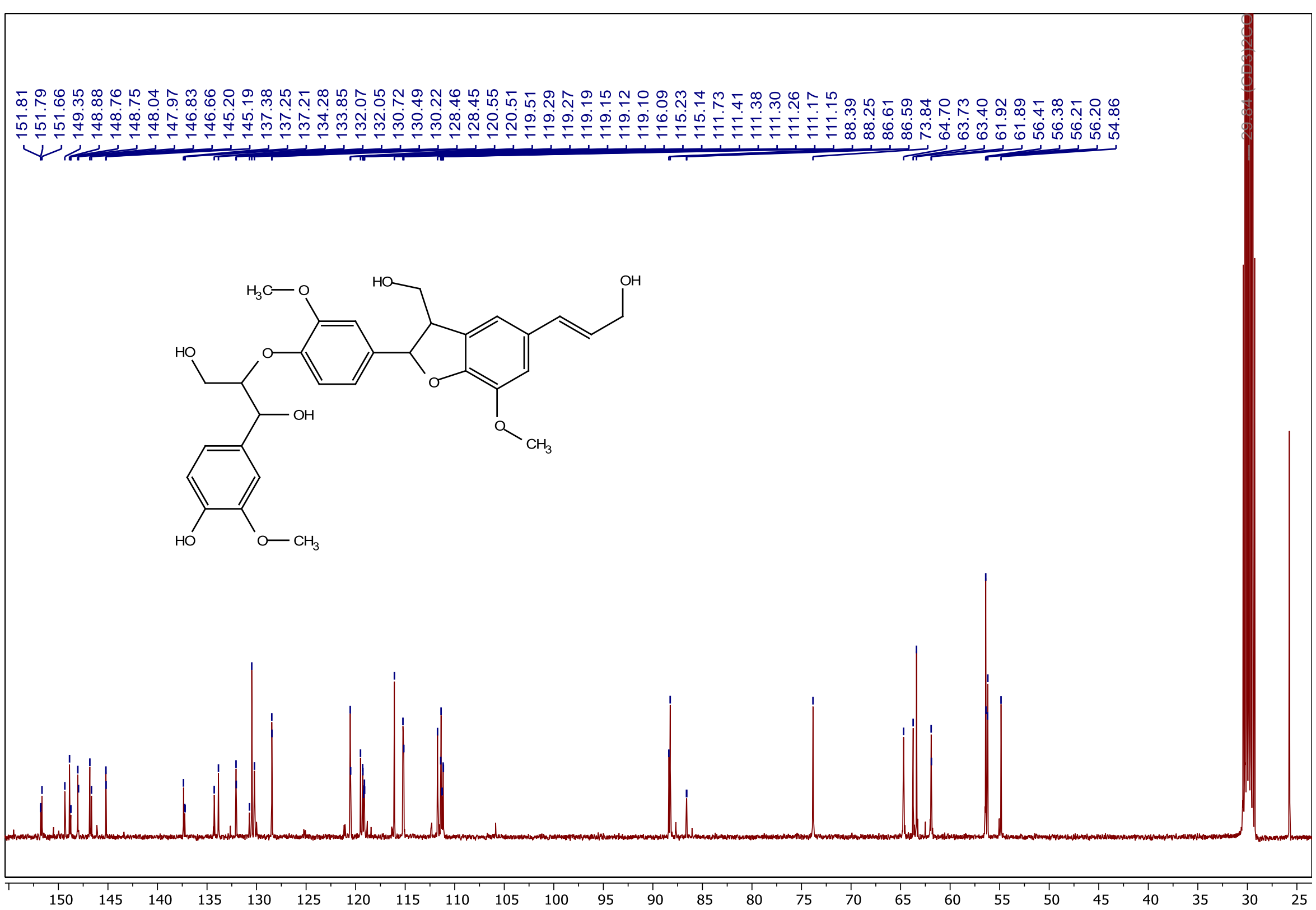

Figure S91. ^{13}C NMR (101 MHz, acetone-d_6) spectrum of **LMC7.**

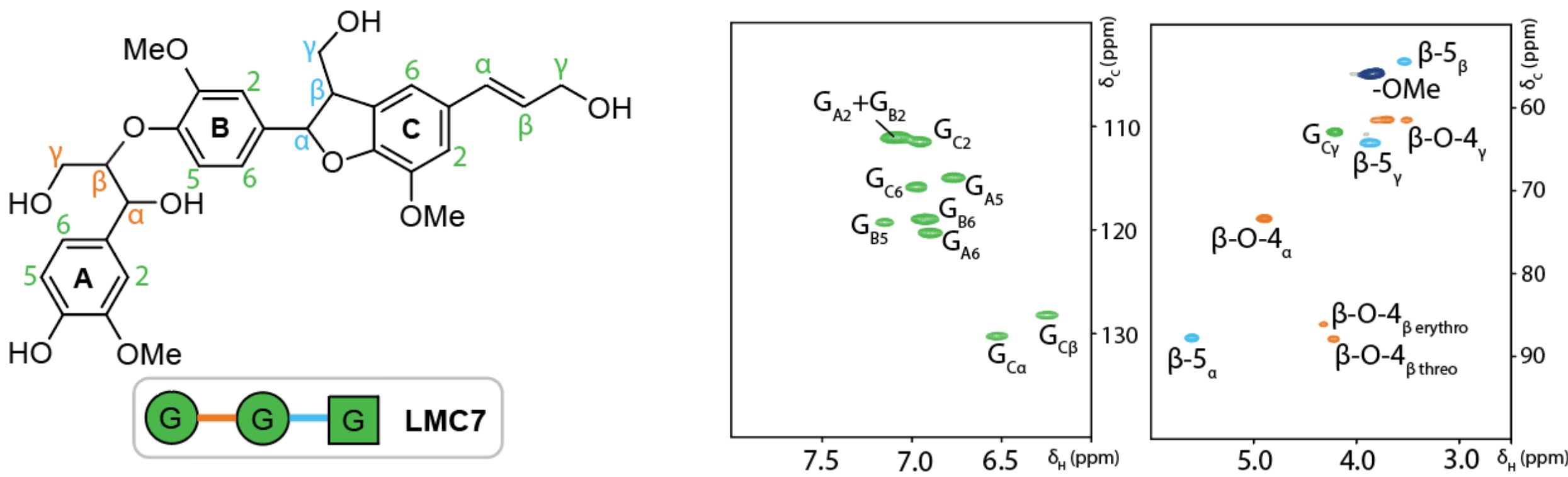

Figure S92. Aromatic and aliphatic regions of ^{1}H-^{13}C HSQC NMR spectra of **LMC7.** Also displayed in Figure 1 of the main manuscript.

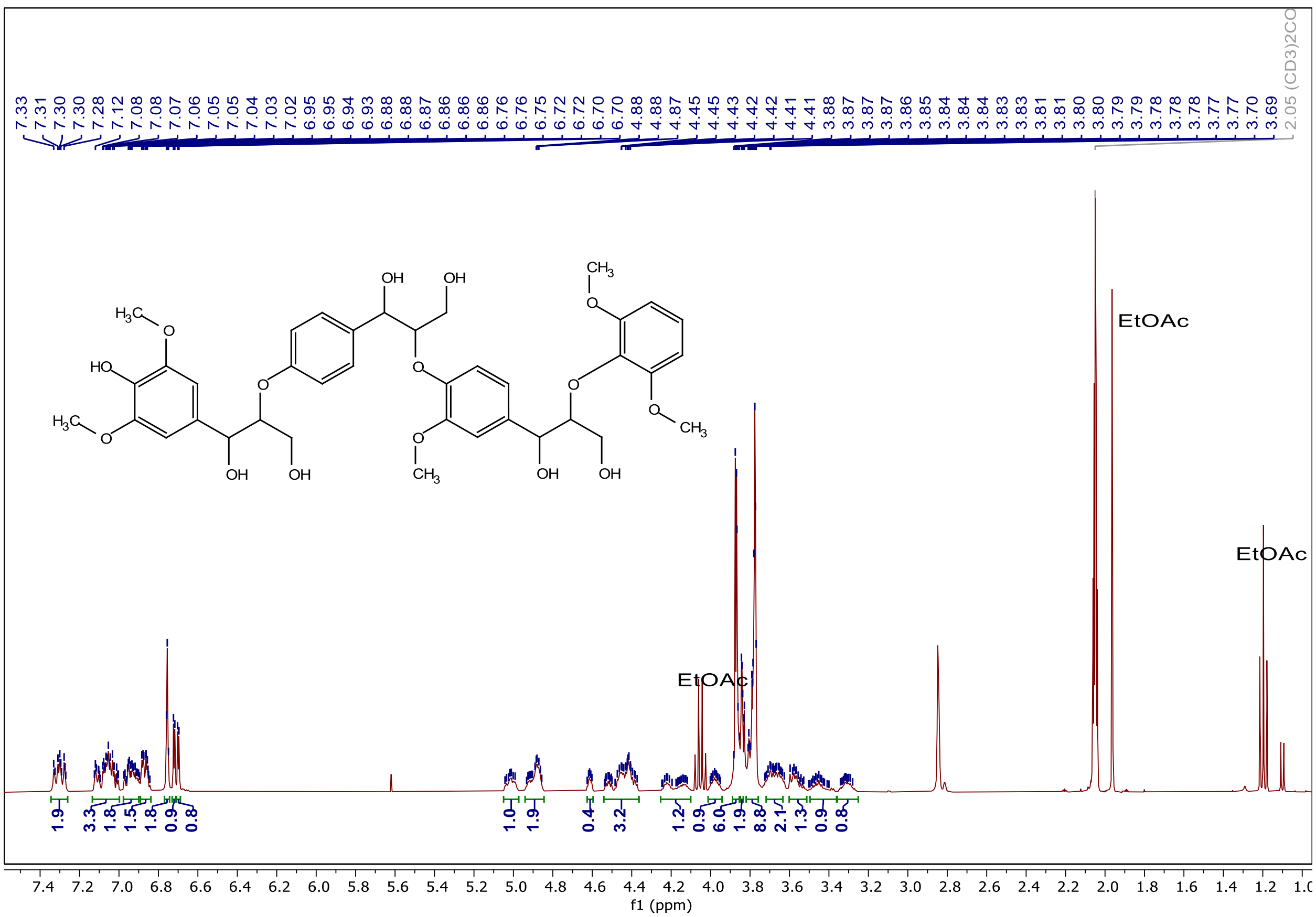

Figure S93. ^{1}H NMR (400 MHz, acetone-d$_6$) spectrum of **LMC8.**

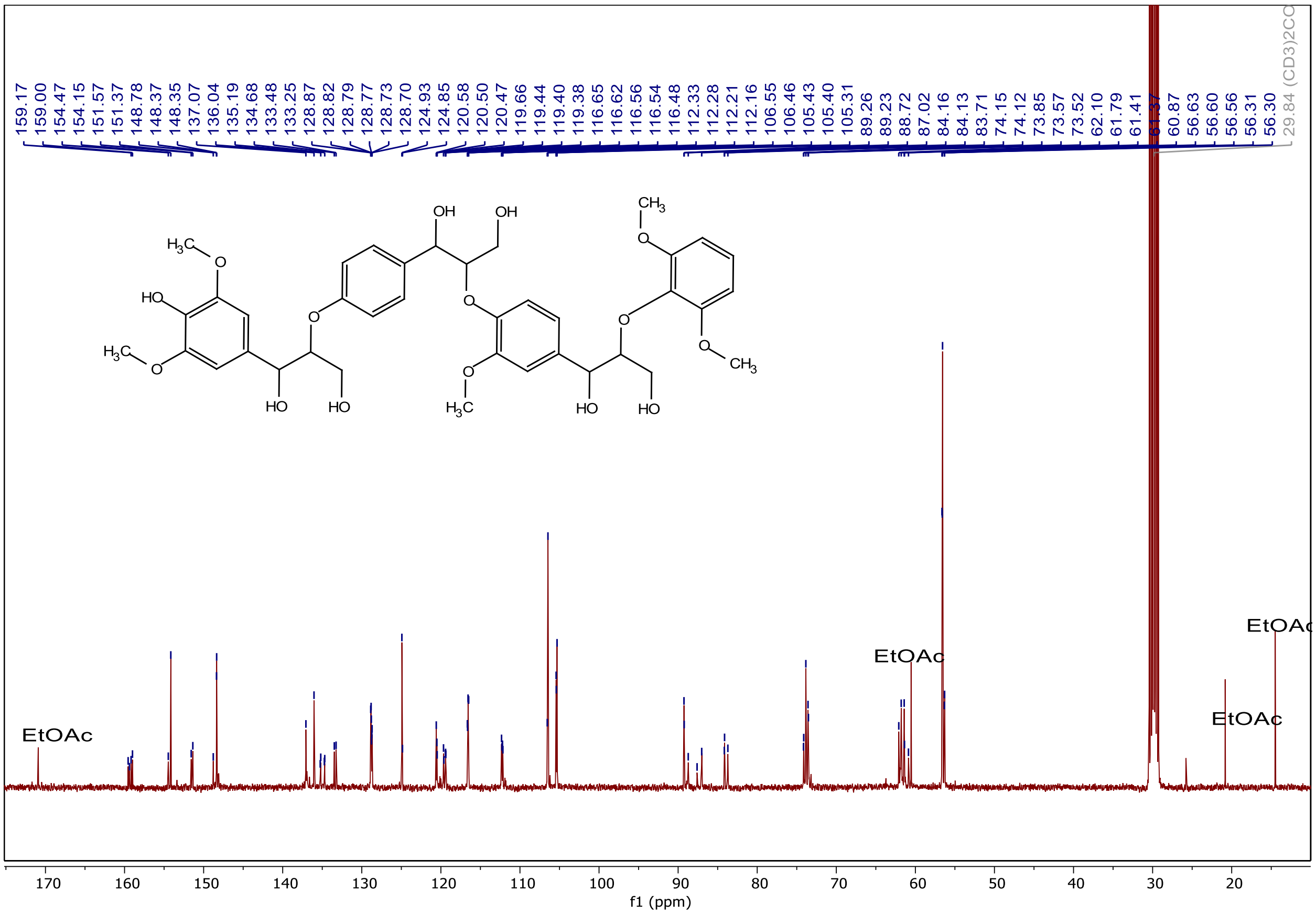

Figure S94. ^{13}C NMR (101 MHz, acetone-d_6) spectrum of **LMC8.**

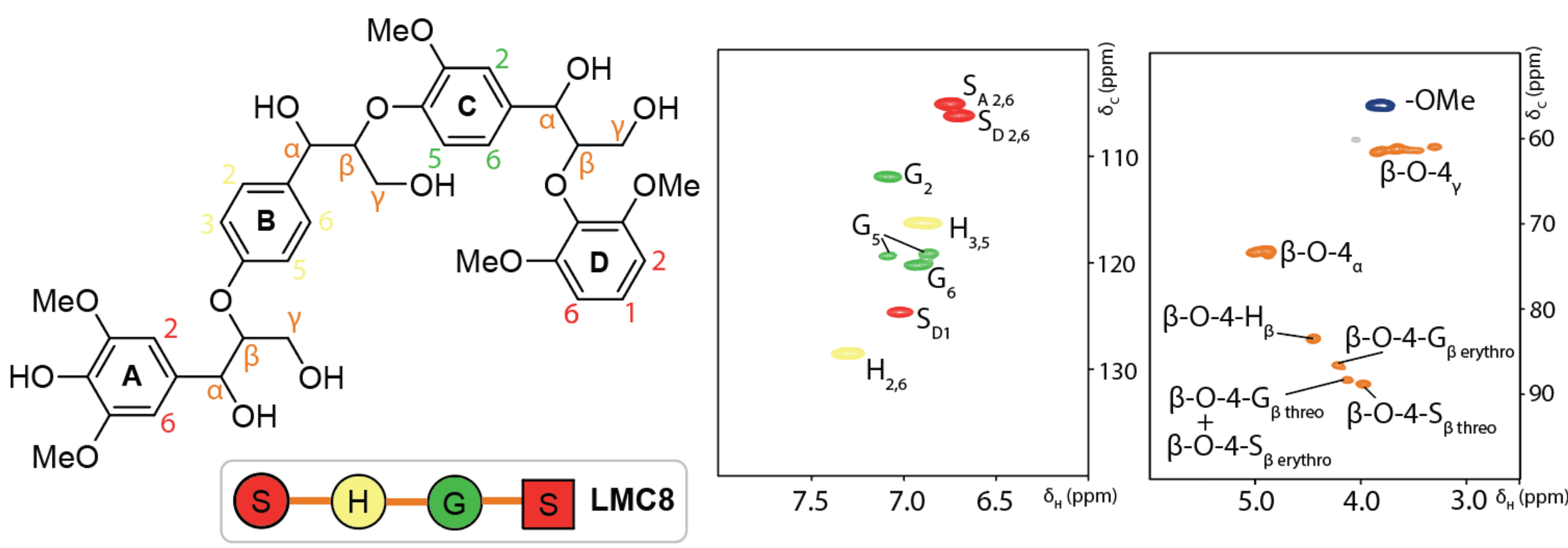

Figure S95. Aromatic and aliphatic regions of ^{1}H-^{13}C HSQC NMR spectra of **LMC8.**

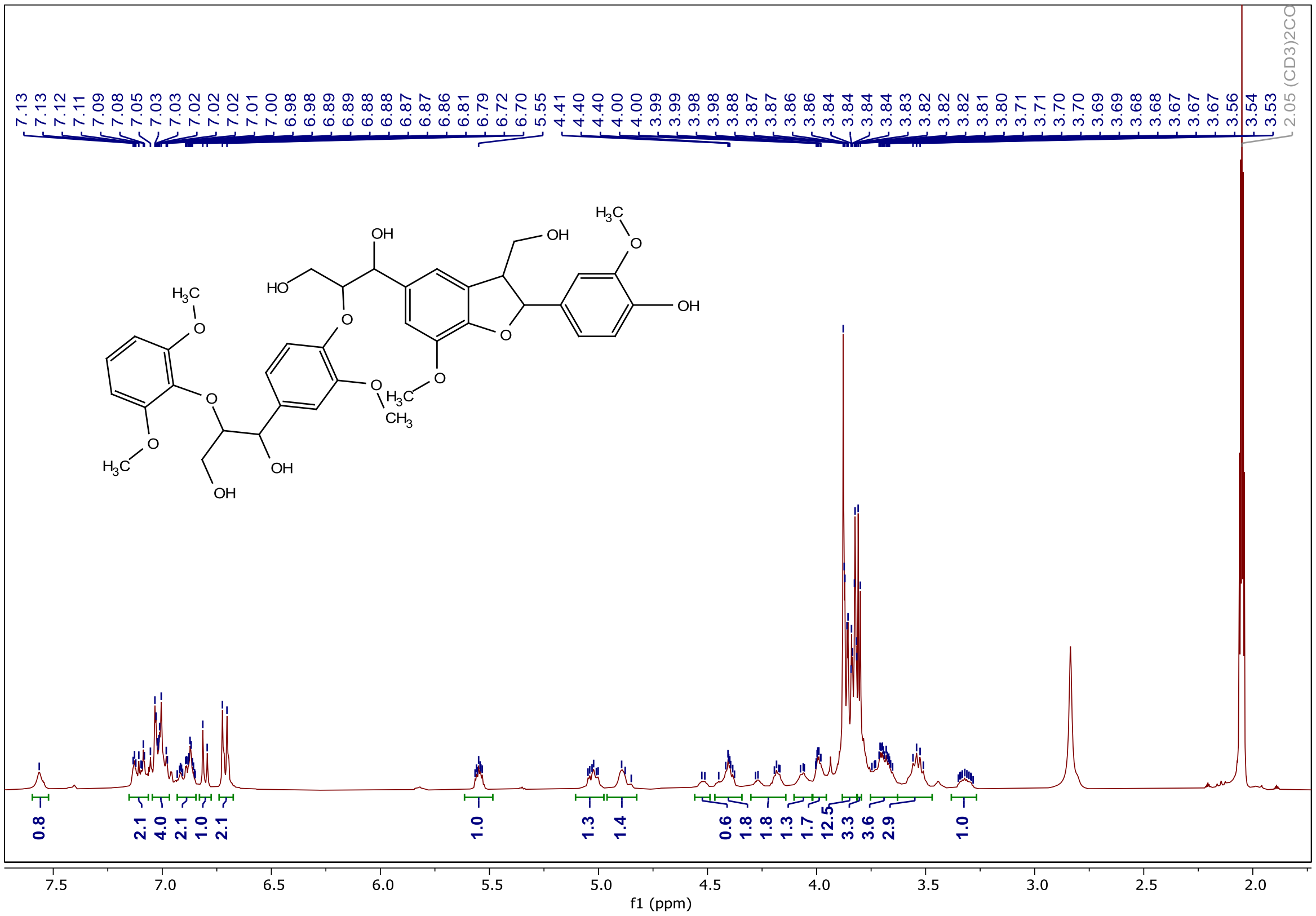

Figure S96. ^{1}H NMR (400 MHz, acetone-d_6) spectrum of **LMC9.**

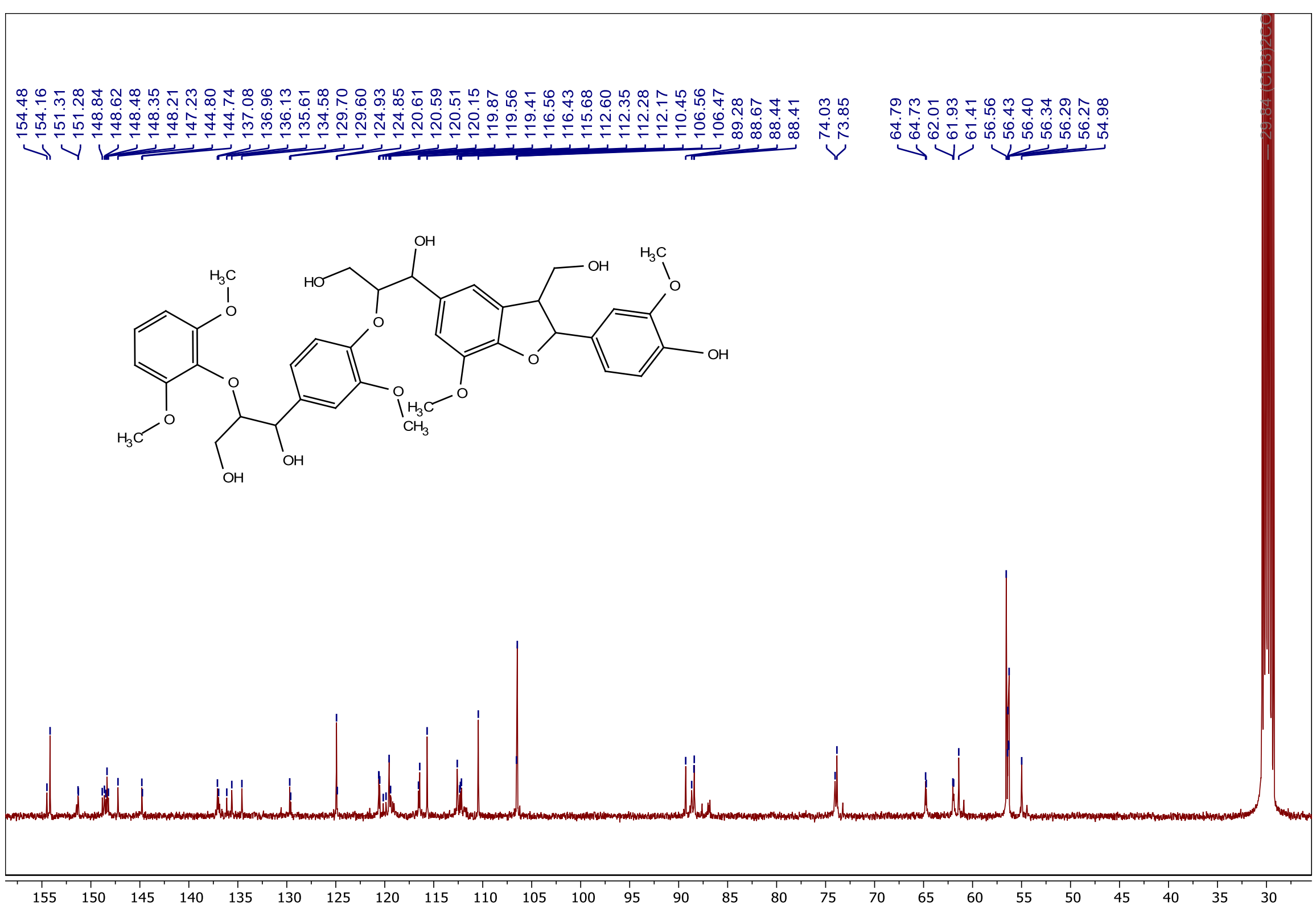

Figure S97. ^{13}C NMR (101 MHz, acetone-d_6) spectrum of **LMC9.**

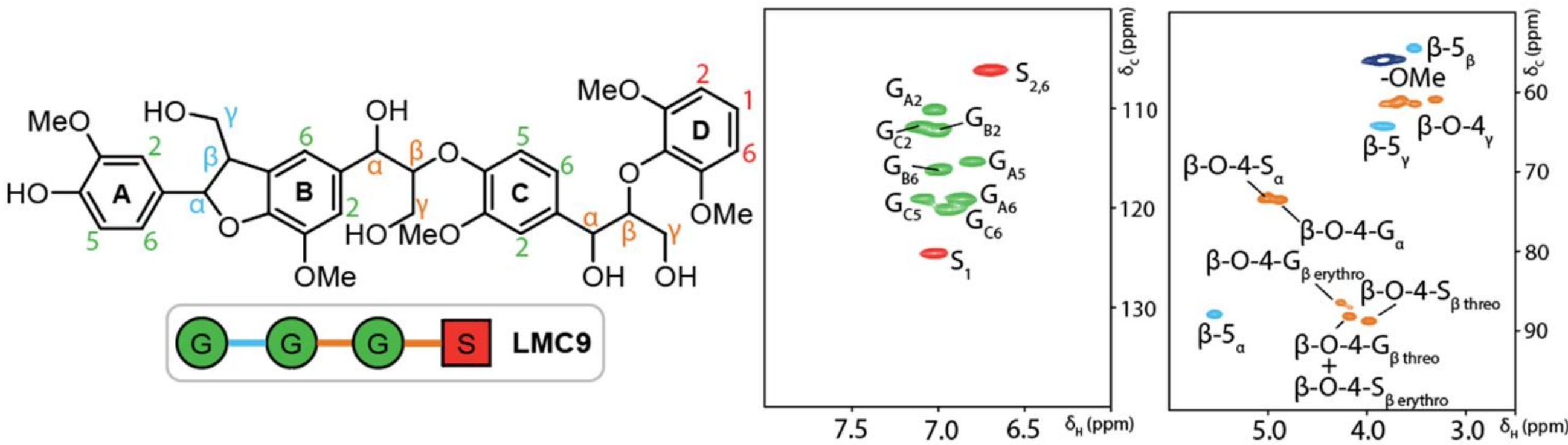

Figure S98. Aromatic and aliphatic regions of ^{1}H-^{13}C HSQC NMR spectra of **LMC9.** Also displayed in Figure 1 of the main manuscript.

References

[35] K. Hu, J.-H. Jeong, *Arch. Pharmacal Res.* **2006**, *29* (7), 563–565. DOI: 10.1007/BF02969265

[46] G. M. Sheldrick, *Acta Crystallogr. A: Found. Adv.* **2015**, *71* (Pt 1), 3–8. DOI: 10.1107/s2053273314026370

[47] G. M. Sheldrick, *Acta Crystallogr. C: Struct. Chem.* **2015**, *71* (Pt 1), 3–8. DOI: 10.1107/s2053229614024218

[48] O. V. Dolomanov, L. J. Bourhis, R. J. Gildea, J. A. K. Howard, H. Puschmann, *J Appl Crystallogr* **2009**, *42*, 339–341. DOI: 10.1107/S0021889808042726

[49] T. Ganesh, J. Jiang, M.-S. Yang, R. Dingledine, *J. Med. Chem.* **2014**, *57* (10), 4173–4184. DOI: 10.1021/jm5000672

[50] R. Ueda, T. Suzuki, K. Mino, H. Tsumoto, H. Nakagawa, M. Hasegawa, R. Sasaki, T. Mizukami, N. Miyata, *J. Am. Chem. Soc.* **2009**, *131* (48), 17536–17537. DOI: 10.1021/ja907055q

[51] K. R. Ganesh, K. R. Rao, T. V. Pratap, A. Raghunadh, S. P. Kumar, M. V. Basaveswara Rao, C. Murthy, S. B. Meruva, *Tetrahedron Lett.* **2016**, *57* (42), 4734–4736. DOI: https://doi.org/10.1016/j.tetlet.2016.09.041

[52] S. Pal, F. Lucarini, A. Ruggi, A. F. M. Kilbinger, *J. Am. Chem. Soc.* **2018**, *140* (9), 3181–3185. DOI: 10.1021/jacs.7b12805

[53] G. Xu, W. Min, Y. Zhongquan, L. Hongbin, D. Weihong, S. Mingwei, H. Yujiao, H. and Dong, *Synth. Commun.* **2021**, *51* (8), 1266–1271. DOI: 10.1080/00397911.2021.1879151

[54] C. Simon, C. Lion, C. Spriet, F. Baldacci-Cresp, S. Hawkins, C. Biot, *Angew. Chem. Int. Ed.* **2018**, *57* (51), 16665–16671. DOI: https://doi.org/10.1002/anie.201808493
